# Randomized trial of intermittent intraputamenal glial cell line-derived neurotrophic factor in Parkinson’s disease

**DOI:** 10.1093/brain/awz023

**Published:** 2019-02-26

**Authors:** Alan Whone, Matthias Luz, Mihaela Boca, Max Woolley, Lucy Mooney, Sonali Dharia, Jack Broadfoot, David Cronin, Christian Schroers, Neil U Barua, Lara Longpre, C Lynn Barclay, Chris Boiko, Greg A Johnson, H Christian Fibiger, Rob Harrison, Owen Lewis, Gemma Pritchard, Mike Howell, Charlie Irving, David Johnson, Suk Kinch, Christopher Marshall, Andrew D Lawrence, Stephan Blinder, Vesna Sossi, A Jon Stoessl, Paul Skinner, Erich Mohr, Steven S Gill

**Affiliations:** 1 Translational Health Sciences, Bristol Medical School, University of Bristol, Bristol, UK; 2 Neurological and Musculoskeletal Sciences Division, North Bristol NHS Trust, Bristol, UK; 3 MedGenesis Therapeutix Inc., Victoria, BC, Canada; 4 Renishaw plc, New Mills, Wotton-under-Edge, Gloucestershire, UK; 5 The Wales Research and Diagnostic Positron Emission Tomography Imaging Centre (PETIC), Cardiff University, Cardiff, UK; 6 School of Psychology, Cardiff University, Cardiff, UK; 7 Department of Physics and Astronomy, The University of British Columbia, Vancouver, BC, Canada; 8 Djavad Mowafaghian Centre for Brain Health, Faculty of Medicine, The University of British Columbia, Vancouver, BC, Canada

**Keywords:** glial cell line-derived neurotrophic factor, GDNF, convection-enhanced delivery, Parkinson’s disease, neurorestoration

## Abstract

We investigated the effects of glial cell line-derived neurotrophic factor (GDNF) in Parkinson’s disease, using intermittent intraputamenal convection-enhanced delivery via a skull-mounted transcutaneous port as a novel administration paradigm to potentially afford putamen-wide therapeutic delivery. This was a single-centre, randomized, double-blind, placebo-controlled trial. Patients were 35–75 years old, had motor symptoms for 5 or more years, and presented with moderate disease severity in the OFF state [Hoehn and Yahr stage 2–3 and Unified Parkinson’s Disease Rating Scale motor score (part III) (UPDRS-III) between 25 and 45] and motor fluctuations. Drug delivery devices were implanted and putamenal volume coverage was required to exceed a predefined threshold at a test infusion prior to randomization. Six pilot stage patients (randomization 2:1) and 35 primary stage patients (randomization 1:1) received bilateral intraputamenal infusions of GDNF (120 µg per putamen) or placebo every 4 weeks for 40 weeks. Efficacy analyses were based on the intention-to-treat principle and included all patients randomized. The primary outcome was the percentage change from baseline to Week 40 in the OFF state (UPDRS-III). The primary analysis was limited to primary stage patients, while further analyses included all patients from both study stages. The mean OFF state UPDRS motor score decreased by 17.3 ± 17.6% in the active group and 11.8 ± 15.8% in the placebo group (least squares mean difference: −4.9%, 95% CI: −16.9, 7.1, *P = *0.41). Secondary endpoints did not show significant differences between the groups either. A *post hoc* analysis found nine (43%) patients in the active group but no placebo patients with a large clinically important motor improvement (≥10 points) in the OFF state (*P = *0.0008). ^18^F-DOPA PET imaging demonstrated a significantly increased uptake throughout the putamen only in the active group, ranging from 25% (left anterior putamen; *P = *0.0009) to 100% (both posterior putamina; *P < *0.0001). GDNF appeared to be well tolerated and safe, and no drug-related serious adverse events were reported. The study did not meet its primary endpoint. ^18^F-DOPA imaging, however, suggested that intermittent convection-enhanced delivery of GDNF produced a putamen-wide tissue engagement effect, overcoming prior delivery limitations. Potential reasons for not proving clinical benefit at 40 weeks are discussed.

## Teaser

Trials of GDNF in Parkinson’s disease have yielded inconsistent results. In a randomised controlled trial, Whone *et al.* administer GDNF using a paradigm designed to optimize delivery to the putamen. [^18^F]DOPA PET reveals putamen-wide uptake, but GDNF does not differ from placebo in its effects on motor function.

## Introduction

Parkinson’s disease is the second most common neurodegenerative disorder (de Lau and Breteler, 2006). Current treatment options are symptomatic and do not prevent disease progression. Over time, patients accrue both motor and cognitive disability and develop complications of dopaminergic therapies (Rascol *et al.*, 2011).

Glial cell line-derived neurotrophic factor (GDNF) is a potent neurotrophic protein for dopaminergic and other neurones (Lin *et al.*, 1993; Airaksinen and Saarma, 2002) and shows robust neurorestorative and neuroprotective effects in nonhuman primate models of Parkinson’s disease when administered to targets within the CNS (Gash *et al.*, 1996; Zhang *et al.*, 1997; Grondin *et al.*, 2002; Allen *et al.*, 2013). In patients with Parkinson’s disease, intracerebroventricular administration of GDNF did not show clinical benefit (Kordower *et al.*, 1999; Nutt *et al.*, 2003). In two open-label studies using continuous intraputamenal infusion, GDNF substantially improved motor function at 6 and 12 months (Gill *et al.*, 2003; Slevin *et al.*, 2005), associated with a focal increase in ^18^F-DOPA uptake at the site of infusion in posterior putamen (Gill *et al.*, 2003). Moreover, single-case reports suggested dopaminergic sprouting (Love *et al.*, 2005) and clinical benefit years beyond end of treatment (Patel *et al.*, 2013). The favourable clinical outcome, however, could not be replicated in a randomized, placebo-controlled study using a similar infusion scheme over 6 months (Lang *et al.*, 2006).

The observed outcome discrepancies were possibly due to insufficient GDNF exposure across the putamen, since continuous low-rate infusions enable only diffusion-dependent, irregular (heterogeneous), spatially restricted distribution (Salvatore *et al.*, 2006). Much wider, homogeneous distribution can be achieved with convection-enhanced delivery (CED) which, however, requires high infusion rates that in turn necessitate intermittent rather than continuous administration to avoid tissue ‘flooding’ (Gimenez *et al.*, 2011). The above, together with recent reports on striatal pharmacokinetics and pharmacodynamics of GDNF (Hadaczek *et al.*, 2010; Taylor *et al.*, 2013), was the rationale for conducting a randomized, placebo-controlled, study of GDNF, administered on an intermittent (every 4 weeks) basis, in a manner to achieve CED across the putamen. The present study was the first clinical study worldwide to evaluate the effects of GDNF (or any other drug) when given via intermittent (every 4 weeks), bilateral, intraputamenal CED.

Dosing in the context of intermittent CED is complex and involves several dimensions, including infusion rate, infusion volume, drug concentration, dosing interval and total (or cumulative) dose over time. The infusion rate and volume were chosen to achieve intraputamenal CED. The intermittent dosing information available at the beginning of the study was mostly limited to reports on striatal pharmacokinetics and pharmacodynamics of GDNF in rats (Hadaczek *et al.*, 2010; Taylor *et al.*, 2013). The infusate GDNF concentration administered was 2-fold higher than in the Lang investigation (0.2 µg/µl versus 0.1 µg/µl) (Lang *et al.*, 2006); however, moving from continuous to every 4-week intermittent dosing means the total dose delivered over 4 weeks (240 µg) is 3.5-fold smaller than the dose most widely used in the historic continuous dosing studies (840 µg). Previously, unexpected clinically silent cerebellar toxicity was observed when very high GDNF doses (2800 µg/4 weeks, translating to a human equivalent dose of 42 000 µg/4 weeks) were given via continuous intraputamenal infusion in a 6-month toxicity study in rhesus monkeys (Hovland *et al.*, 2007). Therefore, to ensure patient safety, the GDNF dosing scheme used in the present study was purposefully low. The associated risk of underdosing was acknowledged but deemed acceptable. Additional dose groups were not feasible financially and logistically in this single-centre investigator-initiated study.

Because of a lack of commercially-available drug delivery devices that would facilitate intermittent infusions to the brain parenchyma over an extended time period, a novel in-house system was specified by the lead neurosurgeon (Lewis *et al.*, 2016). Most importantly, the system included a skull-mounted transcutaneous port allowing for non-invasive repeat infusions via four separate microcatheters. Although a first-in-man device in its entirety, key device performance features and device attributes such as long-term catheter patency, controlled infusions and device safety had been developed previously (Bienemann *et al.*, 2012; Barua *et al.*, 2013, 2016; Gill *et al.*, 2013).

Altogether, the study entered uncharted territory in several areas. The study results, while of primary relevance to GDNF and Parkinson’s disease, were considered potentially useful for other applications and indications where direct, targeted drug delivery to brain parenchyma could be beneficial. The primary hypothesis being tested was that GDNF, if administered in a manner to permit CED across the putamen, would achieve neurorestoration leading to clinically significant benefit.

## Materials and methods

### Study design and structure

This single-centre, placebo-controlled, randomized, double-blind, parallel-group trial of intermittent bilateral intraputamenal infusions of GDNF administered via CED was performed in two stages. The pilot stage (*n = *6) served to assess the safety of the surgical technique and study drug administration, and to optimize planned study procedures. The primary stage (*n = *35) was initiated upon completion of a prespecified safety review of the pilot patients after 12 weeks of treatment. All patients randomized and completing study treatment after 40 weeks had the option to enrol in a subsequent open-label extension study which will be reported separately.

Blocked, web-based randomization with a block size of six was used to randomize patients between GDNF and diluent, artificial CSF, which served as placebo. Randomization was performed by the Bristol Randomised Trials Collaboration, University of Bristol, at a site separate to the investigating site. The randomization ratio was 2:1 in the pilot stage and 1:1 in the primary stage. Patients and investigators were masked to treatment allocation. Ready-to-use preparations of GDNF and artificial CSF were visually identical. To further protect against bias, motor scoring was performed by trained raters blinded to all other aspects of the patient’s condition, and GDNF plasma concentrations and anti-GDNF serum antibodies were assayed only after the study was completed.

Local institutional and ethical committee approval was obtained and all patients provided written informed consent according to the Declaration of Helsinki. The Trial Steering Committee and an independent Data Monitoring Committee provided clinical oversight. The authors vouch for the accuracy and completeness of the data and for adherence to the study protocol (see Supplementary material, part A, for study protocol first and final versions as well as a summary of protocol amendments).

### Patients

Between October 2012 and April 2015, 196 subjects from throughout the UK were prescreened, of whom 64 patients with bilateral idiopathic Parkinson’s disease according to the UK Brain Bank Criteria underwent full study screening (see Supplementary material, part B, for CONSORT flow diagram). All study visits were performed at North Bristol Trust, Bristol, UK (Frenchay Hospital site until May 2014, Southmead Hospital site thereafter), except for the PET scans, which were acquired at the Wales Research and Diagnostic PET Imaging Centre, Cardiff, UK and analysed at the PET Imaging Centre of the University of British Columbia, Vancouver, BC, Canada.

Patients were eligible for implantation surgery if they were 35 to 75 years old, on stable anti-parkinson medication for ≥6 weeks and presented with motor symptom duration ≥5 years, moderate disease severity [Hoehn and Yahr stage 2–3 and Unified Parkinson’s Disease Rating Scale (UPDRS) motor score (part III) between 25 and 45, both in a practically-defined OFF state], motor fluctuations (average of at least 2.5 h of OFF time per day on 3-day fluctuation diaries), and levodopa responsiveness defined as ≥40% improvement in UPDRS motor score following a levodopa challenge. Main exclusion criteria were: atypical parkinsonian syndromes, family history of >1 first-degree relative with Parkinson’s disease, moderate depression (Beck Depression Inventory >20), clinically significant impulse control disorder, cognitive decline [Montreal Cognitive Assessment (MoCA) <24], and increased risk of surgery. Once implanted with the drug delivery system, patients were randomized if they had no relevant sequelae from surgery and demonstrated ≥40% coverage of a predefined volume of interest in the motor putamen on a gadolinium-enhanced test infusion.

### Study procedures and assessments

**Figure 1:**
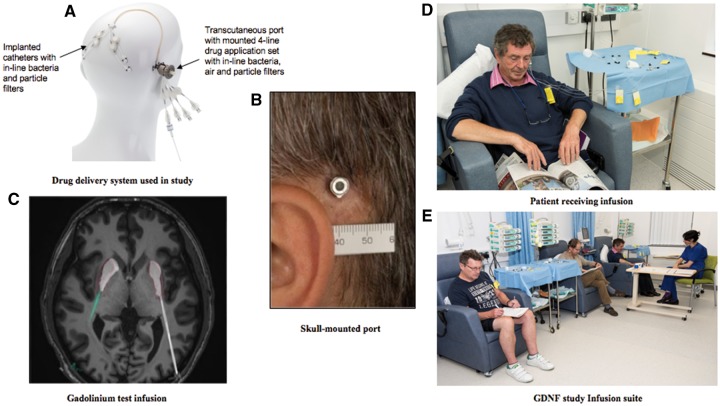
**Method of drug administration.** (**A**) Drug delivery system used in the study. A manikin view of the delivery system is shown. Externally, when infusions are delivered, a titanium application set is attached to a skull-mounted port. The application set houses four independent external lines that feed back to four independent B. Braun pumps (not shown) for the administration of GDNF or placebo. Internally, from the skull mounted port, run four independent catheters. (**B**) The skull-mounted port is the only external component when the patient is not receiving an infusion. (**C**) Gadolinium test infusion. An axial MRI section at the level of the striatum is shown. Two of the four catheters (dorsal two catheters) can be seen entering either side of the brain posteriorly to penetrate the putamen. T_1_ imaging has been acquired post a test infusion of 2 mM gadolinium down the catheters and into each putamen. Gadolinium can be seen distributed through both putamen from the rostral to caudal extent. (**D**) A single patient during infusion. (**E**) Three patients receiving their monthly intraputamenal infusions from B. Braun pumps via their skull-mounted ports, in a standard day-case facility, observed by an accompanying nurse.

After screening, eligible patients underwent robot-assisted surgery for stereotactic implantation of the customized in-house CED system comprising four separate infusion catheters and a single skull-mounted transcutaneous port (Fig. 1A and B; for device background summary and patient infusion images, see Supplementary material, parts C and D, respectively). Four weeks post-operatively, catheter patency and infusate distribution were assessed by T_1_-weighted MRI following an intraputamenal test infusion of 2 mM solution of gadolinium in artificial CSF (Fig. 1C). If sufficient (≥40%) volume of interest coverage was confirmed on the MRI scan, patients proceeded to randomization.

Post-randomization, patients received a total of 10 study treatments at 4-week intervals (Weeks 0 to 36). At each treatment, 400 µl of infusate (300 µl GDNF or placebo, followed by 100 µl artificial CSF) were delivered per catheter. The infusate GDNF concentration was 0.2 µg/µl, and the total GDNF dose given every 4 weeks was 240 µg (120 µg/putamen).

The protocol stated that Parkinson’s disease medication was to be kept stable during the study where possible but could be modified if required for symptom control.

Every 8 weeks post-randomization, starting with the baseline visit at Week 0, prior to infusions, patients completed 3-day diary recordings and underwent assessment of motor function in the practically defined OFF state and following a levodopa challenge. Other efficacy outcome measures were assessed at baseline and Week 40. Samples for GDNF plasma concentrations and anti-GDNF serum antibodies were collected throughout the study. At Week 40, all patients underwent a repeat test infusion of gadolinium-enhanced artificial CSF followed by T_1_-weighted MRI to determine maintenance of infusate delivery.

All patients underwent baseline ^18^F-DOPA PET scanning between randomization and baseline assessments prior to the start of treatment (see Supplementary material, part E, for PET methodology). A further ^18^F-DOPA PET scan was performed at the end of the study, 2 weeks after the last study infusion.

### Study outcomes

The primary endpoint of the study was percentage change from baseline in the practically-defined OFF state UPDRS motor score (part III) after 40 weeks of double-blind treatment.

Secondary endpoints included percentage change from baseline to Week 40 in UPDRS motor score in the ON state, as well as percentage change from baseline in UPDRS activities of daily living (ADL; part II) and total (sum of motor and ADL) scores in the OFF and ON state, UPDRS parts I and IV, and change from baseline to Week 40 in Parkinson’s disease diary ratings. Supplementary efficacy endpoints included timed motor tests in both OFF and ON state, total daily levodopa and levodopa equivalent dose, the Non-Motor Symptom Scale for Parkinson’s disease (NMSS), cognitive, mood and impulsivity measures, the University of Pennsylvania Smell Identification Test (UPSIT) and Parkinson-related quality of life questionnaires (PDQ-39 and EQ-5D). Patients’ satisfaction and impact on quality of life in relation to the delivery device were not specifically explored.

Post-screening, assessment of UPDRS motor and ADL scores, timed taps and timed walks were completed by three trained raters who were blinded to all other aspects of the patient’s condition. Wherever possible, the same rater that performed the baseline assessment also performed the Week 40 assessment. All OFF assessments were performed at a similar time in the morning, following withholding of long-acting anti-Parkinson medications the day before and all other anti-Parkinson’s disease medications from 6 pm the evening before.

During screening, patients were trained on the completion of the Parkinson’s disease diary and had to demonstrate their ability to accurately determine their ON/OFF state as part of the inclusion criteria.

Imaging endpoints included change from baseline to Week 40 in gadolinium-evidenced volume of infusate distribution, volume of interest coverage and total putamenal coverage as assessed on T_1_-weighted MRIs, and in ^18^F-DOPA uptake, as well as correlations between clinical outcome, gadolinium-based coverage and change in ^18^F-DOPA uptake.

Safety was assessed on the basis of adverse events, routine laboratory testing and anti-GDNF serum antibodies. Treatment-emergent adverse events (TEAEs) were defined as adverse events starting on or after the date of the first dose of randomized study medication. Dyskinesias, falls, adverse changes in mood, and impulsivity were summarized as TEAEsof special interest. In addition, patients were monitored for cognitive function (MoCA and Mattis Dementia Rating Scale) and signs of impulsive or compulsive behaviour (Questionnaire for Impulsive-Compulsive Disorders in Parkinson’s disease).

### Statistical analysis

Statistical analyses, as prespecified in the statistical analysis plan (see Supplementary material, part F, for first and final versions of statistical analysis plan as well as a summary of statistical analysis plan amendments), were conducted with the use of Statistical Analysis System (SAS) software, version 9.4 (SAS Institute). Any hypothesis testing was performed with a 2-sided alternative at an alpha level of 0.05. No adjustments for multiplicity were made. Sample size was calculated on the basis of the primary endpoint, assuming a standard deviation (SD) of 20%, a 2-sided type I error of 5%, a power of 80%, and a difference of 20 points in percentage change in OFF state UPDRS motor score from baseline.

Efficacy analyses were based on the intention-to-treat principle and included patients randomized after reaching prespecified post-surgical eligibility criteria. As the pilot stage served to optimize planned study procedures, it was anticipated *a priori* that relevant differences between patients at the two stages might emerge during the study. Therefore, the primary analysis was limited to primary stage patients (*n = *35). Further prespecified efficacy analyses were also performed on all patients from both study stages (*n = *41). A mixed-effect model with repeated measures (MMRM) adjusted for the baseline value was used for the comparison of treatment groups in the primary analysis. Sensitivity analyses of the primary endpoint also included analyses of covariance (ANCOVA) instead of the MMRM. Secondary and supplementary efficacy endpoints were analysed using either the MMRM or an ANCOVA model adjusted for the baseline value of the respective assessment. Non-parametric Spearman rank correlation analyses were used to test for potential correlations between clinical endpoints and imaging endpoints, and between MRI and PET imaging endpoints. No corrections for multiple comparisons were made, and a hierarchical approach to the secondary endpoints was not employed.

Safety was generally evaluated in the overall population including patients from both study stages to make maximum use of the available safety information post-randomization. Additional safety analyses were performed to evaluate the occurrence of adverse events in the peri-surgical period prior to both randomization and start of treatment.

A *post hoc* analysis plan was devised after the final study results became available (Supplementary material, part G). It included analyses of UPDRS motor score subscales, magnitude of motor response, phenotypic covariates and additional imaging endpoints in the overall population. Again, no corrections for multiple comparisons were made.

### Data availability

The data that support the findings of this study are available from the corresponding authors, upon reasonable request.

## Results

### Patients

The 35 primary stage patients had a mean (±SD) age of 56.4 ± 7.9 years (range: 41–72), and a mean disease duration since first symptom of 10.9 ± 5.3 years. Except for gender, other demographic and baseline Parkinson’s disease characteristics were similar between treatment groups (Table 1).

**Table 1:** Demographic and Parkinson’s disease characteristics at screening

Characteristic	GDNF (*n = *17)	Placebo (*n = *18)
Age, years	57.7 ± 8.2	55.1 ± 7.5
Male sex, *n* (%)	7 (41.2)	11 (61.1)
Race, *n* (%)		
White	17 (100)	17 (94.4)
Asian	0	1 (5.6)
OFF-state Hoehn and Yahr stage, *n* (%)		
Stage 2	8 (47.1)	5 (27.8)
Stage 2.5	4 (23.5)	8 (44.4)
Stage 3	5 (29.4)	5 (27.8)
Disease duration, years		
Since first motor symptom	10.8 ± 5.0	10.9 ± 5.8
Since original diagnosis	8.6 ± 4.3	7.9 ± 3.7
UPDRS motor score		
OFF state	37.1 ± 7.2	35.8 ± 6.1
ON state	16.9 ± 5.2	16.9 ± 4.5
Levodopa response, %^a^	54.2 ± 9.4	52.8 ± 9.4
OFF-time per day, h	6.3 ± 2.2	6.1 ± 2.1

^a^Percentage improvement in UPDRS motor score following a levodopa challenge.

### Drug delivery

Catheters were positioned accurately with a mean distance between planned and actual target for catheter tips of 0.6 ± 0.5 mm (range: 0.0–2.0 mm). Mean putamenal gadolinium-evidenced coverage on MRI showed only small differences between hemispheres, treatment groups and time points. Between all of these variables, it ranged from 67.1 ± 15.3% to 78.5 ± 14.2% for the putamenal volume of interest and from 47.8 ± 13.5% to 55.0 ± 17.1% for total putamen.

With 347 (99.1%) of 350 scheduled study drug infusions administered, compliance with infusion visits over the study period was high. Altogether, 9 (5.4%) of 167 GDNF infusions and 10 (5.6%) of 180 placebo infusions were interrupted or terminated early. Misalignment of the application set connector to the skull-mounted port was thought to account for early termination of four infusions in each group. The remaining interruptions or early terminations were typically related to a single catheter and nearly always occurred as an automatic safety pump shut-down response to transient high catheter pressure, which did not translate to any adverse events for the participants. Two occluded infusion channels were identified at the test infusion stage, in response to which a double volume dose was then prescribed down the ipsilateral putamenal catheter for all study infusions in those two subjects, in line with the study protocol.

### Clinical outcomes

**Figure 2:**
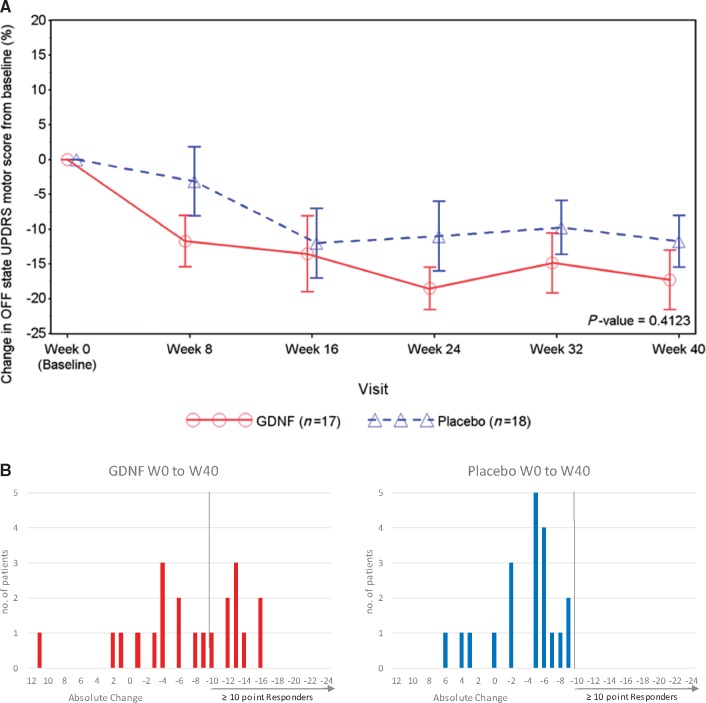
**OFF state UPDRS motor score.** (**A**) OFF state UPDRS motor score: percentage change over time. Note that data points represent means, and error bars represent standard errors. One GDNF patient had a conus injury due to a car accident and was included in the motor score without items 22, 27, 28, 29, and 30. The *P*-value is from a MMRM for the percentage change from baseline to Week 40 between treatment groups. (**B**) Frequency distribution of change from baseline to Week 40 in OFF state UPDRS motor score (intention-to-treat overall population = primary stage and pilot stage patients, *n = *41).

Between baseline and Week 40, mean OFF state UPDRS motor scores decreased by 17.3 ± 17.6% (6.2 ± 7.1 absolute points, from 35.3 ± 9.4 to 29.1 ± 10.3 points) in the GDNF group and 11.8 ± 15.8% (3.4 ± 4.3 absolute points, from 32.2 ± 8.7 to 28.8 ± 9.8 points) in the placebo group, with no statistically significant mean treatment difference in favour of GDNF at any of the 8-weekly time points during the study (least squares mean difference: −4.9%, 95% CI: −16.9, 7.1, *P = *0.41; Table 2 and Fig. 2A). None of the sensitivity analyses showed a statistically significant treatment effect in favour of GDNF, which included an assessment of the primary endpoint using the overall population from both study stages (*n = *41; least squares mean difference: 8.4%, 95% CI: −19.3, 2.5; *P = *0.13). No patients dropped out or were excluded post randomization.

**Table 2:** Efficacy outcomes

Outcome category Variable	GDNF (*n = *17)			Placebo (*n = *18)			Least squares mean difference versus placebo (95% CI); *P*
	Baseline	Week 40	Change, (%)	Baseline	Week 40	Change, (%)	
**UPDRS (part) scores**							
Motor (III) OFF	35.3 ± 9.4	29.1 ± 10.3	−17.3 ± 17.6	32.2 ± 8.7	28.8 ± 9.8	−11.8 ± 15.8	−4.9% (−16.9, 7.1); 0.41*
Motor (III) ON	17.4 ± 5.0	16.3 ± 6.3	−4.3 ± 33.4	16.6 ± 7.5	17.8 ± 8.4	8.8 ± 21.7	−12.2% (−31.6, 7.1); 0.21*
ADL (II) OFF	18.4 ± 6.3	16.0 ± 7.0	−12.2 ± 26.9	16.9 ± 6.1	16.2 ± 5.5	−1.0 ± 26.6	−9.3% (−27.8, 9.2); 0.31*
ADL (II) ON	6.3 ± 4.2	6.3 ± 4.0	30.4 ± 109.9	5.7 ± 3.6	5.8 ± 4.1	−1.5 ± 49.4	33.1% (−24.2, 90.4); 0.25*
Total (II+III) OFF	54.3 ± 13.8	45.9 ± 15.3	−15.2 ± 16.5	49.1 ± 11.6	45.0 ± 12.9	−9.2 ± 10.3	−5.2% (−15.2, 4.8); 0.30*
Total (II+III) ON	23.6 ± 7.5	22.6 ± 9.1	−0.3 ± 41.2	22.3 ± 8.9	23.7 ± 10.5	6.0 ± 19.0	−5.2% (−27.0, 16.6); 0.63*
**Timed tapping, *n***							
OFF state	42.7 ± 14.1	54.4 ± 16.4	11.7 ± 7.1	41.8 ± 9.5	51.4 ± 15.7	9.6 ± 11.0	2.1 (−4.4, 8.6); 0.51*
ON state	60.5 ± 16.1	69.8 ± 16.7	9.3 ± 7.6	58.6 ± 14.8	66.4 ± 16.9	7.8 ± 9.2	1.6 (−4.3, 7.4); 0.59*
**Timed walking, s**							
OFF state	52.6 ± 65.2	24.2 ± 34.0	−23.9 ± 47.6	18.2 ± 11.3	17.0 ± 15.8	−4.8 ± 8.5	4.0 (−11.2, 19.2); 0.59*
ON state	11.5 ± 2.7	11.2 ± 2.0	−0.3 ± 1.6	10.6 ± 2.0	10.1 ± 1.8	−0.4 ± 1.2	0.6 (−0.2, 1.5); 0.14*
**Motor fluctuation diary ratings, h**							
Total OFF time	6.1 ± 1.8	5.1 ± 2.4	−1.0 ± 1.9	4.8 ± 2.3	5.0 ± 2.5	0.4 ± 2.1	−1.0 (−2.4, 0.4); 0.17*
Good quality ON time	10.3 ± 2.1	11.4 ± 3.3	1.3 ± 1.9	12.5 ± 2.7	12.1 ± 2.6	−0.4 ± 1.9	1.2 (−0.3, 2.7); 0.13*
ON time with troublesome dyskinesias	0.5 ± 1.1	0.4 ± 1.3	−0.1 ± 1.2	0.5 ± 1.0	0.4 ± 1.1	−0.1 ± 0.5	0.0 (−0.6, 0.7); 0.92*
**Total daily dose, mg**							
l-DOPA	671 ± 333	655 ± 300	−16 ± 212	569 ± 298	614 ± 306	45 ± 113	−43 (−155, 70); 0.44**
l-DOPA equivalent	1,019 ± 377	1,026 ± 408	8 ± 234	978 ± 392	1,077 ± 410	100 ± 156	−89 (−227, 48); 0.19**
**Non-motor outcomes**							
NMSS total score	38.7 ± 22.7	23.7 ± 18.9	−15.0 ± 21.2	38.3 ± 31.1	30.4 ± 28.3	−7.9 ± 21.2	−6.9 (−19.9, 6.1); 0.29*
PDQ-39 single index	25.4 ± 12.7	26.0 ± 15.4	0.6 ± 10.5	28.5 ± 15.4	23.1 ± 13.9	−5.4 ± 8.7	5.4 (−1.2, 12.0); 0.11**
**^18^F-DOPA uptake (Kocc), 10^−^^2^ min^−^^1^**							
Anterior putamen lt.	0.8 ± 0.3	1.0 ± 0.3	0.2 ± 0.2	0.8 ± 0.2	0.7 ± 0.2	−0.0 ± 0.1	0.2 (0.1, 0.3); 0.0009**
Anterior putamen rt.	0.7 ± 0.2	0.9 ± 0.2	0.2 ± 0.2	0.6 ± 0.2	0.6 ± 0.2	0.0 ± 0.1	0.2 (0.1, 0.3); <0.0001**
Central putamen lt.	0.6 ± 0.2	0.9 ± 0.3	0.3 ± 0.2	0.5 ± 0.2	0.6 ± 0.2	0.0 ± 0.1	0.3 (0.2, 0.4); <0.0001**
Central putamen rt.	0.5 ± 0.2	0.7 ± 0.2	0.3 ± 0.2	0.4 ± 0.1	0.4 ± 0.1	−0.0 ± 0.1	0.3 (0.2, 0.4); <0.0001**
Posterior putamen lt.	0.3 ± 0.1	0.6 ± 0.2	0.3 ± 0.2	0.3 ± 0.1	0.3 ± 0.1	0.0 ± 0.1	0.3 (0.2, 0.3); <0.0001**
Posterior putamen rt.	0.3 ± 0.1	0.6 ± 0.1	0.3 ± 0.1	0.3 ± 0.1	0.3 ± 0.1	0.0 ± 0.1	0.3 (0.2, 0.4); <0.0001**
Caudate lt.	1.1 ± 0.2	1.0 ± 0.3	−0.0 ± 0.1	1.0 ± 0.2	0.9 ± 0.2	−0.0 ± 0.1	0.0 (−0.1, 0.1); 0.79**
Caudate rt.	1.0 ± 0.3	1.0 ± 0.3	0.0 ± 0.1	0.9 ± 0.2	0.9 ± 0.2	−0.0 ± 0.1	0.0 (−0.0, 0.1); 0.24**

*MMRM with baseline variable as a covariate, treatment group and visit and treatment group × visit as fixed effects, and patient within treatment group as a random effect.

**ANCOVA model with baseline variable as a covariate and treatment group as a factor.

One GDNF patient had a conus injury due to a car accident and was included in the UPDRS motor scores without items 22 and 27–30. The same patient was excluded from the UPDRS ADL and total scores. Timed tapping numbers are averages of left and right. UPDRS parts I and IV, EQ-5D, body weight, Simplified Nutritional Appetite Questionnaire, Questionnaire for Impulsive-Compulsive Disorders in Parkinson’s disease, Montreal Cognitive Assessment, Mattis Dementia Rating Scale, Stroop test, Frontal Systems Behavioral Scale, Deary-Liewald reaction time, verbal fluency assessment, Beck Depression Inventory, and University of Pennsylvania Smell Identification Test remained essentially unchanged between baseline and Week 40 in both groups and did not reveal any significant treatment differences between GDNF and placebo. For the assessment of ^18^F-DOPA uptake, single position dynamic PET scans were acquired as 26 time frames over 94.5 min (1 × 30 s, 4 × 1 min, 3 × 2 min, 3 × 3 min, and 15 × 5 min) in a GE Discovery 690 PET/CT (GE Healthcare). The tissue input function was estimated from the time course of the radioactivity concentration in regions of interest placed on the occipital cortex. Analysis occurred following established procedures as previously described by Nandhagopal *et al.* (2009). One GDNF patient was included in the Week 40 analyses of ^18^F-DOPA uptake although the final PET scan was performed 2 days in advance of the visit window specified in the statistical analysis plan.

Analysis of secondary endpoints at Week 40, including percentage change from baseline in UPDRS motor score in the ON state, UPDRS ADL and total scores in both OFF and ON state, and UPDRS parts I and IV, as well as change from baseline in Parkinson’s disease diary ratings, did not reveal any significant difference between the GDNF and placebo treated groups (Table 2). While the primary and secondary results were generally favouring GDNF numerically, the mean UPDRS ADL score in the ON stage was numerically in favour of placebo. This was due primarily to two subjects who showed large percentage increases from small baseline values (367%, 14 versus 3 points; and 200%, 3 versus 1 points). Consistent with this, the absolute mean scores in the GDNF group were identical at baseline and at Week 40 (6.3).

Supplementary efficacy endpoints including timed motor tests in both OFF and ON state, total daily levodopa and levodopa equivalent dose, the NMSS, cognitive, mood and impulsivity measures, the UPSIT and quality of life questionnaires did not reveal any significant difference between the GDNF and placebo treated groups (Table 2).

To investigate the magnitude of motor response, a *post hoc* analysis testing for absolute improvement by ≥5 points or ≥10 points in the OFF state UPDRS motor score was performed in the overall population from both study stages. The analysis showed no difference between GDNF and placebo with the ≥5-point cut-off [13 (62%) versus 13 (65%); *P* > 0.50] but a significant difference in favour of GDNF with the ≥10-point cut-off [9 (43%) versus 0; *P = *0.0008; not corrected for multiple comparisons]. Figure 2B. shows the frequency distribution of motor responses in both groups.

*Post hoc* covariate analyses adjusting for demographic and Parkinson’s disease characteristics (see *post hoc* statistical analysis plan) did not identify any specific clinical features producing change in treatment effect on the primary endpoint.

### PET outcomes

**Figure 3:**
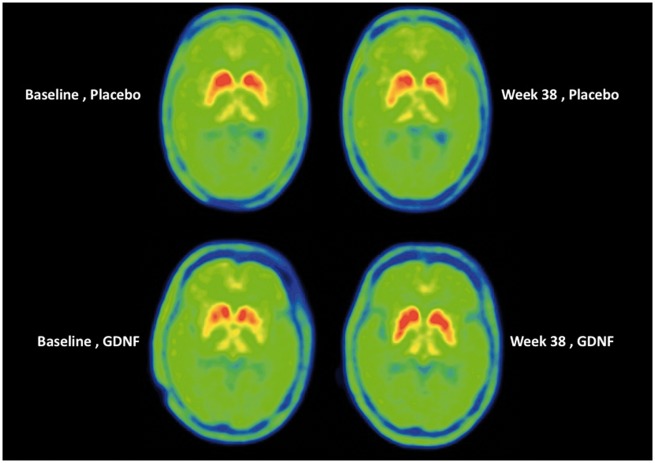
**Representative ^18^F-DOPA images from two patients, shown at baseline and end of double blind study.** *Top*: Images are from a patient who was receiving placebo infusions every 4 weeks; 10 placebo infusions in total. *Bottom*: Images are from a patient who was receiving GDNF infusions every 4 weeks; 10 GDNF infusions in total.

The PET findings at baseline were consistent with the known rostro-caudal gradient of neurodegenerative changes in the striatum of patients with Parkinson’s disease (Stoessl, 2011). Accordingly, the highest mean ^18^F-DOPA uptake rate constants (Kocc, expressed as 10^−2 ^min^−1^) at baseline were found in the caudate nucleus, and the lowest values were found in posterior putamen (Table 2 and Fig. 3).

Between baseline and Week 40, mean Kocc remained unchanged in all regions in the placebo group. In contrast, in the GDNF group, mean Kocc increased by 100% (from 0.3 ± 0.1 to 0.6 ± 0.2) in posterior putamen (left and right side, *P < *0.0001 versus placebo), 50% (left side: from 0.6 ± 0.2 to 0.9 ± 0.3) to 60% (right side: from 0.5 ± 0.2 to 0.7 ± 0.2) in central putamen (*P < *0.0001 versus placebo), and 25% (left side: from 0.8 ± 0.3 to 1.0 ± 0.3) to 29% (right side: from 0.7 ± 0.2 to 0.9 ± 0.2) in anterior putamen (*P = *0.0009 and 0.0001, respectively, versus placebo) (Table 2 and Fig. 3). No significant correlations were seen in either treatment group between percentage change from baseline to Week 40 in OFF state UPDRS motor score and alteration from baseline to Week 40 in ^18^F-DOPA Kocc in any of the putamenal regions assessed.

Data available from prior published PET studies indicate that a normal Kocc control value would be ∼1.0 in both caudate and putamen, and a value of 0.6 (seen in posterior putamen following treatment) would be similar to what would be expected following recent symptom onset (Sossi *et al.*, 2003).

### Safety

TEAEs were reported for all 41 patients (Table 3). No patient had a TEAE leading to discontinuation of study medication. TEAEs that occurred more frequently in the GDNF group than in the placebo group (difference of ≥3 patients between treatment groups) included dyskinesia, paraesthesia, Lhermitte’s sign, ON and OFF phenomena, and diplopia. The overall frequency of TEAEs of special interest was similar in both treatment groups (GDNF: 62%, placebo: 55%).

**Table 3:** Treatment-emergent adverse events experienced by at least three patients of a treatment group (overall population from both study stages)

Adverse event	GDNF (*n = *21) *n* (%)	Placebo (*n = *20) *n* (%)
Patients with at least one TEAE	21 (100)	20 (100)
Dyskinesia	9 (43)	5 (25)
Paresthesia	8 (38)	2 (10)
Lhermitte’s sign	8 (38)	0
ON and OFF phenomena	7 (33)	2 (10)
Nasopharyngitis	6 (29)	8 (40)
Headache	6 (29)	7 (35)
Application site infection	5 (24)	2 (10)
Fall	4 (19)	6 (30)
Freezing phenomenon	4 (19)	3 (15)
Muscle spasms	4 (19)	3 (15)
Constipation	4 (19)	1 (5)
Dizziness	4 (19)	1 (5)
Pain in extremity	4 (19)	1 (5)
Cough	3 (14)	4 (20)
Application site erythema	3 (14)	3 (15)
Pre-existing condition improved	3 (14)	3 (15)
Fatigue	3 (14)	2 (10)
Urinary tract infection	3 (14)	2 (10)
Lethargy	3 (14)	1 (5)
Nausea	3 (14)	1 (5)
PD-related symptoms	3 (14)	1 (5)
Diarrhea	3 (14)	0
Diplopia	3 (14)	0
Back pain	2 (10)	5 (25)
Drug effect decreased	2 (10)	4 (20)
Head injury	2 (10)	4 (20)
Joint injury	2 (10)	4 (20)
Application site pain	1 (5)	4 (20)
Insomnia	1 (5)	3 (15)
Impulsive behaviour	0	3 (15)

Serious TEAEs were reported for five (24%) GDNF patients and no placebo patients; all were unrelated to study medication: device-related events (three), pyelonephritis (one), complications from conus injury following a car accident (one). The three serious TEAEs that were considered to be device related included two occurrences of hypertrophic skin reaction around the port site that required surgical skin thinning (∼11 and ∼25 weeks into the treatment phase, respectively) and a possible port site infection that occurred ∼15 weeks into the treatment phase and required inpatient treatment with oral antibiotics.

The picture of device-related adverse events in general was dominated by port site infections and local hypertrophic scarring around the port site; many of these emerged in the post-surgical pretreatment period. Education in the study population to promote port-site device maintenance, similar to that used by patients with bone-anchored hearing aids, evolved and improved as the study progressed. No intracranial infections occurred during the study. A minor alteration to the original port design at the beginning of the primary stage resulted in port loosening in the first six patients implanted in the primary stage. This was rectified prior to treatment initiation by the introduction of a retro-fit device for firm fixation of the port in the affected patients and all subsequently enrolled patients.

Two enrolled patients did not proceed to randomization and were withdrawn prior to the start of treatment because they failed the post-surgery eligibility criteria; accordingly, they were not included in the efficacy analyses. One patient experienced a mildly symptomatic putamenal ischaemic stroke coincident with the initial test infusion. The patient recovered completely but was withdrawn to avoid unnecessary risks. The second patient suffered a small asymptomatic haemorrhage in both putamina during the initial test infusion. Subsequent observations of developing repeat gadolinium test infusions, using real-time MRI sequencing, indicated limited volume of interest coverage, which prevented the subject from being randomized. This was likely caused by haemorrhage-induced alterations restricting the retrograde flow of the infusate back along the catheter track into the desired target region. It is possible that the haemorrhage may have resulted from ejection of tissue debris collected within the catheter during the implantation. Following this occurrence, additional intraoperative catheter flushing was introduced as a routine step during device implantation, with no further issues observed in remaining subjects.

Blood sample analyses showed no measurable GDNF plasma concentrations and no GDNF-binding serum antibodies in GDNF-treated patients.

## Discussion

Reversing neurodegenerative disease remains one of the greatest medical challenges. Neurotrophic factors are among the most promising candidate therapies, with demonstrated neuroprotective and neurorestorative capabilities in animal models of Parkinson’s disease (Allen *et al.*, 2013). One major difficulty in translating these findings into the clinic has been to find a way of delivering the agents across the blood–brain barrier to meaningful volumes within relevant brain targets and potentially over the remaining lifetime of the patient. Here we show for the first time that this challenge has been met with the development of a long-term implantable drug delivery system that facilitates intermittent intraparenchymal infusions for an extended time period.

However, in this double-blind trial of 41 randomized patients with moderate stage Parkinson’s disease, 40 weeks of fixed-dose GDNF intraputamenal infusions (120 µg GDNF in 600 µl artificial CSF to each putamen), administered every 4 weeks, did not produce a significantly larger percentage improvement than placebo in OFF state UPDRS motor score, the primary study outcome. In addition, no significant improvements over placebo were observed in any of the secondary or exploratory motor endpoints, nor in any of the non-motor or quality of life endpoints. In contrast to the main clinical findings, serial PET imaging revealed a significant increase in ^18^F-DOPA uptake in the GDNF group but not the placebo group, which was spatially extensive and suggested whole putamen target tissue delivery.

The essence of our clinical findings is consistent with an earlier randomized, placebo-controlled trial of GDNF in Parkinson’s disease (Lang *et al.*, 2006), although the latter used continuous diffusion-dependent intraputamenal delivery resulting in spatially limited putamen delivery (Salvatore *et al.*, 2006). In aggregate, therefore, these two studies raise the question as to whether the underlying growth factor hypothesis is flawed, or whether the hypothesis *per se* is correct but the clinical testing in both studies has been flawed and further evaluation is required, informed by this investigation and its follow-on open-label extension.

The regenerative effects of GDNF in Parkinson’s disease have been questioned by studies using a rat model with overexpression of human wild-type α-synuclein. In this model, targeted delivery of GDNF to the striatum failed to prevent loss of nigral dopamine neurones or their terminals in the striatum (Decressac *et al.*, 2011). A subsequent investigation suggested that Nurr1, a regulator of neurotrophic factor signalling, as well as its downstream target, GDNF receptor component RET, are decreased in the presence of excess α-synuclein (Decressac *et al.*, 2012). In other words, the intracellular signalling response to GDNF may be blocked in the presence of excess α-synuclein. A recent study, however, found that *SNCA* (α-synuclein) mRNA is not increased in sporadic Parkinson’s disease, and α-synuclein accumulation does not block GDNF signalling in either Parkinson’s disease or Parkinson’s disease models (Su *et al.*, 2017). Consistent with these findings, the integrity of the GDNF signalling cascade in our patients is supported by the significant putamenal increase in ^18^F-DOPA uptake at Week 40.

Independent of the α-synuclein question, it is conceivable that mostly toxin-based, static preclinical models may not be representative of the progressive human disease. This is contradicted, however, by findings in a single patient in an early open-label phase I study who was treated with GDNF for 43 months via a unilateral continuous intraputamenal infusion and died from myocardial infarction after the study (Love *et al.*, 2005). The patient had unilateral disease on the left and was therefore treated on the right, leaving the contralateral side as an intra-patient control. At 24 months, the patient showed appreciable clinical benefit on the left coupled with improved ^18^F-DOPA uptake in the right posterior putamen. This was contrasted by a small decrease in ^18^F-DOPA uptake on the left and new-onset motor symptoms on the right in the latter part of the treatment. Post-mortem findings showed dopaminergic sprouting in the posterior third of the right putamen and greater expression of tyrosine hydroxylase (TH) on the right than on the left. Consistent with the clinical laterality, the net loss of dopaminergic neurons in the substantia nigra was greater on the right than on the left. At the same time, presumably in response to treatment with GDNF, the nigral expression of GAP43 and synaptophysin was also greater on the right.

If, therefore, the growth factor hypothesis is still valid, the question about the limitations of the clinical studies becomes pertinent. These include: Can this potential treatment only be effective in early stage disease where innervation of the striatum with dopaminergic neurons is maintained above a critical threshold? Was the dose of GDNF selected for this study sufficient? Was the treatment duration long enough? Could patient phenotype, placebo effects or methods of assessment have played a part?

Ethical considerations limited recruitment in the present study to patients with moderate stage Parkinson’s disease. The failure to demonstrate clinical benefit at 40 weeks may be the consequence of recruiting patients too late in their disease. A recent post-mortem study showed that within 4 years post-diagnosis, patients with Parkinson’s disease had an almost complete loss of TH-positive terminals and dopamine transporter immunohistochemistry in the dorsal putamen (Kordower *et al.*, 2013). However, these findings may reflect neuron hibernation and not just neuron death alone. Potentially in favour of hibernation, GDNF-treated patients in the present study, where the mean time from diagnosis was ∼8 years, showed a significant increase in putamenal ^18^F-DOPA uptake throughout the putamen with no or little differences in absolute improvement between the anterior, mid and posterior regions of interest. If the findings of the Kordower study were reflective of neurone death alone, the increase in the posterior third of the putamen, the locus of greatest reduction in TH in Parkinson’s disease, would perhaps have been expected to be smaller than in the anterior putamen. The hibernation hypothesis is also supported by the post-mortem improvements in the posterior putamen observed in the above phase I patient whose treatment commenced 5 years after diagnosis (Love *et al.*, 2005).

Ethical considerations also limited the duration of the present study to 9 months to avoid an undue length of exposure to placebo in a surgical setting. However, 9 months may have been too short a period of repeated putamenal tissue GDNF exposure to achieve adequate functionality of reconnecting neurons. It is possible that there is a lag between a biomarker effect such as ^18^F-DOPA uptake and clinical improvement. Further insight into this question may, in part, be provided by the open-label extension study.

Other potential limitations are associated with drug delivery and dose. Drug distribution in the previous, continuous low-rate infusion clinical studies was diffusion-dependent, heterogeneous, and spatially restricted to less than 10% total putamenal coverage (Salvatore *et al.*, 2006). This could explain the observed lack of clinical benefit in the earlier phase II double blind study (Lang *et al.*, 2006). The intermittent CED dosing scheme used in the present study led to much wider distribution, supported by the gadolinium-evidenced coverage of putamenal volume of interest (67.1–78.5%) and total putamen (47.8–55.0%) over 40 weeks as well as the putamen-wide increase in ^18^F-DOPA uptake in GDNF-treated patients. In contrast, as the dosing information available at the beginning of the study was sparse, a conservative approach was taken to dose, so as to avoid any unnecessary safety risk in view of the cerebellar lesions that were unexpectedly observed in several rhesus monkeys treated with very high GDNF doses (Hovland *et al.*, 2007). Due to the switch from continuous to intermittent dosing, the cumulative 4-week dose per putamen in the present study was 3.5-fold smaller than in the historic continuous dosing studies (120 µg versus 420 µg), albeit the infusate GDNF concentration was twofold higher (0.2 µg/µl versus 0.1 µg/µl).

Considering the combination of 5-fold larger putamenal coverage and 3.5-fold smaller cumulative 4-week doses, the resulting tissue GDNF concentrations in the exposed volumes were ∼18-fold lower in the present study. As retrograde transportation of GDNF from the putamen to the substantia nigra is known to be concentration-dependent (Aoi *et al.*, 2000), it is therefore possible that whilst the tissue GDNF concentrations were sufficient to induce a PET-evidenced biological effect, they were too low to produce clinical benefit within 40 weeks. This would be supported by the fact that a *post hoc* analysis of nigral ^18^F-DOPA uptake did not show any differences between treatment groups (data not shown), whereas a noticeable increase was seen in the only continuous-dosing study that assessed nigral ^18^F-DOPA uptake (Gill *et al.*, 2003). Clinically, the present study found numerical mean differences in favour of GDNF in all OFF state motor and Parkinson’s disease diary-based endpoints at all time points, and a *post hoc* responder analysis showed that significantly more patients on GDNF than on placebo had a moderate-to-large clinically important change in OFF state motor score (Shulman *et al.*, 2010). While in the absence of statistically significant results for the primary and secondary endpoints, inferences are of course speculative, one potential explanation for these findings is that underdosing played a part.

It is also worth noting that the key preclinical studies that were used to derive the clinical dose demonstrated that both the pharmacokinetics and the pharmacodynamics of intrastriatally infused GDNF were dependent on infusate GDNF concentrations (Hadaczek *et al.*, 2010; Taylor *et al.*, 2013). In particular, the Taylor study showed that while a significant increase in striatal synaptogenesis (as determined by synaptophysin concentration) was observed at an infusate GDNF concentration of 0.2 µg/µl, maximal axonal sprouting only occurred at 0.6 µg/µl (Taylor *et al.*, 2013). However, the latter concentration is very close to the concentration used in the rhesus monkeys that developed cerebellar lesions (0.67 µg/µl), and was therefore considered too high at the time (Hovland *et al.*, 2007). Meanwhile, a further 9-month toxicity study testing the intermittent dosing paradigm in rhesus monkeys has established 0.67 µg/µl as the no-observed-adverse-effect level (Luz *et al.*, 2018), thus opening the door to include this threefold higher dose level in future clinical studies.

A potential confounder of the present study was the magnitude of the placebo effect. While similar to that seen in other surgical studies in Parkinson’s disease (Olanow, 2005; Goetz *et al.*, 2008; Marks *et al.*, 2010), it was notably larger than in the earlier phase II double blind study that had been used as the main point of reference when estimating the sample size (Lang *et al.*, 2006). Conceivably, the 4-weekly visit schedule, in concert with dedicated clinical care at a single site committed to maximize patient retention, the prospect of receiving active drug at the end of the study, and investigator bias may have contributed to the observed placebo response. However, the earlier phase II double blind study also included an optional open-label extension, and other structural differences between the studies are relatively subtle considering the magnitude and consistency of the placebo response across different clinical endpoints. Therefore, it is worth considering that putamenal tissue disruption as a result of catheter implantation and repeated high-pressure CED infusions may have played a part. It is known that striatal injury, primarily via a GDNF- and BDNF-dependent mechanism mediated by activated macrophages and microglia, leads to strong and potentially persistent stimulation of the nigrostriatal dopamine system (Batchelor *et al.*, 1999, 2000; Liberatore *et al.*, 1999). Associated with the amount of trauma that was appreciably larger in the present study than in the earlier phase II double blind study (two catheters per putamen versus one, longitudinal versus vertical putamenal trajectories, and continuous low-rate, low-pressure infusions versus intermittent high-rate, high-pressure infusions), these self-repair effects may have been more pronounced in the present study. Although they would presumably have been associated with an increase in ^18^F-DOPA uptake that was not noted in the placebo group, nevertheless it seems possible that an intermittent placebo CED arm produces effects beyond those of a traditional surgical placebo arm.

Two further limitations of the study include the residual effect of symptomatic medication and the sample size. Although we used the commonly agreed definition of the practically defined OFF state (all OFF assessments were performed at a similar time in the morning, following withholding of long-acting anti-parkinson medications the day before and all other anti-parkinson medications from 6 pm the evening before), we recognize that the true ‘wash out’ period for all dopaminergic drugs including long acting agonists exceeds the standard withdrawal period allowed by this paradigm. The sample size was small despite the study being adequately powered to detect significant change in the primary outcome, and we recognize this as a further limitation to interpreting the study results.

The nine 10-point responders in the GDNF group are a potential focus of interest; however, as this a *post hoc* finding we would not wish to over-interpret its meaning. As shown in Fig. 2B, absolute changes in UPDRS motor score demonstrated a fairly even spread across subjects in the active arm from minimal worsening in score to greater than 10-point improvements. In other words, a bimodal distribution of absolute responders versus absolute non-responders was not seen. Furthermore, *post hoc* covariate analyses investigating phenotypic characteristics such as age, disease duration, disease severity, tremor predominance etc. did not identify a subtype of patients predicting an enhanced benefit. The nine 10-point responders did not differ in surgical approach, drug delivery or maintenance of their delivery systems, infusate volumes of distribution on MRI scans or magnitude of ^18^F-DOPA PET response.

At this point, therefore, we are not able to identify *a priori* a particular subgroup of Parkinson’s patients that are either more or less likely to respond to GDNF therapy. That said, it may be that some patients require longer duration of GDNF exposure to experience clinical benefit than others, therefore creating the appearance of responders versus non-responders with a 40-week infusion programme.

In contrast to our clinical outcomes, serial ^18^F-DOPA PET imaging revealed a significant increase in radioligand uptake in the GDNF group. This increase was meaningful in effect size, present throughout the putamen and notably higher than in prior Parkinson’s disease trophic factor studies (Lang *et al.*, 2006; Marks *et al.*, 2008, 2010). The earlier phase II double blind study used continuous infusions via abdominal pumps with subsequent diffusion-dependent putamenal distribution and therefore, showed increased PET signal uptake predominantly around the catheter tips (Lang *et al.*, 2006). With the intermittent CED approach, however, significant change was seen throughout the putamen, with a percentage gradient of increased improvement from posterior to anterior putamen, but not affecting the non-infused caudate, in keeping with a true biological effect of the treatment.

The increase in ^18^F-DOPA signal could indicate terminal sprouting, reawakening of hibernating terminals, an upregulation of aromatic amino acid decarboxylase, or a combination of all three (Moore *et al.*, 2003). It is important to appreciate that ^18^F-DOPA is trapped by all monoaminergic neuron types, and hence, the contribution from nigral dopaminergic versus raphe serotonergic terminal change cannot be differentiated (Moore *et al.*, 2003). Moreover, it cannot be confirmed that such change results in restoration of dopamine neurone function. For this, any potential future investigation would need to include additional imaging outcomes, such as using dopamine neuron-specific radiotracers and raclopride displacement assessments (Piccini *et al.*, 1999; Kaasinen and Vahlberg, 2017).

Comparing with prior investigations, it is worth noting that the biological effect was sufficient to move absolute ^18^F-DOPA PET Kocc uptake values from those typically associated with moderate to advanced disease to those seen in mild Parkinson’s disease (Sossi *et al.*, 2003). This contrasts with the prior clinically negative viral vector neurturin studies in Parkinson’s disease, where no PET signal benefit was observed (Marks *et al.*, 2008, 2010). Despite the marked relative percentage increase, the absolute caudal putamen uptake values at Week 40 remain approximately half that of normal control and perhaps to achieve clinical benefit the absolute level of PET signal improvement needs to be yet higher (Sossi *et al.*, 2003).

Previous foetal graft studies, which also showed a mismatch between ^18^F-DOPA uptake improvement and negative clinical outcomes, are not necessarily a mirror of our findings, as in these studies it was the grafted tissue that accounted for the enhanced radiotracer trapping rather than biological alteration within the endogenous terminal plexus *per se* (Olanow *et al.*, 2003). Indeed, a recent post-mortem examination performed 16 years after foetal grafting showed TH innervation indistinguishable from normal and yet clinical benefit was not observed (Kordower *et al.*, 2017). We fully acknowledge that the history of disease-modifying studies in Parkinson’s disease contains similar examples of achieving significant ^18^F-DOPA imaging improvements while failing to achieve clinical outcomes (Cochen *et al.*, 2003; Whone *et al.*, 2003; Mittermeyer *et al.*, 2012). This study, we believe, is yet further evidence that for disease-modifying studies the field to-date does not have an adequate clinical outcome correlating biomarker.

Patient safety was reviewed with regard to both drug and drug delivery device. As in previous studies using continuous infusion schemes (Gill *et al.*, 2003; Slevin *et al.*, 2005; Lang *et al.*, 2006), GDNF appeared to be well tolerated and safe. There were no serious adverse events related to the study drug. GDNF-related adverse events included dyskinesia and ON/OFF phenomena, but without the problematic diphasic dyskinesias reported in a previous foetal graft trial (Olanow *et al.*, 2003). It cannot be excluded that Lhermitte’s phenomena and paraesthesia that occurred at higher rates in GDNF-treated patients may have led to partial unblinding. In contrast to the earlier phase II double blind study (Lang *et al.*, 2006), no GDNF-binding serum antibodies in GDNF-treated patients were found in the present study.

Regarding the device, it is important to note that it was an in-house system developed for this trial. The 140 micro-catheters implanted into the primary stage population were delivered into the putamen safely with an operational accuracy of 0.6 ± 0.5 mm (range: 0.0–2.0 mm). In total, 417 infusion cycles amounting to 1668 individual catheter infusions were delivered in the study.

The majority of device-related adverse events were port site-associated, most commonly local hypertrophic scarring or infections, amenable to antibiotics. The frequency of these declined during the trial as surgical and device handling experience improved. No confirmed intracranial infections occurred. Test infusions to demonstrate adequate infusate delivery, prior to randomization, produced a minor stroke in one patient that subsequently fully resolved, and an asymptomatic haemorrhage in another leading to a persistent change in the implantation technique. Neither subject was subsequently randomized.

Treatment infusions post randomization were consistently asymptomatic during administration. Catheter systems remained patent during the 9-month treatment period except for one blocked infusion channel in each treatment group. This was thought to be due to filter occlusion and was compensated for by doubling the infusion volume through the paired catheter on the same side.

In conclusion, we have conducted the first randomized trial in Parkinson’s disease to use CED to administer a trophic factor to the putamen on an every-4-weeks basis via a skull-mounted port. Recruiting patients from across the UK and delivering study treatment on an outpatient basis, this trial shows that, independent from conclusions on GDNF, attending for monthly putamenal infusions of a putative neurorestorative therapy is feasible and tolerable. This study, therefore, marks a potential paradigm shift in direct-target delivery of future novel therapies as they become available, for a host of neurological conditions. As evidenced by increased ^18^F-DOPA PET signal, we have shown that this method of administration affords a spatial delivery of GDNF sufficient to achieve a biological effect across the entire putamen. At the 40-week point, however, we have not shown clinical benefit despite this putamen-wide tissue engagement. Future GDNF investigations will need to address potential reasons why our clinical primary endpoint was not reached despite apparently optimizing putamenal therapeutic delivery. The open-label extension study, to be reported separately, providing a further 40 weeks of therapy, may offer evidence on the impact of longer time-duration tissue exposure on clinical outcomes.

## Supplementary Material

Supplementary Data

## Abbreviations

ADL: activities of daily living
CED: convection-enhanced delivery
Kocc: uptake rate constant
TEAE: treatment-emergent adverse event
UPDRS: Unified Parkinson’s Disease Rating Scale
