# Supplementary material for: Randomized trial of intermittent intraputamenal glial cell line-derived neurotrophic factor in Parkinson’s disease
Source: Brain. 2019 Feb 26;142(3):512–25. doi: 10.1093/brain/awz023 (PMC6391602; doi:10.1093/brain/awz023)
Supplement: Supplementary Data [file awz023_supp.zip › awz023-suppl_data/awz023_Supplementary_Material.pdf]

<u>Supplementary material</u>

These appendices have been provided by the authors to give readers additional information about this work.

Part A – Study Protocol: first version, final version and summary of protocol amendments (uploaded separately)

Part B – CONSORT Flow Diagram (see below)

Part C – CED Device, Surgical and Infusion Procedure Background Summary (see below)

Part D – Infusion Photographs (see below)

Part E – PET Methodology (see below)

Part F – Statistical Analysis Plan: first version, final version and summary of SAP amendments (uploaded separately)

Part G – Post-Hoc Statistical Analysis Plan (uploaded separately)

Part B – Figure S1: CONSORT Flow Diagram

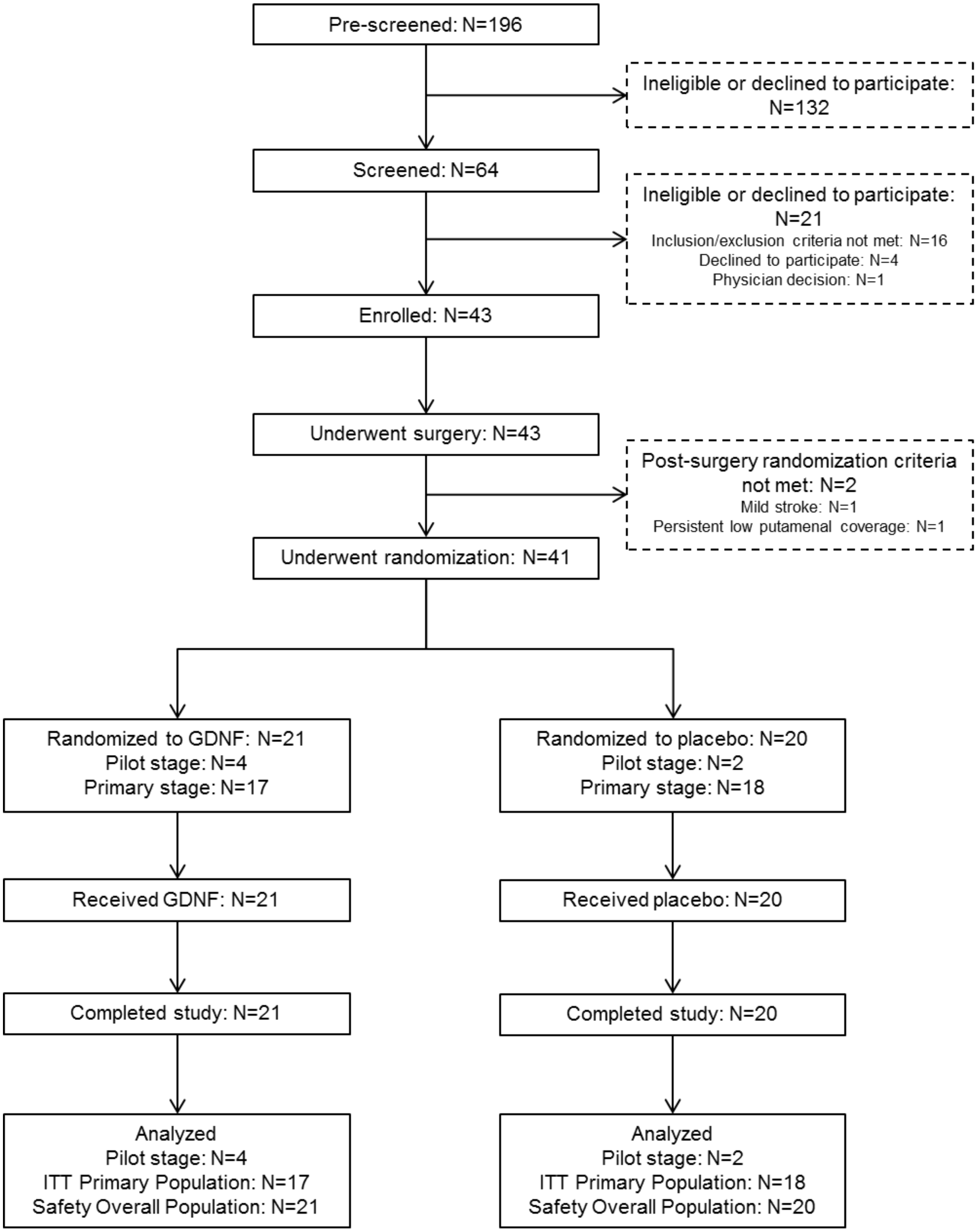

Part C – CED Device, Surgical and Infusion Procedure Background Summary

Although this multi-disciplinary collaboration in itself is not unique, the translational strategy within the R&D and preclinical stages of CED device design and performance evaluation from the laboratory to clinical trials is novel and will be reported as a detailed case study elsewhere. The collaboration was formed to ensure that failed, fragmented approaches of previous similar clinical trials were not repeated (Sampson *et al*., 2010). In our case the device design team researched and adapted systems engineering methods usually applied to the design of large complex systems such as the Bowing Dreamliner or the AirBus A380, to make certain that all aspects of the surgical procedure and reaccess infusion were integrated and operated as a single prescriptive system. For example, we have found that the same CED catheter design can perform very differently and that this can be largely attributed to varying surgical techniques employed during implantation by different surgeons across different centers. This is particularly important to the design of a novel recessed-step catheter design (Gill *et al*., 2013) which possesses features to inhibit and control infusion backflow (reflux). Therefore highly prescriptive surgical and re-access infusion procedures, as reported elsewhere (Woolley *et al*., 2013; Woolley *et al*., 2013; Lewis *et al*., 2015), are vital if such complex systems are to be repeated with minimal variation across international surgical centers and infusion clinics as will be reported further elsewhere.

References:

Gill T, Barua NU, Woolley M, et al. In vitro and in vivo testing of a novel recessed-step catheter for reflux-free convection-enhanced drug delivery to the brain. J Neurosci Methods 2013; 219:1-9.

Lewis O, Woolley M, Johnson DE, et al. A systems based approach to chronic drug delivery - A method that works. Neuro Oncol 2015; 17(Suppl 5): v75–v76.

Sampson JH, Archer G, Pedain C, et al. Poor drug distribution as a possible explanation for the results of the PRECISE trial. J Neurosurg 2010; 113:301-309.

Woolley M, Barua NU, Bienemann AS, et al. Factors facilitating optimised intraparenchymal drug delivery - A method that works. Proceedings of BIT's 3rd Annual Symposium of Drug Delivery Systems 2013

Woolley M, Barua NU, Lewis O, et al. Factors facilitating optimised intraparenchymal drug delivery – A prescriptive systems approach. Partnership opportunities in drug delivery. Boston, USA; Oct. 2013

Part D – Infusion Photographs

Part D – Figure S2.A. Infusion Suite with Patients Receiving Trial Infusions

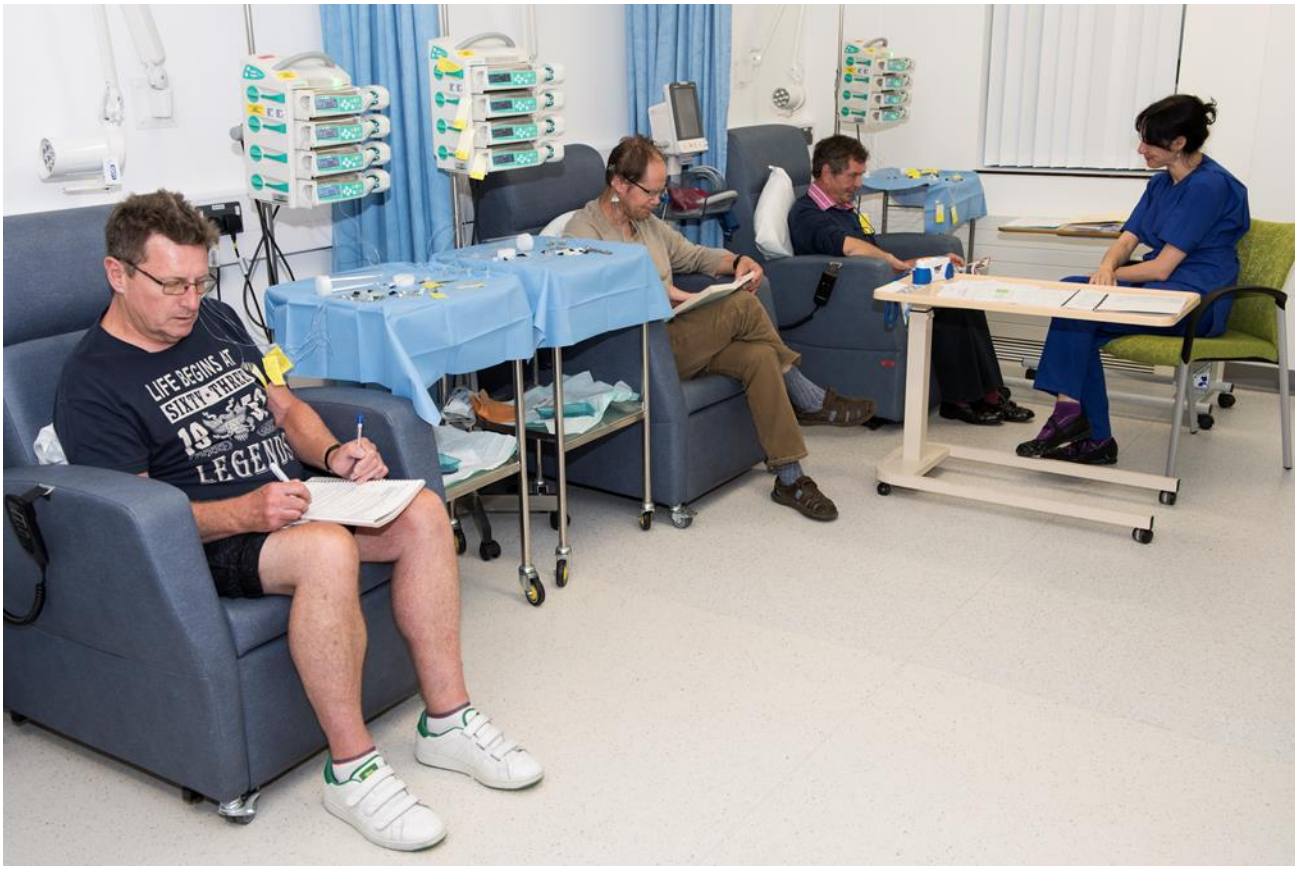

Image shows three patients receiving their monthly intraputamenal infusions from B Braun pumps via their skull-mounted ports, in a standard day-case facility, observed by an accompanying nurse.

Part D – Figure S2.B. Close-up of Patient during Infusion

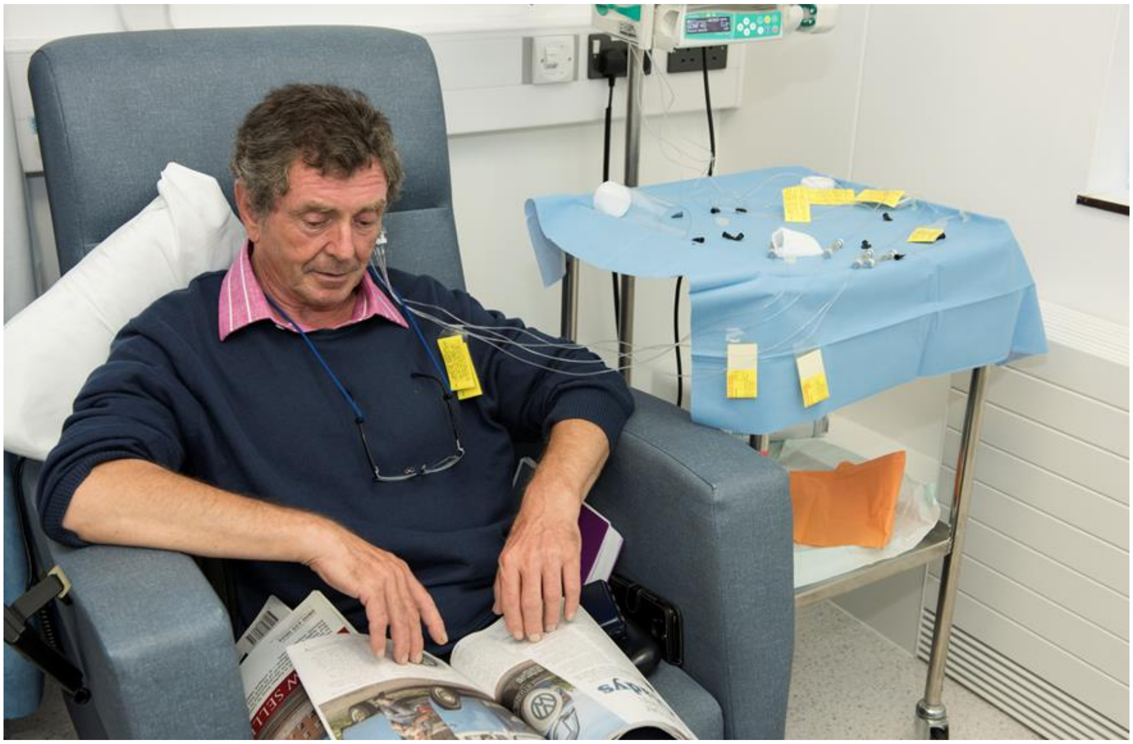

Part E – PET Methodology

[^{18}F]DOPA PET was performed at baseline prior to the first treatment infusion but post the 4-week neurosurgical healing phase and only after an intraputamenal test infusion of gadolinium-enhanced aCSF had demonstrated adequacy of infusate distribution to enable randomization. Follow-up [^{18}F]DOPA PET was undertaken at 9 months (between the last treatment infusion at Week 36 and the final clinical assessment at Week 40). Additional 3-month [^{18}F]DOPA PET scans were obtained at Week 12 in the 6 Pilot patients (data to be published elsewhere).

Patients were instructed to take no PD medications after 6:00 PM on the night before each PET acquisition and to take no long-acting dopamine agonists the day before. No high-protein foods were to be eaten on the morning of PET imaging.

Patients were transferred to the Wales Positron Emission Tomography Imaging Centre (PETIC) of Cardiff University for their PET scans. Patients were administered 150 mg of carbidopa and 400 mg of entacapone an hour before radio-tracer injection. Patients were positioned with headrest and immobilization pads/straps. A low-dose CT scan was acquired for positioning and attenuation correction. Using a GE Discovery 690 PET/CT (GE Healthcare, Chalfont St. Giles, United Kingdom), a single position Dynamic PET data-set was acquired as 26 time-frames over 94.5 min (1 × 30 sec, 4 × 1 min, 3 × 2 min, 3 × 3 min, and 15 × 5 min). Immediately after the scan commenced, 111 MBq of ^{18}F-DOPA in normal saline was administered via a long connecting tube as an intravenous bolus at the start of scanning (avoiding brain stimuli).

Anonymized DICOM images were transferred to the Department of Physics and Astronomy, University of British Columbia (UBC), Vancouver, BC, Canada where they were analyzed following established procedures as described by Nandhagopal et al (Nandhagopal et al., 2009). In brief, a time-integrated

image was created by averaging data obtained over the last 30 minutes of acquisition time. The 5 axial planes in which the striatum was best demonstrated were identified and averaged, to form a spatially and temporally integrated image for placement of regions of interest (ROIs). Circular ROIs were manually placed along the axis of the striatum, identified visually, one on the head of the caudate nucleus, and three along the rostrocaudal axis of each putamen without overlap. As a further refinement, co-registered MRI images were used as a guidance on where to place the ROIs, rather than just visual inspection. The tissue input function was estimated from the time course of the radioactivity concentration in ROIs placed on the occipital cortex. All PET analyses were performed blind to any clinical details.

Results of the PET scan analysis were entered into an electronic data capture (EDC) system by UBC. The EDC system was kept segregated from the clinical team and clinical monitors in order to avoid any potential for unblinding. Data captured included: patient initials, patient number, date of PET scan, and the results ([^{18}F]DOPA uptake rate constant [Kocc] for caudate nucleus [right/left], anterior putamen [right/left], central putamen [right/left], and posterior putamen [right/left]).

An independent, unblinded monitor reviewed the database entries against the source data to ensure completeness and that patient initials, numbers, and PET scan dates matched the expected entries. The PET EDC system was not accessed by the statistical study team until database lock and unblinding, after which, data from the EDC system was accessed by the statistical programmer for inclusion in the study analyses.

References

Nandhagopal R, Kuramoto L, Schulzer M, et al. Longitudinal progression of sporadic Parkinson's disease: a multi-tracer positron emission tomography study. Brain 2009;132:2970-9.
