# Supplementary material for: Randomized trial of intermittent intraputamenal glial cell line-derived neurotrophic factor in Parkinson’s disease
Source: Brain. 2019 Feb 26;142(3):512–25. doi: 10.1093/brain/awz023 (PMC6391602; doi:10.1093/brain/awz023)
Supplement: Supplementary Data [file awz023_supp.zip › awz023-suppl_data/awz023_Supplementary_Data_S2.pdf]

Study Title

A Placebo-Controlled, Randomised, Double-Blind Trial to Assess the Safety and Efficacy of Intermittent Bilateral Intraputamenal Glial Cell Line-Derived Neurotrophic Factor (GDNF) Infusions Administered via Convection Enhanced Delivery (CED) in Subjects with Parkinson's Disease

Internal Reference No: 2553

Ethics Ref: 12/SW/0047

EudraCT Number: 2011-003866-34

Version 1.7, 28 October 2015 (Incorporating Amendments 1-7)

Principal Investigator: Dr A Whone
Consultant Neurologist and Hon Senior Lecturer
Department of Neurology and the Burden Institute
Movement Disorder Service, Steps & Pines
Southmead Hospital
Bristol BS10 5NB, United Kingdom

Study Neurosurgeon: Professor S Gill
Consultant Neurosurgeon
Department of Neurosurgery
Southmead Hospital, North Bristol NHS Trust
Bristol BS10 5NB, United Kingdom

Sponsor: North Bristol NHS Trust (NBT)
Research & Innovation
Floor 3, Learning & Research Building
Bristol BS10 5NB, United Kingdom

Funding Sources: Parkinson's UK
215 Vauxhall Bridge Road
London SW1V 1EJ, United Kingdom

The Cure Parkinson's Trust
The Vestry
1, St Clement's Court
London EC4N 7HB, United Kingdom

Drug Manufacturer: MedGenesis Therapeutix, Inc.
730-730 View Street
Victoria, BC V8W 3Y7, Canada

Protocol authorised by:

Dr Alan Whone **28.10.2015**

TABLE OF CONTENTS

STUDY TITLE ... 1
1 SYNOPSIS ... 8
2 ABBREVIATIONS ... 11
3 BACKGROUND AND RATIONALE ... 13
4 OBJECTIVES ... 21
4.1 Primary Objective ... 21
4.2 Secondary Objectives ... 21
4.3 Other Objectives ... 21
5 TRIAL DESIGN ... 22
5.1 Summary of Trial Design ... 22
5.1.1 Pilot Stage ... 22
5.1.2 Primary Study Stage ... 23
5.1.3 Post-Study Access to Treatment ... 24
5.2 Study Endpoints ... 24
5.2.1 Primary Endpoint ... 24
5.2.2 Secondary Endpoints ... 24
5.2.3 Supplementary Efficacy Endpoints ... 25
5.2.4 Imaging Endpoints ... 26
5.2.5 Safety Endpoints ... 27
5.3 Trial Participants ... 27
5.3.1 Overall Description of Trial Participants ... 27
5.3.2 Inclusion Criteria ... 28
5.3.3 Exclusion Criteria ... 28
5.3.4 Post-Surgery Randomisation Criteria ... 30
5.4 Expenses and Benefits ... 30
5.5 Study Procedures ... 31
5.5.1 Informed Consent ... 32
5.5.2 Presurgery (up to 60 days) ... 33
5.5.3 Surgery and Healing ... 37
5.5.4 Randomisation and Preparation of Study Drug ... 40
5.6 Schedule of Treatments and Assessments ... 41
5.6.1 Baseline and First Treatment (Week 0) ... 42

5.6.2 Post-Baseline Treatments ... 44
5.6.3 Interim Assessments ... 46
5.6.4 Laboratory and Other Assessments ... 47
5.6.5 Week 40 or Early Discontinuation ... 47
5.7 Outcomes Measures ... 50
5.7.1 Efficacy Outcome Measures ... 50
5.7.2 Safety Outcome Measures ... 53
5.8 PET Scanning Procedures and Image Analysis ... 56
5.9 Definition of End of Trial ... 57
5.10 Discontinuation/ Withdrawal of Subjects from Study Treatment ... 57
5.11 Source Data ... 58
6 TREATMENT OF TRIAL PARTICIPANTS ... 59
6.1 Description of Study Medication ... 59
6.2 Description of Convection-Enhanced Delivery System ... 60
6.2.1 Implantation of the Drug Delivery System ... 60
6.2.2 Drug Infusions ... 61
6.3 Double-Blind Treatment ... 62
6.4 Compliance with Study Treatment ... 63
6.5 Accountability of the Study Treatment ... 63
6.6 Test Infusions ... 64
6.7 Concomitant Medication ... 64
7 SAFETY REPORTING ... 65
7.1 Definitions ... 65
7.2 Adverse Event (AE) ... 65
7.3 Adverse Reaction (AR) ... 65
7.4 Serious Adverse Event (SAE) ... 65
7.5 Serious Adverse Reaction (SAR) ... 66
7.6 Suspected Unexpected Serious Adverse Reaction (SUSAR) ... 66
7.7 Causality and Expectedness ... 66
7.8 Procedures for Recording Adverse Events ... 67
7.9 Reporting Procedures for Serious Adverse Events ... 68
7.10 SUSAR Reporting ... 68
7.11 Annual Safety Reports ... 68

8 STATISTICS ... 69
8.1 Description of Statistical Methods ... 69
8.2 Number of Participants ... 69
8.3 Level of Statistical Significance ... 70
8.4 Criteria for the Termination of the Trial ... 70
8.5 Procedure for Accounting for Missing, Unused, and Spurious Data ... 70
8.6 Procedures for Reporting any Deviation(s) from the Original Statistical Plan ... 70
8.7 Inclusion in Analysis ... 71
9 DIRECT ACCESS TO SOURCE DATA/DOCUMENTS ... 72
10 QUALITY CONTROL AND QUALITY ASSURANCE PROCEDURES ... 73
11 SERIOUS BREACHES ... 74
12 ETHICS ... 75
12.1 Declaration of Helsinki ... 75
12.2 ICH Guidelines for Good Clinical Practice ... 75
12.3 Approvals ... 75
12.4 Participant Confidentiality ... 75
13 DATA HANDLING AND RECORD KEEPING ... 76
14 FINANCE AND INSURANCE ... 77
14.1 Compensation for Harm ... 77
15 PUBLICATION POLICY ... 78
16 REFERENCES ... 79
17 APPENDIX A: STUDY SCHEDULE OF EVENTS ... 82
18 APPENDIX B: SAE REPORTING FLOW CHART ...
19 APPENDIX C: MONTREAL COGNITIVE ASSESSMENT (MOCA) ...
20 APPENDIX D: BECK DEPRESSION INVENTORY (BDI) ...
21 APPENDIX E: QUESTIONNAIRE FOR IMPULSIVE-COMPULSIVE DISORDERS IN PARKINSON'S DISEASE (QUIP) ...
22 APPENDIX F: UNIFIED PARKINSON'S DISEASE RATING SCALE (UPDRS) ...
23 APPENDIX G: PD FLUCTUATION DIARY ...
24 APPENDIX H: NON-MOTOR SYMPTOM ASSESSMENT SCALE FOR PD (NMSS) .
25 APPENDIX I: PARKINSON'S DISEASE QUESTIONNAIRE-39 (PDQ-39) ...
26 APPENDIX J: EUROQOL 5-DIMENSIONAL SCALE (EQ-5D) ...

List of Tables

Table 1 Schedule of Events (Presurgery; Surgery and Healing) 82
Table 2 Schedule of Events (Double-Blind Treatment) 84

List of Figures

Figure 1 Comparison of MRI Scans Between the Phase I (left) and Phase II (right) Studies 14
Figure 2 Drug Delivery to Pig Putamen (0.2 mm catheter, 5 μL/minute) 17
Figure 3 Infusing into 0.6% Agarose Gel, a Validated Model of Convection Enhanced Delivery in Brain 17
Figure 4 Clinical Scores After Termination of GDNF Therapy: Total UPDRS 19
Figure 5 Clinical Scores After Termination of GDNF Therapy: Timed Motor Tests ... 19
Figure 6 ^{18}F-DOPA Uptake in a Single Subject at Baseline and 3 Years After Cessation of GDNF 20
Figure 7 Study Schema 23

AMENDMENT HISTORY

Amendment 1 (31 May 2012)

Addition of an inclusion criterion giving contraception advice for male subjects with female partners of childbearing potential.

Amendment 2 (17 October 2012)

Modification of the up-titration portion of the intraputamenal infusion regimen (switch from a stepped up-titration scheme over 20 minutes to a linear ramping scheme over 30 to 40 minutes) and specification of the programmable syringe pump to be used for the infusions.

Amendment 3 (25 April 2013)

Switch from two-weekly infusion intervals to four-weekly infusion intervals, along with a compensatory increase in the concentration of GDNF in the infusate from 0.1 μg/μL to 0.2 μg/μL and the possibility to slow the rate of individual infusions from 5 μL/min to 3-5 μL/min in response to post-infusion MRI findings.

Amendment 4 (25 June 2013)

Introduction of a number of mostly minor changes and clarifications to optimise the design and procedures for the Primary Study Stage on the basis of the consolidated experience gathered during the Pilot Stage.

Amendment 5 (02 June 2014)

Addition of contrast-enhanced T1-weighted MRI monitoring following the test infusion at the end of the healing period in Primary Study Stage subjects and clarification of handling of subjects who are delayed in receiving surgery and/or in having their baseline (Week 0) visit. In addition, the Cattell Culture Fair Intelligence Test (CFIT) has been removed as an outcome measure.

Amendment 5.1 (23 June 2014)

Correction of the addresses of the Principal Investigator and the Study Neurosurgeon to reflect their new location following the move of Frenchay Hospital to new facilities in Southmead.

Amendment 6 (17 September 2014)

Simplification of the MRI schedule and deletion of the previously planned functional MRI substudy.

Amendment 7 (28 October 2015)

Provision of a number of clarifications and refinements that are considered mostly administrative in nature or have evolved during the preparation of the statistical analysis plan.

1 SYNOPSIS

Study Title	A Placebo-Controlled, Randomised, Double-Blind Trial to Assess the Safety and Efficacy of Intermittent Bilateral Intraputamenal Glial Cell Line-Derived Neurotrophic Factor (GDNF) Infusions Administered via Convection Enhanced Delivery (CED) in Subjects with Parkinson's Disease
Internal Ref. No.	2553
Clinical Phase	Phase II
Trial Design	A placebo-controlled, randomised, double-blind study in 2 stages: A Pilot Stage (N = 6) and a Primary Study Stage (N = 36).
Trial Participants	Subjects with bilateral idiopathic Parkinson's disease (PD) according to the United Kingdom (UK) Brain Bank Criteria
Planned Sample Size	42 subjects
Treatment and Follow-up Duration	9 months; subjects who complete the 9-month treatment period will be offered the opportunity to enrol in an open-label active treatment extension study under a separate protocol, pending approval by the Medicines and Healthcare Products Regulatory Agency (MHRA) and the local Research Ethics Committee (REC).
Planned Trial Period	The planned study period, including recruitment, is 27 months.
Primary Objective	To assess the effect of q4 weekly intermittent bilateral intraputamenal GDNF infusions on OFF-state motor function at 9 months.
Secondary Objectives	To assess the effect of intermittent bilateral intraputamenal GDNF infusions on ON-state motor function, motor complications, and ON- and OFF-state activities of daily living (ADL) at 9 months.<br>To assess the safety of intermittent bilateral intraputamenal GDNF infusions in a small pilot cohort of subjects and in the full study population.
Other Objectives	To explore the effects of intermittent bilateral intraputamenal GDNF infusions on other motor and non-motor functions, quality of life assessments, and imaging endpoints at 9 months.
Primary Endpoint	The primary endpoint of the study is the percentage change from baseline in the practically defined OFF-state Unified Parkinson's Disease Rating Scale (UPDRS) motor score (part III) in the Primary Study Stage after 9 months of double-blind treatment.
Secondary Efficacy Endpoints	• Percentage change from baseline in UPDRS motor score (part III) in the ON-state (following a levodopa challenge) after 9 months of double-blind treatment.<br>• Percentage change from baseline in UPDRS ADL score (part II) in the OFF state and in the ON state after 9 months of double-blind treatment.<br>• Percentage change from baseline in UPDRS total score (sum of motor + ADL scores) in the OFF state and in the ON state after 9 months of double-blind treatment.<br>• Percentage change from baseline in UPDRS mentation, behavior, and mood score (part I) after 9 months of double-blind treatment.<br>• Percentage change from baseline in UPDRS complications of therapy score (part IV) after 9 months of double-blind treatment.<br>• Change from baseline in PD diary ratings after 9 months of double-blind treatment; i.e., total OFF-time per day, total good quality ON-time per day (ON without dyskinesias or ON with non-troublesome dyskinesias) and ON-time per day with troublesome dyskinesias.

Supplementary Efficacy Endpoints	• Primary and secondary endpoints in the overall intention-to-treat (ITT) population including subjects randomised in the Pilot Stage.<br>• Change from baseline in supplementary motor, non-motor, medication, and quality of life endpoints after 9 months of double-blind treatment, including the following:<br>o Timed walking test (OFF and ON state).<br>o Timed tapping test (OFF and ON state).<br>o Non-Motor Symptom Assessment Scale for PD (NMSS).<br>o Parkinson's Disease Questionnaire-39 (PDQ-39).<br>o EuroQOL 5-Dimensional Scale (EQ-5D).<br>o Simplified Nutritional Appetite Questionnaire (SNAQ).<br>o Total daily dose of levodopa.
Imaging Endpoints	• Change from baseline in volume of distribution of infusate as determined by contrast-enhanced T1-weighted magnetic resonance imaging (MRI) after 9 months of double-blind treatment.<br>• Change from baseline in volume of interest (VOI) coverage and total putamenal coverage as determined by contrast-enhanced T1-weighted MRI after 9 months of double-blind treatment.<br>• Change from baseline in ^{18}F-DOPA uptake as determined by positron emission tomography (PET) scans after 9 months of double-blind treatment and after 3 months of double-blind treatment (Pilot Stage).<br>• Correlation between primary study endpoint and VOI coverage and total putamenal coverage at baseline as determined by contrast-enhanced T1-weighted MRI.<br>• Correlation between change from baseline to Week 40 in NMSS total score and total putamenal coverage at baseline as determined by contrast-enhanced T1-weighted MRI.<br>• Correlation between primary study endpoint and change from baseline to Week 40 in ^{18}F-DOPA uptake as determined by PET scan.<br>• Correlation between change from baseline to Week 40 in ^{18}F-DOPA uptake as determined by PET scan and VOI coverage and total putamenal coverage at baseline as determined by contrast-enhanced T1-weighted MRI.<br>Volume of distribution of infusate will be determined both by gadolinium contrast-enhanced T1-weighted and by T2-weighted and fluid-attenuated inversion recovery (FLAIR) 3T MRI throughout the study. The first post-infusion scan will be obtained following the test infusion of diluent at the end of the healing period (before the first randomised drug administration) in all subjects. In Pilot Stage subjects, this will be T2-weighted and FLAIR MRI only. In Primary Study Stage subjects, the test infusion will contain gadolinium contrast and be followed by a T1-weighted MRI scan in addition to the T2-weighted and FLAIR MRI scans. Pilot Stage subjects will further undergo T2-weighted post-infusion imaging with all study treatments. In Primary Study Stage subjects, no interim T2-weighted or FLAIR MRI monitoring will be done unless clinically mandated. At Week 40 (where no randomised drug administration is scheduled), Primary Study Stage subjects will receive another gadolinium contrast-containing test infusion of diluent, followed both by T1-weighted and by T2-weighted and FLAIR MRI scans. All post-infusion scans are to be completed within 2 hours following the infusion.<br>PET will be performed at baseline and 9 months in all randomised subjects. Additional 3-month scans will be obtained from subjects randomised during the Pilot Stage.

Safety Endpoints	• Frequency of device-related adverse events (AEs) during the study period<br>• Frequency of treatment-emergent AEs (all treatment-emergent AEs and treatment-emergent AEs related to study drug) during the study period.<br>• Frequency of dyskinesias, falls, adverse changes in mood, and impulsivity reported as treatment-emergent AEs during the study period (AEs of special interest).<br>• Change from baseline in the Questionnaire for Impulsive-Compulsive Disorders in Parkinson's Disease (QUIP) every 8 weeks.<br>• Change from baseline in the Montreal Cognitive Assessment (MoCA) after 9 months of double-blind treatment.<br>• Change from baseline in the Mattis Dementia Rating Scale (MDRS) after 9 months of double-blind treatment.<br>• Adverse changes in MRI findings as captured by AE reporting.<br>• Frequency of subjects with anti-GDNF antibodies during the study.<br>• Results of routine laboratory blood tests (haematology, serum chemistry) and urinalysis performed at baseline and at intervals during the trial.
Statistical Analysis	The analysis of the primary and secondary endpoints will be an ITT analysis restricted to the 36 subjects randomised during the Primary Study Stage. Subjects randomised in the Pilot Stage will, however, be included in the analysis of safety and supplementary efficacy analyses.
Investigational Medicinal Products	GDNF in artificial cerebrospinal fluid (aCSF) at a concentration of 0.2 μg/μL (active treatment) or aCSF alone (placebo).
Form	Intracerebral infusion by CED.
Dose	600 µL (containing 120 μg of GDNF or placebo) per putamen every 4 weeks for 9 months
Route	Intraputamenal infusion via a CED infusion system with 2 indwelling catheters per putamen.

2 ABBREVIATIONS

aCSF	Artificial cerebrospinal fluid
ADL	Activities of daily living
AE	Adverse event
ALT	Alanine transaminase
AR	Adverse reaction
BDI	Beck Depression Inventory
BP	Blood pressure
BRTC	Bristol Randomised Trials Collaboration
C_{CSF}	Concentration in cerebrospinal fluid
CED	Convection enhanced delivery
CFIT	Cattell Culture Fair Intelligence Test
CNS	Central nervous system
CRF	Case report form
CSF	Cerebrospinal fluid
CT	Computed tomography
CTU	Clinical Trials Unit
DeNDRON	Dementias and Neurodegenerative Diseases Research Network
DMC	Data Monitoring Committee
DOPA	Dihydroxyphenylalanine
ECG	Electrocardiogram
eGFR	Estimated glomerular filtration rate
EQ-5D	EuroQOL 5-dimensional scale
FLAIR	Fluid-attenuated inversion recovery
FrSBe	Frontal Systems Behavioural Scale
GCP	Good Clinical Practice
GDNF	Glial cell line-derived neurotrophic factor
HR	Heart rate
ICH	International Conference on Harmonisation
ITT	Intention-to-treat
MCH	Mean cellular haemoglobin
MCHC	Mean cellular haemoglobin concentration
MCV	Mean cellular volume
MDRS	Mattis Dementia Rating Scale
MedDRA	Medical Dictionary for Regulatory Activities
MHRA	Medicines and Healthcare Products Regulatory Agency
MoCA	Montreal Cognitive Assessment

MRI	Magnetic resonance imaging
NART	National Adult Reading Test
NBT	North Bristol NHS Trust
NBT R&I	North Bristol NHS Trust Research and Innovation
NMSS	Non-Motor Symptom Assessment Scale for PD
NOAEL	No observed adverse effect level
PD	Parkinson's Disease
PDQ-39	Parkinson's Disease Questionnaire-39
PET	Positron emission tomography
PI	Principal Investigator
QTc	Corrected QT (interval)
QUIP	Questionnaire for Impulsive-Compulsive Disorders in Parkinson's Disease
RBC	Red blood cell (count)
REC	Research Ethics Committee
RR	Respiration rate
RT	Deary-Liewald Reaction Time
SAE	Serious adverse event
SAP	Statistical analysis plan
SAR	Serious adverse reaction
SMPC	Summary of Medicinal Product Characteristics
SNAQ	Simplified Nutritional Appetite Questionnaire
SUSAR	Suspected unexpected serious adverse reactions
TMF	Trial Master File
UK	United Kingdom
UPDRS	Unified Parkinson's Disease Rating Scale
UPPS-P	Impulsiveness Behaviour Scale
UPSIT	University of Pennsylvania Smell Identification Test
WBC	White blood cell (count)
WHO	World Health Organization

3 BACKGROUND AND RATIONALE

Glial cell line-derived neurotrophic factor (GDNF) is a neurotrophic factor with potent effects on diverse nerve cell lines including dopaminergic, serotonergic, noradrenergic and cholinergic neurones [1, 2]. It has been shown to have both neurorestorative and neuroprotective actions in primate models of Parkinson's disease (PD) when administered intracerebrally, as protein infusion and through gene therapy by viral vectors [3-7]. If GDNF is successfully delivered to the putamen, it could have the capacity to improve a range of motor functions impaired by PD including rigidity, bradykinesia and freezing of gait. In addition, via retrograde transport from the putamen, postural instability and non-motor PD symptoms including depression, executive dysfunction and broader cognitive decline could also be improved.

There have been 4 clinical trials of GDNF in PD. In an initial trial, GDNF was administered intracerebroventricularly; however, this route did not provide sufficient penetration to target tissue for GDNF to have an effect [8]. In 2001, GDNF was infused directly into the putamen in 5 subjects with PD, with significant symptomatic improvement [9]. Success in that phase I study, together with another successful phase I study [10], led to a phase II study that showed improvement in ^{18}F-dihydroxyphenylalanine (DOPA) uptake in the caudal putamen but did not achieve the overall 25% improvement in symptomatic motor scores at 6 months that was expected [11].

Although the factors that led to the failure of that study are unknown, the most significant one likely was failure of adequate drug delivery. Direct administration of drugs to specific locations in the central nervous system (CNS) is complicated by a number of factors that affect drug distribution. Convection enhanced delivery (CED) is a method of delivering large molecule drugs to the CNS that uses a positive infusion pressure to, theoretically, homogenously distribute drugs in CNS tissue across areas larger than the drugs would spread by passive diffusion [12]. CED, however, can result in variable distribution due to pressure gradients in the target tissue. Where local anatomical features produce pressure gradients, the infused material will follow the pressure gradients away from point of delivery; e.g., sulci can create low pressure areas that pull infusate away from the target area. Another source of drug escape can be the track of the catheter used to infuse the drug. Catheters are intended to infuse drug into

the area around the catheter tip, but with larger catheters the catheter/tissue interface provides a lower resistance pathway than that of the interstitial space, and the infusate preferentially refluxes back along the catheter. Factors critical to intracerebral drug delivery, therefore, include the anatomical features of the target area, the ability to accurately locate the catheter tip, and the diameter and design of the catheter [13].

GDNF avidly binds to heparin receptors and will not passively diffuse through the brain by more than 1-2 mm [14]. In order to achieve adequate coverage of the caudal putamen it is necessary to use CED; i.e., to drive the infusate through the interstitial tissue down a pressure gradient. The successful phase I study used an experimental 0.6 mm diameter step-design catheter, but in the unsuccessful phase II study a commercial 1.04 mm non-step-design catheter was used. Figure 1 compares magnetic resonance imaging (MRI) scans taken from the phase I study (left), which shows infusate confined to the putamen, and the phase II study (right), which shows the infusate tracking back along the catheter, with almost no infusate seen in the putamen.

Figure 1 Comparison of MRI Scans Between the Phase I (left) and Phase II (right) Studies

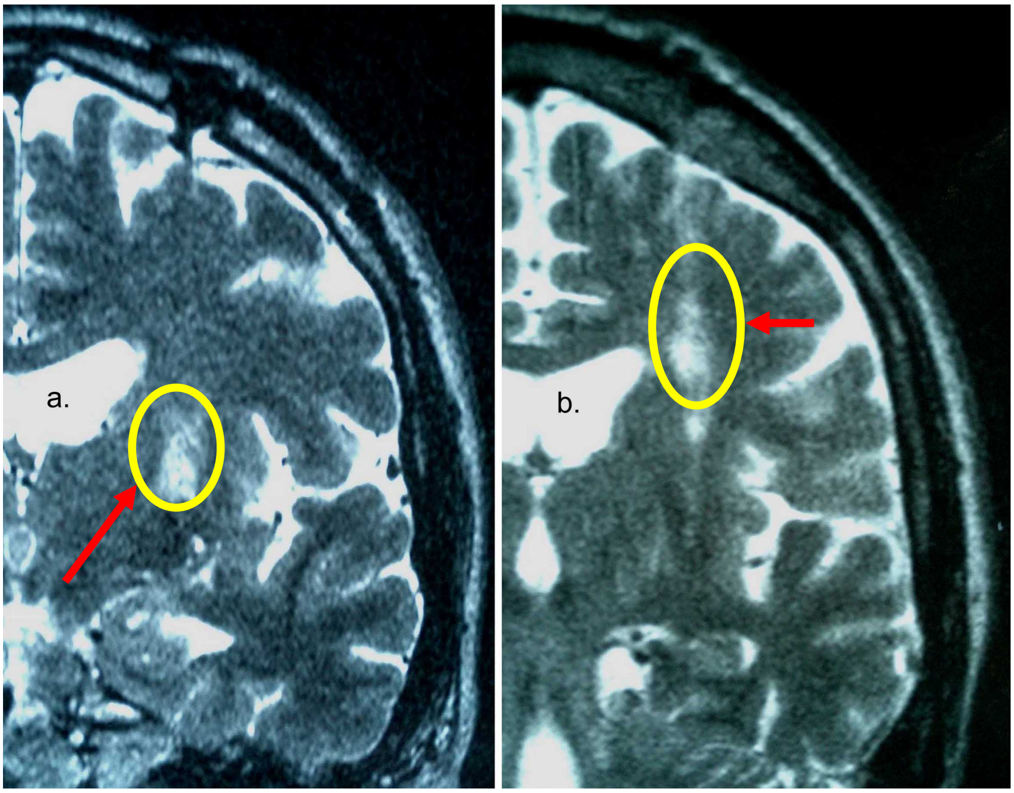

Phase I
0.6 mm catheter (Bristol)
Arrow indicates infusate filling the putamen

Phase II
1.04 mm catheter (Medtronics)
Arrow indicates infusate tracking away from the putamen along the catheter

After the phase II study, additional concerns about cerebellar toxicity in rhesus monkeys [15] and immunogenicity in human subjects [16] were raised. These concerns are addressed individually below.

Cerebellar Toxicity: A 6-month continuous dosing intraputamenal toxicology study in primates conducted by Amgen yielded a concerning result of multifocal cerebellar Purkinje cell loss affecting approximately 21% of the cerebellar cortex at the end of the recovery period in 1 rhesus monkey treated at the 3,000 μg/month level (high dose group) [15]. A subsequent review revealed similar, but less pronounced, lesions in 3 other animals treated at the same dose level. No cerebellar findings were observed at lower dose levels, or in other toxicology studies, and the 900 μg/month level was identified as the no observed adverse effect level (NOAEL) in this study. The affected animals did not show any neurological symptoms or signs, their brains were grossly unremarkable at visual evaluation at necropsy, and the lesions were not evident in standard sequential MRI investigations.

A thorough review of this study and all of the work conducted since [Luz and Mohr, manuscript under preparation] strongly suggests that the lesions were caused by abrupt withdrawal following extended exposure to concentrations in cerebrospinal fluid (CSF) of GDNF (GDNF C_{CSF}), above 1,700 pg/mL. This is consistent with the hypothesis that chronic exposure to high (exogenous) GDNF C_{CSF} leads to suppression of endogenous GDNF synthesis and receptor down-regulation on Purkinje cells. If this is followed by abrupt withdrawal of exogenous GDNF, cells can become atrophic and finally die through a caspase-dependent nonmitochondrial pathway [17].

This clinical study will use intermittent delivery, combined with thin step-design, reflux-free catheters, preventing infusate reflux into the CSF compartment and reducing this risk of chronic exposure to high GDNF C_{CSF}. Further, the dose level in this study affords a safety margin greater than 50 times the NOAEL. It is therefore unlikely that receptor down-regulation in the cerebellum will be induced in this trial.

Immunogenicity: Eighteen of 34 subjects who participated in the Amgen phase II clinical trial developed anti-GDNF antibodies, including 4 subjects who developed neutralizing antibodies [16]. While none of the subjects receiving GDNF experienced adverse effects related to the presence of neutralizing antibodies, the issue of longer term exposure makes neutralizing antibodies a potential safety issue. Although catheter

dysfunction was identified as the source of systemic exposure leading to immunization in some subjects, the most consistent source of contamination and systemic exposure to GDNF was the invasive refill process of the implanted drug reservoir. The delivery system that will be used in this study avoids this problem, as it provides for non-invasive, non-contaminant filling of the infusion catheters.

Since GDNF was withdrawn from subjects, post-mortem examination of the brain has been performed on 1 subject from the phase I study who died of an unrelated myocardial infarction within 3 months of stopping GDNF [18]. In the region of the infusion there was a significant amount of neural sprouting with increased tyrosine hydroxylase which would have led to increased dopamine production. There was also evidence of retrograde transport to the substantia nigra which also showed evidence of neural restoration. The histology also indicated that the volume of tissue in the putamen where there was upregulation of tyrosine hydroxylase covered a radius of 5 mm, but in the inner 3 mm of this area, there was no tyrosine hydroxylase upregulation and there was evidence of gliosis.

These observations indicate that even in the phase I study the infusion parameters were inadequate, and GDNF was accumulating at the catheter tip and having a toxic effect. As GDNF is known to have a long half-life in brain and may be found for 1 month or longer after infusion [19], the peri-catheter doses would have become quite high. Additionally, satisfactory tissue dosing was only achieved in less than 7% of the putamenal volume (400 of 6,000 mm^3) [20]. Despite this, however, significant clinical and ^{18}F-DOPA positron emission tomography (PET) improvements were observed.

Improved delivery offers the possibility of less local toxicity and greater clinical and radiographic improvement. Therefore, an experimental delivery system has been optimised to achieve a consistent and homogenous volume of drug distribution in a volume substantially greater than was achieved in any prior clinical study [Bienemann et al., submitted for publication]. Using an indwelling 0.2 mm catheter with a flow rate of 5 μL/min, which is 50 times greater than was used in the phase I study, the putamen of a Large White pig can be infused homogenously in approximately 1.5 hours. As can be seen in Figure 2, the infusate is entirely confined to the striatum and there is no evidence of reflux along the catheter.

Figure 2 Drug Delivery to Pig Putamen (0.2 mm catheter, 5 μL/minute)

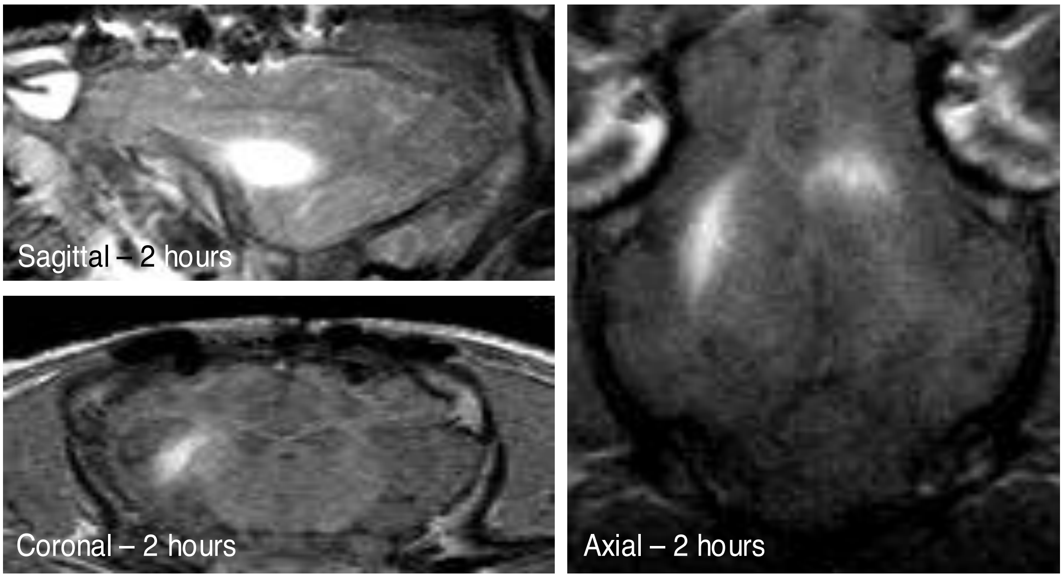

The performance of this catheter can also be appreciated from Figure 3, which shows how it compares with the Medtronic 1.04 mm catheter when infusing into 0.6% agarose gel, which is a validated model of CED in brains.

Figure 3 Infusing into 0.6% Agarose Gel, a Validated Model of Convection Enhanced Delivery in Brain

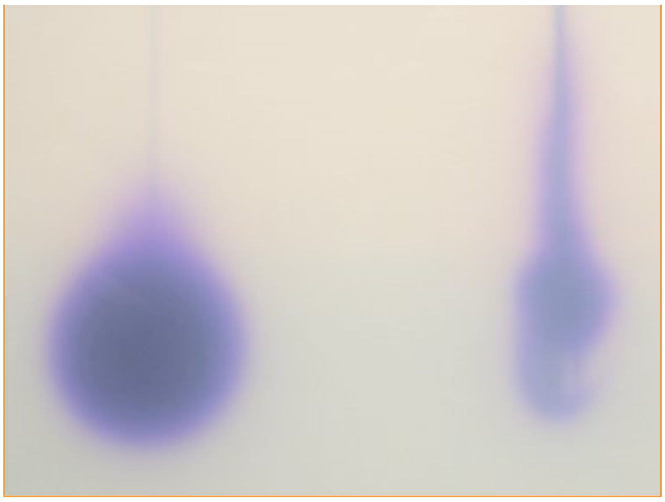

0.2 mm 5μL/minute 1.04 mm 5μL/minute

With infusion rates of this magnitude and using two 0.2 mm catheters over 50% of the human putamen could be filled with GDNF in just over an hour. Lower flow rates will not adequately cover the volume and will result in a build up of GDNF in the pericatheter region, where it might be locally toxic [20]. As GDNF remains active in brain for at least 2-4 weeks [19], repeat doses given at this interval would be optimal.

Such a dosing regimen cannot be achieved with conventional implantable pumps and of course four pumps would be required for each subject, which would be cumbersome and impractical. To overcome this problem this protocol utilises an innovative transcutaneous port solution that provides for non-invasive intermittent delivery using external programmable pumps.

Single use of a stepped catheter with a 0.2 mm outer diameter is technically feasible and works well. However, if used intermittently, this catheter design readily blocks. For intermittent delivery a larger internal diameter of the catheter is therefore required to prevent blockage. However, as the outside diameter increases the catheter is more likely to reflux, particularly if used acutely after the insertion. This is because of a low resistance pathway in traumatised tissue. If the tissue is allowed to heal, then the performance of the catheter completely changes. The optimal dimensions for a chronically implanted, non-refluxing catheter that can be used intermittently without blockage has an outside diameter of 0.6 mm with an inside diameter of 0.4 mm.

Since the termination of the previous GDNF development program at Amgen in 2004, the subjects enrolled in the first phase I study have been monitored. Clinical results from these subjects are presented in Figure 4 and Figure 5. All subjects except the 1 subject who died within 3 months of stopping GDNF were followed to 6 months post-switch off and did not show any significant deterioration in their Unified Parkinson's Disease Rating Scale (UPDRS) scores over this time course, suggesting that the observed effects are unlikely to be placebo effects. Since this time, 1 subject has refused further follow-up, and 2 subjects have undergone deep brain stimulation therapy and are therefore unavailable for further follow-up. As of 2008, 1 subject had been followed for over 3 years since ending GDNF therapy and had remained clinically stable while his levodopa equivalent medication requirement was reduced consistently by more than 50% in comparison to baseline.

Figure 4 Clinical Scores After Termination of GDNF Therapy: Total UPDRS

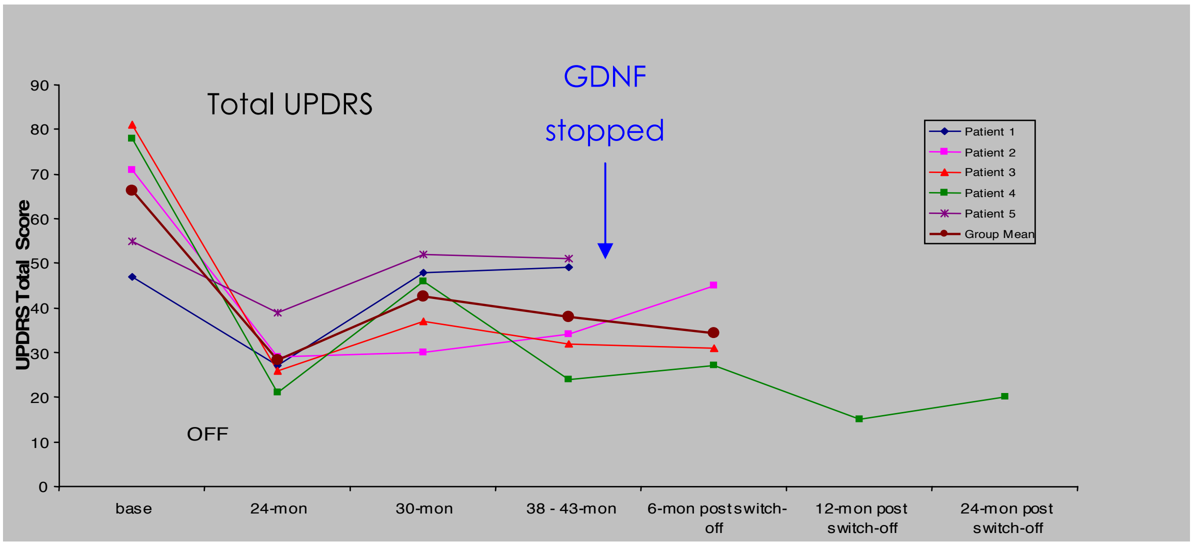

Figure 5 Clinical Scores After Termination of GDNF Therapy: Timed Motor Tests

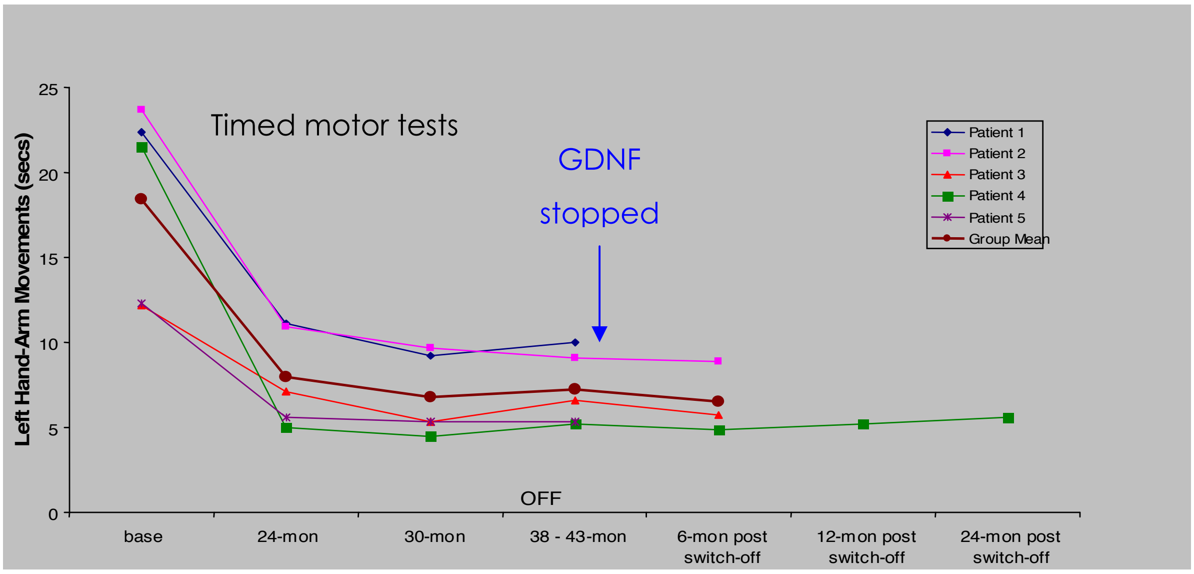

remained clinically stable.

This subject underwent further PET imaging in 2008 and showed a sustained increase in ^{18}F-DOPA uptake in the posterior putamen where the GDNF was delivered, although there was some loss of ^{18}F-DOPA uptake in the head of the caudate (Figure 6).

Figure 6 ^{18}F-DOPA Uptake in a Single Subject at Baseline and 3 Years After Cessation of GDNF

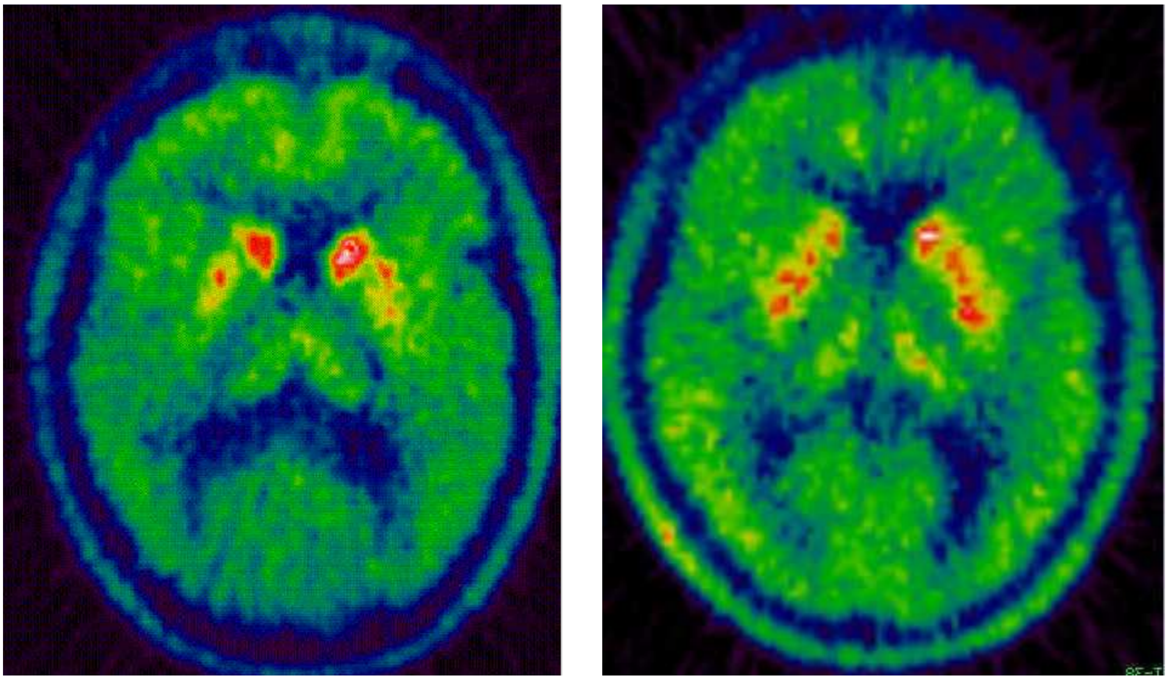

Pre-GDNF (2001) (^{18}F-DOPA PET)

3 Years after GDNF Cessation (2008) (^{18}F-DOPA PET)

Currently there is no therapy licensed for neuroprotection or neurorestoration in PD. PD relentlessly progresses and, with time, subjects accrue both treatment resistance and motor and non-motor complications. The need for effective disease modifying therapies remains paramount. GDNF has the potential to be such a clinically available therapy. The proposed intermittent CED, single-centre, placebo-controlled trial of GDNF is, therefore, long awaited and answers a pressing need to push this therapy forward for clinical usage. The single-centre design limits the number of variables in terms of delivery, which would occur if many centres were involved. Moreover, the customised in-house system for CED (see Section 6.2) will remove the problems previously encountered with a commercial system that was not suitable for CED.

4 OBJECTIVES

4.1 Primary Objective

To assess the effect of q4 weekly intermittent bilateral intraputamenal GDNF infusions on OFF-state motor function at 9 months.

4.2 Secondary Objectives

- To assess the effect of intermittent bilateral intraputamenal GDNF infusions on ON-state motor function, motor complications, and ON- and OFF-state activities of daily living (ADL) at 9 months.
- To assess the safety of intermittent bilateral intraputamenal GDNF infusions in a small pilot cohort of subjects and in the full study population.

4.3 Other Objectives

- To explore the effects of intermittent bilateral intraputamenal GDNF infusions on other motor and non-motor functions, quality of life assessments and imaging endpoints at 9 months.

5 TRIAL DESIGN

5.1 Summary of Trial Design

This is a phase II, single-centre, randomised, double-blind, placebo-controlled trial, in subjects with idiopathic PD, of intermittent bilateral posterior putamen GDNF infusions administered via CED. The study is planned to randomise a total of 42 subjects and will consist of 2 distinct stages: a Pilot Stage (N=6) and a Primary Study Stage (N=36). Figure 7 presents the study schema.

The planned study period, including recruitment, is 27 months. The primary analysis of this study will be an intention-to-treat (ITT) analysis of the 36 subjects randomised in the Primary Study Stage that will be performed when these subjects have completed (or had the opportunity to complete) 9 months of double-blind treatment.

5.1.1 Pilot Stage

The purpose of the Pilot Stage is to ensure the safety of the surgical technique and study drug administration and, if necessary, optimise these prior to initiating the Primary Study Stage. Six subjects with PD of moderate severity will be randomised in a 2:1 allocation (total of 4 subjects receiving active GDNF and 2 subjects receiving placebo) to receive infusions of GDNF or placebo every 4 weeks in a double-blind fashion for 9 months (10 infusions; last infusion at Week 36). A safety review of these 6 subjects will be performed by both the independent Data Monitoring Committee (DMC) and the Medicines and Healthcare Products Regulatory Agency (MHRA) after the last pilot subject has completed 3 months of treatment. While the Pilot Stage is primarily aimed at confirming the safety of the study treatments and procedures and meeting regulatory requirements as articulated by the MHRA, it also serves to potentially optimise options for handling infusions in subjects allowing minor modifications of procedures in response to any surgical, technical, infusion, imaging or logistical issues that may be identified. Subjects randomised in the Pilot Stage will be analysed together with the subjects randomised in the Primary Study Stage as part of the safety and supplementary efficacy analyses but will not be included in the primary efficacy analysis.

Figure 7 Study Schema

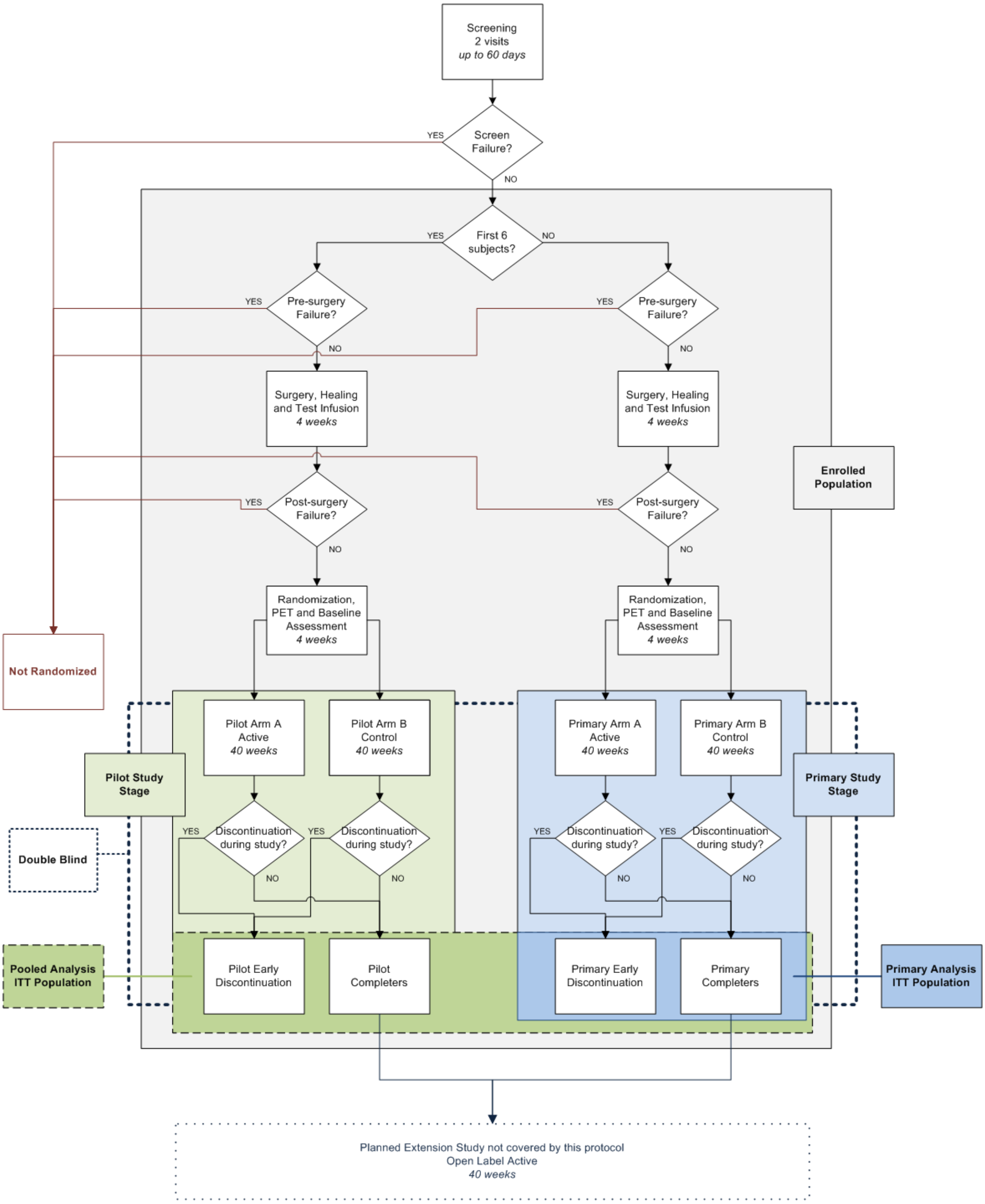

5.1.2 Primary Study Stage

Following the safety review by both the DMC and MHRA after the last pilot subject has completed 3 months of treatment, 36 additional subjects with PD of moderate severity will be randomised in a 1:1 allocation to receive infusions of either GDNF or placebo

every 4 weeks in a double-blind fashion for 9 months (10 infusions; last infusion at Week 36) in the Primary Study Stage. The primary, secondary and other applicable endpoints of the study will be determined when subjects randomised in the Primary Study Stage have completed (or had the opportunity to complete) 9 months of double-blind treatment. As the delivery technique may be slightly modified during the Pilot Stage, only subjects randomised in the Primary Study Stage will be included in the analysis of the primary and secondary endpoints.

5.1.3 Post-Study Access to Treatment

All subjects randomised in the Pilot and Primary Study Stages and completing 9 months of double-blind treatment will be offered the opportunity to enrol in an open-label active treatment extension study under a separate protocol, pending approval by the Medicines and Healthcare Products Regulatory Agency (MHRA) and the local Research Ethics Committee (REC).

The continuation of GDNF treatment beyond this study or the planned extension study can be in no way guaranteed and may well stop even if the subjects have received great benefit. This is explained in extensive detail in the Patient Information Sheet.

5.2 Study Endpoints

The analysis of the primary and secondary endpoints will be an ITT analysis restricted to the 36 subjects randomised during the Primary Study Stage. Subjects randomised in the Pilot Stage will, however, be included in the analysis of safety and supplementary efficacy analyses.

5.2.1 Primary Endpoint

The primary endpoint of the study is the percentage change from baseline in the practically defined OFF-state UPDRS motor score (part III) after 9 months of double-blind treatment.

5.2.2 Secondary Endpoints

- Percentage change from baseline in UPDRS motor score (part III) in the ON-state (following a levodopa challenge) after 9 months of double-blind treatment.

- Percentage change from baseline in UPDRS ADL score (part II) in the OFF state and in the ON state after 9 months of double-blind treatment.
- Percentage change from baseline in UPDRS total score (sum of motor + ADL scores) in the OFF state and in the ON state after 9 months of double-blind treatment.
- Percentage change from baseline in UPDRS mentation, behavior, and mood score (part I) after 9 months of double-blind treatment.
- Percentage change from baseline in UPDRS complications of therapy score (part IV) after 9 months of double-blind treatment.
- Change from baseline in PD diary ratings after 9 months of double-blind treatment; i.e., total OFF-time per day, total good quality ON-time per day (ON without dyskinesias or ON with non-troublesome dyskinesias) and ON-time per day with troublesome dyskinesias.

5.2.3 Supplementary Efficacy Endpoints

- Primary and secondary endpoints in the overall ITT population including subjects randomised in the Pilot Stage.
- Change from baseline in supplementary motor, non-motor, medication, and quality of life endpoints after 9 months of double-blind treatment, including the following:
  - Timed walking test (OFF and ON state).
  - Timed tapping test (OFF and ON state).
  - Non-Motor Symptom Assessment Scale for PD (NMSS).
  - Parkinson's Disease Questionnaire-39 (PDQ-39).
  - EuroQOL 5-Dimensional Scale (EQ-5D).
  - Simplified Nutritional Appetite Questionnaire (SNAQ).
  - Total daily dose of levodopa.

5.2.4 Imaging Endpoints

- Change from baseline in volume of distribution of infusate as determined by contrast-enhanced T1-weighted MRI after 9 months of double-blind treatment.
- Change from baseline in volume of interest (VOI) coverage and total putamenal coverage as determined by contrast-enhanced T1-weighted MRI after 9 months of double-blind treatment.
- Change from baseline in ^{18}F-DOPA uptake as determined by PET scans after 9 months of double-blind treatment and after 3 months of double-blind treatment (Pilot Stage).
- Correlation between primary study endpoint and VOI coverage and total putamenal coverage at baseline as determined by contrast-enhanced T1-weighted MRI.
- Correlation between change from baseline to Week 40 in NMSS total score and total putamenal coverage at baseline as determined by contrast-enhanced T1-weighted MRI.
- Correlation between primary study endpoint and change from baseline to Week 40 in ^{18}F-DOPA uptake as determined by PET scan.
- Correlation between change from baseline to Week 40 in ^{18}F-DOPA uptake as determined by PET scan and VOI coverage and total putamenal coverage at baseline as determined by contrast-enhanced T1-weighted MRI.

Volume of distribution of infusate will be determined both by gadolinium contrast-enhanced T1-weighted and by T2-weighted and fluid-attenuated inversion recovery (FLAIR) 3T MRI throughout the study. The first post-infusion scan will be obtained following the test infusion of diluent at the end of the healing period (before the first randomised drug administration) in all subjects. In Pilot Stage subjects, this will be T2-weighted and FLAIR MRI only. In Primary Study Stage subjects, the test infusion will contain gadolinium contrast and be followed by a T1-weighted MRI scan in addition to the T2-weighted and FLAIR MRI scans. Pilot Stage subjects will further undergo post-infusion imaging with all study treatments. In Primary Study Stage subjects, no interim T2-weighted or FLAIR MRI monitoring will be done unless clinically mandated. At

Week 40 (where no randomised drug administration is scheduled), Primary Study Stage subjects will receive another gadolinium contrast-containing test infusion of diluent, followed both by T1-weighted and by T2-weighted and FLAIR MRI scans. All post-infusion scans are to be completed within 2 hours following the infusion.

PET will be performed at baseline and 9 months in all randomised subjects. Additional 3-month scans will be obtained from subjects randomised during the Pilot Stage. For details of the PET scanning procedures and image analysis, refer to Section 5.8.

5.2.5 Safety Endpoints

- Frequency of device-related adverse events (AEs) during the study period
- Frequency of treatment-emergent AEs (all treatment-emergent AEs and treatment-emergent AEs related to study drug) during the study period.
- Frequency of dyskinesias, falls, adverse changes in mood, and impulsivity reported as treatment-emergent AEs during the study period (AEs of special interest).
- Change from baseline in the Questionnaire for Impulsive-Compulsive Disorders in Parkinson's Disease (QUIP) every 8 weeks.
- Change from baseline in the Montreal Cognitive Assessment (MoCA) after 9 months of double-blind treatment.
- Change from baseline in the Mattis Dementia Rating Scale (MDRS) after 9 months of double-blind treatment.
- Adverse changes in MRI findings as captured by AE reporting.
- Frequency of subjects with anti-GDNF antibodies during the study.
- Results of routine laboratory blood tests (haematology, serum chemistry) and urinalysis performed at baseline and at intervals during the trial.

5.3 Trial Participants

5.3.1 Overall Description of Trial Participants

Subjects with idiopathic PD with motor fluctuations.

5.3.2 Inclusion Criteria

In order to qualify for entry into the surgery and healing periods of the study, subjects MUST meet all of the following criteria:

1. Subjects diagnosed with idiopathic PD according to the United Kingdom (UK) Brain Bank Criteria. Bilateral findings must be present at study entry.
2. Duration of PD symptoms ≥ 5 years, verified by subject's medical records.
3. Age 35-75 years.
4. Presence of motor fluctuations. Subjects must have an average of at least 2.5 hours of OFF-time per day on 3-day fluctuation diaries completed during screening.
5. Ability to reliably distinguish motor states (ON without dyskinesias, ON with non-troublesome dyskinesias, ON with troublesome dyskinesias and OFF) and accurately complete fluctuation diaries.
6. UPDRS motor score (part III) in a practically defined OFF-state between 25-45.
7. Hoehn and Yahr ≤ stage III in the OFF-state.
8. Responsiveness to levodopa (≥ 40% improvement in motor UPDRS [part III] following a levodopa challenge).
9. No change in anti-parkinsonian medication for 6 weeks before screening.
10. Females of childbearing potential must have a negative pregnancy test at study entry and be willing to use an approved (by the PI or designee) form of contraception until the end of the study.
11. Males with female partners of childbearing potential must be willing to use condoms for contraception until the end of the study.
12. Provision of informed consent.

5.3.3 Exclusion Criteria

Subjects who meet any of the following criteria will NOT be eligible for entry into surgery and healing periods of the study:

1. Diagnosed with atypical parkinsonism or any known secondary parkinsonian syndrome including but not limited to medication induced, toxic, vascular, post-traumatic or post-infectious parkinsonism, progressive supranuclear palsy, multiple systems atrophy, or other neurodegenerative disorder associated with parkinsonism.
2. Signs or symptoms suggestive of atypical parkinsonian syndrome including supranuclear gaze palsy, early postural instability and falls (within 3 years of disease onset), cerebellar signs, myoclonus, disproportionate antecollis, extensor plantar responses, cortical sensory loss, emotional incontinence (pseudobulbar affect), severe bulbar dysfunction (dysarthria, dysphonia or dysphagia) or respiratory symptoms such as stridor or inspiratory sighs.
3. Family history of more than 1 first-degree relative with PD.
4. Severe dyskinesias or severe tremor which could interfere with GDNF infusion.
5. Prior neurosurgical treatment for PD, including previous treatment with GDNF or deep brain stimulation.
6. Significant neurological disorder other than PD including clinically significant head trauma, cerebrovascular disease, CSF shunt or other implanted CNS device.
7. Presence of significant depression as defined as a Beck Depression Inventory (BDI) score ≥ 20.
8. Current or past history of psychosis requiring therapy. The presence of benign hallucinosis is not exclusionary.
9. Presence or history of clinically significant impulse control disorder or presence or history of dopamine dysregulation syndrome.
10. MoCA score < 24.
11. Use within 3 months of planned catheter insertion of concomitant medications known to affect PD symptoms other than prescribed PD therapy including but not limited to neuroleptics or other central dopamine receptor blockers.
12. Any medical condition which might impair outcome measure assessments or safety measures including ability to undergo MRI scanning.

13. Screening MRI demonstrating any abnormality which would suggest an alternative cause for subject's parkinsonism.
14. Any medical condition that would put the subject at undue risk from surgical treatment or chronic implants including but not limited to bleeding disorders, chronic infections, or immunosuppressive illness.
15. History within the last 5 years of cancer with the exception of basal cell carcinoma of the skin.
16. History of drug or alcohol abuse within 2 years of planned catheter insertion.
17. Use of any investigational drug or device within 90 days of planned catheter insertion.
18. Active breastfeeding.

5.3.4 Post-Surgery Randomisation Criteria

In order to be eligible for entry into the double-blind period of the study, subjects must meet the following criteria after undergoing surgery:

1. No relevant sequelae from catheter implantation such as clinically significant intracerebral trauma, haemorrhage, or infection.
2. Total distribution volume providing at least 40% volume coverage of a predefined volume of interest (posterior two thirds of the dorsal half of the putamen) in each putamen, as confirmed by the PI on the basis of the MRI scan taken within 2 hours post-test infusion of diluent at the end of the healing period. T2-weighted, FLAIR or T1-weighted MRI scans (Primary Study Stage subjects only) can be used for this assessment.

5.4 Expenses and Benefits

Reasonable travel expenses for any visits additional to normal care will be reimbursed on production of receipts or a mileage allowance provided as appropriate.

5.5 Study Procedures

Planned study participation for each subject (both Pilot and Primary Study Stage cohorts) will comprise the following 4 periods:

- Presurgery: Up to 60 days
- Surgery and Healing: Approximately 8 weeks post-surgery (including 4 weeks recovery followed by a test infusion of diluent to confirm catheter function and drug distribution, and a further 4 week-interval before the first infusion of study drug), or longer if needed to meet the post-surgery randomisation criteria.
- Double-blind Treatment: GDNF or placebo infusions every 4 weeks for 9 months; the total dose of GDNF administered at any given infusion will be 240 μg (600 μL of 0.20 μg/μL GDNF per putamen).

Volumetric catheter performance will be determined both by gadolinium contrast-enhanced T1-weighted and by T2-weighted and FLAIR 3T MRI scans throughout the study. The first post-infusion scan will be obtained following the test infusion of artificial CSF (aCSF) (diluent) approximately 4 weeks into the healing period and 4 weeks prior to the first planned infusion of study drug in all subjects. In Pilot Stage subjects, this will be T2-weighted and FLAIR MRI only. In Primary Study Stage subjects, the test infusion will contain 2 mM gadopentetate dimeglumine and be followed by a T1-weighted MRI scan in addition to the T2-weighted and FLAIR MRI scans. Pilot Stage subjects will further undergo post-infusion imaging with all study treatments. In Primary Study Stage subjects, no interim T2-weighted or FLAIR MRI monitoring will be done unless clinically mandated. At Week 40 (where no randomised drug administration is scheduled), Primary Study Stage subjects will receive another gadolinium contrast-containing test infusion of diluent, followed both by T1-weighted and by T2-weighted and FLAIR MRI scans. All post-infusion scans are to be completed within 2 hours following the infusion.

To allow for optimisation of infusion procedures in response to potential surgical, technical, infusion, imaging or logistical issues, Pilot Stage subjects will be receiving the test infusion of aCSF and the first infusion of study medication in the 3T MRI suite with real-time MR imaging. Rapid acquisition T2-weighted and FLAIR MRI scans will be performed at approximately 20 minute intervals during these infusions.

Key clinical outcome measures will be performed at 8-week intervals throughout the study. Additional clinical outcome measures will be assessed at baseline and at 9 months.

PET will be performed at baseline (after randomisation but before the first infusion with study drug) and 9 months in all randomised subjects. Additional 3-month scans will be obtained from subjects randomised during the Pilot Stage.

In order to minimise any potential placebo effect, educational videos for staff, caregivers, and subjects will be reviewed at the beginning of the consent process. These videos are designed to encourage equipoise by emphasizing the concept of a "research partnership" rather than generating expectations of therapeutic benefit and to educate subjects on their role as a clinical trial subject. In addition, the consenting process will be scripted in an effort to standardize its administration and minimise the potential for the introduction of bias.

5.5.1 Informed Consent

The subject must personally sign and date the latest approved version of the informed consent form before any study specific procedures and assessments are performed.

Written and verbal versions of the participant information and informed consent will be presented to the potential participants detailing no less than: the experimental nature of the study; the implications and constraints of the protocol; the known side effects and any risks involved in taking part. It will be clearly stated that any participant is free to withdraw from the study at any time for any reason without prejudice to future care and with no obligation to give the reason for withdrawal.

The subject will be allowed as much time as wished to consider the information and the opportunity to question the PI or designee, their general practitioner, or other independent parties to decide whether they will participate in the study. Written informed consent will then be obtained by means of participant dated signature and dated signature of the person who presented and obtained the informed consent. The person who obtained the consent must be suitably qualified and experienced and have been authorised to do so by the PI. A copy of the signed informed consent will be given to the participant. The original signed form will be retained at the study site, and a copy will be filed in the medical notes.

For this study, subjects will provide informed consent twice. The initial consent, presented by the PI or designee, gives consent for the initial study procedures and assessments and post-surgical infusion procedures and assessments. For the surgical process itself, a separate consent will be presented by the Study Neurosurgeon or designee, to ensure that subjects are fully informed and have the opportunity to ask questions regarding risks specific to the neurosurgical procedure.

5.5.2 Presurgery (up to 60 days)

Subjects will be recruited from movement disorder clinics at the regional neurosciences centre, North Bristol NHS Trust, Bristol, and additional participants can be identified from an associated PD Nurse Specialist's cohort of subjects. If necessary, recruitment will be expanded to centres through the South West Dementias and Neurodegenerative Diseases Research Network (DeNDRON). It is anticipated that other subjects may be referred in nationally by their neurologist or self-refer although the burden of travel requirements for q4 weekly intraputamenal infusions may make such subjects unsuitable.

Presurgery will comprise 2 (or more) screening visits which may be performed in smaller units to meet any subject's needs. The assessments can be done any time in the period up to 60 days prior to surgery and in any order, with the exception of informed consent and other assessments that require a clear order (for example, PD fluctuation diary training which must be done prior to diary completion and review). The screening visits are performed by the PI or appropriately trained designee.

5.5.2.1 Screening Visit 1

The first screening visit will occur within 60 days before the planned surgical procedure. After the subject has provided informed consent, the following screening procedures and assessments will be performed in an outpatient clinic setting:

- Demographics (including date of birth, sex and race).
- Full medical history, including PD history, Hoehn and Yahr staging and prior treatments for PD
- Full physical examination, including assessments of body systems. Findings on screening physical examination will be recorded as medical history

- Weight and height
- Supine systolic and diastolic blood pressure (BP), heart rate (HR), respiration rate (RR), and temperature will be assessed after the subject has been resting quietly for at least 5 minutes. Standing systolic and diastolic BP and HR will also be assessed after the subject has been standing for 3 minutes.
- Standard 12-lead electrocardiogram (ECG). At least the following ECG parameters will be recorded: HR, PR, QT, QRS and corrected QT (QTc) intervals. The report will be signed by the PI or designee and will be recorded in the case report form (CRF) whether it is normal, abnormal but not clinically significant, or abnormal AND clinically significant (together with details of the abnormality). In the latter case the eligibility of the participants will be reviewed.
- Clinical laboratory tests, including haematology (haematocrit, haemoglobin, mean cellular haemoglobin [MCH], mean cellular haemoglobin concentration [MCHC], mean cellular volume [MCV], platelet count, red blood cell [RBC] count, white blood cell [WBC] count and WBC differential [basophils, eosinophils, lymphocytes, monocytes and neutrophils]), serum chemistry (albumin, alkaline phosphatase, alanine transaminase [ALT], creatinine, estimated glomerular filtration rate [eGFR], glucose, potassium, sodium, total bilirubin and urea) and urinalysis (colour, appearance, pH, glucose, ketones, nitrite and microscopy). A pregnancy test will be performed for all women of childbearing potential (women not of childbearing potential are those who are surgically sterile or are > 45 years and without menses for ≥ 2 years). All laboratory results will be reviewed and the reports signed by the PI or designee for determination of eligibility. Actual values will be recorded in the CRF.
- Pretreatment blood samples for anti-GDNF antibodies and plasma GDNF levels. The samples will be stored at -70°C until central analysis after all subjects have completed the study.
- Current and prior concomitant medications (from 60 days before the planned surgery) will be recorded. All over-the-counter or prescription medications,

vitamins, and/or herbal supplements will be recorded in the CRF with their indications.

- MoCA (Section 5.7.2.1) and MDRS (Section 5.7.2.2) will be performed and should be completed with the subject in the ON state.
- Subjects will be trained on the completion of fluctuation diaries (Section 5.7.1.4). Their ability to accurately assess their state (ON/OFF) will be assessed during this visit. Subjects will be required to assess their state every 30 minutes for 2-4 hours. Their ratings will be compared to those of a qualified staff member. There must be at least a 75% concordance in the ON/OFF rating for the subject to be qualified for diary completion as a prerequisite to enter the study. Should the subject be unable to reach this concordance level, they may have additional training with one further attempt to accurately complete this assessment. If they are unable to accurately complete diaries after one additional retraining session, they will not be able to proceed with the study.

After completion of the Screening Visit 1 procedures and assessments, subject PD fluctuation diaries will be distributed and the subject will be instructed to complete the diaries on 3 successive predetermined days in the week prior to the next study visit. Because of the need to withhold all PD medication for part of the day before the next assessment, this day should NOT be used for diary collection.

All AEs occurring from the time of screening must be recorded as part of the subject's medical record. AEs meeting the criteria for serious adverse events (SAEs; Section 7.4) from the time of screening must be reported as directed in Section 7.9. All AEs occurring from the time of screening (first consent) until the Week 40 visit has been completed (or 28 days after the last dose of study medication for subjects who discontinue the study early) will be recorded on the AE pages of the CRF.

5.5.2.2 Screening Visit 2

For Screening Visit 2, subjects will withhold all PD medications as follows:

- No PD medications will be taken after 6:00 PM on the night before the Screening Visit 2 assessments and

- No long-acting PD medications will be taken on the day before the Screening Visit 2 assessments.

Subjects will refrain from eating any high-protein foods on the morning of the assessments.

At the beginning of the assessment, all OFF period measures are to be completed first:

- UPDRS part II (ADL) and part III (motor) in OFF-state (Section 5.7.1.1)
- Timed walking test (Section 5.7.1.2)
- Timed tapping test (Section 5.7.1.3)

A levodopa challenge will then be performed (dose will be based on the subject's standard treatment in levodopa equivalents; refer to site-specific general guidelines) and when the subject goes ON the following post-challenge tests will be performed:

- Full UPDRS (including parts I, II, III and IV; Section 5.7.1.1)
- Timed walking test (Section 5.7.1.2)
- Timed tapping test (Section 5.7.1.3)

The following procedures may be completed irrespective of the ON/OFF state of the subject:

- Any changes to concomitant medications from the previous assessment will be recorded. All over-the-counter or prescription medications, vitamins, and/or herbal supplements will be recorded in the CRF with their indications.
- Subject PD fluctuation diaries (Section 5.7.1.4) will be collected and reviewed, and investigational centre staff will review the PD fluctuation diaries with the subjects to ensure that they are being properly completed. Any questions the subject may have will be answered.
- Simplified Nutritional Appetite Questionnaire (SNAQ; Section 5.7.1.7).
- University of Pennsylvania Smell Identification Test (UPSIT; Section 5.7.2.7).
- Full brain MRI (including T2-weighted and FLAIR 3T MRI).
- Any AEs observed or reported since Screening Visit 1 will be recorded on the AE pages of the CRF.

The following assessments will be performed during screening to serve as baseline assessment for determination of on-study changes; these are included here to reduce the patient burden at post surgery Baseline (Week 0), and they are not required to be completed on the same day as the above tests provided they are performed with the patient in the ON state:

- Stroop (Section 5.7.2.3).
- National Adult Reading Test (NART; Section 5.7.2.4.1).
- Frontal Systems Behavioural Scale (FrSBe; Section 5.7.2.4.2).
- Deary-Liewald Reaction Time (RT; Section 5.7.2.4.3).
- UPPS-P Impulsiveness Behaviour Scale (Section 5.7.2.4.4).
- Verbal fluency assessment (Section 5.7.2.4.5).
- BDI (Section 5.7.2.5).
- PD Non-Motor Symptom Scale (NMSS; Section 5.7.1.5).
- Parkinson's Disease Questionnaire-39 (PDQ-39; Section 5.7.1.6).
- EuroQOL 5-dimensional scale (EQ-5D; Section 5.7.1.6).
- QUIP (Section 5.7.2.6).

5.5.2.3 Rescreening

Subjects who do not meet entry criteria may be rescreened at a future time point at the discretion of the Principal Investigator.

In the event of a delay between the start of screening and surgery which is more than two weeks outside the visit window, rescreening is required. However, under these circumstances, the following items from the initial screening may be used without repetition: Informed consent, diary training and concordance assessment, screening MRI, UPSIT, NART, verbal fluency assessment and anti-GDNF antibody testing.

5.5.3 Surgery and Healing

Following completion of screening procedures and assessments, eligible subjects will be consented prior to undergoing any surgical procedures.

Within 60 days of the first screening visit, subjects will report to the North Bristol NHS Trust for implantation of CED catheters (2 in each putamen). All surgical procedures will be carried out according to institutional practices and under the direction of the Study Neurosurgeon. Subjects will remain hospitalised postsurgery for a period of time determined by the Study Neurosurgeon or designee and based on the individual subject's course of recovery. Catheter placement will be assessed by computed tomography (CT).

Subjects will be allowed to heal for approximately 4 weeks after surgery. Data collected during surgery and healing will include concomitant medications and AEs.

After the 4-week healing period, subjects who, in the opinion of the Study Neurosurgeon and PI, have not experienced an unacceptable surgical complication and continue to meet the entry criteria (the healing period may be extended if, in the opinion of the Study Neurosurgeon and PI, such extension is necessary to allow adequate recovery) will undergo an intraputamenal test infusion of aCSF (diluent) using the infusion algorithm for study medication (detailed in Section 6.3).

Pilot Stage subjects: The test infusion of aCSF will be performed in the 3T MRI suite with real-time MR imaging. Rapid acquisition T2-weighted and FLAIR MRI scans will be performed at approximately 20 minute intervals. Real-time MRI scans will be assessed by the PI, Study Neurosurgeon or appropriately experienced designee. Real-time imaging will facilitate immediate cessation of infusions via one or more catheters if any of the following criteria are met:

1. Intraputamenal haemorrhage or post-surgical fluid collection is identified
2. The catheter is blocked
3. There is significant reflux of infusate out of the putamen without evidence of intraputamenal distribution

Subjects will be carefully observed for safety during and after administration of the test infusion. During administration, whilst in the scanner, HR, RR and oxygen saturation will be monitored continuously, and systolic and diastolic BP will be collected every 15 minutes. Both seated and standing systolic and diastolic BP and HR will be assessed within 30 minutes after the end of the infusion. A brief neurological screen (Glasgow

Coma Scale) will also be done before infusion, 30 minutes into the infusion, and after completion of the infusion.

Final T2-weighted and FLAIR 3T MRI scans will be completed within 2 hours of the test infusion. These latter scans will be relevant to determine infusate distribution as part of the post-surgery eligibility assessment (Section 5.3.4).

Primary Study Stage subjects: The test infusion of aCSF will contain gadolinium contrast and be followed within 2 hours by T2-weighted, FLAIR and T1-weighted 3T MRI scans to assess catheter patency and infusate distribution. Subjects will be carefully observed for safety during and after administration of the test infusion. During administration, seated vital signs (systolic and diastolic BP, HR and RR) will be collected every 15 minutes, and both seated and standing systolic and diastolic BP and HR will be assessed within 30 minutes after the end of the infusion. A brief neurological screen (Glasgow Coma Scale) will also be done before infusion, 30 minutes into the infusion, and after completion of the infusion.

Subjects randomised in the Pilot Stage will remain at the facility for safety observation for at least 22 hours (i.e., including an overnight stay) after completing the test infusion. Subjects randomised in the Primary Study Stage will be observed for at least 4-6 hours prior to discharge home.

The post-test infusion MRI will be reviewed for the post-surgery eligibility assessment. Subjects who are deemed to have adequate catheter function yielding at least 40% volume coverage of a predefined volume of interest (posterior two thirds of the dorsal half of the putamen) in each putamen will be randomised and will return for their first treatment after approximately 4 weeks. Corrective action will be considered for subjects who have insufficient catheter performance yielding less than 40% volume coverage of a predefined volume of interest (posterior two thirds of the dorsal half of the putamen) in each putamen. This may include the following:

- Increasing the infusion volume for any given catheter to compensate for loss or inability to use the second catheter in the same putamen. The total infusion volume given per putamen must not exceed that given in the standard regimen. The tolerability of an alternate infusion regimen and its ability to provide sufficient putamenal coverage would need to be confirmed using another infusion of aCSF with following MRI prior to proceeding with study drug

infusions. Approximately 4 weeks will be allowed between different test infusions and between the last test infusion and the first study infusion.

- Surgical replacement of blocking or leaking catheters: In case of surgical correction, another 4-week healing period, followed by renewed assessment of catheter performance, will be allowed.

In addition, MoCA (Section 5.7.2.1) will be performed in the ON state on the day of the test infusion, to assess any cognitive effects of the surgery. This must be completed prior to infusion of diluent.

Also during this visit, subject PD fluctuation diaries will be distributed and the subject will be instructed to complete the diaries on 3 successive predetermined days in the week prior to the next study visit.

5.5.4 Randomisation and Preparation of Study Drug

Randomisation will be administered centrally via the Bristol Randomised Trials Collaboration (BRTC) Clinical Trials Unit (CTU) after the inclusion/exclusion criteria have been confirmed and the post-surgery randomisation criteria are met as confirmed by the PI. Eligible subjects in the Pilot Stage will be randomised in a 2:1 ratio to achieve 4 active and 2 placebo subjects. In the Primary Study Stage, eligible subjects will be randomised in a 1:1 ratio to the active or placebo arm, using a blocked randomisation scheme with a block size of 6. The randomisation sequence will be based on a computer programme, which will allocate a random number that determines the study drug (GDNF or aCSF) dispensed by the pharmacy whilst maintaining concealment of allocation.

All study drug will be prepared by an unblinded pharmacist at the study site based on the randomisation. Ready to use preparations of GDNF or aCSF will be provided to the PI or designee in identical containers identifiable only by codes unknown to the PI or any other study staff. Drug preparation guidelines are provided in a separate pharmacy manual.

In the event that there is a delay in randomization which results in the baseline assessment occurring more than 24 weeks after screening visit 2, efficacy outcome measures completed during screening but not repeated at baseline must be repeated

prior to the baseline visit unless they have a significant practice effect. The list of measures to be repeated includes:

- Stroop
- Reaction time
- FrSBe
- UPPS-P
- PD NMSS
- PDQ-39
- EQ-5D
- SNAQ
- UPSIT
- QUIP

5.6 Schedule of Treatments and Assessments

Upon receipt of blinded study drug as described above, the PI or designee will initiate a 40-week (approximately 9 months) course of four-weekly intraputamenal administration of GDNF or placebo via CED. The full course will comprise 11 scheduled visits (baseline and 10 q4 weekly visits). Except for the final visit at Week 40 where no randomised drug infusion will be given, at each visit GDNF/placebo will be administered, followed by clinical assessment of safety, including dyskinesias and falls, mood and impulsivity. If necessary, infusion visits may take place over more than 1 day, for example when study drug infusion is planned for early morning. At Week 40, Primary Study Stage subjects will receive another gadolinium contrast-containing test infusion of diluent, followed both by T1-weighted and by T2-weighted and FLAIR MRI scans. AEs, concomitant medications and vital signs will be recorded at all visits. PD assessments and QUIP will be performed every 8 weeks, and full assessments of safety and PD variables will be performed at baseline and the final assessment.

5.6.1 Baseline and First Treatment (Week 0)

As part of the baseline assessments, all subjects will undergo PET scanning between randomisation and the first infusion with study drug.

The subject will report to the investigative centre (PI or designee) for baseline assessments and to begin treatment.

For this assessment, subjects will withhold all PD medications as follows:

- No PD medications will be taken after 6:00 PM on the night before the Baseline assessments and
- No long-acting PD medications will be taken on the day before the Baseline assessments.

Subjects will refrain from eating any high-protein foods on the morning of the assessments.

At the beginning of the assessment, all OFF period measures are to be completed first:

- UPDRS part II (ADL) and part III (motor) in OFF-state (Section 5.7.1.1)
- Timed walking test (Section 5.7.1.2)
- Timed tapping test (Section 5.7.1.3)

A levodopa challenge will then be performed (refer to study-specific Standard Operating Procedures) and when the subject goes ON the following post-challenge tests will be performed:

- Full UPDRS (including parts I, II, III and IV; Section 5.7.1.1)
- Timed walking test (Section 5.7.1.2)
- Timed tapping test (Section 5.7.1.3)
- QUIP (Section 5.7.2.6)

Additional pretreatment assessments which can be completed irrespective of ON/OFF status will include the following:

- Weight and height.

- Vital signs: Seated systolic and diastolic BP, HR, RR and temperature will be assessed after the subject has been sitting quietly for at least 5 minutes. Standing systolic and diastolic BP and HR will also be assessed after the subject has been standing for 3 minutes.
- Collection of subject PD fluctuation diaries (Section 5.7.1.4)
- Any changes to concomitant medications from the previous visit will be recorded. All over-the-counter or prescription medications, vitamins, and/or herbal supplements will be recorded in the CRF with their indications.
- Any AEs observed or reported since the previous visit will be recorded.

After completion of the pretreatment procedures and assessments, the unblinded pharmacist will provide blinded study drug to the PI or designee, who will administer the drug over a period of approximately 90 to 150 minutes using pre-programmed micro-infusion pumps (detailed in Sections 6.2 and 6.3).

Pilot Stage subjects: The infusion of blinded study drug will be performed in the 3T MRI suite with real-time MR imaging. Rapid acquisition T2-weighted and FLAIR MRI scans will be performed at approximately 20 minute intervals. Real-time MRI scans will be assessed by the PI, Study Neurosurgeon or appropriately experienced designee. Real-time imaging will facilitate immediate cessation of infusions via one or more catheters if any of the following criteria are met:

1. Intraputamenal haemorrhage or post-surgical fluid collection is identified
2. The catheter is blocked
3. There is significant reflux of infusate out of the putamen without evidence of intraputamenal distribution.

Final T2-weighted and FLAIR 3T MRI scans will be completed within 2 hours of the infusion.

Subjects will be carefully observed for safety during and after study drug administration. During administration, whilst in the scanner, HR, RR and oxygen saturation will be monitored continuously, and systolic and diastolic BP will be collected every 15 minutes. Both seated and standing systolic and diastolic BP and HR will be assessed within 30 minutes after the end of the infusion. A brief neurological

screen (Glasgow Coma Scale) will also be done before infusion, 30 minutes into the infusion, and after completion of the infusion.

Subjects will remain at the facility for safety observation for at least 22 hours (i.e., including an overnight stay) after completing study drug administration.

Primary Study Stage subjects: Subjects will be carefully observed for safety during and after study drug administration. During administration, seated vital signs (systolic and diastolic BP, HR and RR) will be collected every 15 minutes, and both seated and standing systolic and diastolic BP and HR will be assessed within 30 minutes after the end of the infusion. A brief neurological screen (Glasgow Coma Scale) will also be done before infusion, 30 minutes into the infusion, and after completion of the infusion.

Subjects will remain at the facility for safety observation for at least 4 to 6 hours after completing study drug administration.

5.6.2 Post-Baseline Treatments

After the Baseline assessment and Week 0 treatment, subjects will return to the clinic for repeat treatments every 4 weeks at Weeks 4, 8, 12, 16, 20, 24, 28, 32 and 36. Week 4 must be ± 1 day of scheduled date; all other treatments may be ± 3 days. If necessary, in the Primary Study Stage, a treatment may be given a maximum of + 7 days from the scheduled date; if the treatment cannot be given within + 7 days, it will be considered missed and the treatments will resume with the following scheduled date. Before each PD outcome measure assessment (Weeks 8, 16, 24 and 32), subjects will withhold all PD medications and begin the assessments in the OFF state (as detailed in Section 5.6.3). For all other treatments, subjects may take their PD medications as usual and report to the hospital irrespective of whether they are ON or OFF.

At every treatment, the following procedures and assessments will be performed before treatment:

- Vital signs: Seated systolic and diastolic BP, HR, RR and temperature will be assessed after the subject has been sitting quietly for at least 5 minutes. Standing systolic and diastolic BP and HR will also be assessed after the subject has been standing for 3 minutes.

- Any changes to concomitant medications from the previous visit will be recorded. All over-the-counter or prescription medications, vitamins, and/or herbal supplements will be recorded in the CRF with their indications.
- Any AEs observed or reported since the previous visit will be recorded.

After completion of the pretreatment procedures and assessments, the unblinded pharmacist will provide blinded study drug to the PI or designee, who will administer the drug over a period of approximately 90 to 150 minutes using pre-programmed micro-infusion pumps.

Subjects will be carefully observed for safety during and after study drug administration. During administration, seated vital signs (systolic and diastolic BP, HR and RR) will be collected every 30 minutes, and both seated and standing systolic and diastolic BP and HR will be assessed within 30 minutes after the end of the infusion. A brief neurological screen (Glasgow Coma Scale) will also be done before infusion, 30 minutes into the infusion, and after completion of the infusion.

Pilot Stage subjects only: Subjects will remain at the facility for safety observation for at least 4 to 6 hours after completing study drug administration at Week 4, for at least 2 to 4 hours after completing administration at Week 8, and for at least 1 to 2 hours after completing study drug administration at all subsequent treatments.

Primary Study Stage subjects: Subjects will remain at the facility for safety observation for at least for at least 2 to 4 hours after completing study drug administration at Week 4, and for at least 1 to 2 hours after completing study drug administration at all subsequent treatments.

The last treatment will be administered at Week 36.

At the treatment before each Interim Assessment to assess PD outcome measures and at the last treatment (i.e., at Weeks 4, 12, 20, 28 and 36), subjects will be issued PD fluctuation diaries (Section 5.7.1.4) to complete at home before the next assessment. The subject will be instructed to complete diaries for 3 consecutive predetermined days in the week prior to the next study treatment. The day immediately prior to the study treatment is NOT to be used for diary recording.

5.6.3 Interim Assessments

Subjects will undergo extended safety and efficacy assessments every 8 weeks at Weeks 8, 16, 24 and 32.

For these assessments, subjects will withhold all PD medications as follows for assessment in the OFF state:

- No PD medications will be taken after 6:00 PM on the night before the assessments and
- No long-acting PD medications will be taken on the day before the assessments.

Subjects will refrain from eating any high-protein foods on the morning of the assessments.

Interim Assessment visits will also be used to administer treatments, and procedures and assessments will include those listed in Section 5.6.2 for Treatments, but the following assessments will also be performed pretreatment:

At the beginning of the assessment, all OFF period measures are to be completed first:

- UPDRS part II (ADL) and part III (motor) in OFF state (Section 5.7.1.1)
- Timed walking test (Section 5.7.1.2)
- Timed tapping test (Section 5.7.1.3)

A levodopa challenge will then be performed (refer to study-specific Standard Operating Procedures) and when the subject goes ON the following post-challenge tests will be performed:

- Full UPDRS (including parts I, II, III and IV; Section 5.7.1.1)
- Timed walking test (Section 5.7.1.2)
- Timed tapping test (Section 5.7.1.3)
- QUIP (Section 5.7.2.6)

Additional study procedures which can be performed irrespective of ON/OFF state include:

- Collection of subject PD fluctuation diaries (Section 5.7.1.4)

- Weight and height

5.6.4 Laboratory and Other Assessments

The following laboratory procedures will be performed at Weeks 4, 16, and 28:

- Clinical laboratory tests, including haematology (haematocrit, haemoglobin, MCH, MCHC, MCV, platelet count, RBC, WBC count and WBC differential [basophils, eosinophils, lymphocytes, monocytes and neutrophils]), serum chemistry (albumin, alkaline phosphatase, ALT, creatinine, eGFR, glucose, potassium, sodium, total bilirubin and urea) and urinalysis (colour, appearance, pH, glucose, ketones and nitrite; microscopy only required if indicated to follow up abnormal findings). A pregnancy test will be performed for all women of childbearing potential.
- Blood samples for anti-GDNF antibodies and plasma GDNF levels. The samples will be stored at -70°C until central analysis after all subjects have completed the study.

Subjects will undergo NMSS (Section 5.7.1.5) and RT (Section 5.7.2.4.3) assessments at Weeks 12 and 24.

In subjects randomised in the Pilot Stage, T2-weighted and FLAIR 3T MRI will be completed within 2 hours of completion of the infusions at all post-baseline treatments. In Primary Study Stage subjects, no interim T2-weighted or FLAIR MRI monitoring will be done unless clinically mandated. However, Primary Study Stage subjects will receive another gadolinium contrast-containing test infusion of diluent, followed both by T1-weighted and by T2-weighted and FLAIR MRI scans, at any time within a 2-week window around Week 40.

In subjects randomised in the Pilot Stage, PET will be performed at Week 12. All subjects will undergo PET scanning at any time in the two weeks prior to Week 40.

5.6.5 Week 40 or Early Discontinuation

Week 40 will mark the completion of the study. A full safety and efficacy assessment will be performed, and this same assessment should be performed, if possible, for any subjects who discontinue the study before Week 40. The Week 40 visit must occur

±3 days of the scheduled date and may be completed over more than 1 day due to the number of assessments.

For this assessment, subjects will withhold all PD medications as follows:

- No PD medications will be taken after 6:00 PM on the night before the assessments and
- No long-acting PD medications will be taken on the day before the assessments.

Subjects will refrain from eating any high-protein foods on the morning of the assessments.

At the beginning of the assessment, all OFF period measures are to be completed first:

- UPDRS part II (ADL) and part III (motor) in OFF state (Section 5.7.1.1)
- Timed walking test (Section 5.7.1.2)
- Timed tapping test (Section 5.7.1.3)

A levodopa challenge will then be performed (refer to study-specific Standard Operating Procedures) and when the subject goes ON the following post-challenge tests will be performed:

- Full UPDRS (including parts I, II, III and IV; Section 5.7.1.1)
- Timed walking test (Section 5.7.1.2)
- Timed tapping test (Section 5.7.1.3)
- MDRS (Section 5.7.2.2)/MoCA (Section 5.7.2.1)
- Stroop (Section 5.7.2.3)
- FrSBe (Section 5.7.2.4.2)
- RT (Section 5.7.2.4.3)
- Verbal fluency assessment (Section 5.7.2.4.5)
- BDI (Section 5.7.2.5)
- PD NMSS (Section 5.7.1.5)
- PDQ-39 (Section 5.7.1.6)

- EQ-5D (Section 5.7.1.6)
- QUIP (Section 5.7.2.6)

The following procedures may be completed irrespective of the subject's ON/OFF status:

- SNAQ (Section 5.7.1.7).
- UPSIT (Section 5.7.2.7)
- Collection of subject PD fluctuation diaries (Section 5.7.1.4)
- Weight and height
- Vital signs: Seated systolic and diastolic BP, HR, RR and temperature will be assessed after the subject has been sitting quietly for at least 5 minutes. Standing systolic and diastolic BP and HR will also be assessed after the subject has been standing for 3 minutes.
- Brief physical examination, targeted, at the investigators discretion, to identify any notable changes from Screening Visit 1
- Standard 12-lead ECG. At least the following ECG parameters will be recorded: HR, PR, QT, QRS and QTc intervals. The report will be signed by the PI or designee and will be recorded in the CRF whether it is normal, abnormal but not clinically significant, or abnormal AND clinically significant (together with details of the abnormality).
- Clinical laboratory tests, including haematology (haematocrit, haemoglobin, MCH, MCHC, MCV, platelet count, RBC, WBC count and WBC differential [basophils, eosinophils, lymphocytes, monocytes and neutrophils]), serum chemistry (albumin, alkaline phosphatase, ALT, creatinine, eGFR, glucose, potassium, sodium, total bilirubin and urea) and urinalysis (colour, appearance, pH, glucose, ketones and nitrite; microscopy only required if indicated to follow up abnormal findings). A pregnancy test will be performed for all women of childbearing potential.

- Blood samples for anti-GDNF antibodies and plasma GDNF levels. The samples will be stored at -70°C until central analysis after all subjects have completed the study.
- Any changes to concomitant medications from the previous visit will be recorded. All over-the-counter or prescription medications, vitamins, and/or herbal supplements will be recorded in the CRF with their indications.
- Any AEs observed or reported since the previous visit will be recorded.

All subjects will undergo PET scanning at any time in the two weeks prior to Week 40.

Primary Study Stage subjects will receive another gadolinium contrast-containing test infusion of diluent, followed both by T1-weighted and by T2-weighted and FLAIR MRI scans, at any time within a 2-week window around Week 40. The infusion will be performed according to the same procedures as described for the test infusion in Section 5.5.3.

5.7 Outcomes Measures

5.7.1 Efficacy Outcome Measures

5.7.1.1 Unified Parkinson's Disease Rating Scale

The UPDRS (Appendix F) was developed to as an outcome measure for rating PD in clinical trials. It has 4 parts which predominantly measure the motor signs and symptoms of PD: Mentation, Behaviour and Mood (part I), ADLs (part II), Motor Examination (part III) and Complications of Therapy (part IV). Except for the motor examination which is performed at the assessment, all sections of the UPDRS rate the subject based on his/her state in the week preceding the assessment. Higher scores represent worse functioning.

In this trial all post-baseline UPDRS ratings will be performed by a trained rater who is blinded to all other aspects of the subject's condition. The motor UPDRS will be performed with the subject in a practically defined OFF state (at least 12 hours since last PD medication) and in the ON state after levodopa challenge. In order to qualify for the study, subjects will need a screening OFF period motor UPDRS between 25 and

45 and will need to experience at least a 40% improvement in motor UPDRS scores with levodopa challenge.

The primary outcome for this study will be the percentage change in motor UPDRS in the practically defined OFF state between baseline and Week 40. Other UPDRS endpoints include UPDRS part II in both the ON and OFF states, UPDRS part III in the ON state after levodopa challenge and total UPDRS scores. ON and OFF UPDRS ratings will be done during screening, at baseline and at Weeks 8, 16, 24, 32 and 40.

5.7.1.2 Timed Walking Test

During the timed walking test, the subject will walk as fast as possible 7 metres back and forth including turning. The time to perform this test is recorded. The subject will complete this test twice during the practically defined OFF period and twice in the ON state after levodopa challenge. The test will be performed during screening, at baseline and at Weeks 8, 16, 24, 32 and 40.

5.7.1.3 Timed Tapping Test

During the timed tapping test, the subject is instructed to alternate tapping the index finger between 2 points spaced 30 cm apart. Each hand is rated twice and the number of taps completed in 20 seconds on each side is to be recorded. This test is to be performed in the practically defined OFF state and in the ON state after levodopa challenge during screening, at baseline and at Weeks 8, 16, 24, 32 and 40.

5.7.1.4 Subject Diaries

Motor fluctuations in this study will be quantified using subject completed diaries (Appendix G). For 3 days prior to the relevant study assessments (not including the day immediately prior to the assessment, as the subject will be asked to withhold PD medication on that day), subjects are to record their state for every half-hour time period. Categories for rating include: ON with no dyskinesias, ON with non-troublesome dyskinesias, ON with troublesome dyskinesias, OFF or asleep. Caregivers may assist with the physical completion of the diary, however, the decision regarding the subject's state is to be made by the subject alone.

During screening, subjects will be trained on the completion of the diary and will have to demonstrate their ability to accurately determine their state prior to study entry by

comparing their own assessments to those of a qualified staff member over a 2-4 hour period. Subjects must be able to complete valid diaries (no more than 3 missing or double entries in a 24-hour period) and have an average of at least 2.5 hours of OFF time per day on their screening diaries to be eligible for study entry. If a subject is unable to provide valid diaries on their first attempt, one repeat attempt after retraining is permitted.

Duration of good ON time (ON without dyskinesias and ON with non-troublesome dyskinesias), OFF time, and ON time with troublesome dyskinesias will be secondary efficacy variables in this study. Duration of ON time with troublesome dyskinesias will also be discussed in the safety results section of the report.

Diaries will be collected during screening and at Weeks 8, 16, 24, 32 and 40. Diaries are to be reviewed by the coordinator each time they are returned, and retraining should be offered if errors in completion are noted. Diaries are to be dispensed at the visit prior to their collection.

5.7.1.5 Non-Motor Symptom Scale

The NMSS (Appendix H) is an interview-based scale developed to rate non-motor symptoms commonly occurring in PD. The 30 item scale rates symptoms which occurred in the preceding month in 9 domains – cardiovascular function including falls, sleep/fatigue, mood/cognition, perceptual problems/hallucinations, attention/memory, gastrointestinal, urinary, sexual function, and miscellaneous. Each item is rated from 0 (none) – 3 (severe) for severity and from 1 (rarely) to 4 (very frequent) and the score for each item is the product of the severity rating multiplied by the frequency.

The NMSS takes 20-30 minutes and is to be completed by the PI or designee when the subject is in the ON state. This scale is administered a total of 4 times in the study - during screening and at Weeks 12, 24 and 40.

5.7.1.6 PDQ-39/EQ-5D

The PDQ-39 (Appendix I) and the EQ-5D (Appendix J) are both subject self-report measures of quality of life. The PDQ-39 is a PD-specific quality of life tool with 39 questions. The EQ-5D is a generic quality of life scale with 5 questions and a health "thermometer." These scales will be completed when the subject is in the ON state

during their visit. The subject may receive assistance with the physical completion of the scales but the subject alone must determine the answer provided. The scales will take approximately 20 minutes for the subject to complete. The PDQ-39 and EQ-5D are completed 2 times in the study, during screening and at the end of treatment.

5.7.1.7 Simplified Nutritional Appetite Questionnaire

The 4-item SNAQ is a very brief measure of appetite. Posterior lateral putamen dopamine appears to be critical for eating, and restoration may induce improved appetite as a signal of efficacy [21]. Loss of appetite as an adverse event will be recorded if reported at study visits as will weight loss but applying this brief measure allows demonstration of improvement. The SNAQ is to be completed regardless of ON/OFF state, 2 times during the study, during screening and at Week 40.

5.7.2 Safety Outcome Measures

5.7.2.1 Montreal Cognitive Assessment

The MoCA (Appendix C) is a cognitive screening tool which assesses both cortical and subcortical function. It has 8 components – visuospatial/executive, naming, memory, attention, language, abstraction, delayed recall, and orientation, and the total score ranges from 0 – 30 with lower scores representing poorer cognitive function. It takes approximately 30 minutes to administer and will be performed 3 times in the study when the subject is in the ON state, during screening, post surgery, and at the end of treatment.

Any trained site personnel may complete this scale. Subjects must score a minimum of 24 on the MoCA to be eligible for the study.

5.7.2.2 Mattis Dementia Rating Scale (MDRS)

The MDRS is a global scale of cognition that is sensitive to the frontal/subcortical deficits that are common in PD. It includes 5 subscales – attention, initiation/ perseveration, construction, conceptualization and memory. Scores range from 0 – 144 with higher scores representing better cognitive function. In PD, scores < 123 are associated with some degree of dementia. The MDRS can be analysed using total scores or using individual subscale scores.

The MDRS must be administered by a trained rater with the subject in the ON state and takes 30 – 45 minutes to complete. It will be performed 2 times during this trial, during screening and at the end of treatment.

5.7.2.3 Stroop

The Stroop is a cognitive test that measures the capacity to direct attention. In this test subjects are presented with words that are the names of colours but are printed in a different colour of ink than the name represents (e.g., "green" printed in red). In the first component of the test, the subject is required to read the colour names and disregard the colour of the text. The second component requires the naming of the colour of the word instead of reading the word.

The Stroop takes 5-10 minutes to complete and is administered with the subject in the ON state 2 times in the study, during screening and at the end of treatment.

5.7.2.4 Other Brief Measures of Cognitive and Executive Function

Additional cognitive tests will be performed at screening, end of study and periodically during the course of the trial.

5.7.2.4.1 The National Adult Reading Test

The National Adult Reading Test (NART) is a widely accepted method of estimating premorbid intelligence levels in English speaking subjects. It is administered by a trained rater and consists of a list of 50 words with atypical phonemic pronunciation. Each word is presented individually and subjects are required to read aloud. There is no time limit for the completion of the test.

The NART will be performed during screening only, as a measure of premorbid intelligence.

5.7.2.4.2 Frontal Systems Behavioural Scale

The Frontal Systems Behavioural Scale (FrSBe) is a brief, reliable, 46-item self-report scale to assess behaviour associated with damage to frontal subcortical circuits. Each item is rated using a 5-point Likert scale and it produces a total score as well as three subscores (for apathy, disinhibition and executive dysfunction). The FrSBe will be completed by the subject, in the ON state, during screening and at Week 40.

5.7.2.4.3 Deary-Liewald Reaction Time

The Deary-Liewald Reaction Time (RT) is a computerized measure of simple and choice reaction time that has been validated in longitudinal studies and is suitable for older individuals. It will be performed, in the ON state, at screening and at Weeks 12, 24 and 40.

5.7.2.4.4 UPPS-P Impulsiveness Behaviour Scale

The UPPS-P Impulsiveness Behaviour Scale is a 59-item scale which measures impulsiveness in five subscales – Premeditation (lack of), Positive Urgency, Negative Urgency, Sensation Seeking and Perseverance (lack of). Each item is rated on a 4-point Likert scale. The UPPS-P will be performed at screening to provide a baseline assessment of subjects' propensity toward impulsive behaviour.

5.7.2.4.5 Verbal Fluency Assessment

Verbal fluency will be assessed by a trained rater using a standard brief paradigm to quantify this particular aspect of executive function. Verbal fluency will be recorded at baseline and at the end of the study.

5.7.2.5 Beck Depression Inventory

The BDI (Appendix D) is a depression scale that is commonly used both in clinical trials and in clinical practice. It has been recommended by the Movement Disorders Society as an outcome measure to rate the severity of depression in PD. The BDI is a 21-question subject self-report scale with each item being rated on a scale of 0 - 3. Higher scores represent greater degrees of depression.

The BDI will be completed by the subject in the ON state during screening and at the end of treatment. Only subjects with BDI scores of less than 20 are eligible for entry into the study.

5.7.2.6 Questionnaire for Impulsive- Compulsive Disorders in Parkinson's Disease

The QUIP (Appendix E) was developed to assess the occurrence of impulsive and compulsive disorders in PD. It is a subject self-administered scale which includes 13 questions covering symptoms related to the 4 commonest impulse control disorders in PD (gambling, sex, buying, and eating) as well as other behaviours and problematic

use of medication. The QUIP is to be completed by the subject while in the ON state during screening, at baseline and at Weeks 8, 16, 24, 32 and 40. Where possible, at these visits, a caregiver will also be requested to complete the assessments.

5.7.2.7 University of Pennsylvania Smell Identification Test

The UPSIT can be self-administered and uses microencapsulated odorants, which are released by scratching standardized odor-impregnated test booklets. The study uses the British version of the UPSIT. The test can identify most malingerers and is sensitive to age, gender, smoking habits, and a wide variety of olfactory disorders. The UPSIT can reliably identify and quantitate olfactory dysfunction in PD. The UPSIT is to be completed regardless of ON/OFF state, 2 times during the study, during screening and at Week 40.

5.8 PET Scanning Procedures and Image Analysis

^{18}F-DOPA PET will be performed at baseline and 9 months in all randomised subjects. Additional 3-month scans will be obtained from subjects randomised during the Pilot Stage.

For the PET scanning procedure, subjects will withhold all PD medications as follows:

- No PD medications will be taken after 6:00 PM on the night before the procedure and
- No long-acting PD medications will be taken on the day before the procedure.

Subjects will refrain from eating any high-protein foods on the morning of the procedure.

Upon arrival, subjects will receive 150 mg of carbidopa and 400 mg of entacapone (peripheral L-DOPA decarboxylase and catechol-o-methyl transferase inhibitors, respectively). After 1 hour, a low dose CT scan will be acquired (3 s) for attenuation correction and then 111 MBq of ^{18}F-DOPA in normal saline will be administered as an intravenous bolus at the start of scanning. Using a GE Discovery 690 PET/CT (GE Healthcare; Chalfont St. Giles, UK), dynamic images will be acquired as 26 time-frames over 94.5 min (1 × 30 sec, 4 × 1 min, 3 × 2 min, 3 × 3 min, and 15 × 5 min). The analyses will follow established procedures as described by Nandhagopal et al.

[22]. As a further refinement, co-registered MRI images will be used as a guidance on where to place the regions of interest, rather than just visual inspection.

5.9 Definition of End of Trial

The end of trial is the date of the last visit of the last subject.

5.10 Discontinuation/ Withdrawal of Subjects from Study Treatment

Each subject has the right to withdraw from the study at any time. In addition, the PI may discontinue a participant from the study at any time if considered necessary for any reason including:

- Pregnancy
- Ineligibility (either arising during the study or retrospectively, not having been identified at screening)
- Significant protocol deviation
- Significant non-compliance with treatment regimen or study requirements
- An AE which requires discontinuation of the study medication or results in inability to continue to comply with study procedures
- Disease progression which requires discontinuation of the study medication or results in inability to continue to comply with study procedures
- Consent withdrawn
- Lost to follow up

Subjects who discontinue the study early will complete all procedures and assessments as indicated for Week 40 (Section 5.6.5) unless they withdraw consent to do so.

The reason for withdrawal will be recorded in the CRF (withdrawal of consent or loss to follow-up will be recorded as such).

If the participant is withdrawn due to an AE, the PI or designee will arrange for follow-up visits or telephone calls until the AE has resolved or stabilised.

5.11 Source Data

Source documents are original documents, data, and records from which participants' CRF data are obtained. These include, but are not limited to, hospital records (from which medical history and previous and concurrent medication may be summarised into the CRF), clinical and office charts, laboratory and pharmacy records, diaries, microfiches, radiographs, and correspondence.

For the FrSBe, the subject will complete the assessment and the results will be transcribed into the CRF. For the other subject-completed questionnaires (PDQ-39, EQ-5D, QUIP, and SNAQ) and the diaries, the subject will complete the assessment directly into the CRF.

All documents will be stored safely in confidential conditions. On all study-specific documents, other than the signed consent, the participant will be referred to by the study participant number/code and initials, not by name.

6 TREATMENT OF TRIAL PARTICIPANTS

6.1 Description of Study Medication

The active drug is GDNF:

Chemical name:	Recombinant-methionyl human glial cell line-derived neurotrophic factor (r-metHuGDNF)
Physical description:	Clear, colourless liquid
Purity	Purity assumed to be 100% for dose calculation purposes
Concentration:	10 mg/mL in a buffer of 10 mM sodium citrate and 150 mM sodium chloride at pH 5.0
Container size:	2.0 mL
Fill size:	0.5 mL
Storage conditions:	-20 ± 5 °C

The placebo is artificial CSF (aCSF):

Physical description:	Clear, colourless liquid
Container size:	20.0 mL
Fill size:	19.8 mL (batch P02611), 19.6 mL (batch P06513)
Storage conditions:	15-30°C

GDNF drug substance is prepared on behalf of MedGenesis Therapeutix, Inc., by Lonza Ltd., Visp, Switzerland, GDNF drug product at Aptuit Ltd., Glasgow, UK.

The placebo is also manufactured on behalf of MedGenesis Therapeutix, Inc. at Aptuit Ltd., Glasgow, UK.

Both GDNF and aCSF will be stored at the study site Pharmacy in secure, limited-access and temperature-controlled conditions.

Commercial gadopentetate dimeglumine (Magnevist®) will be used at a 2 mM concentration in aCSF for test infusions at the end of the healing period and at Week 40 in Primary Study Stage subjects.

6.2 Description of Convection-Enhanced Delivery System

The drug delivery system comprises 4 microcatheters, 4 catheter guide tubes and a skull mounted transcutaneous drug delivery port. The microcatheters are connected under the scalp to separate in-line bacterial filters and further to the drug delivery port.

6.2.1 Implantation of the Drug Delivery System

Prior to surgical implantation an MRI scan will be acquired under general anaesthesia, and the implantation site for each catheter (2 in each putamen) will be determined from the images.

On the day of surgery the subject will be anaesthetised, and a reference frame will secured to the head prior to a CT head scan. The MRI treatment plan will be co-registered with the CT head scan taken in the reference frame.

When the patient is positioned on the operating table a surgical robot is used to assist the surgeon in placing 4 guide tubes to their relevant targets. The position of each guide tube will be confirmed with either a further CT scan or on-table radiography prior to insertion of the microcatheters. The 4 micro catheters will be connected to the individual in-line bacterial filters which are flat and round in shape, measuring 1.3 cm in diameter and 3 mm in height. The filters will be connected to the drug delivery port via separate connecting tubes, and the port is implanted in the skull in a position two finger breadths behind the tip of the left ear.

The port is a small cylinder measuring 5 mm in diameter and 1 cm in length and will be firmly secured in the bone such that it protrudes 5-6 mm above the bone surface. The scalp immediately around the protruding port will be thinned at the time of implantation so that the port protruding above the scalp will lie in a small recess approximately 2 cm in diameter. The thinned scalp in this region becomes hairless, facilitating maintenance of the transcutaneous port. Because of the port's low profile and small dimensions it should be hidden in the surrounding hair and not evident to others.

The skull mounted transcutaneous port has the same dimension and a similar implantation technique to that used for bone anchored hearing aids. These have been implanted in over 20,000 individuals, most commonly in children. Although some local skin reaction may occur in the post-operative period, it can be managed with topical antibiotic cream and local dressings. These local reactions typically resolve within a

few weeks and the need to remove a bone anchored hearing aid because of infection is rare, occurring in no more than 1% patients. The port needs to be maintained daily with hair washing and the use of a small brush to remove any debris that might accumulate at the base of the port where it enters the skin.

Apart from the port implantation site no other elements of the drug delivery system should be visible externally as they are of low profile (up to 3 mm in height) but may be just palpable under the scalp.

6.2.2 Drug Infusions

The drug administration system compromises 4 programmable syringe pumps, four 5-mL Plastipak syringes (Becton Dickinson), 4 sealed extension lines with male Luer attachments (each containing a septum) at one end, 4 double-sided syringe connectors with female Luer attachments (each holding an internal needle), 4 lengths of sealed drug administration tubing with male Luer attachments (each containing a septum) at both ends and a 4-channelled drug administration set. The latter compromises 4 needles mounted in a hub, each connected to independent bacteria and bubble filters and thence to short lengths of tubing, each with a female Luer attachment holding an internal needle. In addition, 3 syringe connectors with female Luer attachments at both ends and holding an internal needle at one end are used by the pharmacist preparing the study drug to fill the sealed extension lines and sealed drug administration lines.

Prior to drug administration, the pharmacist will fill the 4 lengths of drug administration tubing with an appropriate dose of drug (using 2 syringe connectors, one at each end of the sealed tubing), and label each line appropriately without identifying the treatment allocation. The pharmacist will further fill 4 syringes with aCSF, connect them to the open ends of the extension lines and fill the extension lines (using a single syringe connector at the opposite end to evacuate air). All syringe connectors will be disconnected and discarded once all lines have been filled. The filling of devices will take place up to 48 hours before a planned drug infusion.

For the infusion, each syringe/sealed extension line assembly will be connected to a double-sided-syringe connector, then to a drug administration line, then to one of the 4 channels of the drug administration set which prior to this has been flushed with aCSF.

Connection of the drug administration set to the port begins with the health professional cleaning the port and the immediate surrounds with an aseptic technique. The cylindrical needle hub of the drug administration set will be positioned over the port and locked to the port using a socket screw key. The 4 needles will be lowered through the port septum by use of a hand-tightened nut and thence individually guided into their separate channels that conduct the drug to identifiable catheters.

Once the connection has been secured, study medication is administered as detailed in Section 6.3. When the infusion is complete after approximately 90 to 150 minutes the administration set will be disconnected from the port by unscrewing both the nut and the socket screw.

6.3 Double-Blind Treatment

Subjects will receive either GDNF or placebo infusions every 4 weeks for 9 months:

- Group A, active treatment: 600 μL of 0.20 μg/μL GDNF in aCSF per putamen every 4 weeks
- Group B, placebo: 600 μL of aCSF per putamen every 4 weeks.

Study treatments will be prepared by an unblinded pharmacist at the study site based on the randomisation. Ready to use preparations of GDNF or aCSF will be provided to the PI or designee in identical containers identifiable only by codes unknown to the PI or any other study staff. Drug preparations guidelines are provided in a separate pharmacy manual.

At each treatment, infusion tubes will be connected with the transdermal port using standard aseptic technique. Every subject will receive study drug intraputamenally titrated up to an infusion rate of 3-5 μL/minute. The infusion rates will be up-titrated following a linear ramping scheme from 0 μL/min to 3-5 μL/min (0.18-0.30 mL/hour) over 30 to 40 minutes. The infusions will be delivered by the external syringe pumps with pre-programmed infusion algorithms (Perfusor® Space, B Braun, Melsungen, Germany).

Two catheters per putamen will be used, and 300 μL of study drug will be delivered per catheter at each treatment. This amounts to a dose of 120 μg GDNF per putamen per

administration in active subjects. An aCSF "flush" of up to 100 μL per catheter (at 3-5 μL/min) will clear the dead space of the implanted system after each infusion.

The infusions will be delivered by trained personnel. During the infusion, subjects will be semi-recumbent in a reclining chair. The total procedure time for the completion of the infusions will be approximately 90 to 150 minutes depending on potential reflux parameters observed on post infusion MRIs.

6.4 Compliance with Study Treatment

All treatments will be administered by PI or designee. Administration records will be kept on site, and administration information, including time and date of infusion, identification (blinded) of the infusate, infusion rate and duration, and the reasons for any interruptions of the infusion or for any missed or omitted infusions will be recorded in the CRF.

6.5 Accountability of the Study Treatment

It is forbidden to use investigational drug material for purposes other than as defined in this protocol.

All supplies of study medication will be accounted for in accordance with GCP. There will be an individual study drug accountability record for each subject and the pharmacist is to maintain accurate records of the disposition of all study medication supplies received and dispensed during the study. These records will include the amounts and dates clinical drug supplies were received, dispensed to the PI or designee for any given subject, and returned to the manufacturer. If errors or damages in the clinical drug supply shipments occur, the pharmacist will notify the manufacturer immediately so that corrective action can be taken as needed. Copies of the study medication accountability records will be provided by the pharmacist for inclusion in the Trial Master File (TMF) after database lock. The study monitor will periodically check the supplies of study medication held by the pharmacist to verify accountability of all medication used.

The PI or designee will administer the medication only to the identified subjects of this study, according to the procedures described in this study protocol. After the end of the study, all unused medication and all medication containers can be destroyed on-site as

long as proper documentation is supplied. If destruction on-site is not possible then medication and all medication containers will be returned to the manufacturer for destruction and documentation will be returned to the manufacturer. The manufacturer will verify that a final report of drug accountability is prepared and maintained in the Investigator's Study Centre File.

6.6 Test Infusions

A test infusion of diluent will be administered at the end of the healing period in all subjects. In Primary Study Stage subjects, the test infusion will contain gadolinium contrast. A repeat test infusion of gadolinium contrast-containing diluent will be given to Primary Study Stage subjects at Week 40.

The infusions will be freshly prepared on site prior to use following the steps described for study medication above. Specifically, for Primary Study Stage subjects, 4 lengths of drug administration tubing (each holding 300 μL) will be filled 2 mM gadopentetate dimeglumine (1:250 dilution of Magnevist®). The drug administration lines will then be connected as specified in Section 6.2.2, and the infusate will be delivered according to the infusion protocol provided in Section 6.3.

6.7 Concomitant Medication

Subjects should have PD medication optimized and stabilized prior to study entry. Every effort should be made not to increase subjects' medication for PD during the study, but medication may be increased if required for to maintain the subject's well being. PD medication dose may be decreased at the discretion of the PI to manage PD drug-related side-effects. Non-PD medications may be altered at any time at the PI's discretion.

Throughout the study the PI may prescribe any concomitant medications or treatments deemed necessary to provide adequate supportive care.

Any medication other than the study medication, including prescription or over-the-counter medications, vitamins and herbal medications taken during the study will be recorded in the CRF with their indications.

7 SAFETY REPORTING

7.1 Definitions

7.2 Adverse Event (AE)

An AE or adverse experience is:

Any untoward medical occurrence in a subject or clinical investigation participant administered a medicinal product, which does not necessarily have a causal relationship with this treatment (the study medication).

An AE can therefore be any unfavourable and unintended sign (including an abnormal laboratory finding), symptom or disease temporally associated with the use of the study medication, whether or not considered related to the study medication.

7.3 Adverse Reaction (AR)

All untoward and unintended responses to a medicinal product related to any dose.

The phrase "responses to a medicinal products" means that a causal relationship between a study medication and an AE is at least a reasonable possibility, i.e., the relationship cannot be ruled out.

All cases judged by either the reporting medically qualified professional or the Sponsor as having a reasonable suspected causal relationship to the study medication qualify as ARs.

7.4 Serious Adverse Event (SAE)

An SAE is any untoward medical occurrence that at any dose:

- Results in death
- Is life-threatening; NOTE: The term "life-threatening" in the definition of "serious" refers to an event in which the participant was at risk of death at the time of the event; it does not refer to an event which hypothetically might have caused death if it were more severe.
- Requires inpatient hospitalisation or prolongation of existing hospitalisation
- Results in persistent or significant disability/incapacity

- Is a congenital anomaly/birth defect
- Other important medical events; NOTE: Other events that may not result in death, are not life-threatening, or do not require hospitalisation, may be considered an SAE when, based upon appropriate medical judgement, the event may jeopardise the subject and may require medical or surgical intervention to prevent one of the outcomes listed above.

To ensure no confusion or misunderstanding of the difference between the terms "serious" and "severe," which are not synonymous, the following note of clarification is provided:

The term "severe" is often used to describe the intensity (severity) of a specific event (as in mild, moderate, or severe myocardial infarction); the event itself, however, may be of relatively minor medical significance (such as severe headache). This is not the same as "serious," which is based on subject/event outcome or action criteria usually associated with events that pose a threat to a participant's life or functioning as defined in the bullet points above. Seriousness (not severity) serves as a guide for defining regulatory reporting obligations.

7.5 Serious Adverse Reaction (SAR)

An AE (expected or unexpected) that is both serious and, in the opinion of the reporting Investigator, believed with reasonable probability to be due to one of the study treatments, based on the information provided.

7.6 Suspected Unexpected Serious Adverse Reaction (SUSAR)

An SAR, the nature or severity of which is not consistent with the applicable product information (e.g., Investigator's Brochure for an unapproved investigational product or Summary of Medicinal Product Characteristics [SMPC] for an approved product).

7.7 Causality and Expectedness

The relationship of each AE to the trial medication must be determined by a medically qualified individual according to the following definitions:

Related: The AE follows a reasonable temporal sequence from trial medication administration. It cannot reasonably be attributed to any other cause.

Not Related: The AE is probably produced by the participant's clinical state or by other modes of therapy administered to the participant.

The expectedness of each **SAE** in the trial must be determined by a medically qualified individual according to the following definition:

Unexpected Adverse Drug Reaction: An adverse reaction, the nature or severity of which is not consistent with the applicable product information (i.e., Investigator's Brochure).

7.8 Procedures for Recording Adverse Events

All AEs occurring during the study from the time of screening (first consent) until the Week 40 visit has been completed (or 28 days after the last dose of study medication for subjects who discontinue the study early), observed by the PI or designee or reported by the participant, whether or not attributed to study medication, will be recorded on the AE pages of the CRF.

The following information will be recorded: description, date and time of onset, date and time of end, occurrence during infusion, severity, assessment of relatedness to study medication or device, seriousness, action taken, and outcome. Follow-up information should be provided as necessary.

AEs considered related to the study medication or to the device as judged by the PI or a medically qualified designee will be followed until resolution or the event is considered stable. All related AEs that result in a participant's withdrawal from the study or are present at the end of the study, should be followed up until a satisfactory resolution occurs.

It will be left to the PI's or medically qualified designee's clinical judgment whether or not an AE is of sufficient severity to require the participant's removal from treatment. A participant may also voluntarily withdraw from treatment due to what he or she perceives as an intolerable AE. If either of these occurs, the participant must undergo an end of study assessment (unless he or she has withdrawn consent to do so) and be given appropriate care under medical supervision until symptoms cease or the condition becomes stable.

The severity of events will be assessed on the following scale:
1 = mild, 2 = moderate, 3 = severe.

The relationship of AEs to the study medication will be assessed by the PI or a medically qualified designee.

Any pregnancy occurring during the clinical study and the outcome of the pregnancy should be recorded and followed up for any congenital abnormality or birth defect.

7.9 Reporting Procedures for Serious Adverse Events

All SAEs must be reported to North Bristol NHS Trust Research and Innovation (NBT R&I) within 1 working day of discovery or notification of the event. NBT R&I will perform an initial check of the report, request any additional information and ensure it is reviewed. All SAEs must be reviewed at the next Trial Safety Group meeting. All SAE information must be recorded on an SAE form and faxed or emailed to NBT R&I. Additional information received for a case (follow-up or corrections to the original case) need to be detailed on a new SAE form and faxed or emailed to NBT R&I.

7.10 SUSAR Reporting

The PI will support NBT R&I to report all SUSARs to the MHRA, the local REC, and the manufacturer of the drug. Fatal or life-threatening SUSARs must be reported within 7 days and all other SUSARs within 15 days. The PI will inform all study staff concerned of relevant information about SUSARs that could adversely affect the safety of participants.

7.11 Annual Safety Reports

In addition to the expedited reporting above, the PI shall submit once a year throughout the clinical trial or on request a safety report to the MHRA, REC, and manufacturer of the drug.

8 STATISTICS

A full, detailed statistical analysis plan (SAP) will be prepared and completed before study data are unblinded.

8.1 Description of Statistical Methods

The primary analysis will compare the active group with placebo using a mixed-effect model with repeated measures (MMRM) to compare the active group with placebo in all randomised Primary Study Stage subjects. The response variable will be the primary endpoint: the percentage change from baseline in the motor UPDRS in the OFF-state at 9 months, with motor UPDRS in the OFF-state at baseline as a covariate. In addition, sensitivity analyses employing different study populations or baseline values and an analysis of covariance model will be performed.

Secondary and supplementary PD outcome variables based on percent change from baseline and change from baseline will be analysed similarly to the primary endpoint for continuous variables and with corresponding categorical analyses for categorical variables.

Descriptive statistics will be presented for all endpoints by treatment group supporting the primary and secondary analyses. No multiplicity adjustments are planned for this phase II study, and all hypothesis tests will be run with 2-sided alpha = 0.05. Details of all analyses will be provided in the SAP.

Safety data will be presented descriptively, by treatment group, with standard Medical Dictionary for Regulatory Activities (MedDRA) coded AE and SAE frequency and incidence tables as well as shift tables for clinical laboratory parameters.

8.2 Number of Participants

The endpoint used as the basis for the sample size calculation of the Primary Study Population is the percentage change from baseline to 9 months in motor UPDRS in the practically defined OFF state. Assuming a standard deviation of 20%, a 2-sided type I error of 5%, a power of 80% and a difference of 20 points in % change in motor UPDRS from baseline (e.g., a response in the active group of 25% and a placebo response of 5% or a response in the active group of 35% and a placebo response of 15%), a total of 34 evaluable subjects, randomised on a 1:1 basis, will be required to

complete the study. As a point of reference, a 5% placebo response rate was seen in the previous phase II study [11]. To compensate for a potential 5% loss of subjects for the evaluation of the primary endpoint at 9 months (with last observations at earlier time points carried forward to 9 months), a total of 36 subjects will be randomised in the Primary Study Stage and will constitute the primary analysis population.

8.3 Level of Statistical Significance

The study design assumes a 2-sided type I error of 5%, equating to a p-value of < 0.05, in all analyses.

8.4 Criteria for the Termination of the Trial

The study will be monitored by an independent DMC established by the sponsor or designee and governed by a separate charter. The responsibilities of the DMC will include performing the safety analysis after the last pilot subject has completed 3 months of treatment. The DMC may recommend termination of the trial at any point for safety reasons.

This study may be terminated by the Sponsor. The study may also be terminated prematurely at any time when agreed to by both the PI and the Sponsor as being in the best interests of subjects, and justified on either medical or ethical grounds. In terminating the study, the Sponsor and the PI will ensure that adequate consideration is given to the protection of the subjects' interests.

8.5 Procedure for Accounting for Missing, Unused, and Spurious Data

Methods for handling missing or incomplete data will be described in the SAP.

8.6 Procedures for Reporting any Deviation(s) from the Original Statistical Plan

Changes to the SAP planned before study data are unblinded will be documented in a revised SAP or SAP addendum or amendment issued before unblinding. Any changes to the planned analyses made after unblinding will be clearly identified in the final Clinical Study Report.

8.7 Inclusion in Analysis

The primary analysis will be conducted after all Primary Study Stage subjects have reached the 9-month endpoint.

The primary analysis population will include all subjects randomised to the study in the Primary Study Stage of the study. The primary analysis will follow the ITT principle, with all subjects randomised in the Primary Study Stage included and grouped according to randomised treatment (regardless of treatment received). Subjects randomised in the Pilot Stage will not be included in the ITT population for the primary analysis as the treatment parameters may be altered during this phase.

In addition, efficacy analyses will be repeated on the ITT Overall Population comprising all randomised Primary Stage subjects and all randomised Pilot Stage subjects and on the Per Protocol Population, comprising all subjects treated in the study (i.e., including subjects randomised in the Pilot Stage) and without major protocol violations. Misrandomisations constitute major protocol violations.

The Safety Primary Population will comprise all Primary Study Stage subjects who received at least one dose of study medication, with subjects grouped according to treatment actually received. All safety analyses will be performed on the Safety Primary Population. More limited analyses will be performed for the Safety Overall Population comprising all subjects (both Pilot and Primary Study Stage) who received at least one dose of study medication, with subjects grouped by treatment actually received. Safety data for Pilot Stage subjects will be provided and evaluated via individual data listings. Pre-treatment AEs will be analysed in the Safety Enrolled Population comprising all enrolled subjects (Primary Study Stage and Pilot Stage subjects combined).

9 DIRECT ACCESS TO SOURCE DATA/DOCUMENTS

Direct access will be granted to authorised representatives from the Sponsor, host institution, manufacturer, and the regulatory authorities to permit trial-related monitoring, audits and inspections.

10 QUALITY CONTROL AND QUALITY ASSURANCE PROCEDURES

The study will be conducted in accordance with the current approved protocol, International Conference on Harmonisation (ICH) Good Clinical Practices (GCP), relevant regulations and standard operating procedures.

Regular monitoring will be performed according to ICH GCP. The Sponsor or designee will complete a monitoring plan and provide a copy for the TMF. Data will be evaluated for compliance with the protocol and accuracy in relation to source documents. Following written standard operating procedures, the monitors will verify that the clinical trial is conducted and data are generated, documented and reported in compliance with the protocol, GCP and the applicable regulatory requirements.

The Sponsor or designee will establish an independent DMC. The establishment and operation of the DMC will adhere to relevant guidance, and the responsibilities and processes of the DMC will be specified in a DMC charter.

11 SERIOUS BREACHES

The Medicines for Human Use (Clinical Trials) Regulations contain a requirement for the notification of "serious breaches" to the MHRA within 7 days of the Sponsor becoming aware of the breach.

A serious breach is defined as "A breach of GCP or the trial protocol which is likely to affect to a significant degree –

(a) the safety or physical or mental integrity of the subjects of the trial; or

(b) the scientific value of the trial."

In the event that a serious breach is suspected NBT R&I should be contacted as soon as possible. Breaches of GCP will be managed in accordance with ISOP-C05 Noncompliance with ICH Good Clinical Practice (GCP) Guidelines (http://www.nbt.nhs.uk/education__research/researcher_resources/useful_documents__forms/standard_operating_procedures.aspx)

12 ETHICS

12.1 Declaration of Helsinki

The PI will ensure that this study is conducted in accordance with the principles of the Declaration of Helsinki (as amended by the World Medical Association in Seoul, Republic of Korea, October 2008).

12.2 ICH Guidelines for Good Clinical Practice

The PI will ensure that this study is conducted in full conformity with relevant regulations and with the ICH Guidelines for GCP (CPMP/ICH/135/95) July 1996.

12.3 Approvals

The protocol, informed consent form, participant information sheet and any proposed advertising material will be submitted to the appropriate REC, MHRA, and applicable host institution(s) for written approval.

The PI will submit and, where necessary, obtain approval from the above parties for all substantial amendments to the original approved documents.

12.4 Participant Confidentiality

The trial staff will ensure that the participants' anonymity is maintained. The participants will be identified only by initials and a participant identification number in the CRF and any written study materials. All documents will be stored securely and only accessible by trial staff and authorised personnel. The study will comply with the Data Protection Act which requires data to be anonymised as soon as it is practical to do so.

13 DATA HANDLING AND RECORD KEEPING

Paper CRFs will be used for the current study and a Data Management Plan will be prepared by the Sponsor or designee. The CRF data will be compared to source documentation by the study monitors. All monitored data will be processed by a data management group. Data edit checks will be performed using both CRFs and SAS® programming as detailed in the Data Management Plan. Data queries will be issued to the clinical site in order to resolve any discrepancies found during the discrepancy management process, and data will be updated accordingly. The participants will be identified by a study specific participant number and/or code in any database. The name and any other identifying detail will NOT be included in any study data electronic file.

Previous and concomitant medications will be coded using the latest available World Health Organization (WHO) Drug Reference Dictionary. Coexistent diseases and AEs will be coded using MedDRA.

When the database has been declared to be complete and accurate, it will be locked. Any changes to the database after that time can only be made by written agreement and documented in the TMF.

14 FINANCE AND INSURANCE

14.1 Compensation for Harm

The Sponsor undertakes to maintain an appropriate clinical study insurance policy.

Deviations from the study protocol - especially the prescription of a dose other than that scheduled in the study protocol, other modes of administration, other indications, and longer treatment periods - are not permitted and shall not be covered by the statutory subject insurance scheme.

15 PUBLICATION POLICY

Authorship of any publication will be based upon substantial contribution to the design, analysis, interpretation of data, drafting and/or critically revising any manuscript(s) derived from the Study. The Sponsor, PI and/or Study Neurosurgeon shall have the right to publish the results of the Study. Intended publications employing data from the study will be discussed with all members of the Steering Committee in advance of writing up to allow all members to contribute.

The Drug Manufacturer must receive copies of any intended communication at least sixty (60) days in advance to review a manuscript and fifteen (15) days to review any poster presentation, abstract or other written or oral material which describes the results of the Study. In addition, if Drug Manufacturer requests in writing, the Sponsor, PI and/or Study Neurosurgeon shall withhold any publication or presentation an additional thirty (30) days to allow for further clarification.

16 REFERENCES

[1] Lin LF, Doherty DH, Lile JD, Bektesh S, Collins F. GDNF: a glial cell line-derived neurotrophic factor for midbrain dopaminergic neurons. Science 1993;260(5111):1130-2.

[2] Airaksinen MS, Saarma M. The GDNF family: signalling, biological functions and therapeutic value. Nat Rev Neurosci 2002;3(5):383-94.

[3] Gash DM, Zhang Z, Ovadia A, Cass WA, Yi A, Simmerman L, Russell D, Martin D, Lapchak PA, Collins F, Hoffer BJ, Gerhardt GA. Functional recovery in parkinsonian monkeys treated with GDNF. Nature 1996;380(6571):252-5.

[4] Zhang Z, Miyoshi Y, Lapchak PA, Collins F, Hilt D, Lebel C, Kryscio R, Gash DM. Dose response to intraventricular glial cell line-derived neurotrophic factor administration in parkinsonian monkeys. J Pharmacol Exp Ther 1997;282(3):1396-401.

[5] Grondin R, Zhang Z, Yi A, Cass WA, Maswood N, Andersen AH, Elsberry DD, Klein MC, Gerhardt GA, Gash DM. Chronic, controlled GDNF infusion promotes structural and functional recovery in advanced parkinsonian monkeys. Brain 2002;125(Pt 10):2191-201.

[6] Kordower JH, Emborg ME, Bloch J, Ma SY, Chu Y, Leventhal L, McBride J, Chen EY, Palfi S, Roitberg BZ, Brown WD, Holden JE, Pyzalski R, Taylor MD, Carvey P, Ling Z, Trono D, Hantraye P, Deglon N, Aebischer P. Neurodegeneration prevented by lentiviral vector delivery of GDNF in primate models of Parkinson's disease. Science 2000;290(5492):767-73.

[7] Palfi S, Leventhal L, Chu Y, Ma SY, Emborg M, Bakay R, Deglon N, Hantraye P, Aebischer P, Kordower JH. Lentivirally delivered glial cell line-derived neurotrophic factor increases the number of striatal dopaminergic neurons in primate models of nigrostriatal degeneration. J Neurosci 2002;22(12):4942-54.

[8] Nutt JG, Burchiel KJ, Comella CL, Jankovic J, Lang AE, Laws ER, Jr., Lozano AM, Penn RD, Simpson RK, Jr., Stacy M, Wooten GF. Randomized, double-blind trial of glial cell line-derived neurotrophic factor (GDNF) in PD. Neurology 2003;60(1):69-73.

[9] Gill SS, Patel NK, Hotton GR, O'Sullivan K, McCarter R, Bunnage M, Brooks DJ, Svendsen CN, Heywood P. Direct brain infusion of glial cell line-derived neurotrophic factor in Parkinson disease. Nat Med 2003;9(5):589-95.

[10] Slevin JT, Gerhardt GA, Smith CD, Gash DM, Kryscio R, Young B. Improvement of bilateral motor functions in patients with Parkinson disease through the unilateral intraputaminal infusion of glial cell line-derived neurotrophic factor. J Neurosurg 2005;102(2):216-22.

[11] Lang AE, Gill S, Patel NK, Lozano A, Nutt JG, Penn R, Brooks DJ, Hotton G, Moro E, Heywood P, Brodsky MA, Burchiel K, Kelly P, Dalvi A, Scott B, Stacy M, Turner D, Wooten VG, Elias WJ, Laws ER, Dhawan V, Stoessl AJ, Matcham J, Coffey RJ, Traub M. Randomized controlled trial of intraputamenal glial cell line-derived neurotrophic factor infusion in Parkinson disease. Ann Neurol 2006;59(3):459-66.

[12] Bobo RH, Laske DW, Akbasak A, Morrison PF, Dedrick RL, Oldfield EH. Convection-enhanced delivery of macromolecules in the brain. Proc Natl Acad Sci U S A 1994;91(6):2076-80.

[13] Fiandaca MS, Forsayeth JR, Dickinson PJ, Bankiewicz KS. Image-guided convection-enhanced delivery platform in the treatment of neurological diseases. Neurotherapeutics 2008;5(1):123-7.

[14] Barnett MW, Fisher CE, Perona-Wright G, Davies JA. Signalling by glial cell line-derived neurotrophic factor (GDNF) requires heparan sulphate glycosaminoglycan. J Cell Sci 2002;115(Pt 23):4495-503.

[15] Hovland DN, Jr., Boyd RB, Butt MT, Engelhardt JA, Moxness MS, Ma MH, Emery MG, Ernst NB, Reed RP, Zeller JR, Gash DM, Masterman DM, Potter BM, Cosenza ME, Lightfoot RM. Six-month continuous intraputamenal infusion toxicity study of recombinant methionyl human glial cell line-derived neurotrophic factor (r-metHuGDNF) in rhesus monkeys. Toxicol Pathol 2007;35(5):676-92.

[16] Tatarewicz SM, Wei X, Gupta S, Masterman D, Swanson SJ, Moxness MS. Development of a maturing T-cell-mediated immune response in patients with idiopathic Parkinson's disease receiving r-metHuGDNF via continuous intraputaminal infusion. J Clin Immunol 2007;27(6):620-7.

[17] Yu LY, Jokitalo E, Sun YF, Mehlen P, Lindholm D, Saarma M, Arumae U. GDNF-deprived sympathetic neurons die via a novel nonmitochondrial pathway. J Cell Biol 2003;163(5):987-97.

[18] Love S, Plaha P, Patel NK, Hotton GR, Brooks DJ, Gill SS. Glial cell line-derived neurotrophic factor induces neuronal sprouting in human brain. Nat Med 2005;11(7):703-4.

[19] Hadaczek P, Johnston L, Forsayeth J, Bankiewicz KS. Pharmacokinetics and bioactivity of glial cell line-derived factor (GDNF) and neurturin (NTN) infused into the rat brain. Neuropharmacology 2010;DOI:10.1016/j.neuropharm.2010.02.002.

[20] Salvatore MF, Ai Y, Fischer B, Zhang AM, Grondin RC, Zhang Z, Gerhardt GA, Gash DM. Point source concentration of GDNF may explain failure of phase II clinical trial. Exp Neurol 2006;202(2):497-505.

[21] Sotak BN, Hnasko TS, Robinson S, Kremer EJ, Palmiter RD. Dysregulation of dopamine signaling in the dorsal striatum inhibits feeding. Brain Res 2005;1061(2):88-96.

[22] Nandhagopal R, Kuramoto L, Schulzer M, Mak E, Cragg J, Lee CS, McKenzie J, McCormick S, Samii A, Troiano A, Ruth TJ, Sossi V, de la Fuente-Fernandez R, Calne DB, Stoessl AJ. Longitudinal progression of sporadic Parkinson's disease: a multi-tracer positron emission tomography study. Brain. 2009;132(Pt 11):2970-9.

17 APPENDIX A: STUDY SCHEDULE OF EVENTS

Table 1 Schedule of Events (Presurgery; Surgery and Healing)

Assessments	Screening Visit 1 [a]	Screening Visit 2 [a,b]	Surgery and Healing Phase [c]
Informed consent [d]	X		X [e]
Demography	X		
Medical history, including PD history	X		
Hoehn and Yahr staging	X		
Physical examination [f]	X		
Clinical laboratory testing [g]	X		
Anti-GDNF antibody levels and GDNF plasma concentration	X		
ECG	X		
Vital signs [h], weight, height	X		X
MoCA	X		X [j]
MDRS	X		
BDI		X	
Stroop test		X	
NART		X	
FrSBe		X	
RT		X	
UPPS-P		X	
Verbal fluency		X	
QUIP		X	
MRI		X [i]	X [j]
UPDRS part II and part III in OFF state		X	
Timed walking test in OFF state		X	
Timed tapping test in OFF state		X	
Levodopa challenge		X	
UPDRS in ON state		X	
Timed walking test in ON state		X	
Timed tapping test in ON state		X	
PD fluctuation diary training	X		
Dispense PD fluctuation diaries	X		X
Collect PD fluctuation diaries		X	
PD NMSS		X	
PDQ-39		X	
EQ-5D		X	
SNAQ		X	
UPSIT		X	
Concomitant medications [k]	X	X	X
Catheter placement			X
Post-operative CT scan			X
Test infusion of aCSF			X [j]
Glasgow Coma Scale [l]			X
Adverse events [m]	X	X	X

a Assessments need not be completed in a single visit, provided all assessments are within 60 days of surgery.

b No PD medications will be taken after 6:00 PM on the night before the assessments and no long-acting PD medications will be taken on the day before the assessments. Subjects will refrain from eating any high-protein foods on the morning of the assessments.

c All surgical procedures will be carried out according to institutional practices and under the direction of the Study Neurosurgeon. Subjects will remain hospitalised post-surgery for a period of time determined by the Study Neurosurgeon or designee and based on the individual subject's course of recovery.

d Informed consent must be obtained before any study-specific procedures or assessments are performed.

e Following completion of screening procedures, subjects will be reconsented prior to undergoing any surgical procedures.

f Full physical examination, including assessments of body systems. Findings on screening physical examination will be recorded as medical history.

g Haematology (haematocrit, haemoglobin, MCH, MCHC, MCV, platelet count, RBC count, WBC count and WBC differential [basophils, eosinophils, lymphocytes, monocytes and neutrophils]), serum chemistry (albumin, alkaline phosphatase, ALT, creatinine, eGFR, glucose, potassium, sodium, total bilirubin and urea) and urinalysis (colour, appearance, pH, glucose, ketones, nitrite and microscopy). A pregnancy test will be performed for all women of childbearing potential (women not of childbearing potential are those who are surgically sterile or are > 45 years of age and without menses for ≥ 2 years).

h Supine systolic and diastolic BP, HR, RR and temperature will be assessed after the subject has been resting quietly for at least 5 minutes. Standing systolic and diastolic BP and HR will also be assessed after the subject has been standing for 3 minutes. In *Pilot Stage subjects*, during administration of the test infusion, whilst in the scanner, HR, RR and oxygen saturation will be monitored continuously, and systolic and diastolic BP will be collected every 15 minutes. Both seated and standing systolic and diastolic BP and HR will be assessed within 30 minutes after the end of the infusion.

In *Primary Study Stage subjects*, during administration of the test infusion, seated vital signs (systolic and diastolic BP, HR and RR) will be collected every 15 minutes during the first infusion in any subject and every 30 minutes during subsequent infusions, and both seated and standing systolic and diastolic BP and HR will be assessed within 30 minutes after the end of the infusion.

i T2-weighted and FLAIR 3T MRI; for surgical planning and baseline scan for safety.

j For subjects who complete the healing period, an infusion of aCSF will be performed and T2-weighted and FLAIR 3T MRI scans completed within 2 hours for assessment of catheter patency and drug distribution. Pilot Stage subjects will be receiving the aCSF infusion in the MRI suite to allow for additional real-time MR imaging. In Primary Study Stage subjects, the aCSF infusion will contain gadolinium contrast and a T1-weighted MRI scan will also be obtained. A second aCSF infusion and MRI will be performed if infusion parameters are changed. MoCA will be performed on the same day as the test infusion.

k Concomitant medications, including all over-the-counter or prescription medications, vitamins, and herbal supplements, from 60 days before the planned surgery and until Week 40 or early discontinuation will be recorded. All over-the-counter or prescription medications, vitamin and/or herbal supplements will be recorded in the CRF with their indications.

l Performed before infusion, 30 minutes into the infusion, and after completion of the infusion.

m All AEs occurring from the time of screening must be recorded as part of the subject's medical record. AEs meeting the criteria for SAEs (Section 7.4) from the time of screening must be reported as directed in Section 7.9. All AEs occurring from the time of screening (first consent) until the Week 40 visit has been completed (or 28 days after the last dose of study medication for subjects who discontinue the study early) will be recorded on the AE pages of the CRF.

Table 2 Schedule of Events (Double-Blind Treatment)

	Week										
**Procedure/Assessments**	**0[c]**	**4**	**8[c]**	**12**	**16[c]**	**20**	**24[c]**	**28**	**32[c]**	**36**	**40[c]**
MRI (Pilot subjects)[a]	X	X	X	X	X	X	X	X	X	X	
MRI (Primary Study Stage subjects)[b]											X
PET	X[d]			X[e]							X[f]
Vital signs[g]	X	X	X	X	X	X	X	X	X	X	X
Weight and height	X		X		X		X		X		X
Physical examination[h]											X
ECG											X
Laboratory assessment[i]		X			X			X			X
Anti-GDNF antibody levels and GDNF plasma concentration		X			X			X			X
UPDRS part II and part III in OFF state	X		X		X		X		X		X
Timed walking test in OFF state	X		X		X		X		X		X
Timed tapping test in OFF state	X		X		X		X		X		X
Levodopa challenge	X		X		X		X		X		X
UPDRS in ON state	X		X		X		X		X		X
Timed walking test in ON state	X		X		X		X		X		X
Timed tapping test in ON state	X		X		X		X		X		X
PDQ-39											X
EQ-5D											X
MoCA & MDRS											X
Stroop test											X
FrSBe											X
RT				X			X				X
Verbal fluency											X
SNAQ											X
UPSIT											X
NMSS				X			X				X
BDI											X
QUIP	X		X		X		X		X		X
Collect PD fluctuation diaries	X		X		X		X		X		X
Dispense PD fluctuation diaries		X		X		X		X		X	
Randomization	X										
Infusion of study drug	X	X	X	X	X	X	X	X	X	X	
Glasgow Coma Scale[j]	X	X	X	X	X	X	X	X	X	X	X
Adverse events[k]	X	X	X	X	X	X	X	X	X	X	X
Concomitant medications[l]	X	X	X	X	X	X	X	X	X	X	X

a T2-weighted and FLAIR 3T MRI; are to be completed within 2 hours of study drug infusion in Pilot Stage subjects at all time points. Also, Pilot Stage subjects will be receiving the Week 0 infusion in the MRI suite to allow for additional real-time MR imaging.

b T1-weighted as well as T2-weighted and FLAIR 3T MRI scans; are to be completed within 2 hours of a gadolinium contrast-containing test infusion of diluent at Week 40 (±1 week). No interim T2-weighted or FLAIR MRI monitoring will be done in Primary Study Stage subjects unless clinically mandated.

c At Weeks 0, 8, 16, 24, 32 and 40, no PD medications will be taken after 6:00 PM on the night before the assessments and no long-acting PD medications will be taken on the day before the assessments. Subjects will refrain from eating any high-protein foods on the morning of the assessments.

d The Week 0 PET may be performed any time after randomisation and before the first infusion with study drug.

e Subjects randomised during the Pilot Stage only.

f The Week 40 PET may be performed at any time in the two weeks prior to Week 40.

g Seated systolic and diastolic BP, HR, RR and temperature will be assessed after the subject has been sitting quietly for at least 5 minutes. Standing systolic and diastolic BP and HR will also be assessed after the subject has been standing for 3 minutes. During drug administration, seated vital signs (systolic and diastolic BP, HR and RR) will be collected every 15 minutes during the first infusion in any subject and every 30 minutes during subsequent infusions, and both seated and standing systolic and diastolic BP and HR will be assessed within 30 minutes after the end of the infusion.

h Brief physical examination, targeted, at the Investigator's discretion, to identify changes from Screening Visit 1.

i Haematology (haematocrit, haemoglobin, MCH, MCHC, MCV, platelet count, RBC count, WBC count and WBC differential [basophils, eosinophils, lymphocytes, monocytes and neutrophils]), serum chemistry (albumin, alkaline phosphatase, ALT, creatinine, eGFR, glucose, potassium, sodium, total bilirubin and urea) and urinalysis (colour, appearance, pH, glucose, ketones and nitrite; microscopy as required to follow up abnormal observations). A pregnancy test will be performed for all women of childbearing potential (women not of childbearing potential are those who are surgically sterile or are > 45 years of age and without menses for ≥ 2 years).

j Performed before infusion, 30 minutes into the infusion, and after completion of the infusion.

k All AEs occurring from the time of screening must be recorded as part of the subject's medical record. Adverse events meeting the criteria for SAEs (Section 7.4) from the time of screening must be reported as directed in Section 7.9. All AEs occurring from the time of screening (first consent) until the Week 40 visit has been completed (or 28 days after the last dose of study medication for subjects who discontinue the study early) will be recorded on the AE pages of the CRF.

l Concomitant medications, including all over-the-counter or prescription medications, vitamins, and herbal supplements, from 60 days before the planned surgery and until Week 40 or early discontinuation will be recorded. All over-the-counter or prescription medications, vitamins, and/or herbal supplements will be recorded in the CRF with their indications.
