# Supplementary material for: Randomized trial of intermittent intraputamenal glial cell line-derived neurotrophic factor in Parkinson’s disease
Source: Brain. 2019 Feb 26;142(3):512–25. doi: 10.1093/brain/awz023 (PMC6391602; doi:10.1093/brain/awz023)
Supplement: Supplementary Data [file awz023_supp.zip › awz023-suppl_data/awz023_Supplementary_Data_S3.pdf]

Summary of Protocol Amendments

A Placebo-Controlled, Randomised, Double-Blind Trial to Assess the Safety and Efficacy of Intermittent Bilateral Intraputamenal Glial Cell Line-Derived Neurotrophic Factor (GDNF) Infusions Administered via Convection Enhanced Delivery (CED) in Subjects with Parkinson's Disease

Internal Reference No: 2553

Ethics Ref: 12/SW/0047

EudraCT Number: 2011-003866-34

The original protocol (version 1.0) was dated 18 Apr 2012. There were 8 protocol amendments (7 major, 1 minor). A summary of the most important changes introduced by each amendment is given below.

1.1.1.1 Amendment 1, dated 31 May 2012

At the request of the MHRA, an additional inclusion criterion was included, giving contraception advice for male subjects with female partners of childbearing potential.

1.1.1.2 Amendment 2, dated 17 Oct 2012

Prior to the start of the study, the up-titration portion of the intraputamenal infusion regimen was modified from a stepped up-titration scheme over 20 minutes to a linear ramping scheme over 30 to 40 minutes. In addition, the programmable syringe pump to be used for the infusions was specified.

1.1.1.3 Amendment 3, dated 25 Apr 2013

This amendment introduced a switch from 2-weekly infusion intervals to 4-weekly infusion intervals and the possibility to slow the rate of individual infusions from 5 µL/min to 3-5 µL/min in response to post-infusion MRI findings. The changes were made in an effort to minimize the probability and extent of reflux during the infusions. In order to compensate for the lower frequency of infusions and keep the original total dose delivered per 4-week period (240 µg) constant, the concentration of GDNF in the infusate was increased from 0.1 µg/µL to 0.2 µg/µL. Since the frequency of study drug administration was changed from once every 2 weeks to once every 4 weeks by this amendment, the number of treatments was reduced from 20 to 10, occurring at Weeks 0, 4, 8, 12, 16, 20, 24, 28, 32, and 36, while the treatment visits at Weeks 2, 6, 10, 14, 18, 22, 26, 30, 34, and 38 were cancelled. The last treatment was to be given at Week 36 instead of Week 38. The post-infusion MRIs originally to be performed in Pilot Stage subjects at the cancelled visits were rescheduled to be performed at the remaining visits. Amendment 3 was implemented during the Pilot Stage, before the start of the Primary Stage.

In order to address potential safety concerns associated with the occurrence of reflux, the Sponsor decided to switch to 4-weekly infusion intervals and lower infusion rates before acceptance of the amendment by the MHRA and ethics committee, but without the compensatory increase in the concentration of GDNF in the infusate. In the time between submission of the amendment to the MHRA on 29 Apr 2013 (submission to ethics committee on 01 May 2013) and the implementation of the amendment on 19 Jun 2013 after acceptance by the MHRA and ethics committee, treatments were continued at the 0.1 µg/µL concentration level while every second infusion was effectively omitted. The interim switch was documented as a protocol deviation in all Pilot Stage subjects.

1.1.1.4 Amendment 4, dated 26 Jun 2013

This amendment introduced a number of mostly minor changes and clarifications to optimize the design and procedures for the Primary Stage on the basis of the consolidated experience gathered during the Pilot Stage. The most important changes were:

- **BDI score (exclusion criterion):** Definition of significant depression as a BDI score changed from ≥ 14 to ≥ 20. Mild depression, as defined by a BDI score of 14 to 19, is common among subjects with PD and does not correlate with increased suicidality. Also, the BDI includes questions about somatic symptoms which are common in physical complaints of subjects with PD, even in the absence of depression, and therefore the score is likely to overestimate the amount of depression in these subjects.
- **Required coverage of a predefined VOI in each putamen:** Threshold for the required coverage of the predefined VOI in each putamen lowered from 50% to 40% and independent MRI review replaced by confirmation by the principal investigator. The rationale for lowering the threshold for putamenal VOI coverage was that T2- and diffusion-weighted MRI, even when done real-time, underestimates the true volume of distribution during CED over a wide range of molecular sizes by approximately 50% [1]. In the current study, the volume of distribution was determined within 2 hours of the end of infusion which amplifies the underestimating effect, as the MRI signal weakens over time. Therefore, it could be safely assumed that a measured VOI coverage of 40% corresponds to a true coverage of at least 80% which was unprecedented clinically and considered highly sufficient.
- **Duration of healing phase and interval before start of double-blind treatment:** Both periods increased to 4 weeks.
- **MRI scanning:** The amendment clarified that post-infusion MRI scans were to be completed (instead of obtained) within 2 hours of the end of infusion, and increased the post-infusion MRI monitoring frequency for subjects randomized during the Primary Stage to include additional scans at Weeks 0, 4, 8, 12, 20, and 36 in the first 6 subjects and at Weeks 4, 20, and 36 in the remaining 30 subjects. The amended protocol also allowed for additional scans to be performed at the discretion of the principal investigator.
- **Additional safety assessments:** The amendment added clinical assessment of safety, including dyskinesias and falls, mood, and impulsivity at each infusion visit, repetitions of the QUIP every 8 weeks during the study, and several brief measures of cognitive and executive function (NART, FrSBe, RT, UPPS-P, verbal fluency). A pregnancy test was added at Weeks 4, 16, 28, and 40 for all women of childbearing potential.
- **Monitoring interval for vital sign measurements during and after drug administration at postbaseline treatment visits:** The monitoring interval for vital signs at postbaseline treatment visits (Weeks 4, 8, 12, 16, 20, 24, 28, 32, and 36) was increased from 15 to 30 minutes during drug administration and from 15 to approximately 30 minutes after the end of infusion. Vital signs had been found to be very stable during and after study drug administration in Pilot Stage subjects, with no adverse trend being observed for any measure at any treatment.

1.1.1.5 Amendment 5, dated 02 Jun 2014

The most important change introduced by this amendment was the addition of contrast-enhanced T1-weighted MRI following the test infusion at the end of the healing phase in Primary Stage subjects for the determination of the initial VOI coverage in addition to the standard T2-weighted and FLAIR MRI. Other changes included clarification of handling of subjects who were delayed in receiving surgery and/or in having their baseline (Week 0) visit and removal of the Cattell Culture Fair Intelligence Test as an outcome measure.

Although amendment 4 lowered the required minimum coverage of putamenal VOI from 50% to 40%, assuming that this would correspond to a true coverage of at least 80%, VOI coverage following test infusions in the Pilot Stage cohort ranged from 50.8% to 79.4%. In order to further improve the distribution results, catheters were positioned slightly more ventrally and more anteriorly in the first several Primary Stage subjects so that more infusate would be confined within the putamen even if there was modest reflux. It was therefore surprising to find that 4 of the new subjects showed unsatisfactory distribution results and did not reach the 40% threshold for VOI coverage. Potential explanations considered for this observation included mechanical failure (e.g. catheter blockage or leakage), reflux, overflow across putamen boundaries, drainage in the perivascular space, and lack of extension of the extracellular space that would prevent the infusate from being visualized although the distribution may in fact be excellent.

Except for catheter blockage, none of these potential reasons could be excluded without the use of a contrast agent allowing for direct visualization of the infusate, as T2-weighted and FLAIR MRI only visualizes infusion-related expansions of the extracellular space. When the test infusions for the 4 affected subjects were repeated with the addition of a gadolinium contrast agent (gadopentetate dimeglumine), VOI coverage as assessed by contrast-enhanced T1-weighted MRI was shown to range from 44% to 74% in all 4 subjects, making all 4 randomizable. These findings suggested that in humans and under the conditions of this study, T2-weighted and FLAIR MRI were underestimating the true volume of distribution more significantly than anticipated on the basis of nonhuman primate data [1].

Based on the insight provided by the contrast-enhanced T1-weighted MRI, the protocol was amended to include the use of gadolinium contrast with all future test infusions and to allow determining the initial VOI coverage on the basis of contrast-enhanced T1-weighted MRI in addition to the standard T2-weighted and FLAIR MRI. The use of the contrast agent was limited to test infusions of diluent (which were separated from infusions of study medication by 4 weeks), and the infusion protocol included an aCSF “flush” to clear the dead space of the implanted system after each infusion. Therefore, there was effectively no potential for drug interactions between the contrast agent and GDNF.

1.1.1.6 Amendment 5.1, dated 23 Jun 2014

The amendment changed the addresses of the principal investigator and study neurosurgeon to reflect their new location following the move from Frenchay Hospital to new facilities in Southmead Hospital.

1.1.1.7 Amendment 6, dated 17 Sep 2014

Following on from MRI changes already implemented by amendments 4 and 5, this amendment simplified the MRI schedule and deleted the previously planned functional MRI substudy, while adding another contrast-enhanced T1-weighted test infusion of diluent at Week 40.

1.1.1.8 Amendment 7, dated 28 Oct 2015

This amendment provided a number of clarifications and refinements that were considered mostly administrative in nature or evolved during the preparation of the SAP. The most important changes were:

- **Protocol section 5.2 (Study Endpoints):** During the preparation of the SAP, it was recognized that the wording of some secondary and supplementary efficacy endpoints, imaging endpoints, and safety endpoints was less clear than anticipated. In addition, a number of endpoints were added in order to provide for a more comprehensive analysis of the study data. Therefore, the wording of the respective protocol sections was refined. The revised wording for endpoints is used in the respective sections of this study report.
- **Protocol section 5.6.2 (Postbaseline Treatments):** In order to avoid infusion intervals of <3 weeks, the protocol text was modified to clarify that, if necessary, in the Primary Stage, a treatment could be given a maximum of + 7 days from the scheduled date; if the treatment could not be given within + 7 days, it was to be considered missed and the treatments resumed with the following scheduled date.
- **Protocol section 5.7.1.1 (Unified Parkinson's Disease Rating Scale):** Text revised to clarify the statement that all post-randomization UPDRS ratings would be performed by a trained rater who was blinded to all other aspects of the subject's condition. Since the Week 0 assessments were performed prior to first dose of study medication, "post-randomization" was to be understood as "postbaseline" (i.e. after Week 0) in this case.
- **Protocol section 5.8 (PET Scanning Procedures and Image Analysis):** Text revised to indicate that the analysis would follow established procedures as described by Nandhagopal et al. [2], with co-registration of MRI images as a guidance on where to place the ROIs, rather than just visual inspection. The specification of the proposed statistical analysis was deleted since the analysis would be defined in detail in the SAP.
- **Protocol section 8.1 (Description of Statistical Methods):** The ANCOVA at 9 months originally planned as the primary analysis was replaced by a mixed-effect model with repeated measures (MMRM) to compare the OFF state UPDRS motor score (part III) curves over time between the treatment groups. This change was made to allow comparison of the response curves over time rather than limit the primary comparison to the 9-month time point. The ANCOVA was included as part of the sensitivity analyses. A similar approach was taken in the analyses of the secondary and supplementary efficacy endpoints.
- **Protocol section 8.7 (Inclusion in Analysis):** During preparation of the statistical analysis plan, it was determined that the definition of safety populations should follow the logic of the ITT populations. Hence, the complete set of safety analyses was to be performed for the Safety Primary Population comprising all Primary Stage subjects who received at least one study drug (grouped by treatment actually received). More limited safety analyses were to be

performed for the Safety Overall Population comprising all subjects (both Pilot Stage and Primary Stage) who received at least one study drug (grouped by treatment actually received). Safety data for Pilot Stage subjects was not to be tabulated separately, but instead would be evaluated via individual data listings. In addition, a new Safety Enrolled Population was added to provide for the analysis of pre-treatment AEs. This population included all enrolled subjects (Pilot and Primary Stages combined).

- **AE documentation:** The protocol was revised to eliminate inconsistencies with respect to the documentation of AEs at study start. All AEs, both nonserious and serious, were recorded on the AE CRF pages from the time of screening (first consent) until the Week 40 visit was completed (or 28 days after the last dose of study medication for subjects who discontinued the study early). In addition, the text of protocol section 7.8 was revised to correctly reflect the information documented in the CRF for each AE.

References

1. Iyer RR, Butman JA, Walbridge S, Gai ND, Heiss JD, Lonser RR. Tracking accuracy of T2- and diffusion-weighted magnetic resonance imaging for infusate distribution by convection-enhanced delivery. J Neurosurg. 2011;115(3):474-80.
2. Nandhagopal R, Kuramoto L, Schulzer M, Mak E, Cragg J, Lee CS, McKenzie J, McCormick S, Samii A, Troiano A, Ruth TJ, Sossi V, de la Fuente-Fernandez R, Calne DB, Stoessl AJ. Longitudinal progression of sporadic Parkinson’s disease: a multi-tracer positron emission tomography study. Brain. 2009;132(Pt 11):2970-9.
