# Supplementary material for: Randomized trial of intermittent intraputamenal glial cell line-derived neurotrophic factor in Parkinson’s disease
Source: Brain. 2019 Feb 26;142(3):512–25. doi: 10.1093/brain/awz023 (PMC6391602; doi:10.1093/brain/awz023)
Supplement: Supplementary Data [file awz023_supp.zip › awz023-suppl_data/awz023_Supplementary_Data_S5.pdf]

Statistical Analysis Plan

**Sponsor:**	North Bristol NHS Trust (NBT)
**Client:**	MedGenesis Therapeutix
**Protocol No./EudraCT No.:**	GDNF 2553 / 2011-003866-34
**Version No./Date**	1.7 (Amendment 7) / 28-Oct-2015
**Title**	Protocol Title A Placebo-Controlled, Randomized, Double-Blind Trial to Assess the Safety and Efficacy of Intermittent Bilateral Intraputamenal Glial Cell Line-Derived Neurotrophic Factor (GDNF) Infusions Administered via Convection-Enhanced Delivery (CED) in Subjects with Parkinson's Disease
**PRA Project Id:**	MDGGNDNFD-GDNFDM
**SAP Version No./Date:**	Version 2.0 / 16-Jun-2016

Approvals

Principal Investigator (PI)

PI Affiliation: Department of Neurology and the Burden Institute
Movement Disorder Service,
Brain Centre,
Southmead Hospital, North Bristol NHS Trust
Bristol BS10 5NB, United Kingdom

PI Name, Title: Alan Whone, FRCP, PhD, Consultant Senior Lecturer and Hon Consultant Neurologist

Signature, Date: A Whone 17/June 2016.

Sponsor

Sponsor Name: North Bristol NHS Trust (NBT)

Representative, Title: Rebecca Smith, PhD, Deputy Director of Research

Signature, Date: 16 – June – 2016

Client

Client Name: MedGenesis Therapeutix

Representative, Title: Lara Longpre, Chief Operating Officer

Signature, Date: 16-June-2016

Representative, Title: Matthias Luz, MD, Chief Medical Officer

Signature, Date: 16 – June – 2016

PRA

Project Manager, Title: Diana Soto, Project Manager

Signature, Date: Diana Soto 21-JUN-2016

Biostatistician, Title: Emma Lewis, Senior Biostatistician

Signature, Date: E Lewis 20 JUN 2016

Table of Contents

Approvals ... 2
Table of Contents ... 3
1.0 Introduction ... 5
1.1 Changes from Protocol ... 5
2.0 Study Objectives ... 5
2.1 Primary Objective ... 5
2.2 Secondary Objectives ... 5
2.3 Other Objectives ... 5
3.0 Study Design ... 5
3.1 Sample Size Considerations ... 12
3.2 Randomization ... 12
4.0 Study Endpoints and Covariates ... 12
4.1 Efficacy Endpoints ... 12
4.1.1 Primary Efficacy Endpoint ... 12
4.1.2 Secondary Efficacy Endpoints ... 12
4.1.3 Supplementary Efficacy Endpoints ... 13
4.2 Imaging Endpoints ... 13
4.3 Safety Endpoints ... 14
4.4 Predetermined Covariates and Prognostic Factors ... 15
5.0 Definitions ... 15
6.0 Analysis Populations ... 22
6.1 Intent-to-Treat Populations ... 22
6.1.1 ITT Primary Population ... 22
6.1.2 ITT Overall Population ... 22
6.1.3 ITT Pilot Population ... 23
6.2 Per-Protocol Population ... 23
6.3 Safety Populations ... 23
6.3.1 Safety Primary Population ... 23
6.3.2 Safety Overall Population ... 23
6.3.3 Safety Pilot Population ... 23
6.3.4 Safety Enrolled Population ... 23
7.0 Interim Analyses ... 23
8.0 Data Review ... 24
8.1 Data Handling and Transfer ... 24
8.2 Data Screening ... 24
9.0 Statistical Methods ... 24
9.1 Missing Data Methods ... 25
9.1.1 Missing or Partial Dates ... 25
9.1.2 Missing Efficacy Data ... 25
9.1.3 Missing Data for Questionnaires ... 25
9.1.4 Missing Safety Data ... 25
9.1.5 Special Arrangements for Subject 45 ... 26
9.2 Subject Disposition ... 26
9.3 Protocol Deviations ... 26
9.4 Demographic and Baseline Characteristics ... 27

9.4.1 Demographic Characteristics ... 27
9.4.2 Parkinson's Disease History at Screening ... 27
9.4.3 General Medical History ... 28
9.5 Prior and Concomitant Medications ... 28
9.6 Surgery and Test Infusions ... 28
9.6.1 Catheter Trajectories and Positioning Accuracy ... 28
9.6.2 Contrast-Enhanced Test Infusions with T1-Weighted MRI Prior to Start of Study Medication ... 28
9.7 Efficacy Analyses ... 29
9.7.1 Analyses of Primary Efficacy Endpoint ... 29
9.7.2 Analyses of Secondary Efficacy Endpoints ... 31
9.7.3 Analyses of Supplementary Efficacy Endpoints ... 33
9.8 Imaging Analyses ... 36
9.8.1 MRI Analyses ... 36
9.8.2 PET Analyses ... 37
9.8.3 Correlation Analyses ... 37
9.9 Safety Analyses ... 38
9.9.1 Study Medication Exposure ... 38
9.9.2 Adverse Events ... 39
9.9.3 Port Symptoms ... 40
9.9.4 Adverse Changes in MRI Findings ... 40
9.9.5 Laboratory Data ... 40
9.9.6 Anti-GDNF Antibodies ... 41
9.9.7 Plasma GDNF Concentrations ... 41
9.9.8 Physical Examination ... 41
9.9.9 Vital Signs ... 41
9.9.10 Weight ... 42
9.9.11 Electrocardiogram ... 42
9.9.12 Glasgow Coma Scale ... 43
9.9.13 Questionnaire for Impulsive-Compulsive Disorders ... 43
9.9.14 Montreal Cognitive Assessment ... 43
9.9.15 Mattis Dementia Rating Scale ... 44
9.9.16 Stroop Test ... 44
9.9.17 Frontal Systems Behavioural Scale ... 44
9.9.18 Deary-Liewald Reaction Time ... 44
9.9.19 Verbal Fluency Assessment ... 45
9.9.20 Beck Depression Inventory ... 45
9.9.21 University of Pennsylvania Smell Identification Test ... 45
10.0 Validation ... 45
Appendix 1 Glossary of Abbreviations ... 46
Appendix 2 Protocol Deviation Guidance ... 48
Appendix 3 List of MedDRA Preferred Terms for Adverse Events of Special Interest ... 50
Appendix 4 List of Conversion Factors for the Calculation of Levodopa and Levodopa Equivalent Doses ... 51
Appendix 5 List of Post-Text Tables, Figures and Listings ... 52
Appendix 6 Shells for Post-Text Tables, Figures and Listings ... 61
Document History ... 201

1.0 Introduction

This statistical analysis plan (SAP) describes the statistical methods to be used during the reporting and analyses of data collected under North Bristol NHS Trust (NBT) Protocol 2553. Data collected in the separate extension study are not included in the scope of this document.

This SAP should be read in conjunction with the study protocol and case report form (CRF). This version of the plan has been developed using the protocol version 1.7 (Amendment 7) dated 28OCT2015 and the corresponding CRF. Any further changes to the protocol or CRF may necessitate updates to the SAP.

The SAP was developed in two stages. The purpose was to "finalize" a SAP so that PRA could start programming earlier in the process. Versions of the SAP up to initial approval were known as SAP1. Changes following approval of SAP1 were tracked in the SAP Change Log and a final version of the SAP, known as SAP2, was issued for approval prior to database lock.

1.1 Changes from Protocol

Not applicable.

2.0 Study Objectives

2.1 Primary Objective

- To assess the effect of 4-weekly intermittent bilateral intraputamenal GDNF infusions on OFF state motor function at 9 months.

2.2 Secondary Objectives

- To assess the effect of intermittent bilateral intraputamenal GDNF infusions on ON state motor function, motor complications, and ON and OFF state activities of daily living (ADL) at 9 months.
- To assess the safety of intermittent bilateral intraputamenal GDNF infusions in a small pilot cohort of subjects and in the full study population.

2.3 Other Objectives

- To explore the effects of intermittent bilateral intraputamenal GDNF infusions on other motor and non-motor functions, quality of life (QOL) assessments, and imaging endpoints at 9 months.

3.0 Study Design

This is a phase II, single-center, randomized, double-blind, placebo-controlled trial of intermittent bilateral intraputamenal GDNF infusions administered via convection-enhanced delivery (CED) in subjects with idiopathic Parkinson's disease (PD) with motor fluctuations. The study consists of 2 distinct stages: a Pilot Stage (N=6) and a Primary Study Stage (N=36). The Pilot Stage serves to ensure the safety of the surgical technique and study drug administration and to optimize, if necessary, the planned study procedures. The Primary Study Stage serves to provide the main data for the evaluation of the study objectives and endpoints. Figure 1 presents the study schema.

After a screening period of up to 60 days, eligible subjects undergo surgery under general anesthesia for implantation of a customized in-house system for CED comprising 2 indwelling catheters per putamen and a skull-mounted transcutaneous drug delivery port. After healing and if the subject continues to meet the entry criteria, an intraputamenal test infusion of artificial cerebrospinal fluid (aCSF, diluent) is administered, and magnetic resonance imaging (MRI) is performed to assess catheter patency and to determine infusate distribution as part of the post-surgery eligibility assessment. Subjects found to have adequate catheter function and infusate distribution in putamen are eligible for randomization.

In the Pilot Stage, eligible subjects are randomized in a 2:1 allocation of GDNF:placebo. In the Primary Stage, eligible subjects are randomized in a 1:1 allocation of GDNF:placebo. In both stages, randomized subjects are treated with study medication (GDNF or placebo) for 9 months in a double-blind fashion. Treatment consists of 600 µL (containing 120 µg GDNF at 0.20 µg/µL or placebo [60 µg GDNF at 0.10 µg/µL or placebo prior to Amendment 3]) infusions per putamen every 4 weeks [every 2 weeks prior to Amendment 3] for 9 months, for a total of 10 bilateral infusions delivering 240 µg GDNF or placebo each, with the last infusion at Week 36 (20 bilateral infusions delivering 120 µg GDNF or placebo each, with the last infusion at Week 38 prior to Amendment 3). The primary analysis of this study is an intention-to-treat (ITT) analysis of the 36 subjects randomized in the Primary Stage that is performed at Week 40, when these subjects complete (or had the opportunity to complete) the 9-month double-blind treatment period.

Key clinical outcomes are measured at 8-week intervals throughout the study. Additional clinical outcome measures are assessed at baseline and at Week 40. Table 1 and Table 2 depict the Schedule of Events for the study. All subjects randomized in the Pilot and Primary Study Stages and completing double-blind treatment will be offered the opportunity to enroll in an open-label active treatment extension study under a separate protocol (no. 2797).

An independent Data Monitoring Committee is charged with safeguarding the interests of the study subjects. In addition, the United Kingdom Medicines and Healthcare Products Regulatory Agency (MHRA) reviews safety data after the last Pilot Stage subject completes 3 months of treatment and before enrollment in the Primary Stage commences.

Figure 1. Study Schema

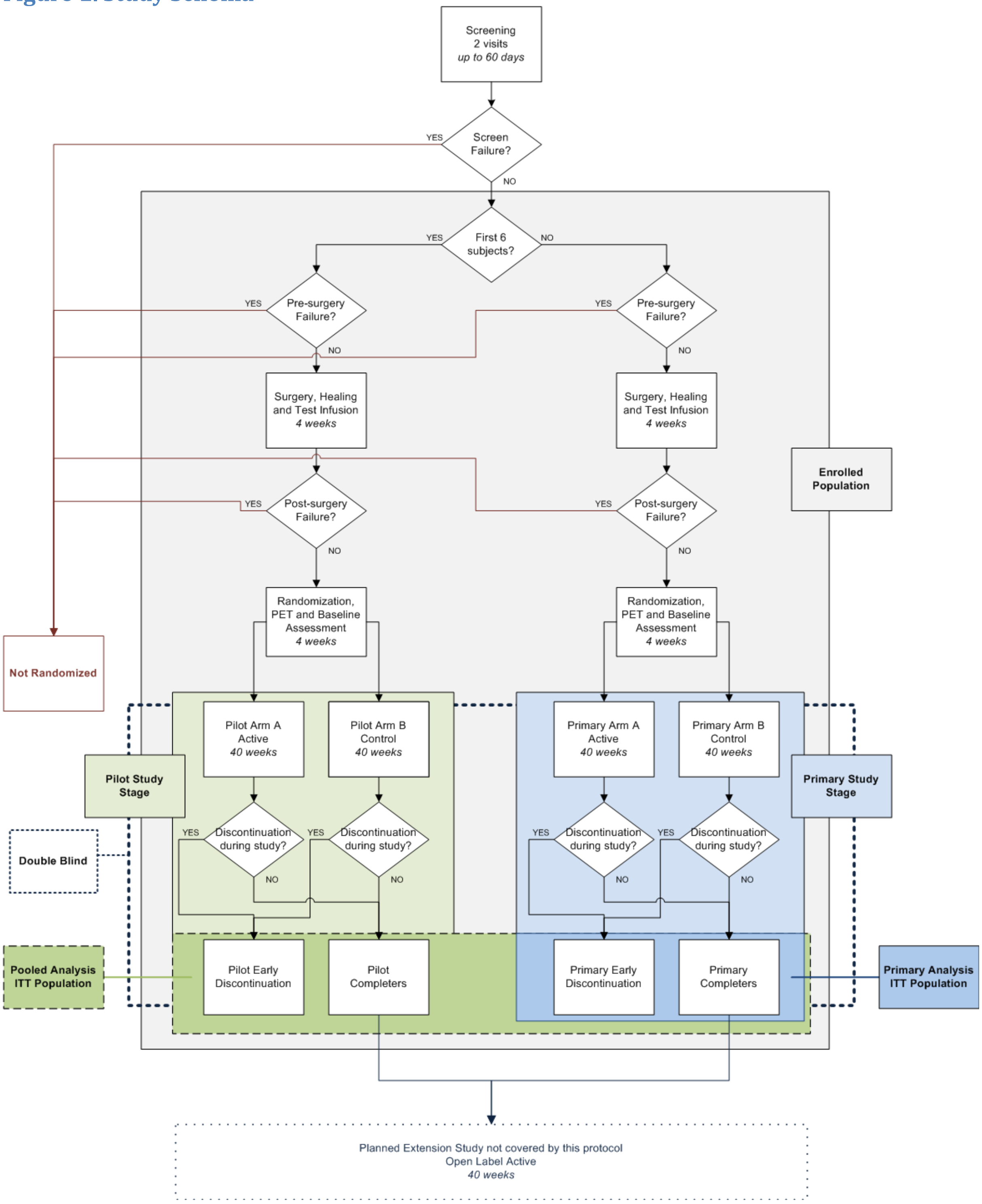

Note: Amendment 4 increased the duration of both the healing phase and the interval between test infusion and first infusion of study drug to 4 weeks, increasing the overall duration of the combined surgery and healing phase to 8 weeks, to harmonize with the 4-weekly treatment schedule introduced in Amendment 3. Both amendments were implemented before the start of the Primary Study Stage.

Table 1. Schedule of Events (Presurgery; Surgery and Healing)

Procedure/Assessment	Presurgery and screening phase		Surgery and healing phase [C]
	Screening Visit 1 [A]	Screening Visit 2 [A,B]	
Informed consent [D]	X		X [E]
Demography	X		
Medical history, including PD history	X		
Hoehn and Yahr staging	X		
Physical examination [F]	X		
Laboratory tests [G]	X		
Anti-GDNF antibodies and GDNF plasma concentrations	X		
Vital signs, weight, height [H]	X		X
ECG	X		
MoCA	X		X [J]
MDRS	X		
Verbal fluency assessment		X	
BDI		X	
Stroop test, NART, FrSBe, RT, UPPS-P		X	
QUIP		X	
MRI		X [I]	X [J]
UPDRS part II and part III in OFF state		X	
Timed walking test in OFF state		X	
Timed tapping test in OFF state		X	
Levodopa challenge		X	
UPDRS in ON state		X	
Timed walking test in ON state		X	
Timed tapping test in ON state		X	
PD fluctuation diary training	X		
Dispense PD fluctuation diaries	X		X
Collect PD fluctuation diaries		X	
NMSS		X	
PDQ-39 and EQ-5D		X	
SNAQ		X	
UPSIT		X	
Prior medications [K]	X	X	X
Catheter placement			X
Post-operative CT scan			X
Test infusion of aCSF			X [J]
Glasgow Coma Scale [L]			X
Adverse events [M]	X	X	X

[A] Assessments did not have to be completed in a single visit, provided that all assessments were done within 60 days of surgery.

[B] No PD medications were to be taken after 6:00 PM on the night before the assessments and no long-acting PD medications were to be taken on the day before the assessments. Subjects were to refrain from eating any high-protein foods on the morning of the assessments.

[C] All surgical procedures were carried out according to institutional practices and under the direction of the study neurosurgeon. Subjects remained hospitalized post-surgery for a period of time determined by the study neurosurgeon or designee and based on the individual subject's course of recovery.

[D] Informed consent was to be obtained before any study-specific procedures or assessments were performed.

[E] Following completion of screening procedures, subjects were to be reconsented prior to undergoing any surgical procedures.

[F] Full physical examination, including assessments of body systems. Findings on screening physical examination were to be recorded as medical history.

[G] Hematology, serum chemistry, urinalysis, pregnancy test. For individual tests, see protocol.

[H] For times of assessment of vital signs, see protocol.

[I] T2-weighted and FLAIR MRI; for surgical planning and baseline scan for safety prior to surgery and to rule out MRI evidenced alternative cause for PD.

[J] For subjects who completed the healing period, an infusion of aCSF was to be administered and T2-weighted and FLAIR MRI scans completed within 2 hours following the infusion. Pilot Stage subjects were to receive the aCSF infusion in the MRI suite to allow for additional real-time MRI. In Primary Study Stage subjects, the aCSF infusion was to contain gadolinium contrast and a T1-weighted MRI scan was also to be obtained. A second aCSF infusion and MRI was to be performed if infusion parameters changed. MoCA assessment was to be performed on the same day as the test infusion.

[K] All over-the-counter or prescription medications, vitamins, and herbal supplements taken from 60 days before the planned surgery until Week 40 or early discontinuation were to be recorded in the CRF, together with the indications for administration.

[L] Performed before infusion, 30 minutes into the infusion, and after completion of the infusion.

[M] AE documentation period: From the time of screening (first consent) until the Week 40 visit had been completed (or 28 days after the last dose of study medication for subjects who discontinued the study early).

Abbreviations used in the table

aCSF: Artificial cerebrospinal fluid; AE: Adverse event; BDI: Beck Depression Inventory; CRF: Case report form; CT: Computed tomography; ECG: Electrocardiogram; EQ-5D: EuroQOL 5-Dimensional Scale; FLAIR: Fluid-attenuated inversion recovery; FrSBe: Frontal Systems Behavioural Scale; GDNF: Glial cell line-derived neurotrophic factor; MDRS: Mattis Dementia Rating Scale; MoCA: Montreal Cognitive Assessment; MRI: Magnetic resonance imaging; NART: National Adult Reading Test; NMSS: Non-Motor Symptom Scale; PD: Parkinson's disease; PDQ-39: Parkinson's Disease Questionnaire-39; QUIP: Questionnaire for Impulsive-Compulsive Disorders in Parkinson's Disease; RT: Deary-Liewald Reaction Time; SNAQ: Simplified Nutritional Appetite Questionnaire; UPDRS: Unified Parkinson's Disease Rating Scale; UPPS-P: UPPS-P Impulsiveness Behaviour Scale; UPSIT: University of Pennsylvania Smell Identification Test.

Table 2. Schedule of Events (Double-Blind Treatment)

**Procedure/Assessment**	**Week**										
	**0** [C]	**4**	**8** [C]	**12**	**16** [C]	**20**	**24** [C]	**28**	**32** [C]	**36**	**40** [C]
MRI (Pilot subjects) [A]	X	X	X	X	X	X	X	X	X	X	
MRI (Primary Study Stage subjects) [B]											X
PET	X [D]			X [E]							X [F]
Vital signs [G]	X	X	X	X	X	X	X	X	X	X	X
Weight and height	X		X		X		X		X		X
Physical examination [H]											X
ECG											X
Laboratory tests [I]		X			X			X			X
Anti-GDNF antibodies and GDNF plasma concentrations		X			X			X			X
UPDRS part II and part III in OFF state	X		X		X		X		X		X
Timed walking test in OFF state	X		X		X		X		X		X
Timed tapping test in OFF state	X		X		X		X		X		X
Levodopa challenge	X		X		X		X		X		X
UPDRS in ON state	X		X		X		X		X		X
Timed walking test in ON state	X		X		X		X		X		X
Timed tapping test in ON state	X		X		X		X		X		X
RT				X			X				X
NMSS				X			X				X
PDQ-39 and EQ-5D											X
MoCA and MDRS											X
Stroop test, FrSBe, verbal fluency assessment											X
SNAQ											X
UPSIT											X
BDI											X
QUIP	X		X		X		X		X		X
Collect PD fluctuation diaries	X		X		X		X		X		X
Dispense PD fluctuation diaries		X		X		X		X		X	
Randomization	X										
Infusion of study drug	X	X	X	X	X	X	X	X	X	X	
Glasgow Coma Scale [J]	X	X	X	X	X	X	X	X	X	X	X
Adverse events [K]	X	X	X	X	X	X	X	X	X	X	X
Concomitant medications [L]	X	X	X	X	X	X	X	X	X	X	X

[A] T2-weighted and FLAIR MRI were to be completed within 2 hours of study drug infusion in Pilot Stage subjects at all time points. Also, Pilot Stage subjects were to receive the Week 0 infusion in the MRI suite to allow for additional real-time MRI.

[B] T1-weighted, T2-weighted, and FLAIR MRI scans were to be completed within 2 hours of a gadolinium contrast-containing test infusion of diluent at Week 40 (±1 week). No interim T2-weighted or FLAIR MRI monitoring was to be done in Primary Study Stage subjects unless clinically mandated.

[C] At Weeks 0, 8, 16, 24, 32, and 40, no PD medications were to be taken after 6:00 PM on the night before the assessments, and no long-acting PD medications were to be taken on the day before the assessments. Subjects were to refrain from eating any high-protein foods on the morning of the assessments.

[D] The Week 0 PET could be performed at any time after randomization and before the first infusion with study drug.

[E] Subjects randomized during the Pilot Stage only.

[F] The Week 40 PET could be performed at any time in the 2 weeks prior to Week 40.

[G] For times of assessment of vital signs, see protocol.

[H] Brief physical examination, targeted, at the investigator’s discretion, to identify changes from Screening Visit 1.

[I] Hematology, serum chemistry, urinalysis, pregnancy test. For individual tests, see protocol.

[J] Performed before infusion, 30 minutes into the infusion, and after completion of the infusion.

[K] AE documentation period: From the time of screening (first consent) until the Week 40 visit had been completed (or 28 days after the last dose of study medication for subjects who discontinued the study early).

[L] All over-the-counter or prescription medications, vitamins, and herbal supplements taken from 60 days before the planned surgery until Week 40 or early discontinuation were to be recorded in the CRF, together with the indications for administration.

Abbreviations used in the table

AE: Adverse event; BDI: Beck Depression Inventory; CRF: Case report form; ECG: Electrocardiogram; EQ-5D: EuroQOL 5-Dimensional Scale; FLAIR: Fluid-attenuated inversion recovery; FrSBe: Frontal Systems Behavioural Scale; GDNF: Glial cell line-derived neurotrophic factor; MDRS: Mattis Dementia Rating Scale; MoCA: Montreal Cognitive Assessment; MRI: Magnetic resonance imaging; NMSS: Non-Motor Symptom Scale; PD: Parkinson’s disease; PDQ-39: Parkinson’s Disease Questionnaire-39; PET: Positron emission tomography; QUIP: Questionnaire for Impulsive-Compulsive Disorders in Parkinson’s Disease; RT: Deary-Liewald Reaction Time; SNAQ: Simplified Nutritional Appetite Questionnaire; UPDRS: Unified Parkinson’s Disease Rating Scale; UPSIT: University of Pennsylvania Smell Identification Test.

3.1 Sample Size Considerations

The endpoint used as the basis for the sample size calculation of the Primary Stage is the percentage change from baseline to Week 40 in motor UPDRS (part III) in the practically defined OFF state. Assuming a standard deviation (SD) of 20%, a 2-sided type I error of 5%, a power of 80%, and a difference of 20 points in percentage change in motor UPDRS from baseline (eg, a response in the active group of 25% and a placebo response of 5% or a response in the active group of 35% and a placebo response of 15%), a total of 34 evaluable subjects, randomized in a 1:1 allocation of GDNF: placebo, are required to complete the study. A total of 36 subjects are to be randomized in the Primary Stage to compensate for a potential 5% loss of subjects for the evaluation of the primary endpoint at Week 40 (with last observations at earlier time points carried forward to Week 40).

3.2 Randomization

Subjects receive either GDNF or placebo infusions every 4 weeks for 36 weeks (every 2 weeks for 38 weeks prior to Amendment 3):

- Group A, active treatment: 600 μL of 0.20 μg/μL GDNF (0.10 μg/mL prior to Amendment 3) in aCSF per putamen;
- Group B, placebo: 600 μL of aCSF per putamen.

Study treatments are prepared by an unblinded pharmacist at the study site based on the unstratified, blocked randomization schedule prepared by the Bristol Randomized Trials Collaboration Clinical Trials Unit. Subjects were randomized in blocks of 6. Ready to use preparations of GDNF or aCSF are provided to the investigator or designee in identical containers identifiable only by codes unknown to the investigator or any other study staff.

Administration records are kept on site, and administration information, including date and time of infusion, identification of the infusate (blinded), infusion rate and duration, and the reasons for any changes to the infusion or for any missed or omitted infusions are recorded in the CRF.

4.0 Study Endpoints and Covariates

4.1 Efficacy Endpoints

4.1.1 Primary Efficacy Endpoint

The primary efficacy endpoint of the study is:

- Percentage change from baseline in the practically defined OFF state UPDRS motor score (part III) after 9 months of double-blind treatment.

The primary efficacy endpoint is evaluated for randomized Primary Stage subjects (ITT Primary Population; see Section 6.1.1).

4.1.2 Secondary Efficacy Endpoints

Secondary efficacy endpoints include the following:

- Percentage change from baseline in UPDRS motor score (part III) in the ON state (following a levodopa challenge) after 9 months of double-blind treatment.
- Percentage change from baseline in UPDRS ADL score (part II) in the OFF state and in the ON state after 9 months of double-blind treatment.

- Percentage change from baseline in UPDRS total score (sum of motor + ADL scores) in the OFF state and in the ON state after 9 months of double-blind treatment. Note: UPDRS parts I and IV are not included in the UPDRS total score as they are collected only during the ON state and therefore, appropriate and meaningful analysis of total score for both states (OFF and ON) is not possible if the values for these parts are included. In addition, because these UPDRS parts mix items that are side-effects of PD treatment with signs and symptoms of the underlying disease, inclusion of these values in the calculation of percentage change in UPDRS total score would blunt the response observed.
- Percentage change from baseline in UPDRS mentation, behavior, and mood score (part I) after 9 months of double-blind treatment.
- Percentage change from baseline in UPDRS complications of therapy score (part IV) after 9 months of double-blind treatment.
- Change from baseline in PD diary ratings after 9 months of double-blind treatment:
    - Total OFF time per day.
    - Total good-quality ON time per day (ON without dyskinesias or ON with non-troublesome dyskinesias).
    - ON time per day with troublesome dyskinesias.

Secondary efficacy endpoints are evaluated for the ITT Primary Population.

4.1.3 Supplementary Efficacy Endpoints

The following are supplementary efficacy endpoints:

- Primary and secondary endpoints in the ITT Overall Population (see Section 6.1.2) including subjects randomized in the Pilot Stage (ITT Pilot Population; see Section 6.1.3).
- Change from baseline in supplementary motor, non-motor, medication, and quality of life endpoints after 9 months of double-blind treatment, including the following:
    - Timed walking test (OFF and ON state).
    - Timed tapping test (OFF and ON state).
    - Non-Motor Symptom Assessment Scale for PD (NMSS).
    - Parkinson's Disease Questionnaire-39 (PDQ-39).
    - EuroQOL 5-Dimensional Scale (EQ-5D).
    - Simplified Nutritional Appetite Questionnaire (SNAQ).
    - Total daily dose of levodopa and total daily levodopa equivalent dose.

  These endpoints are evaluated for the ITT Primary Population.

4.2 Imaging Endpoints

The following are analyzed as imaging endpoints:

- Change from baseline in volume of distribution of infusate as determined by contrast-enhanced T1-weighted MRI after 9 months of double-blind treatment. This endpoint is evaluated for the ITT Primary Population.

- Change from baseline in volume of interest (VOI) coverage and total putamenal coverage as determined by contrast-enhanced T1-weighted MRI after 9 months of double-blind treatment. This endpoint is evaluated for the ITT Primary Population.
- Change from baseline in ^{18}F-DOPA uptake as determined by positron emission tomography (PET) scans after 9 months of double-blind treatment (ITT Primary Population, ITT Overall Population, ITT Pilot Population, and Per-Protocol Population) and after 3 months of double-blind treatment (ITT Pilot Population).
- Correlation between primary study endpoint and VOI coverage and total putamenal coverage at baseline as determined by contrast-enhanced T1-weighted MRI.
- Correlation between change from baseline to Week 40 in NMSS total score and total putamenal coverage at baseline as determined by contrast-enhanced T1-weighted MRI.
- Correlation between primary study endpoint and change from baseline to Week 40 in ^{18}F-DOPA uptake as determined by PET scan.
- Correlation between change from baseline to Week 40 in ^{18}F-DOPA uptake as determined by PET scan and VOI coverage and total putamenal coverage at baseline as determined by contrast-enhanced T1-weighted MRI.

All correlation endpoints are evaluated for the ITT Primary Population.

4.3 Safety Endpoints

The following are analyzed as safety endpoints:

- Frequency of treatment-emergent adverse events (TEAEs; all TEAEs and TEAEs related to study drug) during the study period.
- Frequency of device-related adverse events (AEs) during the study period.
- Frequency of dyskinesias, falls, adverse changes in mood, and impulsivity reported as TEAEs during the study period (AEs of special interest, AESIs).
- Adverse changes in MRI findings as captured by AE reporting.
- Results of routine laboratory blood tests (hematology, serum chemistry) and urinalysis performed at baseline and at intervals during the trial.
- Frequency of subjects with anti-GDNF antibodies during the study.
- Change from baseline in the Questionnaire for Impulsive-Compulsive Disorders in Parkinson’s Disease (QUIP) every 8 weeks.
- Change from baseline in the Montreal Cognitive Assessment (MoCA) after 9 months of double-blind treatment.
- Change from baseline in the Mattis Dementia Rating Scale (MDRS) after 9 months of double-blind treatment.

The following other safety data are also analyzed or listed:

- Exposure to study medication.
- Frequency of port symptoms.
- Pre-treatment AEs.
- Plasma GDNF concentrations.

- Physical examination.
- Vital signs.
- Weight and height.
- Electrocardiogram (ECG).
- Glasgow Coma Scale.
- Stroop test.
- Frontal Systems Behavioural Scale (FrSBe).
- Deary-Liewald reaction time (RT).
- Verbal fluency assessment.
- Beck Depression Inventory (BDI).
- University of Pennsylvania Smell Identification Test (UPSIT).

Safety endpoints and other safety data are analyzed for Primary Stage randomized and treated subjects (Safety Primary Population; see Section 6.3.1). Selected safety data are also analyzed for Pilot Stage and Primary Stage randomized and treated subjects combined (Safety Overall Population; see Section 6.3.2). Pre-treatment AEs are analyzed for the population of all enrolled subjects (Safety Enrolled Population; see Section 6.3.4).

4.4 Predetermined Covariates and Prognostic Factors

Subgroup analyses of the primary efficacy endpoint by the following factors may be undertaken:

- Age group at screening (< 65, ≥ 65 years).
- Sex (female, male).
- Race (white, nonwhite).
- UPDRS motor score (part III) in a practically defined OFF state at baseline (< 35, ≥ 35).
- Duration from first PD motor symptom (< 10 years, ≥ 10 years).
- Anti-GDNF antibodies (positive anytime postbaseline, negative postbaseline, missing).
- Surgical approach used for last catheter positioning prior to randomization (vertical trajectory, horizontal anterior-posterior trajectory, horizontal posterior-anterior trajectory).

5.0 Definitions

Adverse changes in MRI findings

Adverse changes in MRI findings as captured by AE reporting are defined as a Medical Dictionary for Regulatory Activities (MedDRA) preferred term of “Nuclear magnetic resonance imaging brain abnormal” (MedDRA higher level term “Central nervous system imaging procedures”).

Adverse events of special interest

TEAEs including dyskinesias, falls, adverse changes in mood, and impulsivity are considered AESIs in this study. AESIs are defined as follows.

Dyskinesias

Dyskinesia is defined as any of the following MedDRA preferred terms:

- Dyskinesia
- Chorea
- Ballism
- Athetosis
- Dystonia

Falls

A fall is defined as a MedDRA preferred term of "Fall."

Adverse changes in mood / Impulsivity

Prior to database lock, all adverse event data were reviewed by a board-certified neurologist and movement disorder specialist to identify any relevant MedDRA preferred terms for the categories "Adverse changes in mood" and "Impulsivity". The MedDRA preferred terms found are listed in Appendix 3.

Age

The following SAS® code will be used to calculate subject age (years):

Age = floor ((intck('month', birth date, IC date) - (day(IC date) < day(birth date))) / 12),

where intck is a SAS® function counting integer days, birth date is the database variable for date of birth, and informed consent date is the database variable for initial informed consent date.

Baseline, change from baseline, percentage change from baseline

Baseline values for comparisons with postbaseline values

In general for comparisons of postbaseline values to baseline values, the baseline value is defined as the last scheduled or unscheduled measurement prior to the first dose of randomized study medication. For selected assessments, the baseline value is collected at Week 0 (ie, post-surgery). These assessments include all parts of the UPDRS (OFF and ON state), timed tapping test and timed walking test (OFF and ON state), levodopa dose, levodopa equivalent dose, and QUIP. In order to reduce the burden of assessments at Week 0, most of the remaining baseline assessments are performed at the screening visits. Relevant exceptions are infusion performance measures (see below) and the baseline PET scan which can be done at any time between randomization and the first dose of randomized study medication.

Note that screening values of UPDRS motor scores (part III) are used instead of baseline for one sensitivity analysis of the primary efficacy endpoint. If multiple screening values are present for a given subject, then the last screening value is used.

Pre-infusion baseline values for comparison with values during or after infusion

For comparisons of values during or after infusion with pre-infusion values, the baseline is the pre-infusion value. This type of comparison applies to some vital sign assessments.

Screening UPDRS motor score (part III) before and after levodopa challenge

For comparisons of screening UPDRS motor scores (part III) for determination of levodopa responsivity, values before levodopa challenge (ie, OFF state) are the baseline and values after levodopa challenge (ie, ON state) are postbaseline.

Baseline values for measures of infusion performance

The baseline values of volume of distribution, VOI coverage, and putamenal coverage are those measured by post-infusion MRI following the test infusion administered at the end of the healing

period. Where multiple baseline measures were done, the last one (which determines eligibility for randomization) serves as the baseline. Note that left and right side are considered both separately and combined in some analyses.

Change from baseline

Change from baseline is defined as (postbaseline value – value at baseline).

Percentage change from baseline

Percentage change from baseline is defined as [(postbaseline value – value at baseline) / value at baseline] * 100%.

Body mass index

Body mass index (BMI) is calculated as kg/m^2 where kg is weight in kilograms and m^2 is height in meters, squared.

Catheter positioning accuracy

There are 4 catheters per subject (2 catheters per putamen). Catheter positioning accuracy is assessed by measurement of the actual target versus the planned target in mm for the tip of each catheter. This parameter is not derived, but is located in the Post-Operative CT Scan CRF as "Distance between planned target and actual target (mm)" for catheters #1-4 for each subject.

Completion of study

A subject who completes the study is identified as such on the End of Study CRF in the database.

Concomitant medication

Concomitant medications (Parkinson's disease medications and other medications) are defined as any medications ongoing at the start of randomized study medication dosing or with a start date on or after the first randomized study medication dose date, but not after the last randomized study medication dose date. In the case of missing or partial dates, any medications that could have been ongoing at the start of randomized study medication dosing or could have started on or after the first randomized study medication dose date but not after the last randomized study medication dose date are assumed to be concomitant. Medications are defined as prior or concomitant, but not both (see also definition of prior medication in this section).

Double-blind treatment phase

The 9-month double-blind treatment phase is defined as Week 0 through Week 40, inclusive, as assigned by the visit names in the database.

Duration of infusion of study medication

Duration of infusion of study medication in minutes is calculated as (infusion end time – infusion start time + 1). Interruptions are not subtracted.

Duration since first Parkinson's disease symptom, duration since Parkinson's disease diagnosis

Duration since PD symptom onset and duration since PD diagnosis in years is calculated as (screening Visit 1 date – initial PD symptom/diagnosis date + 1)/365.25. If the day only of PD symptom/diagnosis date is missing, then the 1st day of the month is imputed; if the month only is missing or month and day are missing, then January or January 1st is imputed, respectively. PD symptom/diagnosis dates with a missing year are not included in the calculations.

Early termination of study

A subject who terminates the study early is identified as such on the End of Study CRF in the database; a primary reason for early termination is provided.

Enrolled subject

An enrolled subject is one with a record in the database who meets all of the inclusion/exclusion criteria pre-surgery or for whom a waiver of individual inclusion or exclusion criteria was granted while meeting all other criteria pre-surgery.

Levodopa challenge dose

The levodopa challenge dose is the dose of levodopa in mg that the subject receives when undergoing a levodopa challenge.

Levodopa dose, total daily levodopa dose

The levodopa dose at screening, baseline and Week 40 is the total daily levodopa dose in mg that the subject is on at the time of the visit.

The actual daily doses of the individual levodopa preparations taken are documented on the Levodopa and Levodopa Equivalent Medications CRF. Since the bioavailability of levodopa preparations differs, specific conversion factors must be used in order to characterize the subject's effective levodopa dose (see Appendix 4). Immediate release preparations taken without concomitant catechol-O-methyl transferase (COMT) inhibitors do not require conversion (conversion factor 1.0). The daily doses of immediate release preparations taken with COMT inhibitors and of controlled release preparations are multiplied by the corresponding conversion factors. The total daily levodopa dose is then calculated by adding together the converted daily doses of all individual levodopa-containing preparations. COMT inhibitor doses are not included in the calculation.

Levodopa equivalent dose, total daily levodopa equivalent dose

Subjects with PD generally take numerous medications to control their symptoms. In order to have a measure of their total medication intake, a levodopa equivalent dose is calculated. Each PD medication, as documented on the Levodopa and Levodopa Equivalent Medications CRF, is multiplied by a specific conversion factor indicating the drug's relative potency with respect to immediate release levodopa unaccompanied by COMT inhibitors (see Appendix 4 for a full list of conversion factors). The total daily levodopa equivalent dose is calculated by adding together the daily levodopa equivalent doses of all individual PD medications. COMT inhibitor doses are not included in the calculation.

Measures of infusion performance

Measures of infusion performance are determined by hemisphere on the basis of contrast-enhanced T1-weighted MRI.

Volume of distribution

Volume of distribution per hemisphere is documented as "Volume of distribution (mL), left" and "Volume of distribution (mL), right" in the Post-Infusion MRI CRF.

Total volume of putamen

Total volume of putamen is documented as "Total volume of putamen (mL), left" and "Total volume of putamen (mL), right" in the Post Randomization MRI Review CRF (source: Baseline and Planning MRI CRF).

Putamenal volume of distribution

Putamenal volume of distribution is documented as "Volume of distribution (mL), left putamen" and "Volume of distribution (mL), right putamen". The baseline value is located in the Post Randomization MRI Review CRF. The Week 40 value is located in the Post-Infusion MRI CRF, Week 40.

Total putamenal coverage

Total putamenal coverage is defined as (putamenal volume of distribution / total volume of putamen * 100%). This parameter is derived for each putamen.

Volume of interest

Volume of interest is documented as "Volume of interest (mL), left" and "Volume of interest (mL), right" in the Post Randomization MRI Review CRF (source: Baseline and Planning MRI CRF).

Volume of interest coverage (absolute)

Absolute VOI coverage is documented as "Volume of interest covered by infusate (mL), left" and "Volume of interest covered by infusate (mL), right". The baseline value is taken from the Post Randomization MRI Review CRF (source: Post-Infusion MRI CRF, Healing Phase). The Week 40 value is located in the Post-Infusion MRI CRF, Week 40.

Volume of interest coverage (relative)

Relative VOI coverage is defined as (volume of interest covered by infusate / volume of interest * 100%). This parameter is documented as "Volume of interest covered by infusate (%), left" and "Volume of interest covered by infusate (%), right". The baseline value is taken from the Post Randomization MRI Review CRF (source: Post-Infusion MRI CRF, Healing Phase). The Week 40 value is located in the Post-Infusion MRI CRF, Week 40.

Not randomized subject

A not randomized subject is an enrolled subject who fails to meet the post-surgery randomization criteria or withdraws consent after initial surgery and is not randomized. Subjects who undergo surgery but are not randomized are included in the Safety Enrolled Population and included in analyses of pre-treatment AEs.

Pre-treatment adverse event

Pre-treatment AEs are defined as any AEs starting on or after the date of informed consent but before the first randomized study medication dose date. For handling of AEs that fall on the same day as first randomized study medication, see definition of TEAEs in this section.

Prior medication

Prior medications are defined as any medications with start and stop dates prior to the first randomized study medication dose date. Medications are defined as prior or concomitant, but not both (see also definition of concomitant medication in this section).

Protocol deviations

Protocol deviations are recorded on the protocol deviation form. They are categorized for summarization, applying controlled terminology including inclusion criteria, exclusion criteria, post-surgery randomization criteria, randomization, study medication (including overdose), non-study medication, study schedule/visit window, outcome assessment, and other). They are also classified as major or minor, based on whether they potentially impact the outcome of the study. See Appendix 2 for further details. Prior to database lock, the database entries for protocol deviations will be reviewed by an adjudication team (including, at a minimum, the PI, the study statistician and the Chief Medical Officer of MedGenesis Therapeutix) for consistency of the categorizations and classifications. Final determination of the classifications (major or minor) will be made by the Study Sponsor in view of the recommendations made by the adjudication team. Subjects with any major deviation are excluded from the Per-Protocol Population. See Section 6.2 on Per-Protocol Population and Section 9.3 describing summaries of protocol deviations.

Randomized study medication

Randomized study medication is defined as GDNF or placebo.

Randomized subject

A randomized subject is one who receives a randomization number in the database.

Study day, last visit on study

If the assessment date is prior to the first randomized study medication dose date then the study day is calculated as (assessment date – first randomized study medication dose date); if the assessment date is on or after the first randomized study medication dose date then the study day is calculated as (assessment date – first randomized study medication dose date + 1). Per Clinical Data Interchange Standards Consortium (CDISC) Standard Data Tabulation Model (SDTM) conventions, study Day 1 corresponds to the day of the first randomized study medication dose (ie, Week 0 visit).

The last day on study (ie, last visit) is the Week 40/early termination visit date; if this visit spans multiple days, then the last visit date is used. If no early termination visit is attended, because for example the subject is lost to follow-up, then the last on-study visit date is used.

Total exposure to study medication

Total exposure to randomized study medication in mg is calculated as (number of infusions * 0 mg) for subjects receiving placebo and (number of infusions * 0.240 mg) after Amendment 3 and (number of infusions * 0.120 mg) prior to Amendment 3 for subjects receiving GDNF. Note that the switch to higher dose per infusion occurred on and after 17JUN2013, the date of final sponsor approval of Amendment 3. This calculation assumes that the entire dose was infused at each administration.

Unblinded dosing information collected from the monitored pharmacy records is dummied (for dose of placebo) and blinded by PRA unblinded personnel before transfer to the blinded team for blinded programming and dry runs.

Total good-quality ON time per day

Total good-quality ON time per day is defined as the sum of ON time per day without dyskinesias + ON time per day with non-troublesome dyskinesias from the PD diary, where each half-hour interval checked contributes 30 minutes to the sum.

Treatment-emergent adverse event

Treatment-emergent AEs are those AEs starting on or after the first randomized study medication dose date. AEs are collected through Week 40 or up to 28 days after the last dose of randomized study medication for subjects who discontinue the study early. When an AE falls on the same day as first randomized study medication, the time of infusion start for the first dose of randomized study medication will be compared to the start time of the AE to determine if the AE is a TEAE. If either of the times is missing, then the AE will be considered a TEAE. In the case of missing or partial dates, any AE that could have begun on or after the first randomized study medication dose date is assumed to be treatment-emergent.

UPDRS score, OFF state total and ON state total

OFF state UPDRS total score is the sum of the OFF state motor score (part III) and the OFF state ADL score (part II).

ON state UPDRS total score is the sum of the ON state motor score (part III) and the ON state ADL score (part II).

Visits and visit windows

Scheduled visits are as follows:

- Screening Visit 1
- Screening Visit 2

- Surgery and healing phase
- Week 0 / baseline
- [Week 2 prior to Amendment 3]
- Week 4
- [Week 6 prior to Amendment 3]
- Week 8
- [Week 10 prior to Amendment 3]
- Week 12
- [Week 14 prior to Amendment 3]
- Week 16
- [Week 18 prior to Amendment 3]
- Week 20
- [Week 22 prior to Amendment 3]
- Week 24
- [Week 26 prior to Amendment 3]
- Week 28
- [Week 30 prior to Amendment 3]
- Week 32
- [Week 34 prior to Amendment 3]
- Week 36
- [Week 38 prior to Amendment 3]
- Week 40 (Month 9 endpoint)/early termination

The Week 40/early termination visit may occur at any time on or after study Day 1 for early terminators of the study. For subjects who complete the study, this visit occurs at Week 40 and its data is referred to as "Week 40 visit data." For subjects who terminate from the study early, Week 40/early termination assessments are assigned to an earlier scheduled visit using the study day of the early termination visit date. If only the day of the early termination visit date is missing, then the 1st day of the month is imputed. If the early termination visit date has missing month and/or year after the data query process, the Week 40/early termination assessments are not assigned to an earlier visit.

The Week 40/early termination visit may occur during a visit period in which a scheduled visit has already occurred. In this case, the visit that is closer to the nominal study day is selected for tabulations and plots by visit.

The visit windows in Table 3 are applied (see definitions of completion of study, early termination of study, and study day in this section). The visit schedules are distinguished using either database visit labels for Pilot Stage subjects or by study day for Primary Stage subjects.

Table 3. Visit Windows

Visit	Nominal Study Day	Study Day Range Prior to Amendment 3	Study Day Range After Amendment 3
Week 0	0	-6-7	-13-14
Week 2	14	8-21	
Week 4	28	22-35	15-42
Week 6	42	36-49	
Week 8	56	50-63	43-70
Week 10	70	64-77	
Week 12	84	78-91	71-98
Week 14	98	92-105	
Week 16	112	106-119	99-126
Week 18	126	120-133	
Week 20	140	134-147	127-154
Week 22	154	148-161	
Week 24	168	162-175	155-182
Week 26	182	176-189	
Week 28	196	190-203	183-210
Week 30	210	204-217	
Week 32	224	218-231	211-238
Week 34	238	232-245	
Week 36	252	246-259	239-259
Week 38	266	260-273	
Week 40	280	274-287	260-304

The Week 40 visit window is wider than for other visits since the number and duration of assessments required special arrangements in some cases in order to minimize patient burden. Independent of the Week 40 visit window, and consistent with the definition of major protocol deviations (see Appendix 2), performance of the Week 40 OFF state UPDRS motor score (part III) assessment before Day 267 or after Day 294 is considered a major protocol deviation.

Other than the Week 40/early termination visit, postbaseline unscheduled visit values are not windowed and are excluded from tabulations by visit. All unscheduled visit values are included in data listings.

6.0 Analysis Populations

Enrolled and randomized subjects are defined in Section 5.0, as are not randomized subjects.

6.1 Intent-to-Treat Populations

6.1.1 ITT Primary Population

The ITT Primary Population is defined as all randomized Primary Stage subjects. This population is used for analyses of the primary efficacy endpoint, all secondary endpoints, some supplementary efficacy endpoints, and all imaging endpoints. The ITT Primary Population is also used for all summaries of demographic and baseline characteristics. Subjects are counted according to their randomized treatment group.

6.1.2 ITT Overall Population

The ITT Overall Population is defined as all randomized Pilot Stage and all randomized Primary Stage subjects. This population is used for analyses of some supplementary efficacy endpoints and the PET

imaging endpoint, as well as demographic and selected baseline characteristics. Subjects are counted according to their randomized treatment group.

6.1.3 ITT Pilot Population

The ITT Pilot Population is defined as all randomized Pilot Stage subjects. This population is defined for completeness and used predominantly for data listings by subject.

6.2 Per-Protocol Population

The Per-Protocol Population is defined as all ITT Primary Population subjects who did not have a major protocol deviation. This population is used for additional analyses of the primary and secondary efficacy endpoints and PET imaging endpoints only. Subjects are counted according to their randomized treatment group. See also Section 5.0 discussion of protocol deviations and Section 9.3 describing summaries of protocol deviations.

6.3 Safety Populations

6.3.1 Safety Primary Population

The Safety Primary Population is defined as all randomized Primary Stage subjects who received at least one dose of study medication. This population is used for all safety analyses. Subjects are counted according to the group of the treatment actually received. If both treatments are received in error (GDNF and placebo), any subject who received any amount of GDNF infusion is counted as actually receiving GDNF.

6.3.2 Safety Overall Population

The Safety Overall Population is defined as all randomized Pilot Stage subjects who received at least one dose of study medication and all randomized Primary Stage subjects who received at least one dose of study medication. This population is used to repeat selected safety analyses only. Subjects are counted according to the group of the treatment actually received. If both treatments are received in error (GDNF and placebo), any subject who received any amount of GDNF infusion is counted as actually receiving GDNF.

6.3.3 Safety Pilot Population

The Safety Pilot Population is defined as all randomized Pilot Stage subjects who received at least one dose of study medication. This population is defined for completeness, but used only for data listings by subject.

6.3.4 Safety Enrolled Population

The Safety Enrolled Population is defined as all enrolled subjects (Primary and Pilot Stages combined), including not randomized subjects (see definition in Section 5.0). This population is used for analyses of pre-treatment AEs.

7.0 Interim Analyses

No interim analysis is planned for the study.

8.0 Data Review

8.1 Data Handling and Transfer

Data management for this study is performed by PRA. PRA performs data processing according to approved procedures including database specifications, CRF tracking, and dictionary coding and data validation. A quality control of site responses to data queries is also performed.

Data are entered by the investigational site into CRFs, which are entered by PRA into a clinical database built with Oracle Clinical version 4.5.3 and exported as SAS® version 9.4 or higher datasets (SAS Institute, Inc., Cary, NC). Converted datasets are created using SAS® and following CDISC SDTM conventions (v3.1.2 implementation guide v1.2). Derived analysis datasets are generated using SAS® and following standard CDISC Analysis Dataset Model conventions (implementation guide v1.0). Data analyses including summary tables, figures, and listings (TFLs) are produced using SAS®.

No central laboratory is used for this study. Local laboratory results are collected in the CRF in standard units along with clinical significance. Local laboratory reference ranges are collected outside of the CRF and sent to PRA directly.

Medical history and AEs are coded using MedDRA version 19.0 to assign a system organ class (SOC) and preferred term (PT) to each AE. Prior and concomitant medications are coded to preferred names using the World Health Organization Drug Dictionary Enhanced (WHODRUG DDE, 2016Mar01). Anatomical Therapeutic Chemical (ATC) classification coding is included.

PRA's data handling and transfer procedures are documented separately in the study specific data management plan.

8.2 Data Screening

Beyond the data screening built into the PRA Data Management Plan, the PRA programming of analysis datasets and TFLs provides additional data screening. Presumed data issues are output into SAS® logs identified by the word “Problem” and extracted from the logs by a SAS® macro and sent to Data Management.

Review of a post-freeze, random-allocated TFL run on the blinded, frozen database allows for further data screening prior to database lock. The post-freeze TFLs are discussed with the sponsor and client in a data review meeting to identify any final data issues and seek corrections prior to database lock. Database lock and unblinding must be approved by the approvers of the SAP.

9.0 Statistical Methods

All analyses use SAS® version 9.4 or higher. Summary tables are organized by treatment group (except for analyses of pre-treatment AEs). Important CRF data are included in data listings, sorted by treatment group, subject, and by visit within subject. With the exception of subject disposition and pre-treatment AE data, data for not randomized subjects (see definition in Section 5.0) are not listed, but are included in the SDTM datasets.

Unless otherwise noted, categorical data are presented using counts and percentages, with the number of subjects in the analysis population by treatment group as the denominator for percentages. Percentages are rounded to one decimal place. Continuous data, unless otherwise noted, are summarized using the number of observations (n), mean, SD, median, minimum, and maximum. Minima and maxima are rounded to the precision of the original value, and means, medians, and 95% confidence intervals (CIs) are rounded to 1 decimal place greater than the precision of the original value. SD is rounded to 2 decimal places greater than the precision of the original value, up to a maximum of 3 decimal places.

Any hypothesis testing is performed with a 2-sided alternative at the level of $\alpha = 0.05$. P-values are presented with 4 decimal places. No adjustments for multiplicity are made.

9.1 Missing Data Methods

9.1.1 Missing or Partial Dates

Missing or partial dates for first PD symptom and diagnosis, AEs, prior and concomitant medications, dosing records, and Week 40/early termination visits are imputed as described in Section 5.0 (see definitions for duration since PD symptom/diagnosis, TEAE, prior medication, concomitant medication, and visit windows).

9.1.2 Missing Efficacy Data

For the primary endpoint, missing Week 40 UPDRS motor score (part III) data are not imputed.

Missing Week 40 UPDRS motor score (part III) data for one of the sensitivity analyses of the primary efficacy endpoint are imputed using last observation carried forward (LOCF) imputation (see Section 9.7.1.2.2). LOCF imputation is performed by replacing missing data for a subject at a given time point with the last available observed data for that subject at an earlier postbaseline time point.

For secondary endpoints, missing Week 40 UPDRS motor score (part III), UPDRS ADL score (part II), UPDRS mentation, behavior, and mood score (part I), UPDRS complications of therapy score (part IV), and UPDRS total score (sum of motor + ADL scores) data are not imputed. Missing Week 40 PD motor fluctuation diary ratings are not imputed; see Section 9.7.2.6 for details on handling of missing and duplicate PD motor fluctuation diary ratings.

The handling of missing data for supplementary endpoints is described in the appropriate parts of Section 9.7.3.

For imaging endpoints, missing data are not imputed.

9.1.3 Missing Data for Questionnaires

There are 2 levels of missing data possible for questionnaires: either the entire instrument was not assessed at a scheduled time point, or one or more individual items on the instrument were left blank. In the former case of an entirely missed assessment, no imputation is performed.

In the latter case of one or more individual items missed, imputation is performed according to the scoring instructions of the instrument. If the scoring instructions do not address individual missing items, then the imputation method depends on the frequency of postbaseline scheduled assessments. For instruments that have multiple postbaseline scheduled time points, individual missing items are imputed using LOCF (ie, the score for the missing item is taken from the last non-missing postbaseline time point). For instruments that have only one postbaseline scheduled time point and at least 5 individual items in the subscale or scale being scored, individual missing responses are imputed using the average of non-missing scores. An exception to this rule occurs if more than half of the individual items are missing, in which case no imputation is performed and the subscale or scale score is left missing. Finally, for instruments that have only one postbaseline scheduled time point and fewer than 5 individual items (eg, SNAQ), the total score is considered missing if one or more individual response is missing.

Handling of individual missing items for each scale is discussed in the appropriate parts of Section 9.7 and Section 9.9.

9.1.4 Missing Safety Data

No imputation is performed for missing safety data other than questionnaire data.

9.1.5 Special Arrangements for Subject 45

Special arrangements have been made for subject 45 who had a conus injury during the study that was unrelated to study treatment or device. As a result of this, items 27, 28, 29 and 30 of the UPDRS score could not be completed beyond Week 8. Item 22, although recorded, is considered to be confounded. Therefore, for this subject, these 5 items are excluded from all calculations of the UPDRS motor score (part III) used in the efficacy analyses, and a truncated score including all other items of part III is used instead. UPDRS parts I, II and IV were collected as far as possible, but the data are not included in the related efficacy analyses because they are considered to be confounded due to the injury. Timed walk could not be done after Week 8 and is therefore excluded from efficacy analyses. PDQ 39 (items 14 to 39), EQ-5D and NMSS were recorded but are considered to be confounded due to the injury and are therefore excluded from efficacy analyses.

9.2 Subject Disposition

A tabulation of subject disposition is provided for the following categories (see Section 6.0 for population definitions):

- Numbers of Pilot Stage subjects enrolled, undergoing surgery, operated but not randomized, randomized (ITT Pilot Population), treated (Safety Pilot Population), and randomized but not treated.
- Numbers of Primary Stage subjects enrolled, undergoing surgery, operated but not randomized, randomized (ITT Primary Population), treated (Safety Primary Population), randomized but not treated, and included in the Per-Protocol Population.
- Numbers of Pilot Stage + Primary Stage subjects enrolled, undergoing surgery, operated but not randomized, randomized (ITT Overall Population), treated (Safety Overall Population), and randomized but not treated.

The number and percentage of subjects who completed the Week 40 visit is summarized for the ITT Primary Population and ITT Overall Population by treatment group and overall, together with the number and percentage of subjects who withdrew from the study prematurely and a breakdown of the corresponding primary reasons for early termination. See Section 5.0 for definitions of completion of study and early termination.

Disposition data are listed by subject, as are population inclusion data (showing which subjects are included in which analysis population). A separate listing of subjects who gave initial informed consent but failed screening is provided. Data for informed consent, inclusion/exclusion criteria, post-surgery randomization review, randomization approval, and randomization are not listed, but are included in SDTM datasets.

9.3 Protocol Deviations

Protocol deviations are presented by treatment group and overall for the ITT Primary Population by deviation category (major and minor deviations) and deviation name (only major deviations), displaying the number and percentage of subjects in each group to which each deviation category and deviation name apply. Protocol deviations are discussed in Section 5.0, and the Per-Protocol Population is defined in Section 6.2.

Major and minor protocol deviation data are listed by study stage, treatment group, and subject.

9.4 Demographic and Baseline Characteristics

9.4.1 Demographic Characteristics

The following demographic characteristics are tabulated by treatment group and overall for the ITT Primary Population and the ITT Overall Population.

- Age (years)
- Age group (< 65, ≥ 65 years)
- Sex (female, male)
- Race (white, black, Asian, Native Hawaiian or other Pacific Islander, American Indian or Alaska Native, other)
- Ethnicity (Hispanic or Latino, not Hispanic or Latino)
- Baseline weight (kg)
- Baseline height (m)
- Baseline BMI (kg/m^2; see definition in Section 5.0)
- National Adult Reading Test (NART) error score (points)

Demographic data are listed by study stage, treatment group, and subject. Education data are included in SDTM datasets, but are not listed.

9.4.2 Parkinson's Disease History at Screening

The following PD history items are tabulated by treatment group and overall for the ITT Primary Population and the ITT Overall Population.

- Duration since first PD symptom (years; see definition in Section 5.0)
- Duration since PD diagnosis (years; see definition in Section 5.0)
- Hoehn and Yahr stage in OFF state (0, 1, 1.5, 2, 2.5, 3)
- OFF state UPDRS motor score (part III; points)
- ON state UPDRS motor score (part III; points)
- Total daily levodopa dose (mg; see definition in Section 5.0)
- Total daily levodopa equivalent dose (mg; see definition in Section 5.0)
- PD medications as recorded on the Levodopa and Levodopa Equivalent Medications CRF (eg, levodopa preparations, dopamine agonists, COMT inhibitors, MAO-B inhibitors, other)
- Responsiveness to levodopa (ie, percentage change in screening UPDRS motor score [part III] following a levodopa challenge; see definition of percentage change from baseline in Section 5.0)
- OFF time per day (hours)

Impulsiveness Behaviour Scale (UPPS-P) subscale scores are summarized by treatment group and overall for the ITT Primary Population only. The UPPS-P is a 59-item scale that measures impulsiveness in 5 subscales: (negative) urgency, (lack of) premeditation, (lack of) perseverance, sensation seeking, positive urgency. Each question is scored 1 to 4. A lower score indicates less impulsiveness. Individual missing items are imputed using the average of non-missing scores in each subscale. The score for each subscale is calculated from the responses to the individual items in the subscale.

PD history data and UPPS-P data are listed by study stage, treatment group, and subject.

9.4.3 General Medical History

General medical history items that are resolved at screening are summarized by treatment group and overall for the ITT Primary Population by MedDRA SOC and PT.

General medical history items that are current/active at screening are summarized separately by treatment group and overall for the ITT Primary Population by MedDRA SOC and PT.

General medical history data are listed by study stage, treatment group, and subject.

9.5 Prior and Concomitant Medications

Medications received prior to first dose of study medication, categorized by ATC class (level 2) and preferred name (level 5) according to WHODRUG DDE, are summarized by treatment group for the ITT Primary Population. The number and percentage of subjects using any prior medication is displayed together with the number and percentage of subjects using at least one medication within each ATC class and preferred name. See definition of prior medications in Section 5.0.

Medications received concomitantly with study medication, categorized by ATC class and preferred name according to WHODRUG DDE, are summarized by treatment group for the ITT Primary Population. Separate summaries are presented for concomitant PD medications and other concomitant medications. The number and percentage of subjects using any concomitant medication is displayed together with the number and percentage of subjects using at least one medication within each ATC class and preferred name. See definition of concomitant medications in Section 5.0.

Prior and concomitant medication data are listed by study stage, treatment group, and subject, with prior versus concomitant flagged. Levodopa challenge dosing data are not listed, but are included in SDTM datasets.

9.6 Surgery and Test Infusions

9.6.1 Catheter Trajectories and Positioning Accuracy

Initial catheter placement trajectory and catheter repositioning surgery trajectory are tabulated by category (vertical, horizontal anterior-posterior and horizontal posterior-anterior) by treatment group and overall for the ITT Primary Population.

Catheter positioning accuracy post-surgery (see definition in Section 5.0) is assessed for the initial surgery and any subsequent repositioning surgeries necessary. The difference between planned and actual target in mm is reported with summary statistics for all catheters combined by treatment group and overall for the ITT Primary Population. No hypothesis testing is done.

Catheter trajectory data and catheter positioning accuracy data are listed by study stage, treatment group, and subject. All other surgery data, including data from post-operative CT scans, are not listed, but are included in SDTM datasets.

9.6.2 Contrast-Enhanced Test Infusions with T1-Weighted MRI Prior to Start of Study Medication

The number of subjects with one, 2, and 3 or more contrast-enhanced test infusions with T1-weighted MRI prior to start of study medication is tabulated by treatment group and overall for the ITT Primary Population. Only subjects with contrast-enhanced test infusions followed by T1-weighted MRI (with or without additional T2-weighted MRI) are counted in this analysis; subjects with test infusions that are only followed by T2-weighted MRI are not counted. Counting is per occasion (not per putamen), so that a bilateral test infusion is counted as only one test infusion.

Data for all test infusions at healing phase, interim, and Week 40 visits, including repeat test infusions, are listed by study stage, treatment group, and subject.

9.7 Efficacy Analyses

The primary efficacy analysis uses the ITT Primary Population. Sensitivity analyses of the primary endpoint are performed using the ITT Overall Population and/or the Per-Protocol Population or using different analytic methods in the ITT Primary Population. Analyses of secondary endpoints are performed using the ITT Primary Population, ITT Overall Population, and Per-Protocol Population in a manner similar to the analyses of the primary endpoint. Analyses of supplementary endpoints (other than those already covered in analyses of the primary and secondary endpoints) are performed for the ITT Primary Population.

All efficacy endpoints are tested at the $\alpha = 0.05$ level, 2-sided without multiplicity adjustment. End of study/early termination visit data are windowed to the appropriate scheduled visit and are not included in Week 40 scheduled visit data (see Section 5.0 definition of visit windows). Postbaseline UPDRS motor (part III) and ADL (part II) scores are assessed by raters who are blinded to all other aspects of the subject's condition.

For efficacy endpoints, other than the second sensitivity analysis of the primary efficacy endpoint in Section 9.7.1.2.2, missing Week 40 UPDRS motor score (part III), UPDRS ADL Score (part II), UPDRS mentation, behavior, and mood score (part I), UPDRS complications of therapy score (part IV), and UPDRS total score (sum of motor + ADL scores) data are not imputed.

UPDRS data are listed by study stage, treatment group, and subject for all UPDRS parameters, including OFF and ON state motor score (part III), OFF and ON state ADL score (part II), OFF and ON state UPDRS total score (sum of motor + ADL scores), mentation, behavior, and mood score (part I), and complications of therapy score (part IV). Changes from baseline in each score are listed.

9.7.1 Analyses of Primary Efficacy Endpoint

9.7.1.1 Primary Analysis: MMRM of Primary Efficacy Endpoint (ITT Primary Population)

The percentage change from baseline to Week 40 in the practically defined OFF state UPDRS motor score (part III) is compared between treatment groups for the ITT Primary Population using a mixed-effect model with repeated measures (MMRM). Baseline UPDRS score is a covariate, treatment group and visit and treatment group*visit are fixed effects, and subject within treatment group is a random effect. The covariance matrix is unstructured. The following SAS® code fragment approximates the analysis:

```
proc mixed data=<input>;
    class <usubjid> <trt01p> <visit>;
    model <pchg> = <base> <trt01p> <visit> <trt01p>*<visit>/ ddfm=KR [A];
    repeated / type=un subject=<usubjid>(<trt01p>);
    lsmeans <trt01p> / pdiff;
run;
```

[A] The Kenward and Roger method for calculating the denominator degrees of freedom for tests of fixed effects will be used.

Summary statistics are tabulated by treatment group expressed as least squares means with 95% CIs, difference between least squares means (GDNF – placebo) with 95% CIs, and corresponding p-value. Subjects who are missing their Week 40 UPDRS motor score (part III) value are counted in the model at all postbaseline time points for which data are present. Scheduled visits are Week 0 (baseline; see Section 5.0 definition of baseline) and Weeks 8, 16, 24, 32, and 40.

Percentage change from baseline in OFF state UPDRS motor score (part III) over time is plotted on a line plot displaying mean values and standard error (SE) bars. Individual subject data for OFF state UPDRS motor score (part III) over time is also plotted on a line plot by treatment group (displayed in separate graphs).

The GDNF treatment group is judged superior compared with placebo if there is sufficient statistical evidence to reject the following null hypothesis in the direction favorable to GDNF:

> H_0: *No significant difference in the percentage change from baseline to Week 40 in the OFF state UPDRS motor score (part III) between GDNF and placebo*
>
> and accept the alternative hypothesis:
>
> H_a: *A significantly greater percentage decrease (lower UPDRS is better) in the change from baseline to Week 40 in the OFF state UPDRS motor score (part III) for GDNF relative to placebo*

It is also possible that a significantly greater percentage decrease for placebo as compared with GDNF is found, in which case placebo is judged superior to GDNF (ie, the test is 2-sided).

9.7.1.2 Sensitivity Analyses of Primary Efficacy Endpoint

9.7.1.2.1 First Sensitivity Analysis: MMRM of Primary Efficacy Endpoint (ITT Overall Population and Per-Protocol Population)

The primary efficacy analysis is repeated for the ITT Overall Population and the Per-Protocol Population.

9.7.1.2.2 Second Sensitivity Analysis: ANCOVA of Primary Efficacy Endpoint (ITT Primary Population)

This analysis of the primary efficacy endpoint utilizes an analysis of covariance (ANCOVA) model adjusted for baseline UPDRS score. Input data are either restricted to observed data at baseline and Week 40 only, or missing Week 40 data are imputed using LOCF. Estimated mean difference (GDNF – placebo), 95% CIs, and p-value are reported. This analysis is performed for the ITT Primary Population only.

In addition, summary statistics as well as inferential statistics (observed data ANCOVA) are displayed at each visit.

9.7.1.2.3 Third Sensitivity Analysis: MMRM of Primary Efficacy Endpoint Using Screening Instead of Week 0 Values as Baseline (ITT Primary Population)

The primary efficacy analysis is repeated using screening instead of Week 0 values as the baseline values. If multiple screening values are present for a given subject, then the last screening value is used.

9.7.1.2.4 Fourth Sensitivity Analysis: MMRM of Primary Efficacy Endpoint Excluding Subjects with Vertical Catheter Trajectory (ITT Primary Population)

The primary efficacy analysis is repeated excluding subjects whose catheters are positioned using a different surgical approach (ie, vertical instead of horizontal trajectory). Based on initial experience in the study, the size and position of the putamenal volume of distribution is expected to differ depending on the trajectory.

9.7.1.3 Subgroup Analyses of Primary Efficacy Endpoint

Subgroup analyses using the factors specified in Section 4.4 may be performed as required.

9.7.2 Analyses of Secondary Efficacy Endpoints

9.7.2.1 Percentage Change From Baseline in ON State UPDRS Motor Score (Part III)

9.7.2.1.1 MMRM of ON State UPDRS Motor Score (Part III) (ITT Primary Population)

The percentage change from baseline to Week 40 in ON state UPDRS motor score (part III; following a levodopa challenge) is compared between treatment groups for the ITT Primary Population and reported in a manner identical to that for the primary efficacy endpoint using an MMRM. Summary statistics are tabulated by treatment group expressed as least squares means with 95% CIs, difference between least squares means (GDNF – placebo) with 95% CIs, and corresponding p-value.

Percentage change from baseline in ON state UPDRS motor score (part III) over time is plotted on a line plot displaying mean values and SE bars.

9.7.2.1.2 MMRM of ON State UPDRS Motor Score (Part III) (ITT Overall Population and Per-Protocol Population)

The analysis is repeated for the ITT Overall Population and the Per-Protocol Population.

9.7.2.1.3 ANCOVA of ON State UPDRS Motor Score (Part III) (ITT Primary Population)

Summary statistics are displayed by visit for the ITT Primary Population, with inferential statistics (observed data ANCOVA) displayed only at Week 40.

9.7.2.2 Percentage Change From Baseline in OFF State and ON State UPDRS ADL Score (Part II)

9.7.2.2.1 MMRM of OFF State and ON State UPDRS ADL Score (Part II) (ITT Primary Population)

The percentage change from baseline to Week 40 in OFF state and ON state UPDRS ADL score (part II) is compared between treatment groups for the ITT Primary Population and reported in a manner identical to that for the primary efficacy endpoint using an MMRM. Summary statistics are tabulated by treatment group expressed as least squares means with 95% CIs, difference between least squares means (GDNF – placebo) with 95% CIs, and corresponding p-value.

9.7.2.2.2 MMRM of OFF State and ON State UPDRS ADL Score (Part II) (ITT Overall Population and Per-Protocol Population)

The analysis is repeated for the ITT Overall Population and the Per-Protocol Population.

9.7.2.2.3 ANCOVA of OFF State and ON State UPDRS ADL Score (Part II) (ITT Primary Population)

Summary statistics are displayed by visit for the ITT Primary Population, with inferential statistics (observed data ANCOVA) displayed only at Week 40.

9.7.2.3 MMRM of Percentage Change From Baseline in OFF State and ON State UPDRS Total Score

9.7.2.3.1 MMRM of OFF State and ON State UPDRS Total Score (ITT Primary Population)

The percentage change from baseline to Week 40 in the OFF state and ON state UPDRS total score (sum of motor + ADL scores) is compared between treatment groups for the ITT Primary Population and reported in a manner identical to that for the primary efficacy endpoint using an MMRM.

Percentage change from baseline in OFF state and ON state UPDRS total score over time is plotted on line plots displaying mean values and standard error (SE) bars.

9.7.2.3.2 MMRM of OFF State and ON State UPDRS Total Score (ITT Overall Population and Per-Protocol Population)

The analysis is repeated for the ITT Overall Population and the Per-Protocol Population.

9.7.2.4 MMRM of Percentage Change From Baseline in UPDRS Mentation, Behavior, and Mood Score (Part I) (ITT Primary Population)

The UPDRS mentation, behavior, and mood score (part I) is compared between treatment groups for the ITT Primary Population and reported in a manner identical to that for the primary efficacy endpoint using an MMRM.

9.7.2.5 MMRM of Percentage Change From Baseline in UPDRS Complications of Therapy Score (Part IV) (ITT Primary Population)

The UPDRS complications of therapy score (part IV) is compared between treatment groups for the ITT Primary Population and reported in a manner identical to that for the primary efficacy endpoint using an MMRM.

9.7.2.6 Change From Baseline in PD Diary Ratings

PD motor fluctuation diary rating data are collected on scheduled visit days at baseline and every 8 weeks. For diary purposes, a day is defined as a 24-hour period broken into half-hour intervals starting with the 06:00 am interval and ending with the 05:30 am interval the following day. Missing Week 40 PD motor fluctuation diary ratings are not imputed.

A total of 3 diaries per scheduled visit day are completed by subjects. Each diary collects the state that represents the predominant status during each half-hour interval of the 24-hour period (asleep, OFF, ON without dyskinesias, ON with non-troublesome dyskinesias, ON with troublesome dyskinesias). Errors in diary data and multiple responses in the same half-hour interval are defined as missing data. Data for half-hour intervals with errors are not used for analysis, but the remaining data recorded on the diary are valid only if a maximum of 4 errors are present. If 5 or more errors are present in a given diary, then the entire diary is considered invalid and not used for analysis.

Among the valid diaries (up to 3) per subject and scheduled visit, the mean times of any given state over all valid diaries are used for analysis. From these data, total OFF time per day, total good-quality ON time per day, and ON time per day with troublesome dyskinesias are estimated for the subject and scheduled visit, where each half-hour interval checked contributes 30 minutes to the sum (see definitions in Section 5.0).

9.7.2.6.1 MMRM of PD Diary Ratings (ITT Primary Population)

The change from baseline to Week 40 in PD motor fluctuation diary ratings is compared between treatment groups for the ITT Primary Population and reported in a manner identical to that for the primary efficacy endpoint using an MMRM. Parameters include:

- Total OFF time per day
- Total good-quality ON time per day (sum of ON time per day without dyskinesias + ON time per day with non-troublesome dyskinesias)
    - ON time per day without dyskinesias
    - ON time per day with non-troublesome dyskinesias
- ON time per day with troublesome dyskinesias

Summary statistics are displayed by visit, but inferential statistics are presented only for Week 40. Scheduled visits are Screening, Week 0 (baseline; see Section 5.0 definition of baseline) and Weeks 8, 16, 24, 32, and 40.

Change from baseline in total OFF time per day and change from baseline in total good-quality ON time per day are plotted on line plots displaying mean values and SE bars.

PD diary ratings are listed by study stage, treatment group, and subject. Diary training, diary dispensation, and diary collection and review are not listed, but are included in SDTM datasets.

9.7.2.6.2 MMRM of PD Diary Ratings (ITT Overall Population and Per-Protocol Population)

The analysis is repeated for the ITT Overall Population and the Per-Protocol Population.

9.7.3 Analyses of Supplementary Efficacy Endpoints

Primary and secondary endpoints in the ITT Overall Population are defined as supplementary efficacy endpoints. The respective analyses are described in the relevant parts of Sections 9.7.1 and 9.7.2, together with the analyses of these endpoints in the ITT Primary Population and Per-Protocol Population. The text below describes the analyses for the other supplementary efficacy endpoints.

9.7.3.1 MMRM of Change From Baseline in Timed Walking Test (ITT Primary Population)

During the timed walking test, the subject walks as fast as possible 7 meters back and forth including turning. The time to perform this test is recorded for 2 trials in the OFF state and 2 trials in the ON state after levodopa challenge.

The change from baseline to Week 40 in timed walking test is compared between treatment groups for the ITT Primary Population and reported in a manner identical to that for the primary efficacy endpoint using an MMRM. Results are summarized by treatment group expressed as least squares means with 95% CIs, difference between least squares means (GDNF – placebo) with 95% CIs, and corresponding p-value. Timed walking test parameters are OFF state timed walking test and ON state timed walking test in seconds.

Summary statistics are displayed by visit, but inferential statistics are presented only for Week 40. Scheduled visits are Week 0 (baseline; see Section 5.0 definition of baseline) and Weeks 8, 16, 24, 32, and 40. The results of the two separate trials per state at each visit are averaged and the mean used for analysis of each state. If only the first trial is completed for a particular state, then the second trial result will be imputed with the worst non-missing second trial result from all subjects for that particular state during the study. If both trials are missing, then the endpoint is not reported for that visit.

Timed walking test data are listed by study stage, treatment group, and subject. Changes from baseline in the results are listed.

9.7.3.2 MMRM of Change From Baseline in Timed Tapping Test (ITT Primary Population)

During the timed tapping test, the subject alternates tapping the index finger for 20 seconds between 2 points spaced 30 cm apart. The test is performed twice for each hand in the OFF state and twice for each hand in the ON state after levodopa challenge.

The change from baseline to Week 40 in timed tapping test is compared between treatment groups for the ITT Primary Population and reported in a manner identical to that for the primary efficacy endpoint using an MMRM. Results are summarized by treatment group expressed as least squares means with 95% CIs, difference between least squares means (GDNF – placebo) with 95% CIs, and corresponding p-value. Timed tapping test parameters are OFF state timed tapping test (left and right hand separately) and ON state timed tapping test (left and right hand separately) in number of taps completed in 20 seconds.

Summary statistics are displayed by visit, but inferential statistics are presented only for Week 40. Scheduled visits are Week 0 (baseline; see Section 5.0 definition of baseline) and Weeks 8, 16, 24, 32, and 40. The results of the two separate trials per state at each visit per hand are averaged and the mean used for analysis of each hand in each state. If only the first trial is completed for a particular state, then the second trial result will be imputed with the worst non-missing second trial result from all subjects for that particular state during the study. If both trials are missing, then the endpoint is not reported for that visit.

Change from baseline in OFF and ON state timed tapping test over time is plotted on a line plot displaying mean values and standard error (SE) bars. Individual subject data for OFF and ON state timed tapping test over time is also plotted on a line plot by treatment group (displayed in separate graphs).

Timed tapping test data are listed by study stage, treatment group, and subject. Changes from baseline in the results are listed.

9.7.3.3 MMRM of Change from Baseline in NMSS Score (ITT Primary Population)

The NMSS is an interview-based scale used to rate non-motor symptoms commonly occurring in PD (developed by the International Parkinson's Disease Non-Motor Group). It is administered with the subject in the ON state. The 30-item scale rates symptoms that occurred in the preceding month in 9 domains (cardiovascular including falls; sleep/fatigue; mood/cognition; perceptual problems/hallucinations; attention/memory; gastrointestinal tract; urinary; sexual function; miscellaneous). Each item is rated from 0 (none) to 3 (severe) for severity and from 1 (rarely) to 4 (very frequent) for frequency. The score for each item is the product of the severity rating multiplied by the frequency. The maximum score for an individual item is 12. The higher the score, the worse the subject's condition. Individual item scores in each domain are summed to give the domain score, and the domains are summed to give the total score. The maximum NMSS total score is 360. Missing individual item scores are imputed using LOCF.

The change from baseline to Week 40 in NMSS data is compared between treatment groups for the ITT Primary Population and reported in a manner identical to that for the primary efficacy endpoint using an MMRM. Results are summarized by treatment group expressed as least squares means with 95% CIs, difference between least squares means (GDNF – placebo) with 95% CIs, and corresponding p-value. NMSS parameters are the 9 NMSS domains and the NMSS total score.

Summary statistics are displayed by visit, but inferential statistics are presented only for Week 40. Scheduled visits are Screening Visit 2 (baseline; see Section 5.0 definition of baseline) and Weeks 12, 24, and 40.

NMSS data are listed by study stage, treatment group, and subject.

9.7.3.4 ANCOVA of Change From Baseline in PDQ-39 Score (ITT Primary Population)

The PDQ-39 is a self-administered 39-item PD-specific scale that rates symptoms that occurred in the preceding month in the 8 dimensions listed below. Each item is rated from 0 (never) to 4 (always) for frequency. Each dimension is calculated as a scale from 0 to 100 based on the following formulas:

- Mobility = [(Sum of Scores of questions 1 – 10)/ (4*10)] *100
- Activities of Daily Living = [(Sum of Scores of questions 11 – 16)/(4*6)]*100
- Emotional Well Being = [(Sum of Scores of questions 17 – 22)/(4*6)]*100
- Stigma = [(Sum of Scores of questions 23 – 26)/(4*4)]*100
- Social Support = [(Sum of Scores of questions 27 – 29)/(4*3)]*100
    - If respondents indicate that they do not have a spouse/partner on question 28, then Social Support = [(Sum of Scores of questions 27 & 29)/(4*2)]*100
- Cognitions = [(Sum of Scores of questions 30 – 33)/(4*4)]*100
- Communication = [(Sum of Scores of questions 34 – 36)/(4*3)]*100
- Bodily Discomfort = [(Sum of Scores of questions 37 – 39)/(4*3)]*100

The total score, or single index, is the average of all 8 dimension scores. The higher the score, the worse the subject's condition. If the response to an individual question is missing, then no score is calculated for that dimension and therefore the single index score cannot be calculated.

Analysis of PDQ-39 data on the ITT Primary Population utilizes an ANCOVA model adjusted for baseline PDQ-39 score. Input data are restricted to observed data at baseline and Week 40 only. PDQ-39 parameters are the 8 PDQ-39 dimensions and the single index (total) PDQ-39 score.

Summary statistics are displayed for Screening Visit 2 (baseline; see Section 5.0 definition of baseline) and Week 40. Estimated mean difference (GDNF – placebo), 95% CIs, and p-values are reported.

PDQ-39 data are listed by study stage, treatment group, and subject.

9.7.3.5 ANCOVA of Change From Baseline in EQ-5D Score (ITT Primary Population)

The EQ-5D is a subject self-report measure of quality of life consisting of a questionnaire and a visual analog scale. The questionnaire comprises 5 questions on mobility, self-care, usual activities, pain/discomfort, and anxiety/depression, with three possible answers for each item (1=no problem, 2=moderate problem, 3=severe problem). The visual analog scale serves as an indicator of general health status; the scale ranges from 0 to 100, where 0 indicates worst health and 100 indicates best health. Missing values for individual questions are coded as 9; missing values for the visual analog scale are coded as 999. Missing values are not included in observed data analyses.

EQ-5D questionnaire data are reported using frequency counts and percentages for Screening Visit 2 (baseline; see Section 5.0 definition of baseline) and Week 40. Input data are restricted to observed data at baseline and Week 40 only. Parameters are the frequency counts and percentages of subjects with the different answers for each of the 5 questions.

Analysis of EQ-5D visual analog scale data on the ITT Primary Population utilizes an ANCOVA model adjusted for baseline EQ-5D visual analog scale score. Input data are restricted to observed data at baseline and Week 40 only. The parameter is the visual analog scale score. Summary statistics are displayed for Screening Visit 2 (baseline; see Section 5.0 definition of baseline) and Week 40. Estimated mean difference (GDNF – placebo), 95% CIs, and p-values are reported.

EQ-5D data are listed by study stage, treatment group, and subject.

9.7.3.6 ANCOVA of Change From Baseline in SNAQ Score (ITT Primary Population)

The SNAQ is a self-administered 4-question instrument with total scores ranging from 4 to 20 (4=poor appetite, 20=good appetite). The SNAQ has one total score. If an individual question is not answered, then the total score is considered missing.

Analysis of SNAQ data on the ITT Primary Population utilizes an ANCOVA model adjusted for baseline SNAQ score. Input data are restricted to observed data at baseline and Week 40 only.

Summary statistics are displayed for Screening Visit 2 (baseline; see Section 5.0 definition of baseline) and Week 40. Estimated mean difference (GDNF – placebo), 95% CIs, and p-values are reported.

SNAQ data are listed by study stage, treatment group, and subject.

9.7.3.7 ANCOVA of Change From Baseline in Total Daily Levodopa Dose and Total Daily Levodopa Equivalent Dose (ITT Primary Population)

Analysis of change from baseline to Week 40 data on the ITT Primary Population utilizes an ANCOVA model adjusted for baseline total daily levodopa dose and total daily levodopa equivalent dose, respectively (see definitions in Section 5.0). Input data are restricted to observed data at baseline and Week 40 only (no imputation of missing data).

Summary statistics are displayed for Week 0 (baseline; see Section 5.0 definition of baseline) and Week 40. Estimated mean difference (GDNF – placebo), 95% CIs, and p-values are reported.

Levodopa dose and levodopa equivalent dose data are listed by study stage, treatment group, and subject. Separate listings are provided for actual total daily dose of each PD medication and for the total daily dose of levodopa and the total daily dose of levodopa equivalent.

9.8 Imaging Analyses

Missing data are not imputed for imaging endpoints.

9.8.1 MRI Analyses

Post-infusion MRI data needed for the imaging analyses described in this section are listed for Primary Stage subjects; although not analyzed or tabulated, corresponding post-infusion MRI data are also listed for Pilot Stage subjects. All other MRI data, including real-time MRI data, are not listed, but are included in SDTM datasets.

9.8.1.1 ANCOVA of Change From Baseline in Volume of Distribution of Infusate as Determined by Contrast-Enhanced T1-Weighted MRI (ITT Primary Population)

Analysis of change from baseline to Week 40 in volume of distribution of infusate between treatment groups on the ITT Primary Population utilizes an ANCOVA model adjusted for baseline volume of distribution. Input data are restricted to observed data at the end of the healing phase and at Week 40 only. The parameter is the volume of distribution (in mL), separately for left and right hemispheres, as determined by contrast-enhanced T1-weighted MRI.

Summary statistics are displayed for the last test infusion at the end of the healing phase (baseline; see Section 5.0 definition of baseline) and Week 40. Estimated mean difference (GDNF – placebo), 95% CIs, and p-values are reported.

Volume of distribution data are listed by study stage, treatment group, and subject.

9.8.1.2 ANCOVA of Change from Baseline in Volume of Interest Coverage and Total Putamenal Coverage as Determined by Contrast-Enhanced T1-Weighted MRI (ITT Primary Population)

Analysis of change from baseline to Week 40 in VOI coverage and total putamenal coverage for the ITT Primary Population utilizes an ANCOVA model adjusted for baseline coverage. Input data are restricted to observed data at the end of the healing phase and at Week 40 only. Parameters include VOI coverage as a percentage of total VOI and total putamenal coverage as a percentage of total putamenal volume, separately for left and right putamen and for both putamina combined.

Summary statistics are displayed for the last test infusion at the end of the healing phase (baseline; see Section 5.0 definition of baseline) and Week 40. Estimated mean difference (GDNF – placebo), 95% CIs, and p-values are reported for each analysis.

VOI coverage (absolute and relative) and total putamenal coverage data, including underlying data (VOI, putamenal volume of distribution, and total volume of putamen) are listed by study stage, treatment group, and subject.

9.8.2 PET Analyses

9.8.2.1 ANCOVA of Change From Baseline in ^{18}F-DOPA Uptake as Determined by PET Scan (ITT Primary Population, ITT Overall Population, ITT Pilot Population, and Per-Protocol Population)

Analysis of change from baseline to Week 40 in PET imaging of ^{18}F-DOPA uptake data utilizes an ANCOVA model adjusted for baseline ^{18}F-DOPA uptake for the ITT Primary Population. Input data are restricted to observed data at baseline and Week 40 only. The parameter is the ^{18}F-DOPA uptake rate constant determined by PET, separately for left and right hemispheres. Four regions are assessed per hemisphere (anterior, central and posterior putamen, and caudate nucleus).

Summary statistics are displayed for Week 0 (baseline; see Section 5.0 definition of baseline) and Week 40. Estimated mean difference (GDNF – placebo), 95% CIs, and p-values are reported.

This analysis is repeated for the ITT Overall Population, ITT Pilot Population, and Per-Protocol Population. For the ITT Pilot Population, there is an additional time point at Week 12.

PET data for ^{18}F-DOPA uptake are listed by study stage, treatment group, and subject.

9.8.3 Correlation Analyses

9.8.3.1 Correlation Between Primary Study Endpoint and Volume of Interest Coverage and Total Putamenal Coverage at Baseline as Determined by Contrast-Enhanced T1-Weighted MRI (ITT Primary Population)

These analyses use non-parametric Spearman rank correlation to explore the relationship between percentage change from baseline to Week 40 in OFF state UPDRS motor score (part III) and either VOI coverage or total putamenal coverage after the last test infusion at the end of the healing phase by treatment group for the ITT Primary Population. Correlations are calculated separately for each treatment group. Parameters include percentage change from baseline to Week 40 in OFF state UPDRS motor score (part III) and both VOI coverage as a percentage of total VOI and total putamenal coverage as a percentage of total putamenal volume, for both putamina combined.

The estimated correlation coefficient, 95% CI, and p-value are tabulated for each analysis and treatment group, and scatterplots are provided.

9.8.3.2 Correlation Between Change from Baseline in NMSS Total Score and Total Putamenal Coverage at Baseline as Determined by Contrast-Enhanced T1-Weighted MRI (ITT Primary Population)

This analysis uses non-parametric Spearman rank correlation to explore the relationship between change from baseline to Week 40 in NMSS total score and total putamenal coverage after the last test infusion at the end of the healing phase by treatment group for the ITT Primary Population. Correlations are calculated separately for each treatment group. Parameters include change from baseline to Week 40 in NMSS total score and total putamenal coverage as a percentage of total putamenal volume, for both putamina combined.

The estimated correlation coefficient, 95% CI, and p-value are tabulated by treatment group, and scatterplots are provided.

9.8.3.3 Correlation Between Primary Study Endpoint and Change From Baseline in ^{18}F-DOPA Uptake as Determined by PET Scan (ITT Primary Population)

This analysis uses non-parametric Spearman rank correlation to explore the relationship between percentage change from baseline to Week 40 in OFF state UPDRS motor score (part III) and change from baseline to Week 40 in PET imaging of ^{18}F-DOPA uptake by treatment group for the ITT Primary Population. Correlations are calculated separately for each treatment group. Parameters include percentage change from baseline to Week 40 in OFF state UPDRS motor score (part III) and change from baseline to Week 40 in ^{18}F-DOPA uptake rate constant for anterior, central and posterior putamen, and caudate nucleus determined by PET, for both hemispheres combined.

The estimated correlation coefficient, 95% CI, and p-value are tabulated for each analysis and treatment group, and scatterplots are provided.

9.8.3.4 Correlation Between Change From Baseline in ^{18}F-DOPA Uptake as Determined by PET Scan and Volume of Interest Coverage and Total Putamenal Coverage at Baseline as Determined by Contrast-Enhanced T1-Weighted MRI (ITT Primary Population)

These analyses use non-parametric Spearman rank correlation to explore the relationship between change from baseline to Week 40 in PET imaging of ^{18}F-DOPA uptake and either VOI coverage or total putamenal coverage after the last test infusion at the end of the healing phase by treatment group for the ITT Primary Population. Correlations are calculated separately for each treatment group. Parameters include change from baseline to Week 40 in ^{18}F-DOPA uptake rate constant for anterior, central and posterior putamen, and caudate nucleus determined by PET, separately for left and right hemispheres, and both VOI coverage as a percentage of total VOI and total putamenal coverage as a percentage of total putamenal volume on the ipsilateral side (ie, left compared with left, right compared with right).

The estimated correlation coefficient, 95% CI, and p-value are tabulated for each analysis and treatment group, and scatterplots are provided.

9.9 Safety Analyses

No imputation is performed for missing safety data other than questionnaire data, as described below.

9.9.1 Study Medication Exposure

Study medication exposure data include number of infusions received and total GDNF exposure in mg, assuming the entire dose was infused at each administration (see definition of total exposure in Section 5.0). These data are presented by treatment group for the Safety Primary Population and Safety Overall Population.

Infusion details per study medication visit include duration of infusion (minutes) and any infusion interruptions/early terminations (yes/no; see definition of duration of infusion in Section 5.0). These data are summarized by treatment group for the Safety Primary Population and Safety Overall Population by visit.

Study medication exposure data, along with infusion details, are listed by study stage, treatment group, and subject.

9.9.2 Adverse Events

9.9.2.1 All Adverse Events

A summary of AEs including the following categories is presented for the Safety Primary Population and Safety Overall Population by treatment group:

- Overall summary of AEs
- TEAEs by MedDRA SOC and PT
- TEAEs experienced by at least 3 subjects in any treatment group by PT (number of subjects and number of events)
- TEAEs experienced by a subject within 7 days of at least 3 infusion visits by PT (number of subjects, mean and range of number of visits per subject)
- TEAEs by MedDRA SOC, PT, and maximum severity
- Serious TEAEs by MedDRA SOC and PT
- TEAEs leading to permanent discontinuation of study medication by MedDRA SOC and PT
- Study medication-related TEAEs by MedDRA SOC and PT
- Serious study medication-related TEAEs by MedDRA SOC and PT
- Device-related TEAEs by MedDRA SOC and PT
- Serious device-related TEAEs by MedDRA SOC and PT

In addition, the following summaries of pre-treatment AEs (see definition in Section 5.0) are presented for the Safety Enrolled Population:

- Pre-treatment AEs by MedDRA SOC and PT
- Serious pre-treatment AEs by MedDRA SOC and PT
- Device-related pre-treatment AEs by MedDRA SOC and PT
- Serious device-related pre-treatment AEs by MedDRA SOC and PT

Except where specified, counting is by subject, not event, and subjects are only counted once within each SOC or PT. Sorting is alphabetically by SOC and then alphabetically for PT.

All AEs (including non-treatment-emergent AEs) are listed by study stage, treatment group, and subject. In addition, pre-treatment AEs in the Safety Enrolled Population are listed by study stage, group, and subject.

9.9.2.2 Adverse Events of Special Interest

Four categories of treatment-emergent AESIs will be analyzed: dyskinesias; falls; adverse changes in mood; and impulsivity (see definitions in Section 5.0). AESIs are presented by category and PT for the Safety Primary Population by treatment group.

AESIs are listed by category, study stage, treatment group, and subject.

9.9.3 Port Symptoms

Evaluation of port symptoms includes the following categories:

- No skin reaction
- Redness with slight swelling
- Redness, moistness and moderate swelling with tissue granulation
- Overt infection

Port symptoms are listed by study stage, treatment group, and subject.

9.9.4 Adverse Changes in MRI Findings

Adverse changes in MRI findings as captured by AE reporting (see definition in Section 5.0) are listed by study stage, treatment group, and subject.

9.9.5 Laboratory Data

Clinical laboratory evaluations including hematology, serum chemistry, and urinalysis are reported for Screening Visit 1 (baseline) and Weeks 4, 16, 28, and 40. Laboratory parameters are listed in Table 4.

Table 4. Laboratory Parameters

Hematology	Serum Chemistry	Urinalysis	Other
RBC count	Alkaline phosphatase	Color	Pregnancy test
Hematocrit	ALT	Appearance	
Hemoglobin	Total bilirubin	pH	
MCH	Creatinine	Glucose	
MCHC	Urea	Ketones	
MCV	eGFR	Nitrite	
Platelet count	Albumin	Microscopy	
WBC count	Glucose		
WBC differential (basophils, eosinophils, lymphocytes, monocytes and neutrophils)	Potassium		
	Sodium		

Laboratory parameters are grouped by panel (hematology, serum chemistry, and urinalysis) and sorted as in Table 4. Units are as shown on the CRF.

For hematology and serum chemistry, summary statistics for values and change from baseline are tabulated at each scheduled visit for the Safety Primary Population by treatment group. Hematology and serum chemistry data are also summarized using shift tables for the Safety Primary Population by treatment group. Using ranking relative to normal range (low, normal, high), these tables compare postbaseline to baseline values. Postbaseline hematology and serum chemistry results rated clinically significant by the investigator are summarized with the direction of significance indicated (high or low). Results for eGFR are listed only. For ALT and total bilirubin, any results recorded in the format <X or >X

are included in tables as a value of X. These results are listed in the original format. Urinalysis parameters are listed only.

Laboratory data including hematology, serum chemistry, and urinalysis are listed by study stage, treatment group, and subject with values outside of the normal range and clinically significant values flagged. Pregnancy test data are included in SDTM datasets only.

9.9.6 Anti-GDNF Antibodies

The data for anti-GDNF antibodies (this section) and plasma GDNF concentrations (Section 9.9.7 below) will not be available until after database lock. In case of a substantial delay, an addendum to the CSR may be written at a later time containing these analyses.

Anti-GDNF binding and neutralizing antibody data are reported for Screening Visit 1 (baseline) and Weeks 4, 16, 28, and 40. Anti-GDNF antibody data are summarized for the Safety Primary Population and Safety Overall Population by treatment group with number and percentage of subjects in each category (positive, negative, or not done) by visit. Summary data are also provided for subjects who are positive at any postbaseline visit and those who are positive at more than one postbaseline visit. If a subject has a repeat sample, then the worse result is used in the analysis. A positive result is considered worse than a negative result.

Anti-GDNF binding and neutralizing antibody data are listed by study stage, treatment group, and subject.

9.9.7 Plasma GDNF Concentrations

Plasma GDNF concentrations are reported for Screening Visit 1 (baseline) and Weeks 4, 16, 28, and 40. Plasma GDNF concentrations are summarized for the Safety Primary Population and Safety Overall Population by treatment group by comparing postbaseline to baseline values using summary statistics for changes from baseline. Values below the limit of quantitation are not included in summary statistics. If a subject has a repeat sample, then the higher result is used in the analysis.

Plasma GDNF concentrations are listed by study stage, treatment group, and subject.

9.9.8 Physical Examination

A physical examination is conducted at screening with a brief physical examination for changes at Week 40. Physical examination data are listed by study stage, treatment group, subject, and body system.

9.9.9 Vital Signs

Vital sign evaluations including pulse (sitting and standing), systolic and diastolic blood pressure (sitting and standing), respiration rate, and temperature are reported for Screening Visit 1 and all test infusion and study medication infusion visits. During infusion visits, repeated assessments are done pre-dose, at various time points during infusion, and after the end of infusion.

Frequency tabulations for vital signs display the number and percentage of subjects with clinically relevant abnormalities during or after infusion. Findings are displayed for all test infusion visits (healing phase visits, interim visits after catheter repositioning, and Week 40 visits) and study medication infusion visits in the Safety Primary Population. The criteria for clinically relevant postbaseline abnormalities are outlined in Table 5.

Vital sign data from infusions with a clinically revelant postbaseline abnormal result are listed by study stage, treatment group, and subject.

Table 5. Clinically Relevant Postbaseline Abnormalities for Vital Signs Parameters

Parameter	Criterion
Temperature	> 38°C and an increase from pre-dose of at least 1°C

Parameter	Criterion
Respiration rate	< 12 or > 20 breaths/min
Pulse	≥ 120 bpm or an increase from pre-dose of > 20 bpm
Pulse	< 50 bpm
Systolic BP	≥ 180 mm Hg or an increase from pre-dose of ≥ 30 mmHg
Systolic BP	< 90 mmHg or a decrease from pre-dose of ≥ 30 mmHg
Diastolic BP	≥ 105 mmHg or an increase from pre-dose of ≥ 20 mmHg
Diastolic BP	< 50 mmHg or a decrease from pre-dose of ≥ 20 mmHg

Pre-dose relates to the pre-infusion value at the respective visit.

9.9.10 Weight

Body weight is reported for Screening Visit 1 and Weeks 0 (baseline), 8, 16, 24, 32, and 40.

Postbaseline weight will be compared to baseline weight using summary statistics for change from baseline for the Safety Primary Population at study medication infusion visits. A two-sample t-test will be performed to detect a difference between treatment groups in the change from baseline for weight.

Weight data are listed by study stage, treatment group, and subject.

9.9.11 Electrocardiogram

ECG data are reported for Screening Visit 1 (baseline) and Week 40. Quantitative parameters are heart rate (beats/min), PR interval (ms), QRS interval (ms), QT interval (ms), and QTc interval (ms). An overall impression is recorded in the CRF as normal or abnormal and, if abnormal, clinically significant yes (specify abnormality) or no in the investigator's judgment. In addition, objective criteria for clinically relevant QTc abnormalities are defined in Table 6.

Table 6. Clinically Relevant Abnormalities for QTc*

Parameter	Criterion
QTc	> 450 ms
QTc	> 500 ms
QTc change from baseline	> 30 ms
QTc change from baseline	> 60 ms

* Based on ICH E14 guideline.

Quantitative ECG data are tabulated for the Safety Primary Population by treatment group, comparing Week 40 to baseline values using summary statistics for changes from baseline.

A categorical table for the Safety Primary Population summarizes the number and percentage of subjects with normal and abnormal ECG results at Week 40 (overall and those judged clinically significant by the investigator) and the number and percentage of subjects with Week 40 QTc abnormalities assessed as clinically relevant according to the criteria in Table 6 (overall and per category).

ECG data, both quantitative parameters and overall impression, are listed by study stage, treatment group, and subject with flags for clinically significant overall ECG impression and clinically relevant QTc abnormalities.

9.9.12 Glasgow Coma Scale

Glasgow Coma Scale is reported for all test infusion and study medication infusion visits. The assessments are performed before infusion, 30 minutes into the infusion, and after completion of infusion. Glasgow Coma Scale items include visual response, verbal ability, and motor skills. Each item has 4-6 possible responses. The possible responses for each item are listed in Table 7. The best possible total score is 15.

Table 7. Glasgow Coma Scale Scoring

Visual Response	Verbal Ability	Motor Skills
1. No eye opening	1. No verbal response	1. No motor response
2. Eye opening to pain	2. Incomprehensible sounds	2. Extension to pain
3. Eye opening to verbal command	3. Inappropriate words	3. Flexion to pain
4. Eyes open spontaneously	4. Confused	4. Withdrawal from pain
	5. Orientated	5. Localizing pain
		6. Obeys commands

Frequency tabulations display the number and percentage of subjects with a total Glasgow Coma Scale score of 15 or less than 15 at any time during or after infusion. Findings are displayed for all test infusion and study medication infusion visits in the Safety Primary Population.

Glasgow Coma Scale results < 15 or missing are listed by study stage, treatment group, and subject.

9.9.13 Questionnaire for Impulsive-Compulsive Disorders

The QUIP-Current-Full, version 1.0, is a self-administered or informant-completed scale that includes 13 questions covering symptoms related to the 4 commonest impulse control disorders in PD (gambling, sex, buying, and eating) as well as other behaviors and problematic use of medication. The number of "Yes" responses is compared to the number required for a positive result per the scoring sheet. Missing individual responses are imputed using LOCF. Each impulse control disorder, other behavior, and problematic used of medication is rated as a positive result (ie, present) or a negative result (ie, not present).

QUIP data are summarized for both subject and informant parameters in a table comparing postbaseline to baseline values using shifts from baseline for the Safety Primary Population by treatment group and responder. QUIP results are summarized for Weeks 0 (baseline; see Section 5.0 definition of baseline), 8, 16, 24, 32, and 40 (frequency modified to every 8 weeks by amendment 4).

QUIP items answered with yes are listed by study stage, treatment group, subject, and responder (informant/subject).

9.9.14 Montreal Cognitive Assessment

The MoCA version 7.1 is a rater-administered cognitive screening tool with 8 components: visuospatial/executive, naming, memory, attention, language, abstraction, delayed recall, and orientation. The total score ranges from 0 to 30, with lower scores representing poorer cognitive function. A total score of 26 or above is considered normal. The MoCA will be analyzed using total score only. Missing individual scores are imputed using LOCF if necessary.

MoCA data are summarized in a table comparing postbaseline to baseline values using summary statistics for changes from baseline for the Safety Primary Population by treatment group. MoCA results are summarized for screening, pre-test infusion (baseline), and Week 40.

MoCA total score data are listed by study stage, treatment group, and subject.

9.9.15 Mattis Dementia Rating Scale

The MDRS version 2 is a rater-administered global scale of cognition including 5 subscales: attention, initiation/perseveration, construction, conceptualization, and memory. Scores range from 0 to 144, with higher scores representing better cognitive function. In PD, scores <123 are associated with some degree of dementia. The age- and education-corrected Mayo Older Adults Normative Studies (MOANS) scaled score (AEMSS) total score ranges from 0 to 20, with higher scores representing better cognitive function. The MDRS will be analyzed using AEMSS total score only as entered in the CRF. Individual missing items are imputed using the average of non-missing scores in each subscale if necessary.

MDRS data are summarized in a table comparing postbaseline to baseline values using summary statistics for changes from baseline for the Safety Primary Population by treatment group. MDRS results are summarized for Screening Visit 1 (baseline) and Week 40.

MDRS AEMSS total score data are listed by study stage, treatment group, and subject.

9.9.16 Stroop Test

The Stroop test is a global scale of reaction time including 4 conditions: color naming, word reading, inhibition, and inhibition/switching. Total time to complete the test in each condition can range from 0 to 999 seconds, with lower time representing better reaction time. The Stroop test will be analyzed separately in each condition.

Stroop test data are summarized in a table comparing postbaseline to baseline values using summary statistics for changes from baseline for the Safety Primary Population by treatment group. Stroop test results are summarized for Screening Visit 2 (baseline) and Week 40.

Stroop test data are listed by study stage, treatment group, and subject.

9.9.17 Frontal Systems Behavioural Scale

The FrSBe is a scale that assesses behavior related to frontal systems damage including 3 subscales: apathy, disinhibition, and executive dysfunction. Higher subscale scores indicate greater pathology. Total score is not analyzed for this study. Individual missing items are imputed using the average of non-missing scores in each subscale.

FrSBe data are summarized in a table comparing postbaseline (Week 40 “after”) to baseline values (Screening Visit 2 “after” values) for each subscale using summary statistics for changes from baseline for the Safety Primary Population by treatment group. FrSBe results are summarized for Screening Visit 2 (before and after [baseline]) and Week 40.

FrSBe test data are listed by study stage, treatment group, and subject.

9.9.18 Deary-Liewald Reaction Time

The Deary-Liewald RT is a computerized measure of simple and four-choice RT. The parameter is the mean reaction time, variance and SD for correct responses for four-choice RT. A shorter reaction time is better.

Deary-Liewald four-choice RT data are summarized in a table comparing postbaseline to baseline values using summary statistics for changes from baseline for the Safety Primary Population by treatment group. Deary-Liewald RT results are summarized for Screening Visit 2 (baseline) and Weeks 12, 24, and 40.

Four-choice RT test data are listed by study stage, treatment group, and subject. Simple RT data are not listed, but are included in SDTM datasets.

9.9.19 Verbal Fluency Assessment

The verbal fluency assessment is a test of verbal functioning in 2 categories: phonemic and semantic. Scores (number of correct words in one minute) range from 0 to 200, with a higher score representing better verbal functioning. Verbal fluency will be analyzed by number of correct responses in each category.

Verbal fluency data are summarized in a table comparing postbaseline to baseline values using summary statistics for changes from baseline for the Safety Primary Population by treatment group. Verbal fluency results are summarized for Screening Visit 2 (baseline) and Week 40. Parameters are phonemic fluency score and semantic fluency score.

Verbal fluency assessment test data are listed by study stage, treatment group.

9.9.20 Beck Depression Inventory

The Beck Depression Inventory (BDI) is a self-administered test which consists of 21 questions that measure the severity of depression. Scores range from 0 to 63, with higher scores representing worse depression. Individual missing items are imputed using the average of non-missing scores in each subscale. The total score is the parameter analyzed.

BDI data are summarized in a table comparing postbaseline to baseline values using summary statistics for changes from baseline for the Safety Primary Population by treatment group. BDI results are summarized for Screening Visit 2 (baseline) and Week 40.

BDI total score data are listed by study stage, treatment group, and subject.

9.9.21 University of Pennsylvania Smell Identification Test

The UPSIT is a self-administered test which can be used to identify and quantitate olfactory dysfunction in PD. The number of correct responses out of 40 total items constitutes a subject's score with interpretations as shown in Table 8. Lower scores represent greater olfactory dysfunction. Individual missing responses are imputed as zeros (ie, incorrect responses).

Table 8. UPSIT Score Interpretation

Test Score (Males)	Test Score (Females)	Olfactory Diagnosis
0 – 5	0 – 5	Probable malingering
6-18	6-18	Total anosmia
19-25	19-25	Severe microsmia
26-29	26-30	Moderate microsmia
30-33	31-34	Mild microsmia
34-40	35-40	Normosmia

UPSIT data are summarized in a table comparing postbaseline to baseline values using summary statistics for changes from baseline for the Safety Primary Population by treatment group. UPSIT results are summarized for Screening Visit 2 (baseline) and Week 40.

UPSIT test data are listed by study stage, treatment group, and subject.

10.0 Validation

PRA's goal is to ensure that each TFL delivery is submitted to the highest level of quality. Our quality control procedures will be documented separately in the study specific quality control plan.

Appendix 1 Glossary of Abbreviations

**aCSF**	Artificial Cerebrospinal Fluid
**ADL**	Activities of Daily Living
**AE**	Adverse Event
**AESI**	Adverse Event of Special Interest
**ALT**	Alanine Aminotransferase
**ANCOVA**	Analysis of Covariance
**ATC**	Anatomical Therapeutic Chemical
**BDI**	Beck Depression Inventory
**BMI**	Body Mass Index
**BP**	Blood Pressure
**CDISC**	Clinical Data Interchange Standards Consortium
**CED**	Convection-Enhanced Delivery
**CI**	Confidence Interval
**COMT**	Catechol-O-methyl transferase
**CRF**	Case Report Form
**CT**	Computed Tomography
**ECG**	Electrocardiogram
**eGFR**	Estimated Glomerular Filtration Rate
**EQ-5D**	EuroQOL 5-dimensional Scale
**FLAIR**	Fluid-attenuated Inversion Recovery
**FrSBe**	Frontal Systems Behavioural Scale
**GDNF**	Glial Cell Line-derived Neurotrophic Factor
**HR**	Heart Rate
**ITT**	Intent-To-Treat
**LOCF**	Last Observation Carried Forward
**MCH**	Mean Corpuscular Hemoglobin
**MCHC**	Mean Corpuscular Hemoglobin Concentration
**MCV**	Mean Corpuscular Volume
**MDRS**	Mattis Dementia Rating Scale
**MedDRA**	Medical Dictionary for Regulatory Activities
**MHRA**	Medicines and Healthcare Products Regulatory Agency
**MMRM**	Mixed-effect Model with Repeated Measures

**MoCA**	Montreal Cognitive Assessment
**MRI**	Magnetic Resonance Imaging
**NART**	National Adult Reading Test
**NBT**	North Bristol National Health System Trust
**NEC**	Not elsewhere classified
**NMSS**	Non-Motor Symptom Assessment Scale
**PD**	Parkinson’s Disease
**PDQ-39**	Parkinson’s Disease Questionnaire-39
**PET**	Positron Emission Tomography
**PRA**	Pharmaceutical Research Associates
**PT**	Preferred Term
**QUIP**	Questionnaire for Impulsive-Compulsive Disorders in Parkinson’s Disease
**RBC**	Red Blood Cells
**RT**	Reaction Time
**SAE**	Serious Adverse Event
**SAP**	Statistical Analysis Plan
**SD**	Standard Deviation
**SDTM**	Standard Data Tabulation Model
**SE**	Standard Error
**SNAQ**	Simplified Nutritional Appetite Questionnaire
**SOC**	System Organ Class
**TEAE**	Treatment-Emergent Adverse Event
**TFL**	Tables, Figures, and Listings
**UPDRS**	Unified Parkinson’s Disease Rating Scale
**UPPS-P**	Impulsiveness Behaviour Scale
**UPSIT**	University of Pennsylvania Smell Identification Test
**VOI**	Volume of Interest
**WBC**	White Blood Cells
**WHODRUG DDE**	World Health Organization Drug Dictionary Enhanced

Appendix 2 Protocol Deviation Guidance

Definitions

A protocol deviation is any change, divergence, or departure from the study design or procedures defined in the protocol.

Major protocol deviations are a subset of protocol deviations that might significantly affect a subject's rights, safety, or well-being or that might significantly affect the completeness, accuracy, and/or reliability of core study data. For example, major protocol deviations might include enrolling subjects in violation of key eligibility criteria designed to ensure a specific subject population or failing to collect data necessary to interpret the primary endpoint, as this may compromise the scientific value of the trial.

Minor deviations are deviations that do not meet the above criteria, i.e. do not impact subject safety or the scientific integrity of the study.

Examples of major and minor protocol violations are provided in the table below.

Major Deviations	Minor Deviations
Key eligibility criteria violated	Visit outside of window
Subject missed more than one study treatment or was given the wrong treatment (treatment other than the randomized treatment) more than once	Protocol required assessment not completed (except for OFF state UPDRS motor score at baseline and/or Week 40)
Subject significantly overdosed	Failure to report SAE in required timeframe
Baseline and/or Week 40 OFF state UPDRS motor score not completed properly	Entire visit not conducted (other than Baseline and Week 40)
Failure to obtain informed consent from subject	Lab sample preparation or storage not adequate (anti-GDNF antibodies and plasma concentration of GDNF)
Subject not randomized prior to study treatment or incorrectly randomized	

As per the study protocol, the primary analysis will be an intention-to-treat (ITT) analysis including all subjects randomized in the Primary Stage (ITT Primary Population). Efficacy analyses will be repeated on the Per-Protocol Population, comprising all subjects in the ITT Primary Population who do not have major protocol deviations.

Process

From study start until January 2015, protocol deviations were documented in file notes by the study staff, and provided to PRA for entry into their Clinical Trials Management System (see Memo to File from Robert Appel dated 03-Jun-2014, RE: Project Plan: Planned Process Deviations process). Starting in January 2015, Case Report Forms (CRFs) were created to capture protocol deviations for entry into the clinical database. Protocol deviations previously captured in file notes will be transcribed to the Protocol Deviation CRF.

Prior to database lock, all protocol deviations will be reviewed to assess the deviation classification (major/minor) and category (categories and guidance are provided in the table below). For this review, the adjudication team will use the list of protocol deviations generated from the clinical database as an input, and the list of patients in the ITT population from the Biostatistician or Analysis Programmer. Final determination of the deviation classification (major or minor) will be made by the Study Sponsor in view of the recommendations made by the adjudication team.

Category	Guidance
Inclusion criteria	Any deviation relating to inclusion criteria not being met
Exclusion criteria	Any deviation relating to exclusion criteria being met
Post-surgery randomization criteria	Any deviation from post-surgery randomization criteria
Randomization	Any incorrect randomization
Study medication (including overdose)	Any deviation relating to the administration of study medication. This includes GDNF/aCSF only and does not include test infusions or medications given for levodopa challenge or PET scans.
Non-study medication	Any deviation relating to medication other than study medication. This includes PET scan medications, concomitant medications, and PD medications.
Study schedule/visit windows	Any deviation relating to an assessment which was completed, but outside the visit window or incorrectly completed. An assessment refers to efficacy, safety or imaging.
Outcome assessment	Any deviation relating to an assessment which was entirely missed. An assessment refers to efficacy, safety or imaging.
Other, specify	Deviations which clearly do not fit in any of the other categories.

Appendix 3 List of MedDRA Preferred Terms for Adverse Events of Special Interest

Preferred Terms Related to Adverse Changes in Mood:

SOC Psychiatric disorders

- Agitation
- Anxiety
- Depressed mood
- Suicidal ideation
- Tearfulness

Preferred Terms Related to Impulsivity:

SOC Psychiatric disorders

- Compulsive shopping
- Hypersexuality
- Impulsive behaviour
- Libido increased
- Obsessive-compulsive disorder

Appendix 4 List of Conversion Factors for the Calculation of Levodopa and Levodopa Equivalent Doses

PD Medication	Conversion Factor
**Levodopa Preparations**	
Immediate release preparations without COMT inhibition	1.0
Immediate release preparations with entacapone	1.33
Immediate release preparations with tolcapone	1.5
Controlled release preparations	0.75
Levodopa/carbidopa (Duodopa)	1.11
**MAO-B Inhibitors**	
Selegiline oral	10
Selegiline sublingual	80
Rasagiline	100
**Dopamine Agonists**	
Ropinirole immediate release	20
Ropinirole long acting	20
Pramipexole immediate release (base)	140
Pramipexole immediate release (salt)	100
Pramipexole long acting (base)	140
Pramipexole long acting (salt)	100
Cabergoline	70
Rotigotine	30
Piribedil	1
Apomorphine	10
Bromocriptine	10
Pergolide	100
Lisuride	100
Dihydroergocryptine (DHEC)	5
**Other**	
Amantadine	1

Note: COMT inhibitor doses are not included in the calculation of levodopa or levodopa equivalent doses. They contribute to the levodopa or levodopa equivalent dose by modifying the dose of concurrently administered levodopa.

Appendix 5 List of Post-Text Tables, Figures and Listings

16.1 Demographic Data Tables 62
Table 16.1.1.1 Subject Populations - All Enrolled Subjects 62
Table 16.1.1.2.1 Subject Disposition - ITT Primary Population 63
Table 16.1.1.2.2 Subject Disposition - ITT Overall Population 64
Table 16.1.2.1 Protocol Deviations - ITT Primary Population 65
Table 16.1.3.1 Demographic Characteristics - ITT Primary Population 66
Table 16.1.3.2 Demographic Characteristics - ITT Overall Population 68
Table 16.1.4.1 Parkinson's Disease History at Screening - ITT Primary Population 69
Table 16.1.4.2 Parkinson's Disease History at Screening - ITT Overall Population 71
Table 16.1.4.3 Impulsiveness Behaviour Scale (UPPS-P) at Screening - ITT Primary Population 72
Table 16.1.5.1 General Medical History Resolved at Screening by System Organ Class and Preferred Term - ITT Primary Population 73
Table 16.1.5.2 General Medical History Current/Active at Screening by System Organ Class and Preferred Term - ITT Primary Population 74
Table 16.1.6.1 Prior Medications by WHO Drug Dictionary ATC Class and Preferred Name - ITT Primary Population 75
Table 16.1.6.2.1 Concomitant Parkinson's Disease Medications by WHO Drug Dictionary ATC Class and Preferred Name - ITT Primary Population 76
Table 16.1.6.2.2 Other Concomitant Medications by WHO Drug Dictionary ATC Class and Preferred Name - ITT Primary Population 76
Table 16.1.7 Catheter Placement Surgery Trajectories - ITT Primary Population 77
Table 16.1.8 Catheter Positioning Accuracy as Determined by Post-Surgery CT - ITT Primary Population 78
Table 16.1.9 Contrast-Enhanced Test Infusions With T1-weighted MRI Prior to Start of Study Medication - ITT Primary Population 79
16.2 Efficacy Data Tables 80
16.2.1 UPDRS Primary Efficacy Tables 80
Table 16.2.1.1.1 Primary Efficacy Endpoint - OFF State UPDRS Motor Score (Part III): Change from Baseline to Week 40 – MMRM, ITT Primary Population 80
Table 16.2.1.1.2 Sensitivity Analysis - OFF State UPDRS Motor Score (Part III): Change from Baseline to Week 40 – MMRM, ITT Overall Population 81
Table 16.2.1.1.3 Sensitivity Analysis - OFF State UPDRS Motor Score (Part III): Change from Baseline to Week 40 – MMRM, Per-Protocol Population 81
Table 16.2.1.2 Sensitivity Analysis - OFF State UPDRS Motor Score (Part III): Change from Baseline to Week 40 – LOCF ANCOVA, ITT Primary Population 82
Table 16.2.1.3 Sensitivity Analysis - OFF State UPDRS Motor Score (Part III): Change from Baseline to Week 40 – Observed Data ANCOVA, ITT Primary Population 83
Table 16.2.1.4 Sensitivity Analysis - OFF State UPDRS Motor Score (Part III) by Visit: Change from Baseline – Observed Data ANCOVA, ITT Primary Population 84
Table 16.2.1.5 Sensitivity Analysis - OFF State UPDRS Motor Score (Part III): Change from Screening to Week 40 – MMRM, ITT Primary Population 86
Table 16.2.1.6 Sensitivity Analysis - OFF State UPDRS Motor Score (Part III): Change from Baseline to Week 40 - MMRM – Excluding Subjects with Vertical Catheter Trajectory, ITT Primary Population 86

Table 16.2.2.1.1 ON State UPDRS Motor Score (Part III): Change from Baseline to Week 40 – MMRM, ITT Primary Population 87
Table 16.2.2.1.2 ON State UPDRS Motor Score (Part III): Change from Baseline to Week 40 – MMRM, ITT Overall Population 87
Table 16.2.2.1.3 ON State UPDRS Motor Score (Part III): Change from Baseline to Week 40 – MMRM, Per-Protocol Population 87
Table 16.2.2.1.4 ON State UPDRS Motor Score (Part III) by Visit: Change from Baseline – Observed Data ANCOVA, ITT Primary Population 87
Table 16.2.2.2.1 OFF State UPDRS ADL Score (Part II): Change from Baseline to Week 40 – MMRM, ITT Primary Population 87
Table 16.2.2.2.2 OFF State UPDRS ADL Score (Part II): Change from Baseline to Week 40 – MMRM, ITT Overall Population 87
Table 16.2.2.2.3 OFF State UPDRS ADL Score (Part II): Change from Baseline to Week 40 – MMRM, Per-Protocol Population 87
Programming Note: Delete footnote regarding subject 45. 87
Table 16.2.2.2.4 OFF State UPDRS ADL Score (Part II) by Visit: Change from Baseline – Observed Data ANCOVA, ITT Primary Population 87
Table 16.2.2.3.1 ON State UPDRS ADL Score (Part II): Change from Baseline to Week 40 – MMRM, ITT Primary Population 87
Table 16.2.2.3.2 ON State UPDRS ADL Score (Part II): Change from Baseline to Week 40 – MMRM, ITT Overall Population 87
Table 16.2.2.3.3 ON State UPDRS ADL Score (Part II): Change from Baseline to Week 40 – MMRM, Per-Protocol Population 87
Table 16.2.2.3.4 ON State UPDRS ADL Score (Part II) by Visit: Change from Baseline – Observed Data ANCOVA, ITT Primary Population 88
Table 16.2.2.4.1 OFF State UPDRS Total Score: Change from Baseline to Week 40 – MMRM, ITT Primary Population 89
Table 16.2.2.4.2 OFF State UPDRS Total Score: Change from Baseline to Week 40 – MMRM, ITT Overall Population 89
Table 16.2.2.4.3 OFF State UPDRS Total Score: Change from Baseline to Week 40 – MMRM, Per-Protocol Population 89
Table 16.2.2.4.4 ON State UPDRS Total Score: Change from Baseline to Week 40 – MMRM, ITT Primary Population 89
Table 16.2.2.4.5 ON State UPDRS Total Score: Change from Baseline to Week 40 – MMRM, ITT Overall Population 89
Table 16.2.2.4.6 ON State UPDRS Total Score: Change from Baseline to Week 40 – MMRM, Per-Protocol Population 89
Table 16.2.2.5 UPDRS Mentation, Behavior, and Mood Score (Part I): Change from Baseline to Week 40 – MMRM, ITT Primary Population 89
Table 16.2.2.6 UPDRS Complications of Therapy Score (Part IV): Change from Baseline to Week 40 – MMRM, ITT Primary Population 89
16.2.3 PD DIARY SECONDARY EFFICACY TABLES 90
Table 16.2.3.1 Motor Fluctuation Diary Ratings by Visit: Change from Baseline – MMRM, ITT Primary Population 90
Table 16.2.3.2 Motor Fluctuation Diary Ratings by Visit: Change from Baseline – MMRM, ITT Overall Population 92

Table 16.2.3.3 Motor Fluctuation Diary Ratings by Visit: Change from Baseline – MMRM, Per-Protocol Population 92
16.2.4 SUPPLEMENTARY EFFICACY TABLES 93
Table 16.2.4.1 OFF and ON State Timed Walking Test by Visit: Change from Baseline – MMRM, ITT Primary Population 93
Table 16.2.4.2 OFF and ON State Timed Tapping Test by Visit: Change from Baseline – MMRM, ITT Primary Population 94
Table 16.2.4.3 NMSS Score by Visit: Change from Baseline – MMRM, ITT Primary Population 95
Table 16.2.4.4 PDQ-39 Score: Change from Baseline to Week 40 – Observed Data ANCOVA, ITT Primary Population 96
Table 16.2.4.5.1 EQ-5D Questionnaire: Baseline and Week 40 – Observed Data, ITT Primary Population 97
Table 16.2.4.5.2 EQ-5D Visual Analog Scale: Change from Baseline to Week 40 – Observed Data ANCOVA, ITT Primary Population 98
Table 16.2.4.6 SNAQ Score: Change from Baseline to Week 40 – Observed Data ANCOVA, ITT Primary Population 98
Table 16.2.4.7 Total Daily Levodopa Dose (mg): Change From Baseline to Week 40 – Observed Data ANCOVA, ITT Primary Population 99
Table 16.2.4.8 Total Daily Levodopa Equivalent Dose (mg): Change From Baseline to Week 40 – Observed Data ANCOVA, ITT Primary Population 100
16.3 IMAGING TABLES 101
Table 16.3.1 Volume of Distribution of Infusate as Determined by Contrast-Enhanced T1-Weighted MRI: Change from Baseline to Week 40 – Observed Data ANCOVA, ITT Primary Population 101
Table 16.3.2.1 Volume of Interest Coverage as Determined by Contrast-Enhanced T1-Weighted MRI: Change from Baseline to Week 40 – Observed Data ANCOVA, ITT Primary Population 101
Table 16.3.2.2 Total Putamenal Coverage as Determined by Contrast-Enhanced T1-Weighted MRI: Change from Baseline to Week 40 – Observed Data ANCOVA, ITT Primary Population 101
Table 16.3.3.1 ^{18}F-DOPA Uptake as Determined by PET Scan: Change from Baseline to Week 40 – Observed Data ANCOVA, ITT Primary Population 101
Table 16.3.3.2 ^{18}F-DOPA Uptake as Determined by PET Scan: Change from Baseline to Week 40 – Observed Data ANCOVA, ITT Overall Population 101
Table 16.3.3.3 ^{18}F-DOPA Uptake as Determined by PET Scan: Change from Baseline to Week 40 – Observed Data ANCOVA, Per-Protocol Population 101
Table 16.3.3.4 ^{18}F-DOPA Uptake as Determined by PET Scan: Change from Baseline to Week 12 and Week 40 – Observed Data ANCOVA, ITT Pilot Population 102
Table 16.3.4.1 Correlation Analyses of Percentage Change from Baseline to Week 40 in OFF State UPDRS Motor Score (Part III) to Volume of Interest Coverage and Total Putamenal Coverage at Baseline as Determined by Contrast-Enhanced T1-Weighted MRI – ITT Primary Population 103
Table 16.3.4.2 Correlation Analysis of Change from Baseline to Week 40 in NMSS Total Score to Total Putamenal Coverage at Baseline as Determined by Contrast-Enhanced T1-Weighted MRI – ITT Primary Population 104
Table 16.3.4.3 Correlation Analysis of Percentage Change from Baseline to Week 40 in OFF State UPDRS Motor Score (Part III) to Change From Baseline to Week 40 in ^{18}F-DOPA Uptake as Determined by PET Scan - ITT Primary Population 104

Table 16.3.4.4 Correlation Analyses of Change from Baseline to Week 40 in ^{18}F-DOPA Uptake as Determined by PET Scan to Volume of Interest Coverage at Baseline as Determined by Contrast-Enhanced T1-Weighted MRI – ITT Primary Population 104
Table 16.3.4.5 Correlation Analyses of Change from Baseline to Week 40 in ^{18}F-DOPA Uptake as Determined by PET Scan to Total Putamenal Coverage at Baseline as Determined by Contrast-Enhanced T1-Weighted MRI – ITT Primary Population 104
16.4 Safety Data Tables 105
16.4.1 Exposure Data Tables 105
Table 16.4.1.1.1 Exposure to Study Medication - Safety Primary Population 105
Table 16.4.1.1.2 Exposure to Study Medication - Safety Overall Population 106
Table 16.4.1.2.1 Study Medication Infusion Details by Visit - Safety Primary Population 107
Table 16.4.1.2.2 Study Medication Infusion Details by Visit - Safety Overall Population 108
16.4.2 Adverse Event Tables 109
Table 16.4.2.1.1 Overall Summary of Adverse Events - Safety Primary Population 109
Table 16.4.2.1.2 Overall Summary of Adverse Events - Safety Overall Population 110
Table 16.4.2.2.1 Treatment-Emergent Adverse Events by System Organ Class and Preferred Term - Safety Primary Population 111
Table 16.4.2.2.2 Treatment-Emergent Adverse Events by System Organ Class and Preferred Term - Safety Overall Population 112
Table 16.4.2.3.1 Treatment-Emergent Adverse Events Experienced by at Least 3 Subjects in Any Treatment Group by Preferred Term - Safety Primary Population 113
Table 16.4.2.3.2 Treatment-Emergent Adverse Events Experienced by at Least 3 Subjects in Any Treatment Group by Preferred Term - Safety Overall Population 114
Table 16.4.2.4.1 Treatment-Emergent Adverse Events Experienced by a Subject within 7 Days of at Least 3 Infusion Visits by Preferred Term - Safety Primary Population 115
Table 16.4.2.4.2 Treatment-Emergent Adverse Events Experienced by a Subject within 7 Days of at Least 3 Infusion Visits by Preferred Term - Safety Overall Population 116
Table 16.4.2.5.1 Treatment-Emergent Adverse Events by System Organ Class, Preferred Term, and Maximum Severity - Safety Primary Population 117
Table 16.4.2.5.2 Treatment-Emergent Adverse Events by System Organ Class, Preferred Term, and Maximum Severity - Safety Overall Population 118
Table 16.4.2.6.1 Serious Treatment-Emergent Adverse Events by System Organ Class and Preferred Term - Safety Primary Population 118
Table 16.4.2.6.2 Serious Treatment-Emergent Adverse Events by System Organ Class and Preferred Term - Safety Overall Population 118
Table 16.4.2.7.1 Treatment-Emergent Adverse Events Leading to Permanent Discontinuation of Study Medication by System Organ Class and Preferred Term - Safety Primary Population 118
Table 16.4.2.7.2 Treatment-Emergent Adverse Events Leading to Permanent Discontinuation of Study Medication by System Organ Class and Preferred Term - Safety Overall Population 118
Table 16.4.2.8.1 Study Medication-Related Treatment-Emergent Adverse Events by System Organ Class and Preferred Term - Safety Primary Population 119
Table 16.4.2.8.2 Study Medication-Related Treatment-Emergent Adverse Events by System Organ Class and Preferred Term - Safety Overall Population 119
Table 16.4.2.9.1 Serious Study Medication-Related Treatment-Emergent Adverse Events by System Organ Class and Preferred Term - Safety Primary Population 119
Table 16.4.2.9.2 Serious Study Medication-Related Treatment-Emergent Adverse Events by System Organ Class and Preferred Term - Safety Overall Population 119

Table 16.4.2.10.1 Device-Related Treatment-Emergent Adverse Events by System Organ Class and Preferred Term - Safety Primary Population 119
Table 16.4.2.10.2 Device-Related Treatment-Emergent Adverse Events by System Organ Class and Preferred Term - Safety Overall Population 119
Table 16.4.2.11.1 Serious Device-Related Treatment-Emergent Adverse Events by System Organ Class and Preferred Term - Safety Primary Population 119
Table 16.4.2.11.2 Serious Device-Related Treatment-Emergent Adverse Events by System Organ Class and Preferred Term - Safety Overall Population 119
Table 16.4.2.12 Pre-Treatment Adverse Events by System Organ Class and Preferred Term - Safety Enrolled Population 120
Table 16.4.2.13 Serious Pre-Treatment Adverse Events by System Organ Class and Preferred Term - Safety Enrolled Population 121
Table 16.4.2.14 Device-Related Pre-Treatment Adverse Events by System Organ Class and Preferred Term - Safety Enrolled Population 121
Table 16.4.2.15 Serious Device-Related Pre-Treatment Adverse Events by System Organ Class and Preferred Term - Safety Enrolled Population 121
Table 16.4.2.16.1 Treatment-Emergent Adverse Events of Special Interest by Preferred Term - Safety Primary Population 122
Table 16.4.2.16.2 Treatment-Emergent Adverse Events of Special Interest by Preferred Term - Safety Overall Population 123
16.4.3 LABORATORY TABLES 124
Table 16.4.3.1.1 Hematology Results by Visit - Safety Primary Population 124
Table 16.4.3.1.2 Shift Summary of Hematology Results by Reference Range and Visit - Safety Primary Population 125
Table 16.4.3.1.3 Clinically Significant Postbaseline Hematology Results - Safety Primary Population 126
Table 16.4.3.2.1 Serum Chemistry Results by Visit - Safety Primary Population 127
Table 16.4.3.2.2 Shift Summary of Serum Chemistry Results by Reference Range and Visit - Safety Primary Population 127
Table 16.4.3.2.3 Clinically Significant Postbaseline Serum Chemistry Results - Safety Primary Population 127
16.4.4 ANTI-GDNF ANTIBODY TABLES 128
Table 16.4.4.1.1 Anti-GDNF Binding Antibodies by Visit - Safety Primary Population 128
Table 16.4.4.1.2 Anti-GDNF Binding Antibodies by Visit - Safety Overall Population 129
Table 16.4.4.2.1 Anti-GDNF Neutralizing Antibodies by Visit Safety Primary Population 129
Table 16.4.4.2.2 Anti-GDNF Neutralizing Antibodies by Visit - Safety Overall Population 129
16.4.5 PLASMA GDNF CONCENTRATION TABLES 130
Table 16.4.5.1 Plasma GDNF Concentrations by Visit - Safety Primary Population 130
Table 16.4.5.2 Plasma GDNF Concentrations by Visit - Safety Overall Population 130
16.4.6 VITAL SIGN TABLE 131
Table 16.4.6 Clinically Relevant Postbaseline Abnormalities in Vital Sign Results - Safety Primary Population 131
16.4.7 BODY WEIGHT TABLE 132
Table 16.4.7 Body Weight by Visit - Safety Primary Population 132
16.4.8 ELECTROCARDIOGRAM TABLES 133
Table 16.4.8.1 Electrocardiogram Results at Week 40 - Safety Primary Population 133

Table 16.4.8.2 Summary of Electrocardiogram Results at Week 40 - Safety Primary Population 134
16.4.9 Glasgow Coma Scale Table 135
Table 16.4.9 Glasgow Coma Scale Score = 15 or < 15 During or After Infusion by Visit and Time Point - Safety Primary Population 135
16.4.10 Other Safety Tables 136
Table 16.4.10.1 QUIP by Visit - Safety Primary Population 136
Table 16.4.10.2 MoCA at Week 40 - Safety Primary Population 138
Table 16.4.10.3 MDRS at Week 40 - Safety Primary Population 138
Table 16.4.10.4 Stroop Test at Week 40, Safety Primary Population 138
Table 16.4.10.5 FrSBe at Week 40 - Safety Primary Population 138
Table 16.4.10.6 Deary-Liewald Four-Choice Reaction Time by Visit - Safety Primary Population 138
Table 16.4.10.7 Verbal Fluency Assessment at Week 40 - Safety Primary Population 138
Table 16.4.10.8 BDI at Week 40 - Safety Primary Population 138
Table 16.4.10.9 UPSIT at Week 40 - Safety Primary Population 139
16.5 Figures 140
Figure 16.5.1.1 OFF State UPDRS Motor Score (Part III): Percentage Change Over Time - ITT Primary Population 140
Figure 16.5.1.2 ON State UPDRS Motor Score (Part III): Percentage Change Over Time - ITT Primary Population 141
Figure 16.5.1.3 OFF State UPDRS Total Score: Percentage Change Over Time - ITT Primary Population 141
Figure 16.5.1.4 ON State UPDRS Total Score: Percentage Change Over Time - ITT Primary Population 141
Figure 16.5.2.1 Motor Fluctuation Diary Total OFF Time Per Day (Hours): Change Over Time - ITT Primary Population 142
Figure 16.5.2.2 Motor Fluctuation Diary Total Good-Quality ON Time Per Day (Hours): Change Over Time - ITT Primary Population 142
Figure 16.5.3.1 OFF State Timed Tapping Test (Left Hand): Change Over Time - ITT Primary Population 142
Figure 16.5.3.2 OFF State Timed Tapping Test (Right Hand): Change Over Time - ITT Primary Population 142
Figure 16.5.3.3 ON State Timed Tapping Test (Left Hand): Change Over Time - ITT Primary Population 142
Figure 16.5.3.4 ON State Timed Tapping Test (Right Hand): Change Over Time - ITT Primary Population 142
Figure 16.5.4.1 OFF State UPDRS Motor Score (Part III): Subject Scores Over Time - ITT Primary Population 143
Figure 16.5.5.1 OFF State Timed Tapping Test (Left Hand): Subject Values Over Time - ITT Primary Population 144
Figure 16.5.5.2 OFF State Timed Tapping Test (Right Hand): Subject Values Over Time - ITT Primary Population 144
Figure 16.5.5.3 ON State Timed Tapping Test (Left Hand): Subject Values Over Time - ITT Primary Population 144
Figure 16.5.5.4 ON State Timed Tapping Test (Right Hand): Subject Values Over Time - ITT Primary Population 144

Figure 16.5.6.1 Correlation Analysis of Percentage Change from Baseline to Week 40 in OFF State UPDRS Motor Score (Part III) to Volume of Interest Coverage at Baseline as Determined by Contrast-Enhanced T1-Weighted MRI - ITT Primary Population 145
Figure 16.5.6.2 Correlation Analysis of Percentage Change from Baseline to Week 40 in OFF State UPDRS Motor Score (Part III) to Total Putamenal Coverage at Baseline as Determined by Contrast-Enhanced T1-Weighted MRI - ITT Primary Population 146
Figure 16.5.6.3 Correlation Analysis of Change From Baseline to Week 40 in NMSS Total Score to Total Putamenal Coverage at Baseline as Determined by Contrast-Enhanced T1-Weighted MRI - ITT Primary Population 147
Figure 16.5.6.4 Correlation Analysis of Percentage Change from Baseline to Week 40 in OFF State UPDRS Motor Score (Part III) to Change From Baseline to Week 40 in ^{18}F-DOPA Uptake as Determined by PET Scan - ITT Primary Population 148
Figure 16.5.6.5 Correlation Analysis of Change From Baseline to Week 40 in ^{18}F-DOPA Uptake as Determined by PET Scan to Volume of Interest Coverage at Baseline as Determined by Contrast-Enhanced T1-Weighted MRI - ITT Primary Population 148
Figure 16.5.6.6 Correlation Analysis of Change From Baseline to Week 40 in ^{18}F-DOPA Uptake as Determined by PET Scan to Total Putamenal Coverage at Baseline as Determined by Contrast-Enhanced T1-Weighted MRI - ITT Primary Population 148
17 DATA LISTINGS 149
17.2.1 BASELINE LISTINGS 149
Listing 17.2.1.1 Randomization Assignments - ITT Overall Population 149
Listing 17.2.1.2.1 Study Completion Status - ITT Overall Population 150
Listing 17.2.1.2.2 Study Completion Status - Subjects Enrolled but Not Randomized 151
Listing 17.2.1.2.3 Study Completion Status - Subjects with Initial Informed Consent but Not Enrolled 152
Listing 17.2.1.3.1 Major Protocol Deviations - ITT Overall Population 153
Listing 17.2.1.3.2 Minor Protocol Deviations - ITT Overall Population 154
Listing 17.2.1.4 Subject Populations - ITT Overall Population 155
Listing 17.2.1.5.1 Demographic Characteristics - ITT Overall Population 156
Listing 17.2.1.5.2 Parkinson's Disease History at Screening - ITT Overall Population 157
Listing 17.2.1.5.3 Impulsiveness Behaviour Scale (UPPS-P) at Screening - ITT Overall Population 158
Listing 17.2.1.6 General Medical History - ITT Overall Population 159
Listing 17.2.1.7 Prior and Concomitant Medications – ITT Overall Population 160
Listing 17.2.1.8 Catheter Trajectory - ITT Overall Population 161
Listing 17.2.1.9 Catheter Positioning Accuracy by Surgery - ITT Overall Population 162
Listing 17.2.1.10 Test Infusion Data by Visit - ITT Overall Population 163
Listing 17.2.1.11 Test Infusion Catheter Interruptions/Early Terminations by Visit - ITT Overall Population 164
17.2.2 EFFICACY LISTINGS 165
Listing 17.2.2.1 OFF and ON state UPDRS Scores by Visit - ITT Overall Population 165
Listing 17.2.2.2 PD Motor Fluctuation Diary Ratings by Visit - ITT Overall Population 167
Listing 17.2.2.3.1 OFF and ON State Timed Walking Test by Visit - ITT Overall Population 168
Listing 17.2.2.3.2 OFF and ON State Timed Tapping Test by Visit - ITT Overall Population 169
Listing 17.2.2.4 NMSS Scores by Visit - ITT Overall Population 170
Listing 17.2.2.5 PDQ-39 Scores at Screening and Week 40 - ITT Overall Population 170
Listing 17.2.2.6 EQ-5D Scores at Screening and Week 40 - ITT Overall Population 170

Listing 17.2.2.7 SNAQ Scores at Screening and Week 40 - ITT Overall Population ... 170
Listing 17.2.2.8.1 Levodopa and Levodopa Equivalent Medication Actual Total Daily Doses - ITT Overall Population ... 171
Listing 17.2.2.8.2 Effective Levodopa and Levodopa Equivalent Medication Total Daily Doses - ITT Overall Population ... 173
17.2.3 IMAGING LISTINGS ... 174
Listing 17.2.3.1 Volume of Distribution, Volume of Interest Coverage, and Total Putamenal Coverage as Determined by Contrast-Enhanced T1-Weighted MRI by Visit - ITT Overall Population ... 174
Listing 17.2.3.2 PET ^{18}F-DOPA Uptake by Visit - ITT Overall Population ... 174
17.2.4 SAFETY LISTINGS ... 175
Listing 17.2.4.1.1 Exposure to Study Medication - Safety Overall Population ... 175
Listing 17.2.4.1.2 Study Medication Infusion Data by Visit - Safety Overall Population ... 176
Listing 17.2.4.1.3 Study Medication Infusion Catheter Interruptions/Early Terminations by Visit - Safety Overall Population ... 177
Listing 17.2.4.2 Adverse Events - Safety Overall Population ... 178
Listing 17.2.4.3 Pre-Treatment Adverse Events - Safety Enrolled Population ... 179
Listing 17.2.4.4 Adverse Events of Special Interest: Dyskinesias - Safety Overall Population ... 179
Listing 17.2.4.5 Adverse Events of Special Interest: Falls - Safety Overall Population ... 179
Listing 17.2.4.6 Adverse Events of Special Interest: Adverse Changes in Mood - Safety Overall Population ... 179
Listing 17.2.4.7 Adverse Events of Special Interest: Impulsivity - Safety Overall Population ... 179
Listing 17.2.4.8 Port Symptoms by Visit - Safety Overall Population ... 180
Listing 17.2.4.9 Adverse Changes in MRI Findings - Safety Overall Population ... 181
Listing 17.2.4.10 Hematology Results by Visit - Safety Overall Population ... 182
Listing 17.2.4.11 Serum Chemistry Results by Visit - Safety Overall Population ... 183
Listing 17.2.4.12 Urinalysis Results by Visit - Safety Overall Population ... 183
Listing 17.2.4.13 Anti-GDNF Antibodies - Safety Overall Population ... 184
Listing 17.2.4.14 Plasma GDNF Concentration Results by Visit - Safety Overall Population ... 185
Listing 17.2.4.15 Physical Examination at Screening and Week 40 - Safety Overall Population . 186
Listing 17.2.4.16 Vital Signs from Infusions with a Clinically Revelant Postbaseline Abnormal Result by Visit and Time Point - Safety Overall Population ... 188
Listing 17.2.4.17 Weight by Visit - Safety Overall Population ... 189
Listing 17.2.4.18 Electrocardiogram Results at Screening and Week 40 – Continuous Parameters, Safety Overall Population ... 189
Listing 17.2.4.19 Electrocardiogram Results at Screening and Week 40 – Overall Impression, Safety Overall Population ... 190
Listing 17.2.4.20 Glasgow Coma Scale Results < 15 by Visit - Safety Overall Population ... 191
Listing 17.2.4.21 QUIP Items Answered with Yes by Visit - Safety Overall Population ... 192
Listing 17.2.4.22 MoCA Total Score by Visit - Safety Overall Population ... 193
Listing 17.2.4.23 MDRS AEMSS Total Score by Visit - Safety Overall Population ... 194
Listing 17.2.4.24 Stroop Test by Visit - Safety Overall Population ... 195
Listing 17.2.4.25 FrSBe by Visit - Safety Overall Population ... 196
Listing 17.2.4.26 Deary-Liewald Four-Choice Reaction Time by Visit - Safety Overall Population ... 197
Listing 17.2.4.27 Verbal Fluency Assessment by Visit - Safety Overall Population ... 198
Listing 17.2.4.28 BDI by Visit - Safety Overall Population ... 199

Listing 17.2.4.29 UPSIT by Visit - Safety Overall Population 200

Appendix 6 Shells for Post-Text Tables, Figures and Listings

16.1 Demographic Data Tables

Table 16.1.1.1 Subject Populations - All Enrolled Subjects

Population	GDNF	Placebo	Total
Pilot Stage			
Subjects enrolled [n]			xx
Subjects undergoing surgery [n]			xx
Subjects operated but not randomized [n]			xx
Subjects randomized (ITT Pilot Population) [n]	xx	xx	xx
Subjects randomized but not treated [n (%)[a]]	xx (xx.x)	xx (xx.x)	xx (xx.x)
Subjects treated (Safety Pilot Population) [n[b]]	xx	xx	xx
Primary Stage			
Subjects enrolled [n]			xx
Subjects undergoing surgery [n]			xx
Subjects operated but not randomized [n]			xx
Subjects randomized (ITT Primary Population) [n]	xx	xx	xx
Per-Protocol Population [n (%)[a]]	xx (xx.x)	xx (xx.x)	xx (xx.x)
Subjects randomized but not treated [n (%)[a]]	xx (xx.x)	xx (xx.x)	xx (xx.x)
Subjects treated (Safety Primary Population) [n[b]]	xx	xx	xx
Overall (Pilot + Primary Stage)			
Subjects enrolled [n]			xx
Subjects undergoing surgery [n]			xx
Subjects operated but not randomized [n]			xx
Subjects randomized (ITT Overall Population) [n]	xx	xx	xx
Subjects randomized but not treated [n (%)[a]]	xx (xx.x)	xx (xx.x)	xx (xx.x)
Subjects treated (Safety Overall Population) [n[b]]	xx	xx	xx

a Percent of randomized subjects in each stage and overall.
b Percentage not included because subjects are grouped according to treatment actually received and not randomized treatment for Safety Populations.
Note: The Per-Protocol Population is defined as all ITT Primary Population subjects with no major protocol deviations.
Source: Listing xx.x.xx, Dataset: [NAME], Program: xxxxxx.sas, Output: T_16_1_1_1_XXXXX.rtf, Generated on: DDMONYYYY HH:MM
Page 1 of 1

Table 16.1.1.2.1 Subject Disposition - ITT Primary Population

Variable	GDNF (N = XXX) n (%)	Placebo (N = XXX) n (%)	Total (N = XXX) n (%)
Completed study	xx (xx.x)	xx (xx.x)	xx (xx.x)
Discontinued study (primary reason for early termination)	xx (xx.x)	xx (xx.x)	xx (xx.x)
Death	xx (xx.x)	xx (xx.x)	xx (xx.x)
Adverse event(s)	xx (xx.x)	xx (xx.x)	xx (xx.x)
Withdrawal by subject	xx (xx.x)	xx (xx.x)	xx (xx.x)
Protocol violation(s)	xx (xx.x)	xx (xx.x)	xx (xx.x)
Lost to follow-up	xx (xx.x)	xx (xx.x)	xx (xx.x)
Physician decision	xx (xx.x)	xx (xx.x)	xx (xx.x)
Other	xx (xx.x)	xx (xx.x)	xx (xx.x)
Missing	xx (xx.x)	xx (xx.x)	xx (xx.x)

Note: Reasons for discontinuation are based on the End of Study CRF page.
Source: Listing xx.x.xx, Dataset: [NAME], Program: xxxxxx.sas, Output: T_16_1_1_1_XXXXX.rtf, Generated on: DDMONYYYY HH:MM
Page 1 of 1

Programming Note: Sort in order as on CRF. Include all discontinuation reasons on CRF (even if no subjects discontinued for that reason).

Table 16.1.1.2.2 Subject Disposition - ITT Overall Population

Programming Note: Repeat table for different population.

Table 16.1.2.1 Protocol Deviations - ITT Primary Population

**Category**<br>**Deviation (brief description)**	**GDNF (N = XXX) n (%)**	**Placebo (N = XXX) n (%)**	**Total (N = XXX) n (%)**
Subjects with at least one major protocol deviation	xx (xx.x)	xx (xx.x)	xx (xx.x)
[Category 1]	xx (xx.x)	xx (xx.x)	xx (xx.x)
[Deviation 1]	xx (xx.x)	xx (xx.x)	xx (xx.x)
[Deviation 2]	xx (xx.x)	xx (xx.x)	xx (xx.x)
[Category 2]	xx (xx.x)	xx (xx.x)	xx (xx.x)
[Deviation 1]	xx (xx.x)	xx (xx.x)	xx (xx.x)
[Deviation 2]	xx (xx.x)	xx (xx.x)	xx (xx.x)
…			
Subjects with at least one minor protocol deviation	xx (xx.x)	xx (xx.x)	xx (xx.x)
[Category 1]	xx (xx.x)	xx (xx.x)	xx (xx.x)
[Category 2]	xx (xx.x)	xx (xx.x)	xx (xx.x)
…			

Note: For each category and deviation, subjects are included only once, even if they experienced multiple events in that category or deviation.
Source: Listing xx.x.xx, Dataset: [NAME], Program: xxxxxx.sas, Output: T_16_1_1_1_XXXXX.rtf, Generated on: DDMONYYYY HH:MM
Page 1 of 1

Programming Note: Within category, sort should be by decreasing frequency of deviations in Total group.

Table 16.1.3.1 Demographic Characteristics - ITT Primary Population

Variable	GDNF (N = XXX)	Placebo (N = XXX)	Total (N = XXX)
Age (years)			
N	xx	xx	xx
Mean (SD)	xx.x (xx.xx)	xx.x (xx.xx)	xx.x (xx.xx)
Median	xx.x	xx.x	xx.x
Min, Max	xx, xx	xx, xx	xx, xx
Age group [n (%)]			
< 65 years	xx (xx.x)	xx (xx.x)	xx (xx.x)
≥ 65 years	xx (xx.x)	xx (xx.x)	xx (xx.x)
Sex [n (%)]			
Female	xx (xx.x)	xx (xx.x)	xx (xx.x)
Male	xx (xx.x)	xx (xx.x)	xx (xx.x)
Race [n (%)]			
White	xx (xx.x)	xx (xx.x)	xx (xx.x)
Black	xx (xx.x)	xx (xx.x)	xx (xx.x)
Asian	xx (xx.x)	xx (xx.x)	xx (xx.x)
Native Hawaiian or other Pacific Islander	xx (xx.x)	xx (xx.x)	xx (xx.x)
American Indian or Alaska Native	xx (xx.x)	xx (xx.x)	xx (xx.x)
Other	xx (xx.x)	xx (xx.x)	xx (xx.x)
Missing	xx (xx.x)	xx (xx.x)	xx (xx.x)
Ethnicity [n (%)]			
Hispanic or Latino	xx (xx.x)	xx (xx.x)	xx (xx.x)
Not Hispanic or Latino	xx (xx.x)	xx (xx.x)	xx (xx.x)

[a]NART error score is the number of words pronounced incorrectly out of 50 total words.
Source: Listing xx.x.xx, Dataset: [NAME], Program: xxxxxx.sas, Output: T_16_1_1_1_XXXXX.rtf, Generated on: DDMONYYYY HH:MM
Page 1 of 2

Table 16.1.3.1 Demographic Characteristics - ITT Primary Population (cont.)

Variable	GDNF (N = XXX)	Placebo (N = XXX)	Total (N = XXX)
Weight at baseline (kg)			
N	xx	xx	xx
Mean (SD)	xx.xx (xx.xxx)	xx.xx (xx.xxx)	xx.xx (xx.xxx)
Median	xx.x	xx.x	xx.x
Min, Max	xx.x, xx.x	xx.x, xx.x	xx.x, xx.x
Height at baseline (m)			
N	xx	xx	xx
Mean (SD)	xx.x (xx.xx)	xx.x (xx.xx)	xx.x (xx.xx)
Median	xx	xx	xx
Min, Max	xx, xx	xx, xx	xx, xx
BMI at baseline (kg/m^2)			
N	xx	xx	xx
Mean (SD)	xx.x (xx.xx)	xx.x (xx.xx)	xx.x (xx.xx)
Median	xx	xx	xx
Min, Max	xx, xx	xx, xx	xx, xx
NART error score (points)[a]			
n	xx	xx	xx
Mean (SD)	xx.x (xx.xx)	xx.x (xx.xx)	xx.x (xx.xx)
Median	xx	xx	xx
Min, Max	xx, xx	xx, xx	xx, xx

[a] NART error score is the number of words pronounced incorrectly out of 50 total words.
Source: Listing xx.x.xx, Dataset: [NAME], Program: xxxxxx.sas, Output: T_16_1_1_1_XXXXX.rtf, Generated on: DDMONYYYY HH:MM
Page 2 of 2

Programming Note: Add "Missing" if necessary to categorical variables.

Table 16.1.3.2 Demographic Characteristics - ITT Overall Population

Programming Note: Repeat table for different population.

Table 16.1.4.1 Parkinson's Disease History at Screening - ITT Primary Population

Variable	GDNF (N = XXX)	Placebo (N = XXX)	Total (N = XXX)
Duration since first PD symptom (years)			
N	xx	xx	xx
Mean (SD)	xx.xx (xx.xxx)	xx.xx (xx.xxx)	xx.xx (xx.xxx)
Median	xx.x	xx.x	xx.x
Min, Max	xx.x, xx.x	xx.x, xx.x	xx.x, xx.x
Duration since PD diagnosis (years)			
N	xx	xx	xx
Mean (SD)	xx.xx (xx.xxx)	xx.xx (xx.xxx)	xx.xx (xx.xxx)
Median	xx.x	xx.x	xx.x
Min, Max	xx.x, xx.x	xx.x, xx.x	xx.x, xx.x
Hoehn and Yahr stage in OFF state [n (%)]			
Stage 0: No signs of disease	xx (xx.x)	xx (xx.x)	xx (xx.x)
Stage 1: Unilateral symptoms only	xx (xx.x)	xx (xx.x)	xx (xx.x)
Stage 1.5: Unilateral and axial involvement	xx (xx.x)	xx (xx.x)	xx (xx.x)
Stage 2: Bilateral symptoms; no impairment of balance	xx (xx.x)	xx (xx.x)	xx (xx.x)
Stage 2.5: Mild bilateral disease with recovery on pull test	xx (xx.x)	xx (xx.x)	xx (xx.x)
Stage 3: Balance impairment; mild to moderate disease; physically independent	xx (xx.x)	xx (xx.x)	xx (xx.x)
Missing	xx (xx.x)	xx (xx.x)	xx (xx.x)
OFF state UPDRS motor score (part III)			
N	xx	xx	xx
Mean (SD)	xx.xx (xx.xxx)	xx.xx (xx.xxx)	xx.xx (xx.xxx)
Median	xx.x	xx.x	xx.x
Min, Max	xx.x, xx.x	xx.x, xx.x	xx.x, xx.x
ON state UPDRS motor score (part III)			
N	xx	xx	xx
Mean (SD)	xx.xx (xx.xxx)	xx.xx (xx.xxx)	xx.xx (xx.xxx)
Median	xx.x	xx.x	xx.x
Min, Max	xx.x, xx.x	xx.x, xx.x	xx.x, xx.x

[a] Percentage change in UPDRS motor score (part III) following a levodopa challenge.
Source: Listing xx.x.xx, Dataset: [NAME], Program: xxxxxx.sas, Output: T_16_1_1_1_XXXXX.rtf, Generated on: DDMONYYYY HH:MM
Page 1 of 2

Table 16.1.4.1 Parkinson's Disease History at Screening - ITT Primary Population (cont.)

Variable	GDNF (N = XXX)	Placebo (N = XXX)	Total (N = XXX)
Total daily levodopa dose (mg)			
N	xx	xx	xx
Mean (SD)	xx.xx (xx.xxx)	xx.xx (xx.xxx)	xx.xx (xx.xxx)
Median	xx.x	xx.x	xx.x
Min, Max	xx.x, xx.x	xx.x, xx.x	xx.x, xx.x
Total daily levodopa equivalent dose (mg)			
N	xx	xx	xx
Mean (SD)	xx.xx (xx.xxx)	xx.xx (xx.xxx)	xx.xx (xx.xxx)
…	…	…	…
PD medications [n (%)]			
Levodopa preparations	xx (xx.x)	xx (xx.x)	xx (xx.x)
Dopamine agonists	xx (xx.x)	xx (xx.x)	xx (xx.x)
COMT inhibitors	xx (xx.x)	xx (xx.x)	xx (xx.x)
MAO-B inhibitors	xx (xx.x)	xx (xx.x)	xx (xx.x)
Other	xx (xx.x)	xx (xx.x)	xx (xx.x)
Responsiveness to levodopa[a] (%)			
N	xx	xx	xx
Mean (SD)	xx.xx (xx.xxx)	xx.xx (xx.xxx)	xx.xx (xx.xxx)
…	…	…	…
OFF time per day (hours)			
N	xx	xx	xx
Mean (SD)	xx.xx (xx.xxx)	xx.xx (xx.xxx)	xx.xx (xx.xxx)
…	…	…	…

[a] Percentage change in UPDRS motor score (part III) following a levodopa challenge.
Source: Listing xx.x.xx, Dataset: [NAME], Program: xxxxxx.sas, Output: T_16_1_1_1_XXXXX.rtf, Generated on: DDMONYYYY HH:MM
Page 2 of 2

Programming Note: Add "Missing" if necessary to categorical variables. See Section 5.0 definition of screening levodopa and levodopa equivalent dose.

Table 16.1.4.2 Parkinson's Disease History at Screening - ITT Overall Population

Programming Note: Repeat table for different population.

Table 16.1.4.3 Impulsiveness Behaviour Scale (UPPS-P) at Screening - ITT Primary Population

Variable<br>Statistic	GDNF<br>(N = XXX)	Placebo<br>(N = XXX)	Total<br>(N = XXX)
(Negative) Urgency subscale			
N	xx	xx	xx
Mean (SD)	xx.x (xx.xx)	xx.x (xx.xx)	xx.x (xx.xx)
Median	xx.x	xx.x	xx.x
Min, Max	xx, xx	xx, xx	xx, xx
(Lack of) Premeditation subscale			
N	xx	xx	xx
Mean (SD)	xx.x (xx.xx)	xx.x (xx.xx)	xx.x (xx.xx)
Median	xx.x	xx.x	xx.x
Min, Max	xx, xx	xx, xx	xx, xx
…			

Note: The UPPS-P is a 59-item scale that measures impulsiveness in 5 subscales. Each question is scored 1 to 4. A lower score indicates less impulsiveness. Individual missing items are imputed using the average of non-missing scores in each subscale.
Source: Listing xx.x.xx, Dataset: [NAME], Program: xxxxxx.sas, Output: T_16_1_1_1_XXXXX.rtf, Generated on: DDMONYYYY HH:MM
Page x of y

Programming Note: Repeat summary for each of the other 3 subscales (in addition to (Negative) Urgency and (Lack of) Premeditation subscale): (Lack of) Perseverance subscale, Sensation Seeking subscale, Positive Urgency subscale. Calculate the score for each subscale from the responses to the individual items in the subscale.

Table 16.1.5.1 General Medical History Resolved at Screening by System Organ Class and Preferred Term - ITT Primary Population

System Organ Class<br>Preferred Term	GDNF<br>(N = XXX)<br>n (%)	Placebo<br>(N = XXX)<br>n (%)	Total<br>(N = XXX)<br>n (%)
Subjects with at least one resolved medical history item at screening	xx (xx.x)	xx (xx.x)	xx (xx.x)
[System Organ Class 1]	xx (xx.x)	xx (xx.x)	xx (xx.x)
[Preferred Term 1]	xx (xx.x)	xx (xx.x)	xx (xx.x)
[Preferred Term 2]	xx (xx.x)	xx (xx.x)	xx (xx.x)
[System Organ Class 2]	xx (xx.x)	xx (xx.x)	xx (xx.x)
[Preferred Term 1]	xx (xx.x)	xx (xx.x)	xx (xx.x)
[Preferred Term 2]	xx (xx.x)	xx (xx.x)	xx (xx.x)
…			

Note: Medical history is coded using MedDRA version 19.0. For each system organ class and preferred term, subjects are included only once.
Source: Listing xx.x.xx, Dataset: [NAME], Program: xxxxxx.sas, Output: T_16_1_1_1_XXXXX.rtf, Generated on: DDMONYYYY HH:MM
Page 1 of 1

Programming Note: Sort alphabetically by SOC and then alphabetically for PT.

Table 16.1.5.2 General Medical History Current/Active at Screening by System Organ Class and Preferred Term - ITT Primary Population

Programming Note: Repeat table with only "current/active" items. Replace first row header with "Subjects with at least one current/active medical history item at screening."

Table 16.1.6.1 Prior Medications by WHO Drug Dictionary ATC Class and Preferred Name - ITT Primary Population

ATC Class<br>Preferred Name	GDNF<br>(N = XXX)<br>n (%)	Placebo<br>(N = XXX)<br>n (%)	Total<br>(N = XXX)<br>n (%)
Subjects with at least one prior medication	xx (xx.x)	xx (xx.x)	xx (xx.x)
[ATC Class 1]	xx (xx.x)	xx (xx.x)	xx (xx.x)
[Preferred Name 1]	xx (xx.x)	xx (xx.x)	xx (xx.x)
[Preferred Name 2]	xx (xx.x)	xx (xx.x)	xx (xx.x)
[ATC Class 2]	xx (xx.x)	xx (xx.x)	xx (xx.x)
[Preferred Name 1]	xx (xx.x)	xx (xx.x)	xx (xx.x)
[Preferred Name 2]	xx (xx.x)	xx (xx.x)	xx (xx.x)
…			

Note: Prior medications are any medications with start and stop dates prior to the first randomized study medication dose date. Medications are coded using WHO Drug Dictionary Enhanced version 2016Mar01. For each ATC class and preferred name, subjects are included only once.
Source: Listing xx.x.xx, Dataset: [NAME], Program: xxxxxx.sas, Output: T_16_1_1_1_XXXXX.rtf, Generated on: DDMONYYYY HH:MM
Page x of y

Programming Note: Sort alphabetically for both ATC class and preferred name.

Table 16.1.6.2.1 Concomitant Parkinson's Disease Medications by WHO Drug Dictionary ATC Class and Preferred Name - ITT Primary Population

Programming Note: Repeat prior medications table with concomitant PD medications. First row should be "Subjects with at least one concomitant PD medication."

Table 16.1.6.2.2 Other Concomitant Medications by WHO Drug Dictionary ATC Class and Preferred Name - ITT Primary Population

Programming Note: Repeat prior medications table with other concomitant medications. First row should be "Subjects with at least one other concomitant medication."

Table 16.1.7 Catheter Placement Surgery Trajectories - ITT Primary Population

Surgery<br>Category	GDNF<br>(N = XXX)<br>n (%)	Placebo<br>(N = XXX)<br>n (%)	Total<br>(N = XXX)<br>n (%)
Initial Surgery	xx (xx.x)	xx (xx.x)	xx (xx.x)
Vertical	xx (xx.x)	xx (xx.x)	xx (xx.x)
Horizontal: Anterior-Posterior	xx (xx.x)	xx (xx.x)	xx (xx.x)
Horizontal: Posterior-Anterior	xx (xx.x)	xx (xx.x)	xx (xx.x)
Repositioning Surgery 1	xx (xx.x)	xx (xx.x)	xx (xx.x)
Vertical	xx (xx.x)	xx (xx.x)	xx (xx.x)
Horizontal: Anterior-Posterior	xx (xx.x)	xx (xx.x)	xx (xx.x)
Horizontal: Posterior-Anterior	xx (xx.x)	xx (xx.x)	xx (xx.x)
Repositioning Surgery 2	xx (xx.x)	xx (xx.x)	xx (xx.x)
Vertical	xx (xx.x)	xx (xx.x)	xx (xx.x)
Horizontal: Anterior-Posterior	xx (xx.x)	xx (xx.x)	xx (xx.x)
Horizontal: Posterior-Anterior	xx (xx.x)	xx (xx.x)	xx (xx.x)

Source: Listing xx.x.xx, Dataset: [NAME], Program: xxxxxx.sas, Output: T_16_1_1_1_XXXXX.rtf, Generated on: DDMONYYYY HH:MM
Page 1 of 1

Table 16.1.8 Catheter Positioning Accuracy as Determined by Post-Surgery CT - ITT Primary Population

Surgery<br>Parameter (unit)<br>Statistic	GDNF<br>(N = XXX)	Placebo<br>(N = XXX)	Total<br>(N = XXX)
Initial surgery			
Difference between planned target and actual target (mm)			
n	xx	xx	xx
Mean (SD)	xx.xxx (xx.xxxx)	xx.xxx (xx.xxxx)	xx.xxx (xx.xxxx)
Median	xx.xx	xx.xx	xx.xx
Min, Max	xx.xx, xx.xx	xx.xx, xx.xx	xx.xx, xx.xx
Repositioning surgery #1			
Difference between planned target and actual target (mm)			
n	xx	xx	xx
Mean (SD)	xx.xxx (xx.xxxx)	xx.xxx (xx.xxxx)	xx.xxx (xx.xxxx)
Median	xx.xx	xx.xx	xx.xx
Min, Max	xx.xx, xx.xx	xx.xx, xx.xx	xx.xx, xx.xx
…			

Note: N is the number of subjects in each treatment group and n is the total number of catheters for each treatment group.
Source: Listing xx.x.xx, Dataset: [NAME], Program: xxxxxx.sas, Output: xxxx.rtf, Generated on: DDMONYYYY HH:MM
Page x of y

Programming Note: Repeat as necessary for subsequent repositioning surgeries.

Table 16.1.9 Contrast-Enhanced Test Infusions With T1-weighted MRI Prior to Start of Study Medication - ITT Primary Population

**Category**	**GDNF (N = XXX) n (%)**	**Placebo (N = XXX) n (%)**	**Total (N = XXX) n (%)**
Number (%) of subjects with			
1 test infusion	xx (xx.x)	xx (xx.x)	xx (xx.x)
2 test infusions	xx (xx.x)	xx (xx.x)	xx (xx.x)
≥ 3 test infusions	xx (xx.x)	xx (xx.x)	xx (xx.x)

Note: The table excludes <*XX*> subjects <*No. XX, treatment*> who only received a plain aCSF test infusion followed by T2-weighted MRI.
Source: Listing xx.x.xx, Dataset: [NAME], Program: xxxxxx.sas, Output: T_16_1_1_1_XXXXX.rtf, Generated on: DDMONYYYY HH:MM
Page 1 of 1

Programming Note: Per footnote, include only T1-weighted MRIs. There are only a couple of subjects that had only T2-weighted MRI without contrast who require footnoting.

16.2 EFFICACY DATA TABLES

16.2.1 UPDRS PRIMARY EFFICACY TABLES

Table 16.2.1.1.1 Primary Efficacy Endpoint - OFF State UPDRS Motor Score (Part III): Change from Baseline to Week 40 – MMRM, ITT Primary Population

	GDNF (N = XXX)			Placebo (N = XXX)		
Visit<br>Statistic	Value	Change From Baseline	Percentage Change From Baseline	Value	Change From Baseline	Percentage Change From Baseline
Week 0 (Baseline)						
N	xx			xx		
Mean (SD)	xx.xx (xx.xxx)			xx.xx (xx.xxx)		
Median	xx.x			xx.x		
Min, Max	xx.x, xx.x			xx.x, xx.x		
Week 40						
N	xx	xx	xx	xx	xx	xx
Mean (SD)	xx.xx (xx.xxx)	xx.xx (xx.xxx)	xx.xx (xx.xxx)	xx.xx (xx.xxx)	xx.xx (xx.xxx)	xx.xx (xx.xxx)
Median	xx.x	xx.x	xx.x	xx.x	xx.x	xx.x
Min, Max	xx.x, xx.x	xx.x, xx.x	xx.x, xx.x	xx.x, xx.x	xx.x, xx.x	xx.x, xx.x
Least squares mean[a] (95% CI)			xx.x (xx.x, xx.x)			xx.x (xx.x, xx.x)
Least squares mean difference versus placebo[a] (95% CI)			xx.x (xx.x, xx.x)			
p-value[a]			0.xxxx			

[a] Estimates are from a mixed-effect model with repeated measures (MMRM) with baseline UPDRS score as a covariate, treatment group and visit and treatment group*visit as fixed effects, and subject within treatment group as a random effect.
Note: Lower scores represent better functioning. End of study visit data are included in the appropriate visit week. For subject 45, items 22, 27, 28, 29 and 30 are excluded from the score.
Source: Listing xx.x.xx, Dataset: [NAME], Program: xxxxxx.sas, Output: xxxx.rtf, Generated on: DDMONYYYY HH:MM
Page x of y

Table 16.2.1.1.2 Sensitivity Analysis - OFF State UPDRS Motor Score (Part III): Change from Baseline to Week 40 – MMRM, ITT Overall Population

Programming Note: Repeat Table 16.2.1.1.1 for different population.

Table 16.2.1.1.3 Sensitivity Analysis - OFF State UPDRS Motor Score (Part III): Change from Baseline to Week 40 – MMRM, Per-Protocol Population

Programming Note: Repeat Table 16.2.1.1.1 for different population.Delete footnote regarding subject 45.

Table 16.2.1.2 Sensitivity Analysis - OFF State UPDRS Motor Score (Part III): Change from Baseline to Week 40 – LOCF ANCOVA, ITT Primary Population

	GDNF (N = XXX)			Placebo (N = XXX)		
**Visit Statistic**	**Value**	**Change From Baseline**	**Percentage Change From Baseline**	**Value**	**Change From Baseline**	**Percentage Change From Baseline**
Week 0 (Baseline)						
N	xx			xx		
Mean (SD)	xx.xx (xx.xxx)			xx.xx (xx.xxx)		
Median	xx.x			xx.x		
Min, Max	xx.x, xx.x			xx.x, xx.x		
Week 40						
N	xx	xx	xx	xx	xx	xx
Mean (SD)	xx.xx (xx.xxx)	xx.xx (xx.xxx)	xx.xx (xx.xxx)	xx.xx (xx.xxx)	xx.xx (xx.xxx)	xx.xx (xx.xxx)
Median	xx.x	xx.x	xx.x	xx.x	xx.x	xx.x
Min, Max	xx.x, xx.x	xx.x, xx.x	xx.x, xx.x	xx.x, xx.x	xx.x, xx.x	xx.x, xx.x
Mean difference versus placebo[a] (95% CI)			xx.x (xx.x, xx.x)			
p-value[a]			0.xxxx			

[a] Estimates are from an analysis of covariance (ANCOVA) model with baseline UPDRS score as a covariate and treatment group as a factor.
Note: Lower scores represent better functioning. The last UPDRS value prior to Week 40 is carried forward for subjects that have no Week 40 value. For subject 45, items 22, 27, 28, 29 and 30 are excluded from the score.
Source: Listing xx.x.xx, Dataset: [NAME], Program: xxxxxx.sas, Output: xxxx.rtf, Generated on: DDMONYYYY HH:MM
Page x of y

Table 16.2.1.3 Sensitivity Analysis - OFF State UPDRS Motor Score (Part III): Change from Baseline to Week 40 – Observed Data ANCOVA, ITT Primary Population

Programming Note: Repeat Table 16.2.1.2, but using observed data only (no imputation). Please delete the sentence in the footnote: "The last UPDRS value prior to Week 40 is carried forward for subjects that are missing Week 40 value." and replace with: "Only subjects with a Week 40 UPDRS value are included in the observed data analysis."

Table 16.2.1.4 Sensitivity Analysis - OFF State UPDRS Motor Score (Part III) by Visit: Change from Baseline – Observed Data ANCOVA, ITT Primary Population

	GDNF (N = XXX)			Placebo (N = XXX)		
Visit<br>Statistic	Value	Change From Baseline	Percentage Change From Baseline	Value	Change From Baseline	Percentage Change From Baseline
Screening						
N	xx			xx		
Mean (SD)	xx.xx (xx.xxx)			xx.xx (xx.xxx)		
Median	xx.x			xx.x		
Min, Max	xx.x, xx.x			xx.x, xx.x		
Week 0 (Baseline)						
N	xx			xx		
Mean (SD)	xx.xx (xx.xxx)			xx.xx (xx.xxx)		
Median	xx.x			xx.x		
Min, Max	xx.x, xx.x			xx.x, xx.x		
Week 8						
N	xx	xx	xx	xx	xx	xx
Mean (SD)	xx.xx (xx.xxx)	xx.xx (xx.xxx)	xx.xx (xx.xxx)	xx.xx (xx.xxx)	xx.xx (xx.xxx)	xx.xx (xx.xxx)
Median	xx.x	xx.x	xx.x	xx.x	xx.x	xx.x
Min, Max	xx.x, xx.x	xx.x, xx.x	xx.x, xx.x	xx.x, xx.x	xx.x, xx.x	xx.x, xx.x
Mean difference versus placebo[a] (95% CI)			xx.x (xx.x, xx.x)			
p-value[a]			0.xxxx			
Week 16						
…						

[a] Estimates are from an analysis of covariance (ANCOVA) model with baseline UPDRS score as a covariate and treatment group as a factor.
Note: Lower scores represent better functioning. End of study visit data are included in the appropriate visit week. Only subjects with a UPDRS value at the respective visit are included in the observed data analysis. For subject 45, items 22, 27, 28, 29 and 30 are excluded from the score.
Source: Listing xx.x.xx, Dataset: [NAME], Program: xxxxxx.sas, Output: xxxx.rtf, Generated on: DDMONYYYY HH:MM
Page x of y

Programming Note: Visits are screening, baseline, Weeks 8, 16, 24, 32, and 40. Statistics are for all visits only for this by-visit table.

Table 16.2.1.5 Sensitivity Analysis - OFF State UPDRS Motor Score (Part III): Change from Screening to Week 40 – MMRM, ITT Primary Population

Programming Note: Repeat Table 16.2.1.1.1 using screening instead of Week 0 values as the baseline values. In the table itself, please write screening instead of baseline wherever this occurs.

Table 16.2.1.6 Sensitivity Analysis - OFF State UPDRS Motor Score (Part III): Change from Baseline to Week 40 - MMRM – Excluding Subjects with Vertical Catheter Trajectory, ITT Primary Population

Programming Note: Repeat of Table 16.2.1.1.1. Please add "Note: Those subjects whose catheters are positioned using a vertical trajectory are excluded from this analysis."

16.2.2 UPDRS Secondary Efficacy Tables

Table 16.2.2.1.1 ON State UPDRS Motor Score (Part III): Change from Baseline to Week 40 – MMRM, ITT Primary Population

Programming Note: Repeat of Table 16.2.1.1.1, OFF state UPDRS scores (Part III) at Week 40 MMRM for ITT, but for ON state parameters. Please delete "Primary Efficacy Endpoint" from the title.

Table 16.2.2.1.2 ON State UPDRS Motor Score (Part III): Change from Baseline to Week 40 – MMRM, ITT Overall Population

Table 16.2.2.1.3 ON State UPDRS Motor Score (Part III): Change from Baseline to Week 40 – MMRM, Per-Protocol Population

Programming Note: Delete footnote regarding subject 45.

Table 16.2.2.1.4 ON State UPDRS Motor Score (Part III) by Visit: Change from Baseline – Observed Data ANCOVA, ITT Primary Population

Programming Note: Repeat of Table 16.2.1.4, but for ON state motor score (part III). Visits are baseline, Weeks 8, 16, 24, 32, and 40. Inferential statistics are for Week 40 only. Please delete "Sensitivity Analysis" from the title.

Table 16.2.2.2.1 OFF State UPDRS ADL Score (Part II): Change from Baseline to Week 40 – MMRM, ITT Primary Population

Programming Note: Repeat of Table 16.2.1.1.1, but for OFF state ADL (Part II) score. Please delete "Primary Efficacy Endpoint" from the title. Change footnote regarding subject 45 to: "Data for subject 45 are excluded from analysis."

Table 16.2.2.2.2 OFF State UPDRS ADL Score (Part II): Change from Baseline to Week 40 – MMRM, ITT Overall Population

Programming Note: Change footnote regarding subject 45 to: "Data for subject 45 are excluded from analysis."

Table 16.2.2.2.3 OFF State UPDRS ADL Score (Part II): Change from Baseline to Week 40 – MMRM, Per-Protocol Population

Programming Note: Delete footnote regarding subject 45.

Table 16.2.2.2.4 OFF State UPDRS ADL Score (Part II) by Visit: Change from Baseline – Observed Data ANCOVA, ITT Primary Population

Programming Note: Repeat of Table 16.2.1.4, but for OFF state ADL (Part II). Visits are baseline, Weeks 8, 16, 24, 32, and 40. Inferential statistics are for Week 40 only. Please delete "Sensitivity Analysis" from the title. Change footnote regarding subject 45 to: "Data for subject 45 are excluded from analysis."

Table 16.2.2.3.1 ON State UPDRS ADL Score (Part II): Change from Baseline to Week 40 – MMRM, ITT Primary Population

Programming Note: Repeat of Table 16.2.1.1.1, but for ON state ADL (Part II) score. Please delete "Primary Efficacy Endpoint" from the title. Change footnote regarding subject 45 to: "Data for subject 45 are excluded from analysis."

Table 16.2.2.3.2 ON State UPDRS ADL Score (Part II): Change from Baseline to Week 40 – MMRM, ITT Overall Population

Programming Note: Change footnote regarding subject 45 to: "Data for subject 45 are excluded from analysis."

Table 16.2.2.3.3 ON State UPDRS ADL Score (Part II): Change from Baseline to Week 40 – MMRM, Per-Protocol Population

Programming Note: Delete footnote regarding subject 45.

Table 16.2.2.3.4 ON State UPDRS ADL Score (Part II) by Visit: Change from Baseline – Observed Data ANCOVA, ITT Primary Population

Programming Note: Repeat of Table 16.2.1.4, but for ON state ADL (Part II). Visits are baseline, Weeks 8, 16, 24, 32, and 40. Inferential statistics are for Week 40 only. Please delete "Sensitivity Analysis" from the title. Change footnote regarding subject 45 to: "Data for subject 45 are excluded from analysis."

Table 16.2.2.4.1 OFF State UPDRS Total Score: Change from Baseline to Week 40 – MMRM, ITT Primary Population
Programming Note: Repeat of Table 16.2.1.1.1, but for OFF state total score. Please delete "Primary Efficacy Endpoint" from the title. Change footnote regarding subject 45 to: "Data for subject 45 are excluded from analysis."

Table 16.2.2.4.2 OFF State UPDRS Total Score: Change from Baseline to Week 40 – MMRM, ITT Overall Population
Programming Note: Change footnote regarding subject 45 to: "Data for subject 45 are excluded from analysis."

Table 16.2.2.4.3 OFF State UPDRS Total Score: Change from Baseline to Week 40 – MMRM, Per-Protocol Population
Programming Note: Delete footnote regarding subject 45.

Table 16.2.2.4.4 ON State UPDRS Total Score: Change from Baseline to Week 40 – MMRM, ITT Primary Population
Programming Note: Repeat of Table 16.2.1.1.1, but for ON state total score. Please delete "Primary Efficacy Endpoint" from the title. Change footnote regarding subject 45 to: "Data for subject 45 are excluded from analysis."

Table 16.2.2.4.5 ON State UPDRS Total Score: Change from Baseline to Week 40 – MMRM, ITT Overall Population
Programming Note: Change footnote regarding subject 45 to: "Data for subject 45 are excluded from analysis."

Table 16.2.2.4.6 ON State UPDRS Total Score: Change from Baseline to Week 40 – MMRM, Per-Protocol Population
Programming Note: Delete footnote regarding subject 45.

Table 16.2.2.5 UPDRS Mentation, Behavior, and Mood Score (Part I): Change from Baseline to Week 40 – MMRM, ITT Primary Population
Programming Note: Repeat of Table 16.2.1.1.1, but for part I score. Please delete "Primary Efficacy Endpoint" from the title. Change footnote regarding subject 45 to: "Data for subject 45 are excluded from analysis."

Table 16.2.2.6 UPDRS Complications of Therapy Score (Part IV): Change from Baseline to Week 40 – MMRM, ITT Primary Population
Programming Note: Repeat of Table 16.2.1.1.1, but for part IV score. Please delete "Primary Efficacy Endpoint" from the title. Change footnote regarding subject 45 to: "Data for subject 45 are excluded from analysis."

16.2.3 PD Diary Secondary efficacy Tables

Table 16.2.3.1 Motor Fluctuation Diary Ratings by Visit: Change from Baseline – MMRM, ITT Primary Population

Parameter	GDNF (N = XXX)		Placebo (N = XXX)	
Visit<br>Statistic	Value	Change From Baseline	Value	Change From Baseline
OFF time per day (hours)				
Screening				
n	xx		xx	
Mean (SD)	xx.xx (xx.xxx)		xx.xx (xx.xxx)	
Median	xx.x		xx.x	
Min, Max	xx.x, xx.x		xx.x, xx.x	
Week 0 (Baseline)				
…				
Week 40				
n	xx	xx	xx	xx
Mean (SD)	xx.xx (xx.xxx)	xx.xx (xx.xxx)	xx.xx (xx.xxx)	xx.xx (xx.xxx)
Median	xx.x	xx.x	xx.x	xx.x
Min, Max	xx.x, xx.x	xx.x, xx.x	xx.x, xx.x	xx.x, xx.x
Least squares mean[a] (95% CI)		xx.x (xx.x, xx.x)		xx.x (xx.x, xx.x)
Least squares mean difference versus placebo[a] (95% CI)		xx.x (xx.x, xx.x)		
p-value[a]		0.xxxx		

[a] Estimates are from a mixed-effect model with repeated measures (MMRM) with baseline variable as a covariate, treatment group and visit and treatment group*visit as fixed effects, and subject within treatment group as a random effect.
Note: End of study visit data are included in the appropriate visit week.
Note: Total good-quality ON time per day is defined as the sum of ON time per day without dyskinesias + ON time per day with non-troublesome dyskinesias.

Source: Listing xx.x.xx, Dataset: [NAME], Program: xxxxxx.sas, Output: xxxx.rtf, Generated on: DDMONYYYY HH:MM
Page x of y

Programming Note: PD diary parameters are OFF time per day, total good-quality ON time per day (sum of ON time per day without dyskinesias + ON time per day with non-troublesome dyskinesias), ON time per day without dyskinesias, ON time per day with non-troublesome dyskinesias, and ON time per day with troublesome dyskinesias. Visits are Screening, baseline, Weeks 8, 16, 24, 32, and 40. Statistics are for Week 40 only.

Table 16.2.3.2 Motor Fluctuation Diary Ratings by Visit: Change from Baseline – MMRM, ITT Overall Population

Programming Note: Repeat above table for different population. Statistics are for Week 40 only.

Table 16.2.3.3 Motor Fluctuation Diary Ratings by Visit: Change from Baseline – MMRM, Per-Protocol Population

Programming Note: Repeat above table for different population. Statistics are for Week 40 only.

16.2.4 Supplementary Efficacy Tables

Table 16.2.4.1 OFF and ON State Timed Walking Test by Visit: Change from Baseline – MMRM, ITT Primary Population

Parameter<br>Visit<br>Statistic	GDNF (N = XXX)<br>Value	 Change From Baseline	Placebo (N = XXX)<br>Value	 Change From Baseline
OFF state timed walking test (seconds)				
Week 0 (Baseline)				
n	xx		xx	
Mean (SD)	xx.xx (xx.xxx)		xx.xx (xx.xxx)	
Median	xx.x		xx.x	
Min, Max	xx.x, xx.x		xx.x, xx.x	
Week 8				
…				
Week 40				
n	xx	xx	xx	xx
Mean (SD)	xx.xx (xx.xxx)	xx.xx (xx.xxx)	xx.xx (xx.xxx)	xx.xx (xx.xxx)
Median	xx.x	xx.x	xx.x	xx.x
Min, Max	xx.x, xx.x	xx.x, xx.x	xx.x, xx.x	xx.x, xx.x
Least squares mean[a] (95% CI)		xx.x (xx.x, xx.x)		xx.x (xx.x, xx.x)
Least squares mean difference versus placebo[a] (95% CI)		xx.x (xx.x, xx.x)		
p-value[a]		0.xxxx		

[a] Estimates are from a mixed-effect model with repeated measures (MMRM) with baseline time as a covariate, treatment group and visit and treatment group*visit as fixed effects, and subject within treatment group as a random effect.
Note: Shorter times represent better function. Two trials per state per visit are averaged for analysis. If only the first trial is completed for a particular state, then the second trial result will be imputed with the worst non-missing second trial result from all subjects for that particular state during the study. If both trials are missing, then the endpoint is not reported for that visit. End of study visit data are included in the appropriate visit week. Data for subject 45 are excluded from analysis.
Source: Listing xx.x.xx, Dataset: [NAME], Program: xxxxxx.sas, Output: xxxx.rtf, Generated on: DDMONYYYY HH:MM
Page x of y

Programming Note: Timed walking test parameters are OFF state timed walking test and ON state timed walking test (seconds). Visits are baseline, Weeks 8, 16, 24, 32, and 40. Statistics are for Week 40 only.

Table 16.2.4.2 OFF and ON State Timed Tapping Test by Visit: Change from Baseline – MMRM, ITT Primary Population

Programming Note: Repeat Table 16.2.4.1, but for time tapping test by visit. Timed tapping test parameters are OFF state timed tapping test (left hand), OFF state timed tapping test (right hand), ON state timed tapping test (left hand) and ON state timed tapping test (right hand; seconds). Visits are baseline, Weeks 8, 16, 24, 32, and 40. Statistics are for Week 40 only. Replace footnote a text with "…(MMRM) with baseline number of taps as a covariate…". Replace note with: "More taps represent better function. Two trials per state per hand for each visit are averaged for analysis. If only the first trial is completed for a particular state, then the second trial result will be imputed with the worst non-missing second trial result from all subjects for that particular state during the study. If both trials are missing, then the endpoint is not reported for that visit. End of study visit data are included in the appropriate visit week."

Table 16.2.4.3 NMSS Score by Visit: Change from Baseline – MMRM, ITT Primary Population

Parameter<br>Visit<br>Statistic	GDNF (N = XXX)<br>Value	GDNF (N = XXX)<br>Change From Baseline	Placebo (N = XXX)<br>Value	Placebo (N = XXX)<br>Change From Baseline
NMSS Total Score				
Screening (Baseline)				
n	xx		xx	
Mean (SD)	xx.xx (xx.xxx)		xx.xx (xx.xxx)	
Median	xx.x		xx.x	
Min, Max	xx.x, xx.x		xx.x, xx.x	
Week 12				
…				
Week 40				
n	xx	xx	xx	xx
Mean (SD)	xx.xx (xx.xxx)	xx.xx (xx.xxx)	xx.xx (xx.xxx)	xx.xx (xx.xxx)
Median	xx.x	xx.x	xx.x	xx.x
Min, Max	xx.x, xx.x	xx.x, xx.x	xx.x, xx.x	xx.x, xx.x
Least squares mean[a] (95% CI)		xx.x (xx.x, xx.x)		xx.x (xx.x, xx.x)
Least squares mean difference versus placebo[a] (95% CI)		xx.x (xx.x, xx.x)		
p-value[a]		0.xxxx		

[a] Estimates are from a mixed-effect model with repeated measures (MMRM) with baseline score as a covariate, treatment group and visit and treatment group*visit as fixed effects, and subject within treatment group as a random effect.
Note: The NMSS is a 30-item interview-based scale that rates non-motor symptoms that occurred in the preceding month in 9 domains. Each item is rated from 0 (none) to 3 (severe) for severity and from 1 (rarely) to 4 (very frequent) for frequency. The maximum score for an individual item is 12. The higher the score, the worse the subject's condition. The maximum NMSS total score is 360. Missing individual item scores are imputed using LOCF. End of study visit data are included in the appropriate visit week. Data for subject 45 are excluded from analysis.
Source: Listing xx.x.xx, Dataset: [NAME], Program: xxxxxx.sas, Output: xxxx.rtf, Generated on: DDMONYYYY HH:MM
Page x of y

Programming Note: NMSS parameters are Cardiovascular including falls domain, Sleep/fatigue domain, Mood/cognition domain, Perceptual problems/hallucinations domain; Attention/memory domain, Gastrointestinal tract domain, Urinary domain, Sexual function domain, Miscellaneous domain, and NMSS total score. Visits are baseline, Weeks 12, 24, and 40. Statistics are for Week 40 only.

Table 16.2.4.4 PDQ-39 Score: Change from Baseline to Week 40 – Observed Data ANCOVA, ITT Primary Population

Parameter<br>Visit<br>Statistic	GDNF (N = XXX)		Placebo (N = XXX)	
	**Value**	**Change From Baseline**	**Value**	**Change From Baseline**
Single Index (Total) PDQ-39 Score				
Screening (Baseline)				
n	xx		xx	
Mean (SD)	xx.xx (xx.xxx)		xx.xx (xx.xxx)	
Median	xx.x		xx.x	
Min, Max	xx.x, xx.x		xx.x, xx.x	
Week 40				
n	xx	xx	xx	xx
Mean (SD)	xx.xx (xx.xxx)	xx.xx (xx.xxx)	xx.xx (xx.xxx)	xx.xx (xx.xxx)
Median	xx.x	xx.x	xx.x	xx.x
Min, Max	xx.x, xx.x	xx.x, xx.x	xx.x, xx.x	xx.x, xx.x
Mean difference versus placebo[a]				
(95% CI)		xx.x (xx.x, xx.x)		
p-value[a]		0.xxxx		

[a] Estimates are from an analysis of covariance (ANCOVA) model with baseline score as a covariate and treatment group as a factor.
Note: The PDQ-39 is a self-administered 39-item PD-specific scale that rates symptoms that occurred in the preceding month in 8 dimensions. Each item is rated from 0 (never) to 4 (always) for frequency. The score for each dimension is the average of responses in the dimension, weighted by the number of questions and multiplied by 100. PDQ-39 dimension and total scores range from 0 to 100 (0 = no problem, 100 = problem as worse as possible). The higher the score, the worse the subject's condition. Only subjects with a Week 40 value are included in the observed data analysis. If an individual question response is missing, then that dimension score and the single index (total) score are also missing. Data for subject 45 are excluded from analysis.
Source: Listing xx.x.xx, Dataset: [NAME], Program: xxxxxx.sas, Output: xxxx.rtf, Generated on: DDMONYYYY HH:MM
Page x of y

Programming Note: PDQ-39 parameters are Mobility dimension, ADL dimension, Emotional well-being dimension, Stigma dimension, Social support dimension, Cognitions dimension, Communication dimension, Bodily discomfort dimension, and Single index (total) PDQ-39 score. Visits are baseline and Week 40.

Table 16.2.4.5.1 EQ-5D Questionnaire: Baseline and Week 40 – Observed Data, ITT Primary Population

EQ-5D Dimension<br>Visit<br>Response Level	GDNF<br>(N = XXX)<br>n (%)	Placebo<br>(N = XXX)<br>n (%)
Mobility		
Screening (Baseline)		
No problem	xx (xx.x)	xx (xx.x)
Moderate problem	xx (xx.x)	xx (xx.x)
Severe problem	xx (xx.x)	xx (xx.x)
Week 40		
No problem	xx (xx.x)	xx (xx.x)
Moderate problem	xx (xx.x)	xx (xx.x)
Severe problem	xx (xx.x)	xx (xx.x)
Self-care		
Screening (Baseline)		
No problem	xx (xx.x)	xx (xx.x)
Moderate problem	xx (xx.x)	xx (xx.x)
Severe problem	xx (xx.x)	xx (xx.x)
…		

Note: The EQ-5D is a self-administered 5-item scale with three possible answers for each item (1=no problem, 2=moderate problem, 3=severe problem), and a visual analog scale ranging from 0 to 100, where 0 indicates worst health and 100 indicates best health. Only subjects with a Week 40 value are included in the observed data analysis. Data for subject 45 are excluded from analysis.
Source: Listing xx.x.xx, Dataset: [NAME], Program: xxxxxx.sas, Output: T_16_1_1_1_XXXXX.rtf, Generated on: DDMONYYYY HH:MM
Page 1 of 1

Programming Note: Sort in order as on CRF. Continue for Self-care, Usual Activities, Pain / Discomfort, and Anxiety / Depression. Visits are baseline and Week 40. EQ-5D parameters are the frequency counts and percentages of subjects with the different answers to the 5 questions (categorical) and the visual analog scale score (continuous). Visual analog scale data is in the next table.

Table 16.2.4.5.2 EQ-5D Visual Analog Scale: Change from Baseline to Week 40 – Observed Data ANCOVA, ITT Primary Population

Programming Note: Report observed data for baseline, Week 40, and change from baseline summary statistics for visual analog scale (with mean difference, 95% CI, and p-value). Visits are baseline and Week 40. Add note: "The EQ-5D is a self-administered 5-item scale with three possible answers for each item (1=no problem, 2=moderate problem, 3=severe problem), and a visual analog scale ranging from 0 to 100, where 0 indicates worst health and 100 indicates best health. Only subjects with a Week 40 value are included in the observed data analysis. Data for subject 45 are excluded from analysis" EQ-5D parameters are the frequency counts and percentages of subjects with the different answers to the 5 questions (categorical) and the visual analog scale score (continuous).

Table 16.2.4.6 SNAQ Score: Change from Baseline to Week 40 – Observed Data ANCOVA, ITT Primary Population

Programming Note: Repeat observed data ANCOVA table for SNAQ. SNAQ has one total SNAQ score. Visits are baseline and Week 40. Add note: "The SNAQ is a self-administered 4-question instrument with total scores ranging from 4 to 20 (4=poor appetite, 20=good appetite). Only subjects with a Week 40 value are included in the observed data analysis. If an individual question is not answered, then the total score is considered missing."

Table 16.2.4.7 Total Daily Levodopa Dose (mg): Change From Baseline to Week 40 – Observed Data ANCOVA, ITT Primary Population

	GDNF (N = XXX)		Placebo (N = XXX)	
**Visit**<br>**Statistic**	**Value**	**Change From Baseline**	**Value**	**Change From Baseline**
Week 0 (Baseline)				
n	xx		xx	
Mean (SD)	xx.xx (xx.xxx)		xx.xx (xx.xxx)	
Median	xx.x		xx.x	
Min, Max	xx.x, xx.x		xx.x, xx.x	
Week 40				
n	xx	xx	xx	xx
Mean (SD)	xx.xx (xx.xxx)	xx.xx (xx.xxx)	xx.xx (xx.xxx)	xx.xx (xx.xxx)
Median	xx.x	xx.x	xx.x	xx.x
Min, Max	xx.x, xx.x	xx.x, xx.x	xx.x, xx.x	xx.x, xx.x
Mean difference versus placebo[a] (95% CI)		xx.x (xx.x, xx.x)		
p-value[a]		0.xxxx		

[a] Estimates are from an analysis of covariance (ANCOVA) model with baseline levodopa dose as a covariate and treatment group as a factor.
Note: Only subjects with a Week 40 levodopa value are included in the observed data analysis.
Source: Listing xx.x.xx, Dataset: [NAME], Program: xxxxxx.sas, Output: xxxx.rtf, Generated on: DDMONYYYY HH:MM
Page x of y

Programming Note: See Section 5.0 for definition of levodopa dose.

Table 16.2.4.8 Total Daily Levodopa Equivalent Dose (mg): Change From Baseline to Week 40 – Observed Data ANCOVA, ITT Primary Population

	GDNF (N = XXX)		Placebo (N = XXX)	
**Visit**<br>**Statistic**	**Value**	**Change From Baseline**	**Value**	**Change From Baseline**
Week 0 (Baseline)				
n	xx		xx	
Mean (SD)	xx.xx (xx.xxx)		xx.xx (xx.xxx)	
Median	xx.x		xx.x	
Min, Max	xx.x, xx.x		xx.x, xx.x	
Week 40				
n	xx	xx	xx	Xx
Mean (SD)	xx.xx (xx.xxx)	xx.xx (xx.xxx)	xx.xx (xx.xxx)	xx.xx (xx.xxx)
Median	xx.x	xx.x	xx.x	xx.x
Min, Max	xx.x, xx.x	xx.x, xx.x	xx.x, xx.x	xx.x, xx.x
Mean difference versus placebo[a]				
(95% CI)		xx.x (xx.x, xx.x)		
p-value[a]		0.xxxx		

[a] Estimates are from an analysis of covariance (ANCOVA) model with baseline levodopa equivalent dose as a covariate and treatment group as a factor.
Note: Only subjects with a Week 40 levodopa equivalent value are included in the observed data analysis.
Source: Listing xx.x.xx, Dataset: [NAME], Program: xxxxxx.sas, Output: xxxx.rtf, Generated on: DDMONYYYY HH:MM
Page x of y

Programming Note: See Section 5.0 for definition of levodopa equivalent dose.

16.3 Imaging Tables

Table 16.3.1 Volume of Distribution of Infusate as Determined by Contrast-Enhanced T1-Weighted MRI: Change from Baseline to Week 40 – Observed Data ANCOVA, ITT Primary Population

Programming Note: Repeat observed data ANCOVA Table 16.2.4.4. Visits are Healing phase (Baseline) (last test infusion at the end of the healing phase) and Week 40. The post-infusion time point at each visit is used. Parameter is volume of distribution (in mL), separately for left and right hemispheres, as determined by contrast-enhanced T1-weighted MRI. ***Table will have 2 pages, one for left and right hemisphere; please subtitle each page clearly.***

Table 16.3.2.1 Volume of Interest Coverage as Determined by Contrast-Enhanced T1-Weighted MRI: Change from Baseline to Week 40 – Observed Data ANCOVA, ITT Primary Population

Programming Note: Repeat observed data ANCOVA Table 16.2.4.4. Visits are Healing phase (Baseline) (last test infusion at the end of the healing phase) and Week 40. The post-infusion time point at each visit is used. Parameter is VOI coverage as a percentage of total VOI, separately for left putamen and right putamen and for both putamina combined. ***Table will have 3 pages, one for each of left and right putamen and one for both putamina combined; please subtitle each page clearly.***

Table 16.3.2.2 Total Putamenal Coverage as Determined by Contrast-Enhanced T1-Weighted MRI: Change from Baseline to Week 40 – Observed Data ANCOVA, ITT Primary Population

Programming Note: Repeat observed data ANCOVA Table 16.2.4.4. Visits are Randomization (Baseline) and Week 40. The post-infusion time point at each visit is used. Parameter is total putamenal coverage as a percentage of total putamenal volume, separately for left putamen and right putamen and for both putamina combined. ***Table will have 3 pages, one for each of left and right putamen and one for both putamina combined; please subtitle each page clearly.***

Table 16.3.3.1 ^{18}F-DOPA Uptake as Determined by PET Scan: Change from Baseline to Week 40 – Observed Data ANCOVA, ITT Primary Population

Programming Note: Repeat observed data ANCOVA Table 16.2.4.4. Visits are Week 0 and Week 40. Parameter is ^{18}F-DOPA uptake rate constant determined by PET, separately for left and right hemispheres. Four regions are assessed per hemisphere (anterior, central and posterior putamen, and caudate nucleus). ***Table will have 8 pages, left and right for each region; please subtitle each page clearly.***

Table 16.3.3.2 ^{18}F-DOPA Uptake as Determined by PET Scan: Change from Baseline to Week 40 – Observed Data ANCOVA, ITT Overall Population

Programming Note: Repeat of table above, but with a different population. ***Table will have 8 pages, left and right for each region; please subtitle each page clearly.***

Table 16.3.3.3 ^{18}F-DOPA Uptake as Determined by PET Scan: Change from Baseline to Week 40 – Observed Data ANCOVA, Per-Protocol Population

Programming Note: Repeat of table above, but with a different population. ***Table will have 8 pages, left and right for each region; please subtitle each page clearly.***

Table 16.3.3.4 ^{18}F-DOPA Uptake as Determined by PET Scan: Change from Baseline to Week 12 and Week 40 – Observed Data ANCOVA, ITT Pilot Population

Programming Note: Repeat of table above, but with a different population and 2 postbaseline time points (Week 12 and Week 40). ***Table will have 8 pages, left and right for each region (assuming that 2 postbaseline time points fit on one page); please subtitle each page clearly.***

Table 16.3.4.1 Correlation Analyses of Percentage Change from Baseline to Week 40 in OFF State UPDRS Motor Score (Part III) to Volume of Interest Coverage and Total Putamenal Coverage at Baseline as Determined by Contrast-Enhanced T1-Weighted MRI – ITT Primary Population

Parameters	GDNF<br>(N = XXX)<br>n<br>Spearman Rank Correlation<br>(95% CI)<br>p-value	Placebo<br>(N = XXX)<br>n<br>Spearman Rank Correlation<br>(95% CI)<br>p-value
Percentage change from baseline to Week 40 in OFF state UPDRS motor score (part III)<br><br>versus<br><br>VOI coverage at baseline, both putamina combined	xx<br>0.xxx (0.xxx, 0.xxx)<br>0.xxxx	xx<br>0.xxx (0.xxx, 0.xxx)<br>0.xxxx
Percentage change from baseline to Week 40 in OFF state UPDRS motor score (part III)<br><br>versus<br><br>Total putamenal coverage at baseline, both putamina combined	xx<br>0.xxx (0.xxx, 0.xxx)<br>0.xxxx	xx<br>0.xxx (0.xxx, 0.xxx)<br>0.xxxx

Note: For subject 45, items 22, 27, 28, 29 and 30 are excluded from the score.
Source: Listing xx.x.xx, Dataset: [NAME], Program: xxxxxx.sas, Output: xxxx.rtf, Generated on: DDMONYYYY HH:MM
Page x of y

Table 16.3.4.2 Correlation Analysis of Change from Baseline to Week 40 in NMSS Total Score to Total Putamenal Coverage at Baseline as Determined by Contrast-Enhanced T1-Weighted MRI – ITT Primary Population

Programming Note: Repeat above Table 16.3.4.1 for parameters "Change from baseline to Week 40 in NMSS total score" and "Total putamenal coverage at baseline, both putamina combined." Change footnote regarding subject 45 to: "Data for subject 45 are excluded from analysis."

Table 16.3.4.3 Correlation Analysis of Percentage Change from Baseline to Week 40 in OFF State UPDRS Motor Score (Part III) to Change From Baseline to Week 40 in ^{18}F-DOPA Uptake as Determined by PET Scan - ITT Primary Population

Programming Note: Repeat above Table 16.3.4.1. Parameters are "Percentage change from baseline to Week 40 in OFF state UPDRS motor score (part III)" and "Change from baseline to Week 40 in ^{18}F-DOPA uptake rate constant, both hemispheres combined." Perform separate analyses for each of the four regions (anterior, central and posterior putamen, and caudate nucleus). ***Table will have 4 pages, one for each region; please subtitle each page clearly.***

Table 16.3.4.4 Correlation Analyses of Change from Baseline to Week 40 in ^{18}F-DOPA Uptake as Determined by PET Scan to Volume of Interest Coverage at Baseline as Determined by Contrast-Enhanced T1-Weighted MRI – ITT Primary Population

Programming Note: Repeat above Table 16.3.4.1. Parameters are "Change from baseline to Week 40 in ^{18}F-DOPA uptake rate constant, left hemisphere OR right hemisphere" and "VOI coverage at baseline, left putamen OR right putamen". For ^{18}F-DOPA uptake rate constant, perform analyses separately for each of the four regions (anterior, central and posterior putamen, and caudate nucleus), separately for left and right hemispheres. For VOI coverage, perform analyses separately for left and right putamen. Perform correlations with the ipsilateral side (ie, left compared with left, right compared with right). Delete footnote regarding subject 45. ***Table will have 8 pages, left and right for each region; please subtitle each page clearly.***

Table 16.3.4.5 Correlation Analyses of Change from Baseline to Week 40 in ^{18}F-DOPA Uptake as Determined by PET Scan to Total Putamenal Coverage at Baseline as Determined by Contrast-Enhanced T1-Weighted MRI – ITT Primary Population

Programming Note: Repeat above Table 16.3.4.1. Parameters are "Change from baseline to Week 40 in ^{18}F-DOPA uptake rate constant, left hemisphere OR right hemisphere" and "Total putamenal coverage at baseline, left putamen OR right putamen". For ^{18}F-DOPA uptake rate constant, perform analyses separately for each of the four regions (anterior, central and posterior putamen, and caudate nucleus), separately for left and right hemispheres. For total putamenal coverage, perform analyses separately for left and right putamen. Perform correlations with the ipsilateral side (ie, left compared with left, right compared with right). Delete footnote regarding subject 45. ***Table will have 8 pages, left and right for each region; please subtitle each page clearly.***

16.4 Safety Data Tables

16.4.1 Exposure Data Tables

Table 16.4.1.1.1 Exposure to Study Medication - Safety Primary Population

Variable	GDNF (N = XXX)	Placebo (N = XXX)
Number of infusions of study medication		
n	xx	Xx
Mean (SD)	xx.x (xx.xx)	xx.x (xx.xx)
Median	xx.x	xx.x
Min, Max	xx, xx	xx, xx
Total GDNF exposure[a] (mg)		
n	xx	Xx
Mean (SD)	xx.x (xx.xx)	xx.x (xx.xx)
Median	xx.x	xx.x
Min, Max	xx, xx	xx, xx

[a] Total exposure in mg assumes the entire infusion was completed at each administration.
Source: Listing xx.x.xx, Dataset: [NAME], Program: xxxxxx.sas, Output: T_16_1_1_1_XXXXX.rtf, Generated on: DDMONYYYY HH:MM
Page x of y

Table 16.4.1.1.2 Exposure to Study Medication - Safety Overall Population

Programming Note: Repeat table for different population.

Table 16.4.1.2.1 Study Medication Infusion Details by Visit - Safety Primary Population

Visit<br>Variable	GDNF<br>(N = XXX)	Placebo<br>(N = XXX)
Week 0		
Duration of infusion[a] (minutes)		
n	xx	Xx
Mean (SD)	xx.x (xx.xx)	xx.x (xx.xx)
Median	xx.x	xx.x
Min, Max	xx, xx	xx, xx
Any infusion interruption/early termination [n (%)]		
No	xx (xx.x)	xx (xx.x)
Yes	xx (xx.x)	xx (xx.x)
Missing	xx (xx.x)	xx (xx.x)
Week 4		
…		

[a] Duration of infusion of study medication in minutes is calculated as (infusion end time – infusion start time + 1). Interruptions are not subtracted.
Source: Listing xx.x.xx, Dataset: [NAME], Program: xxxxxx.sas, Output: T_16_1_1_1_XXXXX.rtf, Generated on: DDMONYYYY HH:MM
Page x of y

Programming Note: Continue for all 10 infusion visits: Weeks 0, 4, 8, 12, 16, 20, 24, 28, 32, and 36.

Table 16.4.1.2.2 Study Medication Infusion Details by Visit - Safety Overall Population

Programming Note: Repeat table for different population. Include the additional visits of the Pilot Stage subjects.

16.4.2 Adverse Event Tables

Table 16.4.2.1.1 Overall Summary of Adverse Events - Safety Primary Population

Adverse Event Category	GDNF (N = XXX) n (%)	Placebo (N = XXX) n (%)
Any adverse event	xxx (xx.x)	xxx (xx.x)
Any pre-treatment adverse event	xxx (xx.x)	xxx (xx.x)
Any serious pre-treatment adverse event	xxx (xx.x)	xxx (xx.x)
Any TEAE	xxx (xx.x)	xxx (xx.x)
Any severe TEAE	xxx (xx.x)	xxx (xx.x)
Any serious TEAE	xxx (xx.x)	xxx (xx.x)
Any TEAE leading to permanent discontinuation of study medication	xxx (xx.x)	xxx (xx.x)
Any study medication-related TEAE	xxx (xx.x)	xxx (xx.x)
Any serious study medication-related TEAE	xxx (xx.x)	xxx (xx.x)
Any device-related TEAE	xxx (xx.x)	xxx (xx.x)
Any serious device-related TEAE	xxx (xx.x)	xxx (xx.x)

Note: For each category, subjects are included only once, even if they experienced multiple events in that category. Pre-treatment adverse events are defined as any adverse events starting on or after the date of informed consent but before the first randomized study medication dose date. TEAEs are those adverse events starting on or after the first randomized study medication dose date.
Source: Listing xx.x.xx, Dataset: [NAME], Program: xxxxxx.sas, Output: xxxx.rtf, Generated on: DDMONYYYY HH:MM
Page 1 of 1

Table 16.4.2.1.2 Overall Summary of Adverse Events - Safety Overall Population

Programming Note: Repeat table for different population.

Table 16.4.2.2.1 Treatment-Emergent Adverse Events by System Organ Class and Preferred Term - Safety Primary Population

System Organ Class<br>Preferred Term	GDNF<br>(N = XXX)<br>n (%)	Placebo<br>(N = XXX)<br>n (%)
Subjects with at least one TEAE	xx (xx.x)	xx (xx.x)
[System Organ Class 1]	xx (xx.x)	xx (xx.x)
[Preferred Term 1]	xx (xx.x)	xx (xx.x)
[Preferred Term 2]	xx (xx.x)	xx (xx.x)
[System Organ Class 2]	xx (xx.x)	xx (xx.x)
[Preferred Term 1]	xx (xx.x)	xx (xx.x)
[Preferred Term 2]	xx (xx.x)	xx (xx.x)
…		

Note: Adverse events are coded using MedDRA version 19.0. Only TEAEs are summarized. TEAEs are those adverse events starting on or after the first randomized study medication dose date. For each system organ class and preferred term, subjects are included only once, even if they experienced multiple events in that system organ class or preferred term.
Source: Listing xx.x.xx, Dataset: [NAME], Program: xxxxxx.sas, Output: T_16_1_1_1_XXXXX.rtf, Generated on: DDMONYYYY HH:MM
Page x of y

Programming Note: Sort alphabetically by SOC and then alphabetically for PT.

Table 16.4.2.2.2 Treatment-Emergent Adverse Events by System Organ Class and Preferred Term - Safety Overall Population

Programming Note: Repeat SOC & PT table for different population.

Table 16.4.2.3.1 Treatment-Emergent Adverse Events Experienced by at Least 3 Subjects in Any Treatment Group by Preferred Term - Safety Primary Population

	GDNF (N = XXX)		Placebo (N = XXX)	
**Preferred Term**	**n Subjects (%)**	**n Events**	**n Subjects (%)**	**n Events**
[Preferred Term 1]	xx (xx.x)	xxx	xx (xx.x)	Xxx
[Preferred Term 2]	xx (xx.x)	xxx	xx (xx.x)	Xxx
[Preferred Term 3]	xx (xx.x)	xxx	xx (xx.x)	Xxx
[Preferred Term 4]	xx (xx.x)	xxx	xx (xx.x)	Xxx
[Preferred Term 5]	xx (xx.x)	xxx	xx (xx.x)	Xxx
[Preferred Term 6]	xx (xx.x)	xxx	xx (xx.x)	Xxx
[Preferred Term 7]	xx (xx.x)	xxx	xx (xx.x)	Xxx
[Preferred Term 8]	xx (xx.x)	xxx	xx (xx.x)	Xxx
…				

Note: Adverse events are coded using MedDRA version 19.0. Only TEAEs are summarized. TEAEs are those adverse events starting on or after the first randomized study medication dose date. For each preferred term, subjects are included only once, even if they experienced multiple events in that preferred term.
Source: Listing xx.x.xx, Dataset: [NAME], Program: xxxxxx.sas, Output: T_16_1_1_1_XXXXX.rtf, Generated on: DDMONYYYY HH:MM
Page 1 of 1

Programming Note: Sort alphabetically by PT. Calculation of >=3 should be within GDNF or placebo only, not combined.

Table 16.4.2.3.2 Treatment-Emergent Adverse Events Experienced by at Least 3 Subjects in Any Treatment Group by Preferred Term - Safety Overall Population

Programming Note: Repeat table for different population.

Table 16.4.2.4.1 Treatment-Emergent Adverse Events Experienced by a Subject within 7 Days of at Least 3 Infusion Visits by Preferred Term - Safety Primary Population

	GDNF (N = XXX)			Placebo (N = XXX)		
		Visits			Visits	
Preferred Term	n Subjects (%)	Mean	Range	n Subjects (%)	Mean	Range
[Preferred Term 1]	xx (xx.x)	xxx	XX-XX	xx (xx.x)	xxx	XX-XX
[Preferred Term 2]	xx (xx.x)	xxx	XX-XX	xx (xx.x)	xxx	XX-XX
[Preferred Term 3]	xx (xx.x)	xxx	XX-XX	xx (xx.x)	xxx	XX-XX
[Preferred Term 4]	xx (xx.x)	xxx	XX-XX	xx (xx.x)	xxx	XX-XX
[Preferred Term 5]	xx (xx.x)	xxx	XX-XX	xx (xx.x)	xxx	XX-XX
[Preferred Term 6]	xx (xx.x)	xxx	XX-XX	xx (xx.x)	xxx	XX-XX
[Preferred Term 7]	xx (xx.x)	xxx	XX-XX	xx (xx.x)	xxx	XX-XX
[Preferred Term 8]	xx (xx.x)	xxx	XX-XX	xx (xx.x)	xxx	XX-XX
…						

Note: Adverse events are coded using MedDRA version 19.0. Only TEAEs are summarized. TEAEs are those adverse events starting on or after the first randomized study medication dose date. For each preferred term, subjects are included only once, independent of the number of events they experienced in that preferred term.
Source: Listing xx.x.xx, Dataset: [NAME], Program: xxxxxx.sas, Output: T_16_1_1_1_XXXXX.rtf, Generated on: DDMONYYYY HH:MM
Page 1 of 1

Programming Note: Sort alphabetically by PT. Count the number of visits at which the event occurred after infusion for each subject, then calculate the mean and range across subjects.

Table 16.4.2.4.2 Treatment-Emergent Adverse Events Experienced by a Subject within 7 Days of at Least 3 Infusion Visits by Preferred Term - Safety Overall Population

Programming Note: Repeat table for different population.

Table 16.4.2.5.1 Treatment-Emergent Adverse Events by System Organ Class, Preferred Term, and Maximum Severity - Safety Primary Population

	GDNF (N = XXX)				Placebo (N = XXX)			
**System Organ Class<br>Preferred Term**	**Mild<br>n (%)**	**Moderate<br>n (%)**	**Severe<br>n (%)**	**Total<br>n (%)**	**Mild<br>n (%)**	**Moderate<br>n (%)**	**Severe<br>n (%)**	**Total<br>n (%)**
Subjects with at least one TEAE	xxx (xx.x)	xxx (xx.x)	xxx (xx.x)	xxx (xx.x)	xxx (xx.x)	xxx (xx.x)	xxx (xx.x)	xxx (xx.x)
[System Organ Class 1]	xxx (xx.x)	xxx (xx.x)	xxx (xx.x)	xxx (xx.x)	xxx (xx.x)	xxx (xx.x)	xxx (xx.x)	xxx (xx.x)
[Preferred Term 1]]	xxx (xx.x)	xxx (xx.x)	xxx (xx.x)	xxx (xx.x)	xxx (xx.x)	xxx (xx.x)	xxx (xx.x)	xxx (xx.x)
[Preferred Term 2]	xxx (xx.x)	xxx (xx.x)	xxx (xx.x)	xxx (xx.x)	xxx (xx.x)	xxx (xx.x)	xxx (xx.x)	xxx (xx.x)
[Preferred Term 3]	xxx (xx.x)	xxx (xx.x)	xxx (xx.x)	xxx (xx.x)	xxx (xx.x)	xxx (xx.x)	xxx (xx.x)	xxx (xx.x)
[Preferred Term 4]	xxx (xx.x)	xxx (xx.x)	xxx (xx.x)	xxx (xx.x)	xxx (xx.x)	xxx (xx.x)	xxx (xx.x)	xxx (xx.x)
…								
[System Organ Class 2]	xxx (xx.x)	xxx (xx.x)	xxx (xx.x)	xxx (xx.x)	xxx (xx.x)	xxx (xx.x)	xxx (xx.x)	xxx (xx.x)
[Preferred Term 1]	xxx (xx.x)	xxx (xx.x)	xxx (xx.x)	xxx (xx.x)	xxx (xx.x)	xxx (xx.x)	xxx (xx.x)	xxx (xx.x)
[Preferred Term 2]	xxx (xx.x)	xxx (xx.x)	xxx (xx.x)	xxx (xx.x)	xxx (xx.x)	xxx (xx.x)	xxx (xx.x)	xxx (xx.x)
…								
[System Organ Class 3]	xxx (xx.x)	xxx (xx.x)	xxx (xx.x)	xxx (xx.x)	xxx (xx.x)	xxx (xx.x)	xxx (xx.x)	xxx (xx.x)
…								

Note: Adverse events are coded using MedDRA version 19.0. Only TEAEs are summarized. TEAEs are those adverse events starting on or after the first randomized study medication dose date. For each system organ class and preferred term, subjects are included only once, in the maximum severity, for that TEAE. TEAEs without severity information are counted in the Total column only.
Source: Listing xx.x.xx, Dataset: [NAME], Program: xxxxxx.sas, Output: xxxx.rtf, Generated on: DDMONYYYY HH:MM
Page x of y

Programming Note: Sort alphabetically by SOC and then alphabetically for PT.

Table 16.4.2.5.2 Treatment-Emergent Adverse Events by System Organ Class, Preferred Term, and Maximum Severity - Safety Overall Population

Programming Note: Repeat table for different population.

Table 16.4.2.6.1 Serious Treatment-Emergent Adverse Events by System Organ Class and Preferred Term - Safety Primary Population

Programming Note: Repeat SOC & PT Table 16.4.2.2.1. First row label is “Subjects with at least one serious TEAE.”

Table 16.4.2.6.2 Serious Treatment-Emergent Adverse Events by System Organ Class and Preferred Term - Safety Overall Population

Programming Note: Repeat table for different population.

Table 16.4.2.7.1 Treatment-Emergent Adverse Events Leading to Permanent Discontinuation of Study Medication by System Organ Class and Preferred Term - Safety Primary Population

Programming Note: Repeat SOC & PT Table 16.4.2.2.1. First row label is “Subjects with at least one TEAE leading to permanent discontinuation of study medication.”

Table 16.4.2.7.2 Treatment-Emergent Adverse Events Leading to Permanent Discontinuation of Study Medication by System Organ Class and Preferred Term - Safety Overall Population

Programming Note: Repeat table for different population.

Table 16.4.2.8.1 Study Medication-Related Treatment-Emergent Adverse Events by System Organ Class and Preferred Term - Safety Primary Population

Programming Note: Repeat SOC & PT Table 16.4.2.2.1. First row label is "Subjects with at least one study medication-related TEAE."

Table 16.4.2.8.2 Study Medication-Related Treatment-Emergent Adverse Events by System Organ Class and Preferred Term - Safety Overall Population

Programming Note: Repeat table for different population.

Table 16.4.2.9.1 Serious Study Medication-Related Treatment-Emergent Adverse Events by System Organ Class and Preferred Term - Safety Primary Population

Programming Note: Repeat Table SOC & PT 16.4.2.2.1. First row label is "Subjects with at least one serious study medication-related TEAE."

Table 16.4.2.9.2 Serious Study Medication-Related Treatment-Emergent Adverse Events by System Organ Class and Preferred Term - Safety Overall Population

Programming Note: Repeat table for different population.

Table 16.4.2.10.1 Device-Related Treatment-Emergent Adverse Events by System Organ Class and Preferred Term - Safety Primary Population

Programming Note: Repeat Table SOC & PT 16.4.2.2.1. First row label is "Subjects with at least one device-related TEAE."

Table 16.4.2.10.2 Device-Related Treatment-Emergent Adverse Events by System Organ Class and Preferred Term - Safety Overall Population

Programming Note: Repeat table for different population.

Table 16.4.2.11.1 Serious Device-Related Treatment-Emergent Adverse Events by System Organ Class and Preferred Term - Safety Primary Population

Programming Note: Repeat Table SOC & PT 16.4.2.2.1. First row label is "Subjects with at least one serious device-related TEAE."

Table 16.4.2.11.2 Serious Device-Related Treatment-Emergent Adverse Events by System Organ Class and Preferred Term - Safety Overall Population

Programming Note: Repeat table for different population.

Table 16.4.2.12 Pre-Treatment Adverse Events by System Organ Class and Preferred Term - Safety Enrolled Population

**System Organ Class**<br>**Preferred Term**	**GDNF**<br>**(N = XXX)**<br>**n (%)**	**Placebo**<br>**(N = XXX)**<br>**n (%)**	**Not Randomized**<br>**(N = 2)**<br>**n (%)**	**Total**<br>**(N = XXX)**<br>**n (%)**
Subjects with at least one pre-treatment AE	xx (xx.x)	xx (xx.x)	x (xx.x)	xx (xx.x)
[System Organ Class 1]	xx (xx.x)	xx (xx.x)	x (xx.x)	xx (xx.x)
[Preferred Term 1]	xx (xx.x)	xx (xx.x)	x (xx.x)	xx (xx.x)
[Preferred Term 2]	xx (xx.x)	xx (xx.x)	x (xx.x)	xx (xx.x)
[System Organ Class 2]	xx (xx.x)	xx (xx.x)	x (xx.x)	xx (xx.x)
[Preferred Term 1]	xx (xx.x)	xx (xx.x)	x (xx.x)	xx (xx.x)
[Preferred Term 2]	xx (xx.x)	xx (xx.x)	x (xx.x)	xx (xx.x)
…				

Note: AEs are coded using MedDRA version 19.0. For each system organ class and preferred term, subjects are included only once, even if they experienced multiple events in that system organ class or preferred term. Pre-treatment adverse events are defined as any adverse events starting on or after the date of informed consent but before the first randomized study medication dose date. The Safety Enrolled Population is defined as all enrolled subjects (Primary and Pilot Stages combined), including not randomized subjects.
Source: Listing xx.x.xx, Dataset: [NAME], Program: xxxxxx.sas, Output: T_16_1_1_1_XXXXX.rtf, Generated on: DDMONYYYY HH:MM
Page x of y

Programming Note: Sort alphabetically by SOC and then alphabetically for PT.

Table 16.4.2.13 Serious Pre-Treatment Adverse Events by System Organ Class and Preferred Term - Safety Enrolled Population

Programming Note: Repeat pre-treatment AE Table 16.4.2.12. First row label is "Subjects with at least one serious pre-treatment AE."

Table 16.4.2.14 Device-Related Pre-Treatment Adverse Events by System Organ Class and Preferred Term - Safety Enrolled Population

Programming Note: Repeat pre-treatment AE Table 16.4.2.12. First row label is "Subjects with at least one device-related pre-treatment AE."

Table 16.4.2.15 Serious Device-Related Pre-Treatment Adverse Events by System Organ Class and Preferred Term - Safety Enrolled Population

Programming Note: Repeat pre-treatment AE Table 16.4.2.12. First row label is "Subjects with at least one serious device-related pre-treatment AE."

Table 16.4.2.16.1 Treatment-Emergent Adverse Events of Special Interest by Preferred Term - Safety Primary Population

AESI Category<br>Preferred Term	GDNF<br>(N = XXX)<br>n (%)	Placebo<br>(N = XXX)<br>n (%)
Subjects with at least one treatment-emergent AESI	xx (xx.x)	xx (xx.x)
Dyskinesias	xx (xx.x)	xx (xx.x)
[Preferred Term 1]	xx (xx.x)	xx (xx.x)
[Preferred Term 2]	xx (xx.x)	xx (xx.x)
Falls	xx (xx.x)	xx (xx.x)
[Preferred Term 1]	xx (xx.x)	xx (xx.x)
[Preferred Term 2]	xx (xx.x)	xx (xx.x)
Adverse changes in mood	xx (xx.x)	xx (xx.x)
[Preferred Term 1]	xx (xx.x)	xx (xx.x)
[Preferred Term 2]	xx (xx.x)	xx (xx.x)
Impulsivity	xx (xx.x)	xx (xx.x)
[Preferred Term 1]	xx (xx.x)	xx (xx.x)
[Preferred Term 2]	xx (xx.x)	xx (xx.x)
…		

Note: Adverse events are coded using MedDRA version 19.0. Only TEAEs are summarized. TEAEs are those adverse events starting on or after the first randomized study medication dose date. For each category and preferred term, subjects are included only once, even if they experienced multiple events in that category or preferred term.
Source: Listing xx.x.xx, Dataset: [NAME], Program: xxxxxx.sas, Output: T_16_1_1_1_XXXXX.rtf, Generated on: DDMONYYYY HH:MM
Page 1 of 1

Table 16.4.2.16.2 Treatment-Emergent Adverse Events of Special Interest by Preferred Term - Safety Overall Population

Programming Note: Repeat table for different population.

16.4.3 Laboratory Tables

Table 16.4.3.1.1 Hematology Results by Visit - Safety Primary Population

Parameter (unit)<br>Visit<br>Statistic	GDNF (N = XXX) Value	GDNF (N = XXX) Change From Baseline	Placebo (N = XXX) Value	Placebo (N = XXX) Change From Baseline
Hemoglobin (g/dL)				
Screening (Baseline)				
n	xx		xx	
Mean (SD)	xx.xx (xx.xxx)		xx.xx (xx.xxx)	
Median	xx.x		xx.x	
Min, Max	xx.x, xx.x		xx.x, xx.x	
Week 4				
n	xx	xx	xx	Xx
Mean (SD)	xx.xx (xx.xxx)	xx.xx (xx.xxx)	xx.xx (xx.xxx)	xx.xx (xx.xxx)
Median	xx.x	xx.x	xx.x	xx.x
Min, Max	xx.x, xx.x	xx.x, xx.x	xx.x, xx.x	xx.x, xx.x
Week 16				
n	xx	xx	xx	Xx
Mean (SD)	xx.xx (xx.xxx)	xx.xx (xx.xxx)	xx.xx (xx.xxx)	xx.xx (xx.xxx)
Median	xx.x	xx.x	xx.x	xx.x
Min, Max	xx.x, xx.x	xx.x, xx.x	xx.x, xx.x	xx.x, xx.x
Week 28				
…				

Source: Listing xx.x.xx, Dataset: [NAME], Program: xxxxxx.sas, Output: xxxx.rtf, Generated on: DDMONYYYY HH:MM
Page x of y

Programming Note: Visits are screening and Weeks 4, 16, 28, and 40. See text for parameters. Sort by order of parameters shown in SAP Table 4.

Table 16.4.3.1.2 Shift Summary of Hematology Results by Reference Range and Visit - Safety Primary Population

	GDNF (N = XXX)				Placebo (N = XXX)			
	Postbaseline Value				Postbaseline Value			
**Parameter<br>Visit<br>Baseline Value**	**Low<br>n (%)**	**Normal<br>n (%)**	**High<br>n (%)**	**Missing<br>n (%)**	**Low<br>n (%)**	**Normal<br>n (%)**	**High<br>n (%)**	**Missing<br>n (%)**
Lab Parameter 1								
Week 4								
Low	xxx (xx.x)	xxx (xx.x)	xxx (xx.x)	xxx (xx.x)	xxx (xx.x)	xxx (xx.x)	xxx (xx.x)	xxx (xx.x)
Normal	xxx (xx.x)	xxx (xx.x)	xxx (xx.x)	xxx (xx.x)	xxx (xx.x)	xxx (xx.x)	xxx (xx.x)	xxx (xx.x)
High	xxx (xx.x)	xxx (xx.x)	xxx (xx.x)	xxx (xx.x)	xxx (xx.x)	xxx (xx.x)	xxx (xx.x)	xxx (xx.x)
Missing	xxx (xx.x)	xxx (xx.x)	xxx (xx.x)	xxx (xx.x)	xxx (xx.x)	xxx (xx.x)	xxx (xx.x)	xxx (xx.x)
Week 16								
Low	xxx (xx.x)	xxx (xx.x)	xxx (xx.x)	xxx (xx.x)	xxx (xx.x)	xxx (xx.x)	xxx (xx.x)	xxx (xx.x)
Normal	xxx (xx.x)	xxx (xx.x)	xxx (xx.x)	xxx (xx.x)	xxx (xx.x)	xxx (xx.x)	xxx (xx.x)	xxx (xx.x)
High	xxx (xx.x)	xxx (xx.x)	xxx (xx.x)	xxx (xx.x)	xxx (xx.x)	xxx (xx.x)	xxx (xx.x)	xxx (xx.x)
Missing	xxx (xx.x)	xxx (xx.x)	xxx (xx.x)	xxx (xx.x)	xxx (xx.x)	xxx (xx.x)	xxx (xx.x)	xxx (xx.x)
Week 28								
Low	xxx (xx.x)	xxx (xx.x)	xxx (xx.x)	xxx (xx.x)	xxx (xx.x)	xxx (xx.x)	xxx (xx.x)	xxx (xx.x)
Normal	xxx (xx.x)	xxx (xx.x)	xxx (xx.x)	xxx (xx.x)	xxx (xx.x)	xxx (xx.x)	xxx (xx.x)	xxx (xx.x)
High	xxx (xx.x)	xxx (xx.x)	xxx (xx.x)	xxx (xx.x)	xxx (xx.x)	xxx (xx.x)	xxx (xx.x)	xxx (xx.x)
Missing	xxx (xx.x)	xxx (xx.x)	xxx (xx.x)	xxx (xx.x)	xxx (xx.x)	xxx (xx.x)	xxx (xx.x)	xxx (xx.x)
…								

Note: Percentages are based on the total number of observations for that visit.
Source: Listing xx.x.xx, Dataset: [NAME], Program: xxxxxx.sas, Output: xxxx.rtf, Generated on: DDMONYYYY HH:MM
Page x of y

Programming Note: Visits are screening and Weeks 4, 16, 28, and 40. See text for parameters. Sort by order of parameters shown in SAP Table 4.

Table 16.4.3.1.3 Clinically Significant Postbaseline Hematology Results - Safety Primary Population

Parameter	GDNF (N = XXX) n (%)	Placebo (N = XXX) n (%)
[Lab parameter 1 – high]	xxx (xx.x)	xxx (xx.x)
[Lab parameter 2]	xxx (xx.x)	xxx (xx.x)
[Lab parameter 3]	xxx (xx.x)	xxx (xx.x)
[Lab parameter 4]	xxx (xx.x)	xxx (xx.x)
[Lab parameter 5]	xxx (xx.x)	xxx (xx.x)
[Lab parameter 6]	xxx (xx.x)	xxx (xx.x)
[Lab parameter 7]	xxx (xx.x)	xxx (xx.x)
[Lab parameter 8]	xxx (xx.x)	xxx (xx.x)
…		

Note: Results were rated by the investigator as clinically significant on the CRF based on medical judgment, not using any pre-specified numerical criteria. For each parameter, subjects are included only once, even if they experienced more than one clinically significant result.
Source: Listing xx.x.xx, Dataset: [NAME], Program: xxxxxx.sas, Output: xxxx.rtf, Generated on: DDMONYYYY HH:MM
Page x of y

Programming Note: See text for parameters. Sort by order of parameters shown in SAP Table 4.

Table 16.4.3.2.1 Serum Chemistry Results by Visit - Safety Primary Population

Programming Note: Repeat hematology by visit Table 16.4.3.1.1 for serum chemistry parameters.Add the footnote “Note: For ALT and total bilirubin, any results recorded in the format <X or >X are included in tables as a value of X.”

Table 16.4.3.2.2 Shift Summary of Serum Chemistry Results by Reference Range and Visit - Safety Primary Population

Programming Note: Repeat hematology shift Table 16.4.3.1.2 for serum chemistry parameters.

Table 16.4.3.2.3 Clinically Significant Postbaseline Serum Chemistry Results - Safety Primary Population

Programming Note: Repeat clinically significant hematology Table 16.4.3.1.3 for serum chemistry parameters.

16.4.4 Anti-GDNF Antibody Tables

Table 16.4.4.1.1 Anti-GDNF Binding Antibodies by Visit - Safety Primary Population

Visit<br>Variable	GDNF<br>(N = XXX)<br>n (%)	Placebo<br>(N = XXX)<br>n (%)
All visits		
All Negative	xxx (xx.x)	xxx (xx.x)
1 Positive	xxx (xx.x)	xxx (xx.x)
> 1 Positive	xxx (xx.x)	xxx (xx.x)
Screening (Baseline)		
Positive	xxx (xx.x)	xxx (xx.x)
Negative	xxx (xx.x)	xxx (xx.x)
Not done	xxx (xx.x)	xxx (xx.x)
Week 4		
Positive	xxx (xx.x)	xxx (xx.x)
Negative	xxx (xx.x)	xxx (xx.x)
Not done	xxx (xx.x)	xxx (xx.x)
Week 16		
Positive	xxx (xx.x)	xxx (xx.x)
Negative	xxx (xx.x)	xxx (xx.x)
Not done	xxx (xx.x)	xxx (xx.x)
…		

Source: Listing xx.x.xx, Dataset: [NAME], Program: xxxxxx.sas, Output: xxxx.rtf, Generated on: DDMONYYYY HH:MM
Page x of y

Programming Note: Visits are screening and Weeks 4, 16, 28, 40, and All visits.

Table 16.4.4.1.2 Anti-GDNF Binding Antibodies by Visit - Safety Overall Population

Programming Note: Repeat table for different population.

Table 16.4.4.2.1 Anti-GDNF Neutralizing Antibodies by Visit Safety Primary Population

Programming Note: Repeat table for neutralizing antibodies.

Table 16.4.4.2.2 Anti-GDNF Neutralizing Antibodies by Visit - Safety Overall Population

Programming Note: Repeat table for different population.

16.4.5 Plasma GDNF Concentration Tables

Table 16.4.5.1 Plasma GDNF Concentrations by Visit - Safety Primary Population
Programming Note: Repeat hematology by visit Table 16.4.3.1.1 for plasma GDNF concentration (unit). Visits are screening and Weeks 4, 16, 28, and 40.

Table 16.4.5.2 Plasma GDNF Concentrations by Visit - Safety Overall Population
Programming Note: Repeat table for different population.

16.4.6 VITAL SIGN TABLE

Table 16.4.6 Clinically Relevant Postbaseline Abnormalities in Vital Sign Results - Safety Primary Population

**Parameter and Criterion**<br>**..Visit**<br>**Time Point**	**GDNF**<br>**(N = XXX)**<br>**n (%)**	**Placebo**<br>**(N = XXX)**<br>**n (%)**
[VS parameter and criterion 1] at any visit	xxx (xx.x)	xxx (xx.x)
[Visit 1]	xxx (xx.x)	xxx (xx.x)
[Time point 1]	xxx (xx.x)	xxx (xx.x)
[Time point 2]	xxx (xx.x)	xxx (xx.x)
…		

Note: bpm = beats per minute. For each parameter and criterion, visit, and time point, subjects are included only once, even if they experienced more than one clinically relevant abnormality. For the comparisons of values during or after infusion with pre-infusion values, the baseline is the pre-infusion value.
Source: Listing xx.x.xx, Dataset: [NAME], Program: xxxxxx.sas, Output: xxxx.rtf, Generated on: DDMONYYYY HH:MM
Page x of y

Programming Note: Only include those VS parameters and criteria that have at least one clinically relevant abnormality present in the data. This table should include test infusion visits (healing phase visits, interim visits after catheter repositioning, and Week 40 visits) as well as study medication infusion visits.

16.4.7 Body Weight Table

Table 16.4.7 Body Weight by Visit - Safety Primary Population

	GDNF (N = XXX)		Placebo (N = XXX)	
**Visit**<br>**Statistic**	**Value**	**Change From Baseline**	**Value**	**Change From Baseline**
Week 0 (Baseline)				
n	xx		xx	
Mean (SD)	xx.xx (xx.xxx)		xx.xx (xx.xxx)	
Median	xx.x		xx.x	
Min, Max	xx.x, xx.x		xx.x, xx.x	
Week 8				
n	xx	xx	xx	Xx
Mean (SD)	xx.xx (xx.xxx)	xx.xx (xx.xxx)	xx.xx (xx.xxx)	xx.xx (xx.xxx)
Median	xx.x	xx.x	xx.x	xx.x
Min, Max	xx.x, xx.x	xx.x, xx.x	xx.x, xx.x	xx.x, xx.x
p-value[a]		0.xxx		
Week 16				
n	xx	xx	xx	Xx
Mean (SD)	xx.xx (xx.xxx)	xx.xx (xx.xxx)	xx.xx (xx.xxx)	xx.xx (xx.xxx)
Median	xx.x	xx.x	xx.x	xx.x
Min, Max	xx.x, xx.x	xx.x, xx.x	xx.x, xx.x	xx.x, xx.x
p-value[a]		0.xxx		
Week 24				
…				

a P-value based on a paired two-sample t-test.
Source: Listing xx.x.xx, Dataset: [NAME], Program: xxxxxx.sas, Output: xxxx.rtf, Generated on: DDMONYYYY HH:MM
Page x of y

Programming Note: Visits are Week 0 (baseline) and Weeks 8, 16, 24, 32, and 40.

16.4.8 ELECTROCARDIOGRAM TABLES

Table 16.4.8.1 Electrocardiogram Results at Week 40 - Safety Primary Population

Programming Note: Repeat hematology by visit Table 16.4.3.1.1 for ECG parameters. Parameters are heart rate (beats/min), PR interval (ms), QRS interval (ms), QT interval (ms), and QTc interval (ms). Visits are screening and Week 40. Add the following footnote;
Note: Hodges QT correction formula was used.

Table 16.4.8.2 Summary of Electrocardiogram Results at Week 40 - Safety Primary Population

ECG Evaluation	GDNF (N = XXX) n (%)	Placebo (N = XXX) n (%)
Overall impression		
Normal Week 40 result	xxx (xx.x)	xxx (xx.x)
Any abnormal Week 40 result	xxx (xx.x)	xxx (xx.x)
Any clinically significant abnormal Week 40 result	xxx (xx.x)	xxx (xx.x)
Any clinically relevant abnormal Week 40 QTc interval result based on criteria below	xxx (xx.x)	xxx (xx.x)
[Criterion 1]	xxx (xx.x)	xxx (xx.x)
[Criterion 2]	xxx (xx.x)	xxx (xx.x)
…		

Note: Overall ECG impression was rated by the investigator as abnormal and clinically significant on the CRF based on medical judgment. Hodges QT correction formula was used.

Source: Listing xx.x.xx, Dataset: [NAME], Program: xxxxxx.sas, Output: xxxx.rtf, Generated on: DDMONYYYY HH:MM
Page x of y

Programming Note: Present all QTc parameters even if no subjects had an abnormal result.

16.4.9 Glasgow Coma Scale Table

Table 16.4.9 Glasgow Coma Scale Score = 15 or < 15 During or After Infusion by Visit and Time Point - Safety Primary Population

**Visit and Time Point**<br>**Glasgow Coma Scale Score**	**GDNF**<br>**(N = XXX)**<br>**n (%)**	**Placebo**<br>**(N = XXX)**<br>**n (%)**
Healing phase at any time during or after infusion		
All scores = 15	xx (xx.x)	xx (xx.x)
Any score < 15	xx (xx.x)	xx (xx.x)
No score < 15 but at least one score is missing	xx (xx.x)	xx (xx.x)
Week 0 at any time during or after infusion		
All scores = 15	xx (xx.x)	xx (xx.x)
Any score < 15	xx (xx.x)	xx (xx.x)
No score < 15 but at least one score is missing	xx (xx.x)	xx (xx.x)
Week 4 at any time during or after infusion		
…		

Note: Glasgow Coma Scale items include visual response, verbal ability, and motor skills. Each item has 4-6 possible responses. The best possible total score is 15.
Source: Listing xx.x.xx, Dataset: [NAME], Program: xxxxxx.sas, Output: xxxx.rtf, Generated on: DDMONYYYY HH:MM
Page x of y

Programming Note: Present for all test infusion visits and study medication infusion visits. Add information in Table 7 as an endnote.

16.4.10 Other Safety Tables

Table 16.4.10.1 QUIP by Visit - Safety Primary Population

	GDNF (N = XXX)			Placebo (N = XXX)		
	Postbaseline Value			Postbaseline Value		
**Assessor**<br>**Parameter**<br>**Visit**<br>**Baseline Value**	**Positive n (%)**	**Negative n (%)**	**Missing n (%)**	**Positive n (%)**	**Negative n (%)**	**Missing n (%)**
Subject assessment						
Issue with too much gambling						
Week 0 (Baseline)						
Positive	xxx (xx.x)	xxx (xx.x)	xxx (xx.x)	xxx (xx.x)	xxx (xx.x)	xxx (xx.x)
Negative	xxx (xx.x)	xxx (xx.x)	xxx (xx.x)	xxx (xx.x)	xxx (xx.x)	xxx (xx.x)
Missing	xxx (xx.x)	xxx (xx.x)	xxx (xx.x)	xxx (xx.x)	xxx (xx.x)	xxx (xx.x)
Week 8						
Positive	xxx (xx.x)	xxx (xx.x)	xxx (xx.x)	xxx (xx.x)	xxx (xx.x)	xxx (xx.x)
Negative	xxx (xx.x)	xxx (xx.x)	xxx (xx.x)	xxx (xx.x)	xxx (xx.x)	xxx (xx.x)
Missing	xxx (xx.x)	xxx (xx.x)	xxx (xx.x)	xxx (xx.x)	xxx (xx.x)	xxx (xx.x)
Week 16						
Positive	xxx (xx.x)	xxx (xx.x)	xxx (xx.x)	xxx (xx.x)	xxx (xx.x)	xxx (xx.x)
Negative	xxx (xx.x)	xxx (xx.x)	xxx (xx.x)	xxx (xx.x)	xxx (xx.x)	xxx (xx.x)
Missing	xxx (xx.x)	xxx (xx.x)	xxx (xx.x)	xxx (xx.x)	xxx (xx.x)	xxx (xx.x)
…						

Note: The QUIP is a self-administered or informant-completed scale that includes 13 questions covering symptoms related to the 4 commonest impulse control disorders in PD, other behaviors, and problematic use of medication. The number of “Yes” responses is compared to the number required for a positive result. Missing individual responses are imputed using LOCF. Each impulse control disorder, other behavior, and problematic use of medication is rated as a positive result (ie, present) or a negative result (ie, not present). Percentages are based on the total number of observations for that visit.
Source: Listing xx.x.xx, Dataset: [NAME], Program: xxxxxx.sas, Output: xxxx.rtf, Generated on: DDMONYYYY HH:MM
Page x of y

Programming Note: Visits are Week 0 (baseline) and Weeks 8, 16, 24, 32, and 40. Assessor includes subject assessment and informant assessment. Parameters (30) include: Issue with too much gambling, issue with too much sex, issue with too much buying, issue with too much eating,
Think too much about gambling, think too much about sex, think too much about buying, think too much about eating,
Excessive or distressing urge for gambling, excessive or distressing urge for sex, excessive or distressing urge for buying, excessive or distressing urge for eating,

Difficulty controlling gambling, difficulty controlling sex, difficulty controlling buying, difficulty controlling eating,
Engage in activities specifically to continue gambling, engage in activities specifically to continue sex, engage in activities specifically to continue buying, engage in activities specifically to continue eating,
Spend too much time on specific tasks hobbies or other organized activities, spend too much time repeating certain simple motor activities, spend too much time walking or driving with no intended goal or specific purpose, difficulty controlling the amount of time spent on these activities, activities interfere with daily functioning or cause relationship or work difficulties,
Consistently take too much PD medications, increased PD medications without medical advice for psychological reasons, increased PD medications without medical advice because only feel fully "on" when dyskinetic, difficulty controlling use of PD medications, hoard or hide PD medications to increase overall dosage.

Table 16.4.10.2 MoCA at Week 40 - Safety Primary Population

Programming Note: Repeat Table 16.4.3.1.1 (hematology by visit) for MoCA total score. Visits are screening, pre-test infusion (baseline) and Week 40. Add footnote: "Note: The MoCA is a rater-administered cognitive screening tool with 8 components. The total score ranges from 0 to 30, with lower scores representing poorer cognitive function. A total score of 26 or above is considered normal. Missing individual scores are imputed using LOCF if necessary."

Table 16.4.10.3 MDRS at Week 40 - Safety Primary Population

Programming Note: Repeat Table 16.4.3.1.1 (hematology by visit) for MDRS total score. Visits are screening (baseline) and Week 40. Add footnote: "Note: The MDRS is a rater-administered global scale of cognition including 5 subscales. The total score ranges from 0 to 144, with higher scores representing better cognitive function. A total score lower than 123 is associated with some degree of dementia in PD. The AEMSS total score ranges from 0 to 20, with higher scores representing better cognitive function. Individual missing items are imputed using the average of non-missing scores in each subscale if necessary."

Table 16.4.10.4 Stroop Test at Week 40, Safety Primary Population

Programming Note: Repeat Table 16.4.3.1.1 (hematology by visit) for Stroop test parameters. The parameters are the 4 conditions: color naming, word reading, inhibition, and inhibition/switching. Visits are screening and Week 40. Add footnote: "Note: The Stroop test is a global scale of reaction time including 4 conditions. Total time to complete the test in each condition can range from 0 to 999 seconds, with lower time representing better reaction time."

Table 16.4.10.5 FrSBe at Week 40 - Safety Primary Population

Programming Note: Repeat Table 16.4.3.1.1 (hematology by visit) for FrSBe parameters. The parameters are the 3 subscales: apathy, disinhibition, and executive dysfunction. Total scores are not calculated. Visits are screening "before", screening "after" (baseline), and Week 40 "after." Add footnote: "Note: The FrSBe is a scale that assesses behavior related to frontal systems damage including 3 subscales. Higher subscale scores indicate greater pathology. Individual missing items are imputed using the average of non-missing scores in each subscale."

Table 16.4.10.6 Deary-Liewald Four-Choice Reaction Time by Visit - Safety Primary Population

Programming Note: Repeat Table 16.4.3.1.1 (hematology by visit) for RT parameters. Visits are screening (baseline) and Weeks 12, 24, and 40. Add footnote: "Note: The Deary-Liewald RT is a computerized measure of simple and four-choice reaction time. The parameter is the mean reaction time, variance and SD for correct responses for four-choice reaction time. A shorter reaction time is better."

Table 16.4.10.7 Verbal Fluency Assessment at Week 40 - Safety Primary Population

Programming Note: Repeat Table 16.4.3.1.1 (hematology by visit) for verbal fluency parameters. Parameters are phonemic and semantic verbal fluency. Visits are screening (baseline) and Week 40. Add footnote: "Note: The verbal fluency assessment measures verbal functioning in 2 categories. Scores represent number of correct words in one minute and range from 0 to 200. Higher scores represent better verbal functioning."

Table 16.4.10.8 BDI at Week 40 - Safety Primary Population

Programming Note: Repeat Table 16.4.3.1.1 (hematology by visit) for BDI parameters. The parameter is the total score. Visits are screening (baseline) and Week 40. Add footnote: "Note: The BDI is a self-administered test which consists of 21 questions that measure the severity of depression. Scores range from 0 to 63, with higher scores representing worse depression. Individual missing items are imputed using the average of non-missing scores in each subscale."

Table 16.4.10.9 UPSIT at Week 40 - Safety Primary Population

Programming Note: Repeat Table 16.4.3.1.1 (hematology by visit) for UPSIT parameters. The parameter is the number of correct responses out of 40 total items. *Visits are screening (baseline) and Week 40. Add footnote: "Note: The UPSIT is a self-administered test which can be used to identify and quantitate olfactory dysfunction in PD. The number of correct responses out of 40 total items constitutes a subject's score. Lower scores represent greater olfactory dysfunction. Individual missing responses are imputed as zeros (ie, incorrect responses)." Add information in SAP Table 8 as an endnote.*

16.5 FIGURES

Figure 16.5.1.1 OFF State UPDRS Motor Score (Part III): Percentage Change Over Time - ITT Primary Population

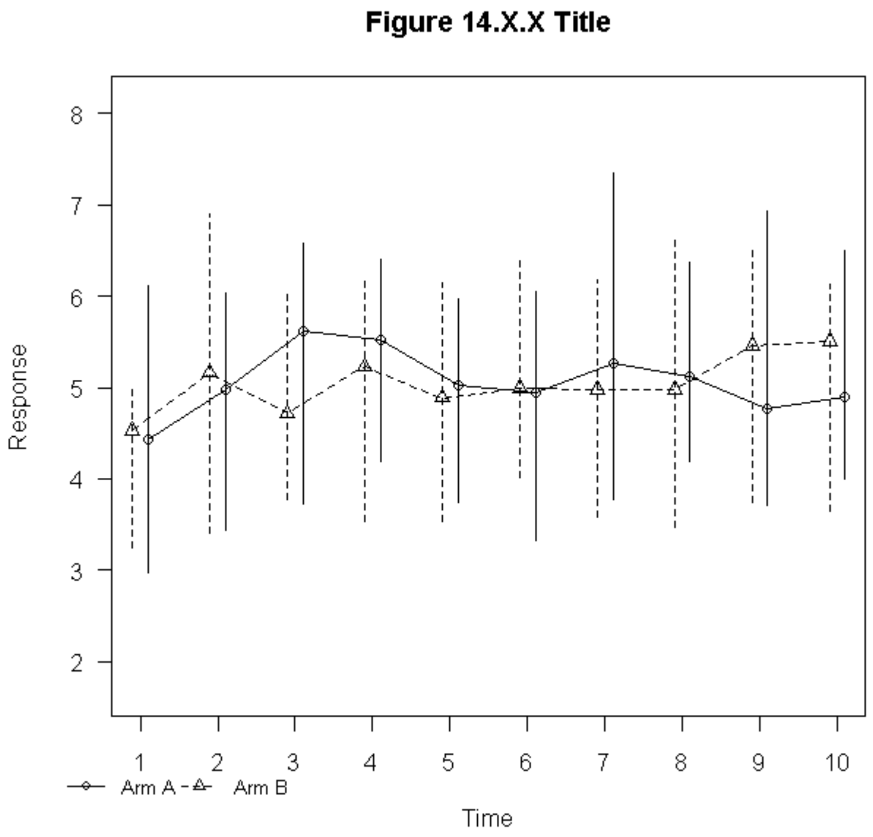

Note: Data points represent means, and error bars represent standard errors. For subject 45, items 22, 27, 28, 29 and 30 are excluded from the score.
Programming Note: Replace with appropriate titles, labels (eg, for y-axis "Change in OFF state UPDRS Motor Score from baseline [unit]"), and legend. Visits are Weeks 0, 8, 16, 24, 32, and 40.

Figure 16.5.1.2 ON State UPDRS Motor Score (Part III): Percentage Change Over Time - ITT Primary Population
Programming Note: Repeat figure above for ON state UPDRS motor score (part III). Visits are Weeks 0, 8, 16, 24, 32, and 40.

Figure 16.5.1.3 OFF State UPDRS Total Score: Percentage Change Over Time - ITT Primary Population
Programming Note: Repeat figure above for OFF state UPDRS total score. Visits are Weeks 0, 8, 16, 24, 32, and 40. Change footnote regarding subject 45 to: "Data for Subject 45 are excluded from analysis."

Figure 16.5.1.4 ON State UPDRS Total Score: Percentage Change Over Time - ITT Primary Population
Programming Note: Repeat figure above for ON state UPDRS total score. Visits are Weeks 0, 8, 16, 24, 32, and 40. Change footnote regarding subject 45 to: "Data for Subject 45 are excluded from analysis."

Figure 16.5.2.1 Motor Fluctuation Diary Total OFF Time Per Day (Hours): Change Over Time - ITT Primary Population
Programming Note: Repeat figure above for OFF time per day (hours). Visits are Weeks 0, 8, 16, 24, 32, and 40. Delete footnote regarding subject 45.

Figure 16.5.2.2 Motor Fluctuation Diary Total Good-Quality ON Time Per Day (Hours): Change Over Time - ITT Primary Population
Programming Note: Repeat figure above for good-quality ON time per day (hours). Visits are Weeks 0, 8, 16, 24, 32, and 40. Delete footnote regarding subject 45. Add footnote "Note: Total good-quality ON time per day is defined as the sum of ON time per day without dyskinesias + ON time per day with non-troublesome dyskinesias."

Figure 16.5.3.1 OFF State Timed Tapping Test (Left Hand): Change Over Time - ITT Primary Population
Programming Note: Repeat figure above for OFF state timed tapping test in left hand (number of taps). Visits are baseline, Weeks 8, 16, 24, 32, and 40. Delete footnote regarding subject 45.

Figure 16.5.3.2 OFF State Timed Tapping Test (Right Hand): Change Over Time - ITT Primary Population
Programming Note: Repeat figure above for OFF state timed tapping test in right hand (number of taps). Visits are baseline, Weeks 8, 16, 24, 32, and 40. Delete footnote regarding subject 45.

Figure 16.5.3.3 ON State Timed Tapping Test (Left Hand): Change Over Time - ITT Primary Population
Programming Note: Repeat figure above for ON state timed tapping test in left hand (number of taps). Visits are baseline, Weeks 8, 16, 24, 32, and 40. Delete footnote regarding subject 45.

Figure 16.5.3.4 ON State Timed Tapping Test (Right Hand): Change Over Time - ITT Primary Population
Programming Note: Repeat figure above for ON state timed tapping test in right hand (number of taps). Visits are baseline, Weeks 8, 16, 24, 32, and 40. Delete footnote regarding subject 45.

Figure 16.5.4.1 OFF State UPDRS Motor Score (Part III): Subject Scores Over Time - ITT Primary Population

Figure	16.5.4.1
Title 1	OFF State UPDRS Motor Score (Part III): Subject Scores Over Time
Title 2	ITT Primary Population
Type of graph	Lineplot
y-axis	Individual subject OFF State UPDRS Motor Score
y-axis (label)	OFF State UPDRS Motor Score
x-axis	Visit
x-axis (label)	Visit
Legend (if applicable)	Not applicable
Footnote 1	Note: For subject 45, items 22, 27, 28, 29 and 30 are excluded from the score.
Footnote 2	
Additional information	GDNF and placebo groups should be on separate pages, keeping the y-axis scale the same for both

Figure 16.5.5.1 OFF State Timed Tapping Test (Left Hand): Subject Values Over Time - ITT Primary Population

Programming Note: Repeat figure above for OFF state timed tapping test in left hand (number of taps). Visits are baseline, Weeks 8, 16, 24, 32, and 40. Delete footnote regarding subject 45.

Figure 16.5.5.2 OFF State Timed Tapping Test (Right Hand): Subject Values Over Time - ITT Primary Population

Programming Note: Repeat figure above for OFF state timed tapping test in right hand (number of taps). Visits are baseline, Weeks 8, 16, 24, 32, and 40. Delete footnote regarding subject 45.

Figure 16.5.5.3 ON State Timed Tapping Test (Left Hand): Subject Values Over Time - ITT Primary Population

Programming Note: Repeat figure above for ON state timed tapping test in left hand (number of taps). Visits are baseline, Weeks 8, 16, 24, 32, and 40. Delete footnote regarding subject 45.

Figure 16.5.5.4 ON State Timed Tapping Test (Right Hand): Subject Values Over Time - ITT Primary Population

Programming Note: Repeat figure above for ON state timed tapping test in right hand (number of taps). Visits are baseline, Weeks 8, 16, 24, 32, and 40. Delete footnote regarding subject 45.

Figure 16.5.6.1 Correlation Analysis of Percentage Change from Baseline to Week 40 in OFF State UPDRS Motor Score (Part III) to Volume of Interest Coverage at Baseline as Determined by Contrast-Enhanced T1-Weighted MRI - ITT Primary Population

Figure	16.5.6.1
Title 1	Correlation Analysis of Percentage Change from Baseline to Week 40 in OFF State UPDRS Motor Score (Part III) to Volume of Interest Coverage at Baseline as Determined by Contrast-Enhanced T1-Weighted MRI
Title 2	ITT Primary Population
Type of graph	Scatterplot
y-axis	Change From Baseline to Week 40 in OFF State UPDRS Motor Score (Part III) (%)
y-axis (label)	Change From Baseline to Week 40 in OFF State UPDRS Motor Score (Part III) (%)
x-axis	Volume of Interest Coverage at Baseline, Both Putamina Combined (%)
x-axis (label)	Volume of Interest Coverage at Baseline, Both Putamina Combined (%)
Legend (if applicable)	Treatment group symbol (please include N’s)
Footnote 1	Note: Analysis uses non-parametric Spearman rank correlation. For subject 45, items 22, 27, 28, 29 and 30 are excluded from the score.
Footnote 2	
Additional information	Include estimated correlation coefficient value and p-value.

Figure 16.5.6.2 Correlation Analysis of Percentage Change from Baseline to Week 40 in OFF State UPDRS Motor Score (Part III) to Total Putamenal Coverage at Baseline as Determined by Contrast-Enhanced T1-Weighted MRI - ITT Primary Population

Figure	16.5.6.2
Title 1	Correlation Analysis of Percentage Change from Baseline to Week 40 in OFF State UPDRS Motor Score (Part III) to Total Putamenal Coverage at Baseline as Determined by Contrast-Enhanced T1-Weighted MRI
Title 2	ITT Primary Population
Type of graph	Scatterplot
y-axis	Change From Baseline to Week 40 in OFF State UPDRS Motor Score (Part III) (%)
y-axis (label)	Change From Baseline to Week 40 in OFF State UPDRS Motor Score (Part III) (%)
x-axis	Total Putamenal Coverage at Baseline, Both Putamina Combined (%)
x-axis (label)	Total Putamenal Coverage at Baseline, Both Putamina Combined (%)
Legend (if applicable)	Treatment group symbol (please include N's)
Footnote 1	Note: Analysis uses non-parametric Spearman rank correlation. For subject 45, items 22, 27, 28, 29 and 30 are excluded from the score.
Footnote 2	
Additional information	Include estimated correlation coefficient value and p-value.

Figure 16.5.6.3 Correlation Analysis of Change From Baseline to Week 40 in NMSS Total Score to Total Putamenal Coverage at Baseline as Determined by Contrast-Enhanced T1-Weighted MRI - ITT Primary Population

Figure	16.5.6.3
Title 1	Correlation Analysis of Change From Baseline to Week 40 in NMSS Total Score to Total Putamenal Coverage at Baseline as Determined by Contrast-Enhanced T1-Weighted MRI
Title 2	ITT Primary Population
Type of graph	Scatterplot
y-axis	Change From Baseline to Week 40 in NMSS Total Score (%)
y-axis (label)	Change From Baseline to Week 40 in NMSS Total Score (%)
x-axis	Total Putamenal Coverage at Baseline, Both Putamina Combined (%)
x-axis (label)	Total Putamenal Coverage at Baseline, Both Putamina Combined (%)
Legend (if applicable)	Treatment group symbol (please include N’s)
Footnote 1	Note: Analysis uses non-parametric Spearman rank correlation. Data for subject 45 are excluded from analysis.
Footnote 2	
Additional information	Include estimated correlation coefficient value and p-value.

Figure 16.5.6.4 Correlation Analysis of Percentage Change from Baseline to Week 40 in OFF State UPDRS Motor Score (Part III) to Change From Baseline to Week 40 in ^{18}F-DOPA Uptake as Determined by PET Scan - ITT Primary Population

Programming Note: Repeat above figures. Parameters are "Percentage change from baseline to Week 40 in OFF state UPDRS motor score (part III)" and "Change from baseline to Week 40 in ^{18}F-DOPA uptake rate constant, both hemispheres combined". Perform separate analyses for each of the four regions (anterior, central and posterior putamen, and caudate nucleus). Change footnote regarding subject 45 to: "For subject 45, items 22, 27, 28, 29 and 30 are excluded from the score." ***Figure will have 4 pages, one for each of 4 regions; please subtitle each page clearly.***

Figure 16.5.6.5 Correlation Analysis of Change From Baseline to Week 40 in ^{18}F-DOPA Uptake as Determined by PET Scan to Volume of Interest Coverage at Baseline as Determined by Contrast-Enhanced T1-Weighted MRI - ITT Primary Population

Programming Note: Repeat above figures. Parameters are "Change from baseline to Week 40 in ^{18}F-DOPA uptake rate constant, left hemisphere OR right hemisphere" and "VOI coverage at baseline, left putamen OR right putamen". For ^{18}F-DOPA uptake rate constant, perform analyses separately for each of the four regions (anterior, central and posterior putamen, and caudate nucleus), separately for left and right hemispheres. For VOI coverage, perform analyses separately for left and right putamen. Perform correlations with the ipsilateral side (ie, left compared with left, right compared with right). Delete footnote regarding subject 45. ***Figure will have 8 pages, one for left and right for each of 4 regions; please subtitle each page clearly.***

Figure 16.5.6.6 Correlation Analysis of Change From Baseline to Week 40 in ^{18}F-DOPA Uptake as Determined by PET Scan to Total Putamenal Coverage at Baseline as Determined by Contrast-Enhanced T1-Weighted MRI - ITT Primary Population

Programming Note: Repeat above figures. Parameters are "Change from baseline to Week 40 in ^{18}F-DOPA uptake rate constant, left hemisphere OR right hemisphere" and "Total putamenal coverage at baseline, left putamen OR right putamen". For ^{18}F-DOPA uptake rate constant, perform analyses separately for each of the four regions (anterior, central and posterior putamen, and caudate nucleus), separately for left and right hemispheres. For total putamenal coverage, perform analyses separately for left and right putamen. Perform correlations with the ipsilateral side (ie, left compared with left, right compared with right). Delete footnote regarding subject 45. ***Figure will have 8 pages, one for left and right for each of 4 regions; please subtitle each page clearly.***

17 Data Listings

17.2.1 Baseline Listings

Listing 17.2.1.1 Randomization Assignments - ITT Overall Population

ITT Pilot Stage (N=XXX)

Subject ID<br>Age/Race/Ethnicity/Sex	Date of<br>Randomization	Randomized Treatment Group	Actual Treatment Group[a]
xxxxxxxxxx/xx/x/x/x	DDMMMYYYY	GDNF	GDNF
xxxxxxxxxx/xx/x/x/x	DDMMMYYYY	Placebo	GDNF

[a] Actual treatment group is GDNF if any amount of GDNF was infused.
Note: W = white; B = black; P = native Hawaiian or other Pacific islander; A = Asian; I = American Indian or Alaska native; H = Hispanic or Latino; N = not Hispanic or Latino; M = male; F = female.
Source: Dataset: [NAME], Program: xxxxxx.sas, Output: xxxx.rtf, Generated on: DDMONYYYY HH:MM
Page x of y

Programming Note: Repeat listing for ITT Primary Stage (N=XXX). Sort by stage and subject ID. Include all ITT Overall subjects.

Listing 17.2.1.2.1 Study Completion Status - ITT Overall Population

ITT Pilot Stage (N=XXX)

GDNF (N=XXX)

Subject ID Age/Race/Ethnicity/ Sex	Completed Study?	Last visit	Date of Last Dose of Study Medication	Date of Discontinuation	Primary Reason for Discontinuation
xxxxxxxxxx/xx/x/x/x	Yes/No	Week XX, DDMMMYYYY	DDMMMYYYY	DDMMMYYYY	Xxxxxxxxxxxxxxxxxxxxxxxxxxxxxxxxxxxxxxxxxx

Note: W = white; B = black; P = native Hawaiian or other Pacific islander; A = Asian; I = American Indian or Alaska native; H = Hispanic or Latino; N = not Hispanic or Latino; M = male; F = female.
Source: Dataset: [NAME], Program: xxxxxx.sas, Output: xxxx.rtf, Generated on: DDMONYYYY HH:MM
Page x of y

Programming Note: Repeat listing for ITT Pilot Stage (N=XXX) Placebo (N=XXX), then ITT Primary Stage (N=XXX) GDNF (N=XXX) and Placebo (N=XXX). Sort by stage, treatment group, and subject ID. Include all ITT Overall subjects. If primary reason for discontinuation is Other, then concatenate specify text.

Listing 17.2.1.2.2 Study Completion Status - Subjects Enrolled but Not Randomized

Programming Note: Repeat table for different population. Omit treatment group header and study medication column.

Listing 17.2.1.2.3 Study Completion Status - Subjects with Initial Informed Consent but Not Enrolled

Programming Note: Repeat table for different population. Omit treatment group header and study medication column.

Listing 17.2.1.3.1 Major Protocol Deviations - ITT Overall Population

ITT Pilot Stage (N=XXX)

GDNF (N=XXX)

Subject ID Age/Race/Ethnicity/ Sex	Date of Deviation	Category	Deviation (brief description)
xxxxxxxxxx/xx/x/x/x	DDMMMYYYY	Xxxxxxxxxxxxxxxxxxxxx	Xxxxxxxxxxxxxxxxxxxxxxxxxxxxxxxxxxxxxxxxxxxxxxxxxxxxxxxxxxxxxxxxxxx

Note: W = white; B = black; P = native Hawaiian or other Pacific islander; A = Asian; I = American Indian or Alaska native; H = Hispanic or Latino; N = not Hispanic or Latino; M = male; F = female.
Source: Dataset: [NAME], Program: xxxxxx.sas, Output: xxxx.rtf, Generated on: DDMONYYYY HH:MM
Page x of y

Programming Note: Repeat listing for ITT Pilot Stage (N=XXX) Placebo (N=XXX), then ITT Primary Stage (N=XXX) GDNF (N=XXX) and Placebo (N=XXX). Sort by stage, treatment group, and subject ID. Include all ITT Overall subjects with major protocol deviations.

Listing 17.2.1.3.2 Minor Protocol Deviations - ITT Overall Population

Programming Note: Repeat listing above for minor protocol deviations.

Listing 17.2.1.4 Subject Populations - ITT Overall Population

ITT Pilot Stage (N=XXX)					
GDNF (N=XXX)					
**Subject ID Age/Race/Ethnicity/Sex**	**Treatment Received?**	**Safety Population**	**ITT Population**	**Per-Protocol Population**	**Reason for Exclusion from Per-Protocol Population**
xxxxxxxxxx/xx/x/x/x	Yes	Yes	Yes		

Note: W = white; B = black; P = native Hawaiian or other Pacific islander; A = Asian; I = American Indian or Alaska native; H = Hispanic or Latino; N = not Hispanic or Latino; M = male; F = female.
<Subjects who are not treated are excluded from the safety population; subjects with no postbaseline assessments are excluded from the ITT population.>
Source: Dataset: [NAME], Program: xxxxxx.sas, Output: xxxx.rtf, Generated on: DDMONYYYY HH:MM
Page x of y

Programming Note: Repeat listing for ITT Pilot Stage (N=XXX) Placebo (N=XXX), then ITT Primary Stage (N=XXX) GDNF (N=XXX) and Placebo (N=XXX). Sort by stage, treatment group, and subject ID. Include all ITT Overall subjects. List all reasons for exclusion from Per-Protocol Population. Per-Protocol Population is not applicable for Pilot Stage (print "No" with reason of deviation from treatment schedule, could be additional reasons).

Listing 17.2.1.5.1 Demographic Characteristics - ITT Overall Population

ITT Pilot Stage (N=XXX)							
GDNF (N=XXX)							
**Subject ID Age/Race/Ethnicity/ Sex**	**Date of Birth**	**Date of Informed Consent**	**Race (specify if Other)**	**Baseline Weight (kg) Height (m)**	**Baseline BMI (kg/m^2)**	**Anti-GDNF Antibodies at Screening**	**NART Error Score (points)**
xxxxxxxxxx/xx/x/x/x	DDMMMYYYY	DDMMMYYYY	Xxxxxxxxxxx	xxx.x<br>xxx.x	xx.x	Negative	xx

Note: W = white; B = black; P = native Hawaiian or other Pacific islander; A = Asian; I = American Indian or Alaska native; H = Hispanic or Latino; N = not Hispanic or Latino; M = male; F = female.
Source: Dataset: [NAME], Program: xxxxxx.sas, Output: xxxx.rtf, Generated on: DDMONYYYY HH:MM
Page x of y

Programming Note: Repeat listing for ITT Pilot Stage (N=XXX) Placebo (N=XXX), then ITT Primary Stage (N=XXX) GDNF (N=XXX) and Placebo (N=XXX). Sort by stage, treatment group, and subject ID. Include all ITT Overall subjects.

Listing 17.2.1.5.2 Parkinson's Disease History at Screening - ITT Overall Population

ITT Pilot Stage (N=XXX)							
GDNF (N=XXX)							
**Subject ID Age/Race/Ethnicity/ Sex**	**Date of First PD Symptom**	**Date of PD Diagnosis**	**Hoehn and Yahr Stage in OFF State**	**UPDRS Motor Score (Part III) OFF State**	**UPDRS Motor Score (Part III) ON State**	**OFF Time per Day (hours)**	**Response to Levodopa[a] (%)**
xxxxxxxxxx/xx/x/x/x	DDMMMYYYY	DDMMMYYYY	x	xx	xx	xx	xx.x

[a] Percentage change in screening UPDRS motor score (part III) following a levodopa challenge.

Note: W = white; B = black; P = native Hawaiian or other Pacific islander; A = Asian; I = American Indian or Alaska native; H = Hispanic or Latino; N = not Hispanic or Latino; M = male; F = female.

Source: Dataset: [NAME], Program: xxxxxx.sas, Output: xxxx.rtf, Generated on: DDMONYYYY HH:MM

Page x of y

Programming Note: Repeat listing for ITT Pilot Stage (N=XXX) Placebo (N=XXX), then ITT Primary Stage (N=XXX) GDNF (N=XXX) and Placebo (N=XXX). Sort by stage, treatment group, and subject ID. Include all ITT Overall subjects.

Listing 17.2.1.5.3 Impulsiveness Behaviour Scale (UPPS-P) at Screening - ITT Overall Population

ITT Pilot Stage (N=XXX)

GDNF (N=XXX)

Subject ID Age/Race/Ethnicity/ Sex	Date	(Negative) Urgency Subscale	(Lack of) Premeditation Subscale	(Lack of) Perseverance Subscale	Sensation Seeking Subscale	Positive Urgency Subscale
xxxxxxxxxx/xx/x/x/x	DDMMMYYYY	xx	Xx	xx	xx	xx

Note: The UPPS-P is a 59-item scale that measures impulsiveness in 5 subscales. A lower score indicates less impulsiveness. W = white; B = black; P = native Hawaiian or other Pacific islander; A = Asian; I = American Indian or Alaska native; H = Hispanic or Latino; N = not Hispanic or Latino; M = male; F = female.
Source: Dataset: [NAME], Program: xxxxxx.sas, Output: xxxx.rtf, Generated on: DDMONYYYY HH:MM
Page x of y

Programming Note: Repeat listing for ITT Pilot Stage (N=XXX) Placebo (N=XXX), then ITT Primary Stage (N=XXX) GDNF (N=XXX) and Placebo (N=XXX). Sort by stage, treatment group, and subject ID. Include all ITT Overall subjects. Do not list imputed values. Calculate the score for each subscale from the responses to the individual items in the subscale.

Listing 17.2.1.6 General Medical History - ITT Overall Population

ITT Pilot Stage (N=XXX)			
GDNF (N=XXX)			
**Subject ID<br>Age/Race/Ethnicity/<br>Sex**	**System Organ Class<br>Preferred Term<br>VERBATIM TERM**	**Start Date**	**Stop Date or Current/Active**
xxxxxxxxxx/xx/x/x/x	xxxxxxxxx xxxxxxxxxx<br>xxxxxxxxxxxxxxxxxxx<br>xxxxxxxxxxxxxx	DDMONYYYY	DDMONYYYY

Note: Medical history data are coded using MedDRA version 19.0. W = white; B = black; P = native Hawaiian or other Pacific islander; A = Asian; I = American Indian or Alaska native; H = Hispanic or Latino; N = not Hispanic or Latino; M = male; F = female.
Source: Dataset: [NAME], Program: xxxxxx.sas, Output: xxxx.rtf, Generated on: DDMONYYYY HH:MM
Page x of y

Programming Note: Repeat listing for ITT Pilot Stage (N=XXX) Placebo (N=XXX), then ITT Primary Stage (N=XXX) GDNF (N=XXX) and Placebo (N=XXX). Sort by stage, treatment group, subject ID, and start date. Include all ITT Overall subjects with data.

Listing 17.2.1.7 Prior and Concomitant Medications – ITT Overall Population

ITT Pilot Stage (N=XXX)

GDNF (N=XXX)							
**Subject ID Age/Race/Ethnicity/ Sex**	**Concomitant Flag[a]**	**ATC Class Coded Medication Name Verbatim Medication Name**	**Start Date (Study Day)/ Stop Date (Study Day) or Ongoing**	**Dose per Frequency**	**Unit**	**Fre-quency**	**Indication**
xxxxxxxxxx/xx/x/x/x	x	xxxxxxxxxxxxxxxxxxx<br>xxxxxxxxxxxxxxxxxxx<br>xxxxxxxxxxxxxx	DDMONYYYY (xxx)/<br>DDMONYYYY (xxx)	xxx	xxx	xxx	xxxxxxxxxxxx

Note: Medications are coded using WHODRUG DDE version MONYYYY. W = white; B = black; P = native Hawaiian or other Pacific islander; A = Asian; I = American Indian or Alaska native; H = Hispanic or Latino; N = not Hispanic or Latino; M = male; F = female.
a P=Prior, C=Concomitant.
Source: Dataset: [NAME], Program: xxxxxx.sas, Output: xxxx.rtf, Generated on: DDMONYYYY HH:MM
Page x of y

Programming Note: Repeat listing for ITT Pilot Stage (N=XXX) Placebo (N=XXX), then ITT Primary Stage (N=XXX) GDNF (N=XXX) and Placebo (N=XXX). Sort by stage, treatment group, subject ID and start date. Include all ITT Overall subjects with data.

Listing 17.2.1.8 Catheter Trajectory - ITT Overall Population

ITT Pilot Stage (N=XXX)			
GDNF (N=XXX)			
**Subject ID Age/Race/Ethnicity/ Sex**	**Surgery**	**Date of Surgery (Study Day)**	**Catheter Placement**
xxxxxxxxxx/xx/x/x/x	Initial/ Respositioning Surgery x	DDMONYYYY (xxx)	Vertical/ Horizontal: Anterior-Posterior/ Horizontal: Posterior-Anterior

Note: W = white; B = black; P = native Hawaiian or other Pacific islander; A = Asian; I = American Indian or Alaska native; H = Hispanic or Latino; N = not Hispanic or Latino; M = male; F = female.
Source: Dataset: [NAME], Program: xxxxxx.sas, Output: xxxx.rtf, Generated on: DDMONYYYY HH:MM
Page x of y

Programming Note: Repeat listing for ITT Pilot Stage (N=XXX) Placebo (N=XXX), then ITT Primary Stage (N=XXX) GDNF (N=XXX) and Placebo (N=XXX). Sort by stage, treatment group, subject ID and date of surgery. Include all ITT Overall subjects with data.

Listing 17.2.1.9 Catheter Positioning Accuracy by Surgery - ITT Overall Population

ITT Pilot Stage (N=XXX)

GDNF (N=XXX)

Subject Age/Race/ Ethnicity/ Sex	Surgery	Date and Time	Distance Between Planned Target and Actual Target (mm) Catheter 1	Catheter 2	Catheter 3	Catheter 4	Mean Across All Catheters	Catheter Positioning Satisfactory	Hemorrhage
xxxxxxxxxx/xx /x/x/x	Initial Surgery	DDMONYYYY HHMM	xx.x	xx.x	xx.x	xx.x	xx.x	Satisfactory<br>Required Repositioning<br>Other: xxxxxxx	No Hemorrhage Detected<br>Minor Hemorrhage without Clinical Signs<br>Minor Hemorrhage with Clinical Signs<br>Major Hemorrhage
	Repositioning Surgery #1	DDMONYYYY HHMM	xx.x	xx.x	xx.x	xx.x	xx.x		

Note: W = white; B = black heritage; P = native Hawaiian or other Pacific islander; A = Asian; I = American Indian or Alaska native; H = Hispanic or Latino; N = not Hispanic or Latino; M = male; F = female.
Source: Dataset: [NAME], Program: xxxxxx.sas, Output: xxxx.rtf, Generated on: DDMONYYYY HH:MM
Page x of y

Programming Note: Repeat listing for ITT Pilot Stage (N=XXX) Placebo (N=XXX), then ITT Primary Stage (N=XXX) GDNF (N=XXX) and Placebo (N=XXX). Sort by stage, treatment group, and subject ID then surgery. Include all ITT Overall subjects with data. Surgeries are Initial Surgery, Repositioning surgery #1, and so on. Abbreviate as necessary.

Listing 17.2.1.10 Test Infusion Data by Visit - ITT Overall Population

ITT Pilot Stage (N=XXX)

GDNF (N=XXX)							
**Subject ID Age/Race/Ethnicity/ Sex**	**Visit**	**Date**	**Time First Start Time Last Stop**	**Catheter 1**	**Catheter 2**	**Catheter 3**	**Catheter 4**
xxxxxxxxxx/xx/x/x/x	Healing Phase	DDMONYYYY	HH:MM HH:MM	Standard	Standard	Standard	Not Used
	Week 40	DDMONYYYY	HH:MM HH:MM	Non-standard	Standard	Standard	Not Used

Note: W = white; B = black; P = native Hawaiian or other Pacific islander; A = Asian; I = American Indian or Alaska native; H = Hispanic or Latino; N = not Hispanic or Latino; M = male; F = female.
Note: Standard regime is a linear ramp up infusion rate of 3-5 µL/min with 400 µL total volume.
Source: Dataset: [NAME], Program: xxxxxx.sas, Output: xxxx.rtf, Generated on: DDMONYYYY HH:MM
Page x of y

Programming Note: Repeat listing for ITT Pilot Stage (N=XXX) Placebo (N=XXX), then ITT Primary Stage (N=XXX) GDNF (N=XXX) and Placebo (N=XXX). Sort by stage, treatment group, subject ID, visit, and date of infusion. Include all ITT Overall subjects with data. Include all test infusions at healing phase, interim, and Week 40 visits, including repeat test infusions. For all listings by visit, use actual visit labels and not “screening” or “baseline.”

Listing 17.2.1.11 Test Infusion Catheter Interruptions/Early Terminations by Visit - ITT Overall Population

ITT Pilot Stage (N=XXX)

GDNF (N=XXX)

Subject ID Age/Race/Ethnicity/ Sex	Visit	Date	Time First Start Time Last Stop	Catheter No. Interrupted/ Terminated Early	Time Infusion Stopped	Time Infusion Restarted	Reason for Stop
xxxxxxxxxx/xx/x/x/x	Healing Phase	DDMONYYYY	HH:MM HH:MM	1	HH:MM	HH:MM	xxxxxx
				2	HH:MM	HH:MM	xxxxxx
				3	HH:MM	HH:MM	xxxxxx
				4	HH:MM	HH:MM	xxxxxx
	Week 40	DDMONYYYY	HH:MM HH:MM	1	HH:MM	HH:MM	xxxxxx
				2	HH:MM	HH:MM	xxxxxx
				3	HH:MM	HH:MM	xxxxxx
				4	HH:MM	HH:MM	xxxxxx

Note: W = white; B = black; P = native Hawaiian or other Pacific islander; A = Asian; I = American Indian or Alaska native; H = Hispanic or Latino; N = not Hispanic or Latino; M = male; F = female.
Source: Dataset: [NAME], Program: xxxxxx.sas, Output: xxxx.rtf, Generated on: DDMONYYYY HH:MM
Page x of y

Programming Note: Repeat listing for ITT Pilot Stage (N=XXX) Placebo (N=XXX), then ITT Primary Stage (N=XXX) GDNF (N=XXX) and Placebo (N=XXX). Sort by stage, treatment group, subject ID, visit, and date of infusion. Include all ITT Overall subjects with data. Include all test infusions at healing phase, interim, and Week 40 visits, including repeat test infusions. For all listings by visit, use actual visit labels and not "screening" or "baseline."

17.2.2 Efficacy Listings

Listing 17.2.2.1 OFF and ON state UPDRS Scores by Visit - ITT Overall Population

ITT Pilot Stage (N=XXX)

GDNF (N=XXX)

Subject ID Age/Race/ Ethnicity/ Sex	Parameter (points)	Visit	Date and Time	Result	Change From Baseline
xxxxxxxxxx/xx /x/x/x	OFF state UPDRS motor score (part III)	Screening Visit 2	DDMONYYYY HHMM	xxx	
		Week 0	DDMONYYYY HHMM	xxx	
		Week 8	DDMONYYYY HHMM	xxx	xxx.x
		…	…	…	…
	ON state UPDRS motor score (part III)	Screening Visit 2	DDMONYYYY HHMM	xxx	
		Week 0	DDMONYYYY HHMM	xxx	
		Week 8	DDMONYYYY HHMM	xxx	xxx.x
		…	…	…	…
	OFF state UPDRS ADL score (part II)	Screening Visit 2	DDMONYYYY HHMM	xxx	
		Week 0	DDMONYYYY HHMM	xxx	
		Week 8	DDMONYYYY HHMM	xxx	xxx.x
		…	…	…	…
	ON state UPDRS ADL score (part II)	Screening Visit 2	DDMONYYYY HHMM	xxx	
		Week 0	DDMONYYYY HHMM	xxx	
		Week 8	DDMONYYYY HHMM	xxx	xxx.x
	etc.	…	…	…	…

Note: Lower scores represent better functioning. OFF and ON state UPDRS total score are sums of motor score (part III) and ADL score (part II). For subject 45, items 22, 27, 28, 29 and 30 are excluded from the motor score (part III). Subject 45 had observed OFF state UPDRS motor score (part III) values of XX, XX, … at visits X, X, … respectively. Subject 45 had observed ON state UPDRS motor score (part III) values of XX, XX, … at visits X, X, … respectively. The observed OFF state and ON state UPDRS motor scores at Screening of subject 45 are used in the calculation of summary statistics in Tables 16.1.4.1 and 16.1.4.2. (Parkinson's Disease History at Screening). W = white; B = black; P = native Hawaiian or other Pacific islander; A = Asian; I = American Indian or Alaska native; H = Hispanic or Latino; N = not Hispanic or Latino; M = male; F = female.

Source: Dataset: [NAME], Program: xxxxxx.sas, Output: xxxx.rtf, Generated on: DDMONYYYY HH:MM

Page x of y

Programming Note: Repeat listing for ITT Pilot Stage (N=XXX) Placebo (N=XXX), then ITT Primary Stage (N=XXX) GDNF (N=XXX) and Placebo (N=XXX). Sort by stage, treatment group, and subject ID then parameter, OFF/ON state, and visit. Include all ITT Overall subjects with data. Parameters are OFF and ON state UPDRS Motor score (part III), OFF and ON state UPDRS Activities of daily living score (part II), OFF and ON state UPDRS total score (sum of motor + ADL

scores), Mentation, behavior, and mood score (part I), and Complications of therapy score (part IV). Visits are Screening Visit 2, Weeks 0, 8, 16, 24, 32, and 40. Baseline is Week 0 for all parameters. Abbreviate as necessary. Do not include imputed values. For all listings by visit, use actual visit labels and not "screening" or "baseline."

Listing 17.2.2.2 PD Motor Fluctuation Diary Ratings by Visit - ITT Overall Population

ITT Pilot Stage (N=XXX)

GDNF (N=XXX)

Subject ID Age/Race/ Ethnicity/ Sex	Parameter (hours)	Visit	Date	Result	Change From Baseline
xxxxxxxxxx/xx /x/x/x	OFF time per day	Screening Visit 2	DDMONYYYY	xx	
		Week 0	DDMONYYYY	xx	
		Week 8	DDMONYYYY	xx	xx.x
		…	…	…	…
	Total good-quality ON time per day	Screening Visit 2	DDMONYYYY	xx	
		Week 0	DDMONYYYY	xx	
		Week 8	DDMONYYYY	xx	xx.x
		…	…	…	…
	ON time per day without dyskinesias	Screening Visit 2	DDMONYYYY	xx	
		Week 0	DDMONYYYY	xx	
		Week 8	DDMONYYYY	xx	xx.x
		…	…	…	…
	ON time per day with non-troublesome dyskinesias	Screening Visit 2	DDMONYYYY	xx	
		Week 0	DDMONYYYY	xx	
		Week 8	DDMONYYYY	xx	xx.x
		…	…	…	…
	ON time per day with troublesome dyskinesias	Screening Visit 2	DDMONYYYY	xx	
		Week 0	DDMONYYYY	xx	
		Week 8	DDMONYYYY	xx	xx.x
	etc.	…	…	…	…

Note: W = white; B = black; P = native Hawaiian or other Pacific islander; A = Asian; I = American Indian or Alaska native; H = Hispanic or Latino; N = not Hispanic or Latino; M = male; F = female.
Note: Total good-quality ON time per day is defined as the sum of ON time per day without dyskinesias + ON time per day with non-troublesome dyskinesias.Source: Dataset: [NAME], Program: xxxxxx.sas, Output: xxxx.rtf, Generated on: DDMONYYYY HH:MM
Page x of y

Programming Note: Repeat listing for ITT Pilot Stage (N=XXX) Placebo (N=XXX), then ITT Primary Stage (N=XXX) GDNF (N=XXX) and Placebo (N=XXX). Sort by stage, treatment group, and subject ID then parameter, and visit. Include all ITT Overall subjects with data. PD diary parameters are OFF time per day, total good-quality ON time per day (sum of ON time per day without dyskinesias + ON time per day with non-troublesome dyskinesias), ON time per day without dyskinesias, ON time per day with non-troublesome dyskinesias, and ON time per day with troublesome dyskinesias. Visits are Screening Visit 2, Weeks 0, 8, 16, 24, 32, and 40. Baseline is Week 0. Abbreviate as necessary. For all listings by visit, use actual visit labels and not "screening" or "baseline."

Listing 17.2.2.3.1 OFF and ON State Timed Walking Test by Visit - ITT Overall Population

ITT Pilot Stage (N=XXX)

GDNF (N=XXX)

Subject Age/Race/ Ethnicity/ Sex	Parameter (seconds)	Visit	Date and Time	Result (mean of replicates)	Change From Baseline
xxxxxxxxxx/xx /x/x/x	OFF state timed walking test	Screening Visit 2	DDMONYYYY HHMM	xx	
		Week 0	DDMONYYYY HHMM	xx	
		Week 8	DDMONYYYY HHMM	xx	xx.x
		…	…	…	…
	ON state timed walking test	Screening Visit 2	DDMONYYYY HHMM	xx	
		Week 0	DDMONYYYY HHMM	xx	
		Week 8	DDMONYYYY HHMM	xx	xx.x
		…	…	…	…

Note: Shorter times represent better function. Two trials per state per visit are averaged for analysis. If only the first trial is completed for a particular state, then the second trial result will be imputed with the worst non-missing second trial result from all subjects for that particular state during the study. If both trials are missing, then the endpoint is not reported for that visit. W = white; B = black; P = native Hawaiian or other Pacific islander; A = Asian; I = American Indian or Alaska native; H = Hispanic or Latino; N = not Hispanic or Latino; M = male; F = female.

Source: Dataset: [NAME], Program: xxxxxx.sas, Output: xxxx.rtf, Generated on: DDMONYYYY HH:MM

Page x of y

Programming Note: Same format as Listing 17.2.2.1. Repeat listing for ITT Pilot Stage (N=XXX) Placebo (N=XXX), then ITT Primary Stage (N=XXX) GDNF (N=XXX) and Placebo (N=XXX). Sort by stage, treatment group, and subject ID then parameter, OFF/ON state, and visit. Include all ITT Overall subjects with data. Parameters are OFF state timed walking test and ON state timed walking test. Visits are Screening Visit 2, Weeks 0, 8, 16, 24, 32, and 40. Baseline is Week 0. Abbreviate as necessary. For all listings by visit, use actual visit labels and not "screening" or "baseline."

Listing 17.2.2.3.2 OFF and ON State Timed Tapping Test by Visit - ITT Overall Population

ITT Pilot Stage (N=XXX)

GDNF (N=XXX)

**Subject Age/Race/ Ethnicity/ Sex**	**Parameter (taps)**	**Visit**	**Date and Time**	**Result (mean of replicates)**	**Change From Baseline**
xxxxxxxxxx/xx /x/x/x	OFF state timed tapping test (left hand)	Screening Visit 2	DDMONYYYY HHMM	xx	
		Week 0	DDMONYYYY HHMM	xx	
		Week 8	DDMONYYYY HHMM	xx	xx.x
		…	…	…	…
	ON state timed tapping test (left hand)	Screening Visit 2	DDMONYYYY HHMM	xx	
		Week 0	DDMONYYYY HHMM	xx	
		Week 8	DDMONYYYY HHMM	xx	xx.x
		…	…	…	…
	OFF state timed tapping test (right hand)	Screening Visit 2	DDMONYYYY HHMM	xx	
		Week 0	DDMONYYYY HHMM	xx	
		Week 8	DDMONYYYY HHMM	xx	xx.x
		…	…	…	…

Note: More taps represent better function. Two trials per state per hand for each visit are averaged for analysis. If only the first trial is completed for a particular state, then the second trial result will be imputed with the worst non-missing second trial result from all subjects for that particular state during the study. If both trials are missing, then the endpoint is not reported for that visit. W = white; B = black; P = native Hawaiian or other Pacific islander; A = Asian; I = American Indian or Alaska native; H = Hispanic or Latino; N = not Hispanic or Latino; M = male; F = female.

Source: Dataset: [NAME], Program: xxxxxx.sas, Output: xxxx.rtf, Generated on: DDMONYYYY HH:MM

Page x of y

Programming Note: Same format as Listing 17.2.2.1. Repeat listing for ITT Pilot Stage (N=XXX) Placebo (N=XXX), then ITT Primary Stage (N=XXX) GDNF (N=XXX) and Placebo (N=XXX). Sort by stage, treatment group, and subject ID then parameter, OFF/ON state, and visit. Include all ITT Overall subjects with data. Parameters are OFF state timed tapping test (left hand), OFF state timed tapping test (right hand),ON state timed tapping test (left hand) and ON state timed tapping test (right hand). Visits are Screening Visit 2, Weeks 0, 8, 16, 24, 32, and 40. Baseline is Week 0. Abbreviate as necessary. For all listings by visit, use actual visit labels and not "screening" or "baseline."

Listing 17.2.2.4 NMSS Scores by Visit - ITT Overall Population

Programming Note: Repeat listing as above (17.2.2.x). Repeat listing for ITT Pilot Stage (N=XXX) Placebo (N=XXX), then ITT Primary Stage (N=XXX) GDNF (N=XXX) and Placebo (N=XXX). Sort by stage, treatment group, and subject ID then parameter, and visit. Include all ITT Overall subjects with data. NMSS parameters are Cardiovascular including falls domain, Sleep/fatigue domain, Mood/cognition domain, Perceptual problems/hallucinations domain; Attention/memory domain, Gastrointestinal tract domain, Urinary domain, Sexual function domain, Miscellaneous domain, and NMSS total score. Visits are Screening Visit 2, Weeks 12, 24, and 40. Abbreviate as necessary. Add footnote "Note: The NMSS is a 30-item interview-based scale that rates non-motor symptoms that occurred in the preceding month in 9 domains. Each item is rated from 0 (none) to 3 (severe) for severity and from 1 (rarely) to 4 (very frequent) for frequency. The maximum score for an individual item is 12. The higher the score, the worse the subject's condition. The maximum NMSS total score is 360." Do not list imputed values. For all listings by visit, use actual visit labels and not "screening" or "baseline."

Listing 17.2.2.5 PDQ-39 Scores at Screening and Week 40 - ITT Overall Population

Programming Note: Repeat listing as above (17.2.2.x). *Repeat listing for ITT Pilot Stage (N=XXX) Placebo (N=XXX), then ITT Primary Stage (N=XXX) GDNF (N=XXX) and Placebo (N=XXX). Sort by stage, treatment group, and subject ID then parameter, and visit. Include all ITT Overall subjects with data. PDQ-39 parameters are Mobility dimension, ADL dimension, Emotional well-being dimension, Stigma dimension, Social support dimension, Cognitions dimension, Communication dimension, Bodily discomfort dimension, and the Single index (total) PDQ-39 score. Visits are Screening Visit 2 and Week 40. Abbreviate as necessary. Add note: The PDQ-39 is a self-administered 39-item PD-specific scale that rates symptoms that occurred in the preceding month in 8 dimensions. Each item is rated from 0 (never) to 4 (always) for frequency. The score for each dimension is the average of responses in the dimension, weighted by the number of questions and multiplied by 100. PDQ-39 dimension and total scores range from 0 to 100 (0 = no problem, 100 = problem as worse as possible). The higher the score, the worse the subject's condition. For all listings by visit, use actual visit labels and not "screening" or "baseline."*

Listing 17.2.2.6 EQ-5D Scores at Screening and Week 40 - ITT Overall Population

Programming Note: Repeat listing as above (17.2.2.x). *Repeat listing for ITT Pilot Stage (N=XXX) Placebo (N=XXX), then ITT Primary Stage (N=XXX) GDNF (N=XXX) and Placebo (N=XXX). Sort by stage, treatment group, and subject ID then parameter, and visit. Include all ITT Overall subjects with data. Parameters are the individual item scores and the visual analog scale score. Visits are Screening Visit 2 and Week 40. Abbreviate as necessary. Add note: The EQ-5D is a self-administered 5-item scale with three possible answers for each item (1=no problem, 2=moderate problem, 3=severe problem), and a visual analog scale ranging from 0 to 100, where 0 indicates worst health and 100 indicates best health. Change from baseline only applies to the visual analog scale. For all listings by visit, use actual visit labels and not "screening" or "baseline."*

Listing 17.2.2.7 SNAQ Scores at Screening and Week 40 - ITT Overall Population

Programming Note: Repeat listing as above (17.2.2.x). *Repeat listing for ITT Pilot Stage (N=XXX) Placebo (N=XXX), then ITT Primary Stage (N=XXX) GDNF (N=XXX) and Placebo (N=XXX). Sort by stage, treatment group, and subject ID then parameter, and visit. Include all ITT Overall subjects with data. Parameters are total SNAQ score. Visits are Screening Visit 2 and Week 40. Abbreviate as necessary. Add note: The SNAQ is a self-administered 4-question instrument with total scores ranging from 4 to 20 (4=poor appetite, 20=good appetite). For all listings by visit, use actual visit labels and not "screening" or "baseline."*

Listing 17.2.2.8.1 Levodopa and Levodopa Equivalent Medication Actual Total Daily Doses - ITT Overall Population

ITT Pilot Stage (N=XXX)

GDNF (N=XXX)

Subject ID Age/Race/Ethnicity/ Sex	Category	Medication	Actual Total Daily Dose (mg) Screening Visit 2	Baseline (Week 0)	Week 40
xxxxxxxxxx/xx /x/x/x	Levodopa preparations	Immediate release preparations without COMT inhibition	xx	xx	xx
		Immediate release preparations with entacapone			
		Immediate release preparations with tolcapone			
		Controlled release preparations			
		Levodopa/carbidopa (Duodopa)			
	Dopamine agonists	Ropinirole immediate release			
		Ropinirole long acting			
		Pramipexole immediate release (base)			
		Pramipexole immediate release (salt)			
		Pramipexole long acting (base)			
		Pramipexole long acting (salt)			
		Cabergoline			
		Rotigotine			
		Piribedil			
		Apomorphine			
		Bromocriptine			
		Pergolide			
		Lisuride			
		Dihydroergocryptine (DHEC)			
	COMT inhibitors	Entacapone			
		Tolcapone			
	MAO-B inhibitors	Selegiline oral			
		Selegiline sublingual			
		Rasagiline			
	Other	Amantadine			
		Other: xxxxxxxx			

Note: W = white; B = black; P = native Hawaiian or other Pacific islander; A = Asian; I = American Indian or Alaska native; H = Hispanic or Latino; N = not Hispanic or Latino; M = male; F = female.
Source: Dataset: [NAME], Program: xxxxxx.sas, Output: xxxx.rtf, Generated on: DDMONYYYY HH:MM
Page x of y

Programming Note: Repeat listing for ITT Pilot Stage (N=XXX) Placebo (N=XXX), then ITT Primary Stage (N=XXX) GDNF (N=XXX) and Placebo (N=XXX). Sort

by stage, treatment group, subject ID and category. Include all ITT Overall subjects with data. ***Actual dose means as documented on the CRF (ie, unconverted). Include only those categories and medications with data.***

Listing 17.2.2.8.2 Effective Levodopa and Levodopa Equivalent Medication Total Daily Doses - ITT Overall Population

ITT Pilot Stage (N=XXX)

GDNF (N=XXX)

**Subject ID Age/Race/Ethnicity/ Sex**	**Medication**	**Total Daily Dose (mg)** Screening Visit 2	Baseline (Week 0)	Week 40
xxxxxxxxxx/xx /x/x/x	Effective Levodopa	xx	xx	xx
	Levodopa Equivalent	xx	xx	xx
xxxxxxxxxx/xx /x/x/x	Effective Levodopa	xx	xx	xx
	Levodopa Equivalent	xx	xx	xx

Note: W = white; B = black; P = native Hawaiian or other Pacific islander; A = Asian; I = American Indian or Alaska native; H = Hispanic or Latino; N = not Hispanic or Latino; M = male; F = female.

Note: Specific conversion factors are used in order to characterize the subject's effective levodopa dose. Immediate release preparations taken without concomitant catechol-O-methyl transferase (COMT) inhibitors do not require conversion. The daily doses of immediate release preparations taken with COMT inhibitors and of controlled release preparations are multiplied by the corresponding conversion factors. The total daily levodopa dose is then calculated by adding together the converted daily doses of all individual levodopa-containing preparations. Levodopa equivalent dose is calculated by multiplying each PD medication dose by a specific conversion factor indicating the drug's relative potency with respect to immediate release levodopa unaccompanied by COMT inhibitors. The total daily levodopa equivalent dose is calculated by adding together the daily levodopa equivalent doses of all individual PD medications. COMT inhibitor doses are not included in either of these calculations.

Source: Dataset: [NAME], Program: xxxxxx.sas, Output: xxxx.rtf, Generated on: DDMONYYYY HH:MM
Page x of y

Programming Note: Repeat listing for ITT Pilot Stage (N=XXX) Placebo (N=XXX), then ITT Primary Stage (N=XXX) GDNF (N=XXX) and Placebo (N=XXX). Sort by stage, treatment group, subject ID and category. Include all ITT Overall subjects with data. ***Total daily doses are the converted doses.***

17.2.3 Imaging Listings

Listing 17.2.3.1 Volume of Distribution, Volume of Interest Coverage, and Total Putamenal Coverage as Determined by Contrast-Enhanced T1-Weighted MRI by Visit - ITT Overall Population

Programming Note: Repeat listing as above (17.2.2.x). *Sort by stage, treatment group and subject ID then parameter and visit. Include all ITT Overall subjects with data. Parameters are volume of distribution (mL, left and right), VOI (mL, left and right), VOI coverage (mL, left and right; %, left and right), putamenal volume of distribution (mL, left and right), total volume of putamen (mL, left and right), total putamenal coverage (%, left and right). Visits are last test infusion at the end of the healing phase and Week 40. Abbreviate as necessary. For all listings by visit, use actual visit labels and not "screening" or "baseline."*

Listing 17.2.3.2 PET ^{18}F-DOPA Uptake by Visit - ITT Overall Population

Programming Note: Repeat listing as above (17.2.2.x). *Sort by stage, treatment group, subject ID and visit. Include all ITT Overall subjects with data. Parameter is ^{18}F-DOPA uptake rate constant (for anterior, central and posterior putamen, and caudate nucleus, each for left and right hemispheres). Visits are Week 0 and Week 40 (also Week 12 for Pilot Stage). Abbreviate as necessary. For all listings by visit, use actual visit labels and not "screening" or "baseline."*

17.2.4 Safety Listings

Listing 17.2.4.1.1 Exposure to Study Medication - Safety Overall Population

Safety Pilot Stage (N=XXX)		
GDNF (N=XXX)		
**Subject ID Age/Race/Ethnicity/ Sex**	**No. of Infusions of Study Medication**	**Total Exposure (mg)**
xxxxxxxxxx/xx/x/x/x	xx	xxxx.x

Note: W = white; B = black; P = native Hawaiian or other Pacific islander; A = Asian; I = American Indian or Alaska native; H = Hispanic or Latino; N = not Hispanic or Latino; M = male; F = female.
Source: Dataset: [NAME], Program: xxxxxx.sas, Output: xxxx.rtf, Generated on: DDMONYYYY HH:MM
Page x of y

Programming Note: Repeat listing for Safety Pilot Stage (N=XXX) Placebo (N=XXX), then Safety Primary Stage (N=XXX) GDNF (N=XXX) and Placebo (N=XXX). Sort by stage, treatment group, and subject ID. Include all Safety Overall subjects.

Listing 17.2.4.1.2 Study Medication Infusion Data by Visit - Safety Overall Population

Safety Pilot Stage (N=XXX)

GDNF (N=XXX)

Subject ID Age/Race/Ethnicity/ Sex	Visit	Date	Time First Start Time Last Stop	Catheter 1	Catheter 2	Catheter 3	Catheter 4
xxxxxxxxxx/xx/x/x/x	Week x	DDMONYYYY	HH:MM HH:MM	Standard	Standard	Standard	Not Used
	Week x	DDMONYYYY	HH:MM HH:MM	Non-standard	Standard	Standard	Not Used

Note: W = white; B = black; P = native Hawaiian or other Pacific islander; A = Asian; I = American Indian or Alaska native; H = Hispanic or Latino; N = not Hispanic or Latino; M = male; F = female.
Note: Standard regime is a linear ramp up infusion rate of 3-5 µL/min with 400 µL total volume.

Source: Dataset: [NAME], Program: xxxxxx.sas, Output: xxxx.rtf, Generated on: DDMONYYYY HH:MM
Page x of y

Programming Note: Repeat listing for Safety Pilot Stage (N=XXX) Placebo (N=XXX), then Safety Primary Stage (N=XXX) GDNF (N=XXX) and Placebo (N=XXX). Sort by stage, treatment group, and subject ID then visit. Include all Safety Overall subjects. For all listings by visit, use actual visit labels and not "screening" or "baseline."

Listing 17.2.4.1.3 Study Medication Infusion Catheter Interruptions/Early Terminations by Visit - Safety Overall Population

Safety Pilot Stage (N=XXX)

GDNF (N=XXX)

Subject ID Age/Race/Ethnicity/ Sex	Visit	Date	Time First Start Time Last Stop	Catheter No. Interrupted/ Terminated Early	Time Infusion Stopped	Time Infusion Restarted	Reason for Stop
xxxxxxxxxx/xx/x/x/x	Week x	DDMONYYYY	HH:MM	1	HH:MM	HH:MM	
			HH:MM	2	HH:MM	HH:MM	
				3	HH:MM	HH:MM	
				4	HH:MM	HH:MM	xxxxxx
	Week x	DDMONYYYY	HH:MM	1	HH:MM	HH:MM	
			HH:MM	2	HH:MM	HH:MM	
				3	HH:MM	HH:MM	
				4	HH:MM	HH:MM	xxxxxx

Note: W = white; B = black; P = native Hawaiian or other Pacific islander; A = Asian; I = American Indian or Alaska native; H = Hispanic or Latino; N = not Hispanic or Latino; M = male; F = female.
Source: Dataset: [NAME], Program: xxxxxx.sas, Output: xxxx.rtf, Generated on: DDMONYYYY HH:MM
Page x of y

Programming Note: Repeat listing for Safety Pilot Stage (N=XXX) Placebo (N=XXX), then Safety Primary Stage (N=XXX) GDNF (N=XXX) and Placebo (N=XXX). Sort by stage, treatment group, and subject ID then visit. Include all Safety Overall subjects. For all listings by visit, use actual visit labels and not "screening" or "baseline."

Listing 17.2.4.2 Adverse Events - Safety Overall Population

Safety Pilot Stage (N=XXX)								
GDNF (N=XXX)								
**Subject ID Age/Race/Ethnicity Sex**	**MedDRA Preferred Term VERBATIM TERM**	**Start Date and Time (Day)/ Stop Date and Time (Day) or Ongoing**	**SAE**	**Severity**	**Related to Study Drug?**	**Related to Device?**	**Out-come[a]**	**Ac-tion[b]**
xxxxxxxxxx/xx/x/x/x	xxxxxxxxxxx xxxxxxxxxxx	DDMONYYYY HHMM (xxx)/ DDMONYYYY HHMM (xxx)	Yes	Mild	No	Yes	xx	99: xxxxxx

Note: Adverse events are coded using MedDRA version 19.0. W = white; B = black; P = native Hawaiian or other Pacific islander; A = Asian; I = American Indian or Alaska native; H = Hispanic or Latino; N = not Hispanic or Latino; M = male; F = female.

a Outcome: 1 = Recovered/resolved; 2 = Not recovered/not resolved; 3 = Recovered/resolved with sequelae; 4 = Fatal.

b Action: 1 = Current infusion interrupted and restarted; 2 = Current infusion terminated; 3 = Infusion protocol modified; 4 = Infusion schedule suspended and resumed; 5 = Treatment discontinued permanently; 6 = Surgical revision/replacement of extracerebral device parts; 7 = Surgical repositioning/replacement of intracerebral device parts; 99 = Other.

Source: Dataset: [NAME], Program: xxxxxx.sas, Output: xxxx.rtf, Generated on: DDMONYYYY HH:MM

Page x of y

Programming Note: Repeat listing for Safety Pilot Stage (N=XXX) Placebo (N=XXX), then Safety Primary Stage (N=XXX) GDNF (N=XXX) and Placebo (N=XXX). Sort by stage, treatment group, subject ID, start date and time. Include all Safety Overall subjects with data.

Listing 17.2.4.3 Pre-Treatment Adverse Events - Safety Enrolled Population
Programming Note: Repeat AE listing above, using the groups GDNF, Placebo and Not Randomized Sort by stage, group, subject ID, start date and time.

Listing 17.2.4.4 Adverse Events of Special Interest: Dyskinesias - Safety Overall Population
Programming Note: Repeat AE listing above. Repeat listing for Safety Pilot Stage (N=XXX) Placebo (N=XXX), then Safety Primary Stage (N=XXX) GDNF (N=XXX) and Placebo (N=XXX). Sort by stage, treatment group, subject ID, start date and time. Include all Safety Overall subjects with data.

Listing 17.2.4.5 Adverse Events of Special Interest: Falls - Safety Overall Population
Programming Note: Repeat AE listing above. Repeat listing for Safety Pilot Stage (N=XXX) Placebo (N=XXX), then Safety Primary Stage (N=XXX) GDNF (N=XXX) and Placebo (N=XXX). Sort by stage, treatment group, subject ID, start date and time. Include all Safety Overall subjects with data.

Listing 17.2.4.6 Adverse Events of Special Interest: Adverse Changes in Mood - Safety Overall Population
Programming Note: Repeat AE listing above. Repeat listing for Safety Pilot Stage (N=XXX) Placebo (N=XXX), then Safety Primary Stage (N=XXX) GDNF (N=XXX) and Placebo (N=XXX). Sort by stage, treatment group, subject ID, start date and time. Include all Safety Overall subjects with data.

Listing 17.2.4.7 Adverse Events of Special Interest: Impulsivity - Safety Overall Population
Programming Note: Repeat AE listing above. Repeat listing for Safety Pilot Stage (N=XXX) Placebo (N=XXX), then Safety Primary Stage (N=XXX) GDNF (N=XXX) and Placebo (N=XXX). Sort by stage, treatment group, subject ID, start date and time. Include all Safety Overall subjects with data.

Listing 17.2.4.8 Port Symptoms by Visit - Safety Overall Population

Safety Pilot Stage (N=XXX)			
GDNF (N=XXX)			
**Subject ID Age/Race/Ethnicity Sex**	**Visit**	**Date Performed**	**Result**
xxxxxxxxxx/xx/x/x/x	Healing Phase	DDMONYYYY	No skin reaction
	…	….	Redness with slight swelling
			Redness, moistness and moderate swelling with tissue granulation
			Overt infection

Note: W = white; B = black; P = native Hawaiian or other Pacific islander; A = Asian; I = American Indian or Alaska native; H = Hispanic or Latino; N = not Hispanic or Latino; M = male; F = female.
Source: Dataset: [NAME], Program: xxxxxx.sas, Output: xxxx.rtf, Generated on: DDMONYYYY HH:MM
Page x of y

Programming Note: Repeat listing for Safety Pilot Stage (N=XXX) Placebo (N=XXX), then Safety Primary Stage (N=XXX) GDNF (N=XXX) and Placebo (N=XXX). Sort by stage, treatment group, and subject ID then visit. Include all Safety Overall subjects with data.

Listing 17.2.4.9 Adverse Changes in MRI Findings - Safety Overall Population

Programming Note: Repeat AE listing 17.2.4.2. Sort by stage, treatment group, subject ID then by start date and time.

Listing 17.2.4.10 Hematology Results by Visit - Safety Overall Population

Safety Pilot Stage (N=XXX)								
GDNF (N=XXX)								
**Subject ID Age/Race/Ethnicity/ Sex**	**Parameter (Unit)**	**Visit**	**Sample Date**	**Result**	**Change from Baseline**	**Normal Range**	**Flag**	**Clin Sig?**
xxxxxxxxxx/xx/x/x/x	xxxxxxxxxxxxx	Screening Visit 1	DDMONYYYY	xxx.xx		xxx.xx – xxx.xx	H	No
		Week 4	DDMONYYYY	xxx.xx	xx.xx	xxx.xx – xxx.xx	H	No
		…	…	…	…	…	…	…

Note: W = white; B = black; P = native Hawaiian or other Pacific islander; A = Asian; I = American Indian or Alaska native; H = Hispanic or Latino; N = not Hispanic or Latino; M = male; F = female; H = high; L = low.
Source: Dataset: [NAME], Program: xxxxxx.sas, Output: xxxx.rtf, Generated on: DDMONYYYY HH:MM
Page x of y

Programming Note: Repeat listing for Safety Pilot Stage (N=XXX) Placebo (N=XXX), then Safety Primary Stage (N=XXX) GDNF (N=XXX) and Placebo (N=XXX). Sort by stage, treatment group, and subject ID then parameter (order as shown in SAP Table 4), and visit. Include all Safety Overall subjects with data. For all listings by visit, use actual visit labels and not "screening" or "baseline."

Listing 17.2.4.11 Serum Chemistry Results by Visit - Safety Overall Population

Programming Note: Repeat listing for serum chemistry parameters. Repeat listing for Safety Pilot Stage (N=XXX) Placebo (N=XXX), then Safety Primary Stage (N=XXX) GDNF (N=XXX) and Placebo (N=XXX). Sort by stage, treatment group, and subject ID then parameter (order as shown in SAP Table 4), and visit. Include all Safety Overall subjects with data. For all listings by visit, use actual visit labels and not "screening" or "baseline."

Listing 17.2.4.12 Urinalysis Results by Visit - Safety Overall Population

Programming Note: Repeat listing for urinalysis parameters. Repeat listing for Safety Pilot Stage (N=XXX) Placebo (N=XXX), then Safety Primary Stage (N=XXX) GDNF (N=XXX) and Placebo (N=XXX). Sort by stage, treatment group, and subject ID then parameter (order as shown in SAP Table 4), and visit. Include all Safety Overall subjects with data. For all listings by visit, use actual visit labels and not "screening" or "baseline."

Listing 17.2.4.13 Anti-GDNF Antibodies - Safety Overall Population

Safety Pilot Stage (N=XXX)				
GDNF (N=XXX)				
Subject ID Age/Race/Ethnicity/ Sex	Parameter	Visit	Sample Date	Result
xxxxxxxxxx/xx/x/x/x	Anti-GDNF Binding Antibody	Screening Visit 1 …	DDMONYYYY	Negative
	Anti-GDNF Neutralizing Antibody	…		Positive

Note: W = white; B = black; P = native Hawaiian or other Pacific islander; A = Asian; I = American Indian or Alaska native; H = Hispanic or Latino; N = not Hispanic or Latino; M = male; F = female.
Source: Dataset: [NAME], Program: xxxxxx.sas, Output: xxxx.rtf, Generated on: DDMONYYYY HH:MM
Page x of y

Programming Note: Repeat listing for Safety Pilot Stage (N=XXX) Placebo (N=XXX), then Safety Primary Stage (N=XXX) GDNF (N=XXX) and Placebo (N=XXX). Sort by stage, treatment group, and subject ID then parameter and visit. Include all Safety Overall subjects with data. Include all visits with data. Abbreviate as necessary. For all listings by visit, use actual visit labels and not “screening” or “baseline.”

Listing 17.2.4.14 Plasma GDNF Concentration Results by Visit - Safety Overall Population

Safety Pilot Stage (N=XXX)				
GDNF (N=XXX)				
**Subject ID Age/Race/Ethnicity/ Sex**	**Visit**	**Sample Date**	**Plasma GDNF Concentration (Unit)**	**Change from Baseline**
xxxxxxxxxx/xx/x/x/x	Screening Visit 1	DDMONYYYY	xxx.xx	
	Week 4	DDMONYYYY	xxx.xx	xx.xx

Note: W = white; B = black; P = native Hawaiian or other Pacific islander; A = Asian; I = American Indian or Alaska native; H = Hispanic or Latino; N = not Hispanic or Latino; M = male; F = female.
Source: Dataset: [NAME], Program: xxxxxx.sas, Output: xxxx.rtf, Generated on: DDMONYYYY HH:MM
Page x of y

Programming Note: Repeat listing for Safety Pilot Stage (N=XXX) Placebo (N=XXX), then Safety Primary Stage (N=XXX) GDNF (N=XXX) and Placebo (N=XXX). Sort by stage, treatment group, and subject ID then parameter, and visit. Include all Safety Overall subjects with data. For all listings by visit, use actual visit labels and not "screening" or "baseline."

Listing 17.2.4.15 Physical Examination at Screening and Week 40 - Safety Overall Population

Safety Pilot Stage (N=XXX)

GDNF (N=XXX)					
**Subject ID Age/Race/Ethnicity/ Sex**	**Body System**	**Visit**	**Date**	**Normal/ Abnormal/ Not Done**	**Abnormality**
xxxxxxxxxx/xx/x/x/x	Skin	Screening Visit 1	DDMONYYYY	xxxxxxxx	xxxxxxxxxxxxxxxxxxxx
		Week 40	DDMONYYYY	xxxxxxxx	xxxxxxxxxxxxxxxxxxxx
	Head, Ears, Eyes, Nose, and Throat	Screening Visit 1	DDMONYYYY	xxxxxxxx	xxxxxxxxxxxxxxxxxxxx
		Week 40	DDMONYYYY	xxxxxxxx	xxxxxxxxxxxxxxxxxxxx
	Respiratory	Screening Visit 1	DDMONYYYY	xxxxxxxx	xxxxxxxxxxxxxxxxxxxx
		Week 40	DDMONYYYY	xxxxxxxx	xxxxxxxxxxxxxxxxxxxx
	Cardiovascular	Screening Visit 1	DDMONYYYY	xxxxxxxx	xxxxxxxxxxxxxxxxxxxx
		Week 40	DDMONYYYY	xxxxxxxx	xxxxxxxxxxxxxxxxxxxx
	Abdomen (incl. liver and kidneys)	Screening Visit 1	DDMONYYYY	xxxxxxxx	xxxxxxxxxxxxxxxxxxxx
		Week 40	DDMONYYYY	xxxxxxxx	xxxxxxxxxxxxxxxxxxxx
	Musculoskeletal	Screening Visit 1	DDMONYYYY	xxxxxxxx	xxxxxxxxxxxxxxxxxxxx
		Week 40	DDMONYYYY	xxxxxxxx	xxxxxxxxxxxxxxxxxxxx
	Neurological	Screening Visit 1	DDMONYYYY	xxxxxxxx	xxxxxxxxxxxxxxxxxxxx
		Week 40	DDMONYYYY	xxxxxxxx	xxxxxxxxxxxxxxxxxxxx
	Gastrointestinal	Screening Visit 1	DDMONYYYY	xxxxxxxx	xxxxxxxxxxxxxxxxxxxx
		Week 40	DDMONYYYY	xxxxxxxx	xxxxxxxxxxxxxxxxxxxx
	Genitourinary	Screening Visit 1	DDMONYYYY	xxxxxxxx	xxxxxxxxxxxxxxxxxxxx
		Week 40	DDMONYYYY	xxxxxxxx	xxxxxxxxxxxxxxxxxxxx
	Endocrine	Screening Visit 1	DDMONYYYY	xxxxxxxx	xxxxxxxxxxxxxxxxxxxx
		Week 40	DDMONYYYY	xxxxxxxx	xxxxxxxxxxxxxxxxxxxx
	Lymph nodes	Screening Visit 1	DDMONYYYY	xxxxxxxx	xxxxxxxxxxxxxxxxxxxx
		Week 40	DDMONYYYY	xxxxxxxx	xxxxxxxxxxxxxxxxxxxx
	Other	Screening Visit 1	DDMONYYYY	xxxxxxxx	xxxxxxxxxxxxxxxxxxxx
		Week 40	DDMONYYYY	xxxxxxxx	xxxxxxxxxxxxxxxxxxxx

Note: W = white; B = black; P = native Hawaiian or other Pacific islander; A = Asian; I = American Indian or Alaska native; H = Hispanic or Latino; N = not Hispanic or Latino; M = male; F = female.
Source: Dataset: [NAME], Program: xxxxxx.sas, Output: xxxx.rtf, Generated on: DDMONYYYY HH:MM
Page x of y

Programming Note: Repeat listing for Safety Pilot Stage (N=XXX) Placebo (N=XXX), then Safety Primary Stage (N=XXX) GDNF (N=XXX) and Placebo (N=XXX). Sort by stage, treatment group, and subject ID then body system and visit. Include all Safety Overall subjects with data. Visits are Screening Visit 1 and Week 40. Abbreviate as necessary. For all listings by visit, use actual visit labels and not "screening" or "baseline."

Listing 17.2.4.16 Vital Signs from Infusions with a Clinically Revelant Postbaseline Abnormal Result by Visit and Time Point - Safety Overall Population

Safety Pilot Stage (N=XXX)

GDNF (N=XXX)

Subject ID Age/Race/Ethnicity/ Sex	Parameter (Unit)	Visit	Date	Time Point	Result	Change from Baseline[a]	Clinically Relevant? If Y, then criteria
xxxxxxxxxx/xx/x/x/x	xxxxxxxxxxxxx	Screening Visit 1	DDMONYYYY		xxx.xx		
		…					
		Week x	DDMONYYYY	Pre-infusion	xxx.xx		
				15 min after infusion start	xxx.xx	xx.xx	N
				…	…	…	Y/ < 50 bpm

[a] For test infusion and study medication infusion visits, change from baseline refers to change from pre-infusion value.

Note: W = white; B = black; P = native Hawaiian or other Pacific islander; A = Asian; I = American Indian or Alaska native; H = Hispanic or Latino; N = not Hispanic or Latino; M = male; F = female.

Note: All values for a given parameter with a clinically relevant abnormal result at a particular visit are listed.

Source: Dataset: [NAME], Program: xxxxxx.sas, Output: xxxx.rtf, Generated on: DDMONYYYY HH:MM

Page x of y

Programming Note: Repeat listing for Safety Pilot Stage (N=XXX) Placebo (N=XXX), then Safety Primary Stage (N=XXX) GDNF (N=XXX) and Placebo (N=XXX). Sort by stage, treatment group, and subject ID then parameter, visit, and time point. Include all Safety Overall subjects with data. Parameters are pulse (sitting and standing; beats/min), respiration (breaths/min), systolic and diastolic blood pressure (BP; sitting and standing; mmHg), and temperature (°C). Data are presented for Screening Visit 1, all test infusion visits (healing phase visits, interim visits after catheter repositioning, and Week 40 visits) and all study medication infusion visits (Weeks 0, 4, 8, 12, 16, 20, 24, 28, 32, and 36), including pre-infusion value, 15 min after infusion start, 30 min after infusion start, 45 min after infusion start, 60 min after infusion start, 75 min after infusion start, 90 min after infusion start, 105 min after infusion start, 120 min after infusion start, and post-dose, where applicable. If clinically relevant, then concatenate the criteria listed in SAP Table 5. For all listings by visit, use actual visit labels and not "screening" or "baseline."

Listing 17.2.4.17 Weight by Visit - Safety Overall Population

Programming Note: Repeat UPDRS Listing 17.2.2.1 omitting the parameter column. Repeat listing for Safety Pilot Stage (N=XXX) Placebo (N=XXX), then Safety Primary Stage (N=XXX) GDNF (N=XXX) and Placebo (N=XXX). Sort by stage, treatment group, and subject ID then parameter and visit. Include all Safety Overall subjects with data. Data are presented for Screening Visit 1 and all 10 infusion visits (Weeks 0, 4, 8, 12, 16, 20, 24, 28, 32, 36 and 40). Abbreviate as necessary. For all listings by visit, use actual visit labels and not "screening" or "baseline."

Listing 17.2.4.18 Electrocardiogram Results at Screening and Week 40 – Continuous Parameters, Safety Overall Population

Programming Note: Repeat VS Listing 17.2.4.16 (without column for time points). Repeat listing for Safety Pilot Stage (N=XXX) Placebo (N=XXX), then Safety Primary Stage (N=XXX) GDNF (N=XXX) and Placebo (N=XXX). Sort by stage, treatment group, and subject ID then parameter and visit. Include all Safety Overall subjects with data. Parameters are heart rate (beats/min), PR interval (ms), QRS interval (ms), QT interval (ms), and QTc interval (ms). Visits are Screening Visit 1 and Week 40. Abbreviate as necessary. If clinically relevant, then concatenate the criteria listed in SAP Table 6. For all listings by visit, use actual visit labels and not "screening" or "baseline." Add the following footnote:
Note: Hodges QT correction formula was used.

Listing 17.2.4.19 Electrocardiogram Results at Screening and Week 40 – Overall Impression, Safety Overall Population

Safety Pilot Stage (N=XXX)

GDNF (N=XXX)

Subject ID Age/Race/Ethnicity/Sex	Visit	Date	Overall Impression (Normal or Abnormal?)	Clinically Significant?	Comment
xxxxxxxxxx/xx/x/x/x	Screening Visit 1	DDMONYYYY	Abnormal	Yes	xxxxxxxxxxxxxxxxxxxxxxx
	Week 40	DDMONYYYY	Normal		

Note: W = white; B = black; P = native Hawaiian or other Pacific islander; A = Asian; I = American Indian or Alaska native; H = Hispanic or Latino; N = not Hispanic or Latino; M = male; F = female.
Source: Dataset: [NAME], Program: xxxxxx.sas, Output: xxxx.rtf, Generated on: DDMONYYYY HH:MM
Page x of y

Programming Note: Repeat listing for Safety Pilot Stage (N=XXX) Placebo (N=XXX), then Safety Primary Stage (N=XXX) GDNF (N=XXX) and Placebo (N=XXX). Sort by stage, treatment group, and subject ID then visit. Include all Safety Overall subjects with data. Visits are Screening Visit 1 and Week 40. Abbreviate as necessary. For all listings by visit, use actual visit labels and not “screening” or “baseline.”

Listing 17.2.4.20 Glasgow Coma Scale Results < 15 by Visit - Safety Overall Population

Safety Pilot Stage (N=XXX)

GDNF (N=XXX)

Subject ID Age/Race/ Ethnicity/Sex	Parameter	Visit	Date and Time	Time Point	Result
xxxxxxxxxx/xx/x/x/x	Visual Response	Healing Phase	DDMONYYYY HHMM	Pre-infusion	x
		Healing Phase	DDMONYYYY HHMM	30 min after infusion start	x
		Healing Phase	DDMONYYYY HHMM	Post-infusion	x
	Verbal Ability	Healing Phase	DDMONYYYY HHMM	Pre-infusion	x
		Healing Phase	DDMONYYYY HHMM	30 min after infusion start	x
		…	…	…	…

Note: Glasgow Coma Scale items include visual response, verbal ability, and motor skills. Each item has 4-6 possible responses. The best possible total score is 15. W = white; B = black; P = native Hawaiian or other Pacific islander; A = Asian; I = American Indian or Alaska native; H = Hispanic or Latino; N = not Hispanic or Latino; M = male; F = female.
Source: Dataset: [NAME], Program: xxxxxx.sas, Output: xxxx.rtf, Generated on: DDMONYYYY HH:MM
Page x of y

Programming Note: Repeat listing for Safety Pilot Stage (N=XXX) Placebo (N=XXX), then Safety Primary Stage (N=XXX) GDNF (N=XXX) and Placebo (N=XXX). Sort by stage, treatment group, and subject ID, then parameter, visit, date, and time point. Include all Safety Overall subjects with a result <15 for a particular visit. Abbreviate as necessary. Include the scoring information in SAP Table 7 in an endnote.

Listing 17.2.4.21 QUIP Items Answered with Yes by Visit - Safety Overall Population

Safety Pilot Stage (N=XXX)

GDNF (N=XXX)

Subject ID Age/Race/ Ethnicity/Sex	Reported By	Question	Behavior	Visit	Date
xxxxxxxxxx/xx/x/x/x	Subject	Do you or others think you have an issue with too much gambling, sex, buying, or eating behaviors?	Gambling	Screening Visit 1	DDMONYYYY
		…	…	Week 8	DDMONYYYY
			Sex	…	DDMONYYYY
			Buying	…	DDMONYYYY
			Eating	…	DDMONYYYY
			…	…	
	Informant	Do you or others think you have an issue with too much gambling, sex, buying, or eating behaviors?	Gambling	Screening Visit 1	DDMONYYYY
		…	…	…	…

Note: The QUIP is a self-administered or informant-completed scale that includes 13 questions covering symptoms related to the 4 commonest impulse control disorders in PD, other behaviors, and problematic use of medication. W = white; B = black; P = native Hawaiian or other Pacific islander; A = Asian; I = American Indian or Alaska native; H = Hispanic or Latino; N = not Hispanic or Latino; M = male; F = female.
Source: Dataset: [NAME], Program: xxxxxx.sas, Output: xxxx.rtf, Generated on: DDMONYYYY HH:MM
Page x of y

Programming Note: Repeat listing for Safety Pilot Stage (N=XXX) Placebo (N=XXX), then Safety Primary Stage (N=XXX) GDNF (N=XXX) and Placebo (N=XXX). Sort by stage, treatment group, and subject ID, then reported by, question, behavior, visit, and date. Include all Safety Overall subjects with data. Do not list imputed values. Note that behavior column is not applicable for "Other Behaviors" (subquestions can go in "Behavior" column) and "Medication Use" (leave "Behavior" column blank) sections. For all listings by visit, use actual visit labels and not "screening" or "baseline."

Listing 17.2.4.22 MoCA Total Score by Visit - Safety Overall Population

Safety Pilot Stage (N=XXX)

GDNF (N=XXX)

**Subject ID Age/Race/ Ethnicity/Sex**	**Visit**	**Date**	**Result**
xxxxxxxxxx/xx/x/x/x	Screening Visit 1	DDMONYYYY	x
	Pre-test infusion	DDMONYYYY	x
	Week 40	DDMONYYYY	x
		DDMONYYYY	x
		DDMONYYYY	x
	…	…	…

Note: The MoCA is a rater-administered cognitive screening tool with 8 components. The total score ranges from 0 to 30, with lower scores representing poorer cognitive function. A total score of 26 or above is considered normal. W = white; B = black; P = native Hawaiian or other Pacific islander; A = Asian; I = American Indian or Alaska native; H = Hispanic or Latino; N = not Hispanic or Latino; M = male; F = female.
Source: Dataset: [NAME], Program: xxxxxx.sas, Output: xxxx.rtf, Generated on: DDMONYYYY HH:MM
Page x of y
Programming Note: Repeat listing for Safety Pilot Stage (N=XXX) Placebo (N=XXX), then Safety Primary Stage (N=XXX) GDNF (N=XXX) and Placebo (N=XXX). Sort by stage, treatment group, and subject ID, then visit and date. Include all Safety Overall subjects with data. Do not list imputed values. For all listings by visit, use actual visit labels and not “screening” or “baseline.”

Listing 17.2.4.23 MDRS AEMSS Total Score by Visit - Safety Overall Population

Safety Pilot Stage (N=XXX)

GDNF (N=XXX)

Subject ID Age/Race/ Ethnicity/Sex	Visit	Date	AEMSS Total Score
xxxxxxxxxx/xx/x/x/x	Screening Visit 1	DDMONYYYY	x
	Week 40	DDMONYYYY	x
		DDMONYYYY	x
		DDMONYYYY	x
		DDMONYYYY	x
	…	…	x
			xxx

Note: The MDRS is a rater-administered global scale of cognition including 5 subscales. The total score ranges from 0 to 144, with higher scores representing better cognitive function. A total score lower than 123 is associated with some degree of dementia in PD. W = white; B = black; P = native Hawaiian or other Pacific islander; A = Asian; I = American Indian or Alaska native; H = Hispanic or Latino; N = not Hispanic or Latino; M = male; F = female.
Source: Dataset: [NAME], Program: xxxxxx.sas, Output: xxxx.rtf, Generated on: DDMONYYYY HH:MM
Page x of y
Programming Note: Repeat listing for Safety Pilot Stage (N=XXX) Placebo (N=XXX), then Safety Primary Stage (N=XXX) GDNF (N=XXX) and Placebo (N=XXX). Sort by stage, treatment group, and subject ID, then visit and date. Include all Safety Overall subjects with data. Do not list imputed values. For all listings by visit, use actual visit labels and not “screening” or “baseline.”

Listing 17.2.4.24 Stroop Test by Visit - Safety Overall Population

Safety Pilot Stage (N=XXX)

GDNF (N=XXX)

Subject ID Age/Race/ Ethnicity/Sex	Condition	Visit	Date and Time	Total Uncorrected Errors	Total Self-Corrected Errors	Total Time to Complete (secs)
xxxxxxxxxx/xx/x/x/x	Color Naming	Screening Visit 2	DDMONYYYY HHMM	xx	xx	xx
		Week 40	DDMONYYYY HHMM	xx	xx	xx
	Word Reading		DDMONYYYY HHMM			
	Inhibition		DDMONYYYY HHMM			
	Inhibition/Switching	…	…			

Note: The Stroop test is a global scale of reaction time including 4 conditions. Total time to complete the test in each condition can range from 0 to 999 seconds, with lower time representing better reaction time. W = white; B = black; P = native Hawaiian or other Pacific islander; A = Asian; I = American Indian or Alaska native; H = Hispanic or Latino; N = not Hispanic or Latino; M = male; F = female.

Source: Dataset: [NAME], Program: xxxxxx.sas, Output: xxxx.rtf, Generated on: DDMONYYYY HH:MM

Page x of y

Programming Note: Repeat listing for Safety Pilot Stage (N=XXX) Placebo (N=XXX), then Safety Primary Stage (N=XXX) GDNF (N=XXX) and Placebo (N=XXX). Sort by stage, treatment group, and subject ID, then condition (CRF order), visit, and date. Include all Safety Overall subjects with data. For all listings by visit, use actual visit labels and not "screening" or "baseline."

Listing 17.2.4.25 FrSBe by Visit - Safety Overall Population

Safety Pilot Stage (N=XXX)					
GDNF (N=XXX)					
**Subject ID Age/Race/ Ethnicity/Sex**	**Scale**	**Visit**	**Date**	**Time Point**	**Result**
xxxxxxxxxx/xx/x/x/x	Apathy Score	Screening Visit 2	DDMONYYYY	Before	x
		Screening Visit 2	DDMONYYYY	After	x
		Week 40	DDMONYYYY	After	x
	Disinhibition	…	…	Before	…
	Disinhibition			After	
	Executive Dysfunction			Before	
	Executive Dysfunction			After	

Note: The FrSBe is a scale that assesses behavior related to frontal systems damage including 3 subscales. Higher subscale scores indicate greater pathology. W = white; B = black; P = native Hawaiian or other Pacific islander; A = Asian; I = American Indian or Alaska native; H = Hispanic or Latino; N = not Hispanic or Latino; M = male; F = female.
Source: Dataset: [NAME], Program: xxxxxx.sas, Output: xxxx.rtf, Generated on: DDMONYYYY HH:MM
Page x of y
Programming Note: Repeat listing for Safety Pilot Stage (N=XXX) Placebo (N=XXX), then Safety Primary Stage (N=XXX) GDNF (N=XXX) and Placebo (N=XXX). Sort by stage, treatment group, and subject ID, then scale (CRF order), visit, and date. Include all Safety Overall subjects with data. Baseline value is “after” at Screening Visit 2; Week 40 data has “after” only. Do not list imputed values. For all listings by visit, use actual visit labels and not “screening” or “baseline.”

Listing 17.2.4.26 Deary-Liewald Four-Choice Reaction Time by Visit - Safety Overall Population

Safety Pilot Stage (N=XXX)

GDNF (N=XXX)

Subject ID Age/Race/ Ethnicity/Sex	For correct responses for the four-choice RT: Parameter	Visit	Date	Result (ms)
xxxxxxxxxx/xx/x/x/x	Mean reaction time	Screening Visit 2	DDMONYYYY	x
	Variance	Week 12	DDMONYYYY	x
	SD	Week 24	DDMONYYYY	x
		Week 40	DDMONYYYY	x

Note: The Deary-Liewald RT is a computerized measure of simple and four-choice reaction time. The parameter reported is the mean reaction time, variance, and SD for correct responses for four-choice reaction time. A shorter reaction time is better. W = white; B = black; P = native Hawaiian or other Pacific islander; A = Asian; I = American Indian or Alaska native; H = Hispanic or Latino; N = not Hispanic or Latino; M = male; F = female.

Source: Dataset: [NAME], Program: xxxxxx.sas, Output: xxxx.rtf, Generated on: DDMONYYYY HH:MM

Page x of y

Programming Note: Repeat listing for Safety Pilot Stage (N=XXX) Placebo (N=XXX), then Safety Primary Stage (N=XXX) GDNF (N=XXX) and Placebo (N=XXX). Sort by stage, treatment group, and subject ID, then parameter, visit, and date. Include all Safety Overall subjects with data. For all listings by visit, use actual visit labels and not “screening” or “baseline.”

Listing 17.2.4.27 Verbal Fluency Assessment by Visit - Safety Overall Population

Safety Pilot Stage (N=XXX)

GDNF (N=XXX)

**Subject ID Age/Race/ Ethnicity/Sex**	**Test**	**Visit**	**Date**	**Number of Correct Responses in 60 Seconds**
xxxxxxxxxx/xx/x/x/x	Phonemic Verbal Fluency	Screening Visit 2	DDMONYYYY	xx
		Week 40	DDMONYYYY	xx
	Semantic Verbal Fluency	Screening Visit 2	DDMONYYYY	xx
		Week 40	DDMONYYYY	xx

Note: The verbal fluency assessment measures verbal functioning in 2 categories. Scores represent number of correct words in one minute and range from 0 to 200. Higher scores represent better verbal functioning. W = white; B = black; P = native Hawaiian or other Pacific islander; A = Asian; I = American Indian or Alaska native; H = Hispanic or Latino; N = not Hispanic or Latino; M = male; F = female.
Source: Dataset: [NAME], Program: xxxxxx.sas, Output: xxxx.rtf, Generated on: DDMONYYYY HH:MM
Page x of y
Programming Note: Repeat listing for Safety Pilot Stage (N=XXX) Placebo (N=XXX), then Safety Primary Stage (N=XXX) GDNF (N=XXX) and Placebo (N=XXX). Sort by stage, treatment group, and subject ID, then test, visit, and date. Include all Safety Overall subjects with data. For all listings by visit, use actual visit labels and not "screening" or "baseline."

Listing 17.2.4.28 BDI by Visit - Safety Overall Population

Safety Pilot Stage (N=XXX)			
GDNF (N=XXX)			
**Subject ID Age/Race/ Ethnicity/Sex**	**Visit**	**Date**	**Total Score**
xxxxxxxxxx/xx/x/x/x	Screening Visit 2	DDMONYYYY	xx
	Week 40	DDMONYYYY	
	..		
	Screening Visit 2	DDMONYYYY	
	Week 40	DDMONYYYY	

Note: The BDI is a self-administered test which consists of 21 questions that measure the severity of depression. Scores range from 0 to 63, with higher scores representing worse depression. W = white; B = black; P = native Hawaiian or other Pacific islander; A = Asian; I = American Indian or Alaska native; H = Hispanic or Latino; N = not Hispanic or Latino; M = male; F = female.
Source: Dataset: [NAME], Program: xxxxxx.sas, Output: xxxx.rtf, Generated on: DDMONYYYY HH:MM
Page x of y
Programming Note: Repeat listing for Safety Pilot Stage (N=XXX) Placebo (N=XXX), then Safety Primary Stage (N=XXX) GDNF (N=XXX) and Placebo (N=XXX). Sort by stage, treatment group, and subject ID, then visit and date. Include all Safety Overall subjects with data. For all listings by visit, use actual visit labels and not “screening” or “baseline.”

Listing 17.2.4.29 UPSIT by Visit - Safety Overall Population

Safety Pilot Stage (N=XXX)					
GDNF (N=XXX)					
**Subject ID Age/Race/ Ethnicity/Sex**	**Visit**	**Date**	**Total Number of Correct Responses**	**Percentile Value**	**Descriptive Term**
xxxxxxxxxx/xx/x/x/x					Normosmia/ Mild microsmia/ Moderate microsmia/ Severe microsmia/ Anosmia/ Probable malingering
	Screening Visit 2	DDMONYYYY	xx	xx	
	Week 40	DDMONYYYY	xx	xx	
	..				
	Screening Visit 2	DDMONYYYY			
	Week 40	DDMONYYYY			

Note: The UPSIT is a self-administered test which can be used to identify and quantitate olfactory dysfunction in PD. The number of correct responses out of 40 total items constitutes a subject’s score. Lower scores represent greater olfactory dysfunction. W = white; B = black; P = native Hawaiian or other Pacific islander; A = Asian; I = American Indian or Alaska native; H = Hispanic or Latino; N = not Hispanic or Latino; M = male; F = female.
Source: Dataset: [NAME], Program: xxxxxx.sas, Output: xxxx.rtf, Generated on: DDMONYYYY HH:MM
Page x of y
Programming Note: Repeat listing for Safety Pilot Stage (N=XXX) Placebo (N=XXX), then Safety Primary Stage (N=XXX) GDNF (N=XXX) and Placebo (N=XXX). Sort by stage, treatment group, and subject ID, then visit, and date. Include all Safety Overall subjects with data. For all listings by visit, use actual visit labels and not “screening” or “baseline.”

Document History

Version Date	Modified/Reviewed By	Brief Summary of changes to current version
15-MAY-2015	Debbie Anderson/ Heather Thomas/ Lara Longpre/ Chris Priestley/ Matthias Luz	Draft version 0.1. Created from PRS 005 T 17 E.
05-JUN-2015	Debbie Anderson/ Lara Longpre/ Chris Priestley/ Matthias Luz/ Trial Steering Committee/ John Werth	Draft version 0.2. Incorporated client comments and minor corrections. Added additional imaging endpoints.
30-JUL-2015	Debbie Anderson/ Lara Longpre/ Chris Priestley/ Matthias Luz/ Trial Steering Committee/ Eunhee Hwang/ John Werth	Draft version 0.3. Incorporated client comments, including steering committee advice.
24-SEP-2015	Christopher Tait/ Debbie Anderson/ Lara Longpre/ Chris Priestley/ Matthias Luz	Draft version 0.4. Incorporated client comments and scoring information for some of the QoL instruments.
16-OCT-2015	Debbie Anderson/ Lara Longpre/ Chris Priestley/ Matthias Luz/ Trial Steering Committee/ Eunhee Hwang/ John Werth	Draft version 0.5. Incorporated client comments and further scoring information.
13-NOV-2015	Debbie Anderson/ Lara Longpre/ Chris Priestley/ Matthias Luz	Version 1.0. Incorporated client comments and further scoring information. Added Appendix 3 (placeholder) for levodopa-containing medications. Corrected visit labels for listings. Added/ revised text pertaining to PD diary data.
02-DEC-2015<br>03-DEC-2015<br>04-DEC-2015	Debbie Anderson/ Lara Longpre/ Chris Priestley/ Matthias Luz	Version 1.0. Added content in Appendix 3. Added levodopa equivalents analysis per client.
04-JAN-2016	Debbie Anderson	Changed “enrolled” population definition to account for subjects enrolled despite not meeting entry critieria.
04-MAY-2016	Emma Lewis/ Lara Longpre/ Chris Priestley/ Matthias Luz	Draft version 1.1. Made updates following the pre-freeze data review meeting.
10-JUN-2016<br>14-JUN-2016<br>15-JUN-2016<br>16-JUN2016	Emma Lewis/ Lara Longpre/ Lynn Barclay/ Chris Priestley/ Matthias Luz	Version 2.0. Updates made following the post-freeze data review meeting.
