# Supplementary material for: Randomized trial of intermittent intraputamenal glial cell line-derived neurotrophic factor in Parkinson’s disease
Source: Brain. 2019 Feb 26;142(3):512–25. doi: 10.1093/brain/awz023 (PMC6391602; doi:10.1093/brain/awz023)
Supplement: Supplementary Data [file awz023_supp.zip › awz023-suppl_data/awz023_Supplementary_Data_S6.pdf]

Statistical Analysis Plan Change Log

Section / Table / Listing	Page	Comment
Section 5, Enrolled subject	18	Modified definition of enrolled subject to include subjects enrolled despite not meeting an entry criterion.
Listing 17.2.1.5.3	153	UPPS collected only for Primary Stage subjects, not Pilot Stage subjects.
Multiple	NA	Several updates made following the pre-freeze data review meeting on 08Mar2016. Details of which can be found in the meeting minutes.
Appendix 3	51	List of AEs of special interest preferred terms updated
Table 16.1.6.1.1	81	Table deleted as only high level information required which is already included in T16.1.4.1
Listings 17.2.1.7.1, 17.2.1.7.2	170, 171	Listings combined into one
Section 9.1.2	25	Clarification regarding subject 045 added
Section 9.9.13, Listing 17.2.4.21	43, 204	Title updated to QUIP items answered with yes by visit for clarity
Section 9.9.2.1, Table 16.4.2.4.1	39, 124	Updated to show mean and range rather than number of visits
Section 5.0 Table 3	22	Visit windows for Week 36 changed to 239-257 and Week 40 changed to 258+
Table 16.1.1.1	67	Changed title to all enrolled subjects and removed initial informed consent rows
Listing 17.2.2.3.2	182	Corrected footnote to say more taps represent better function
Table 16.3.4.1	112	Added n
Table 16.4.1.1.1	114	Changed second section to Total GDNF exposure
Table 16.4.2.1.1	118	Added a serious pre-treament AE row
Table 16.1.9	87	Title changed to prior to study start as one subject had test infusion after randomization
Section 9.1.5	26	Section added to detail special handling of subject 45 data
Approvals	2	NBT representative changed
Section 1.0	5	Tense changed in third paragraph
Section 5.0 and Appendix 3	16, 51	Definition of adverse changes in mood and impulsivity updated
Section 5.0 Table 3	22	Upper window for Week 40 changed to 304
Multiple	NA	Subject 045 changed to 45 throughout to match what is displayed in the listings
Section 9.2	26	Text describing the screen failure data added

Section 9.6.2, Table 16.1.9	28, 82	Prior to randomization changed to prior to start of study medication
Section 5.0 Table 3	22	Visit window for Week 40 changed to 260-304. Sentence added describing rationale for wider Week 40 window. Sentence added detailing major protocol deviations for Week 40 UPDRS assessment being before Day 267 or after Day 294.
Approvals	2	Updated Dr Whone's address

Medgenesis Pre-Freeze Data Review Meeting 08Mar2016

Excerpts regarding SAP changes

Tables:

- Show 2-digit subject numbers only
- Show "visit name (baseline)" usually Week 0 (baseline), there are some exceptions where we have "Screening (baseline)"
- Listings should always show scheduled visit
- In order to consistently exclude all Pilot subjects from the per-protocol analysis, the definition of the per-protocol population will start with the ITT primary population and then exclude subjects with major deviations (needs update in SAP2).
- Some major protocol deviations will be reclassified to minor due to complex protocol amendments.
- Sort AEs and Medical History alphabetically by SOC and by PT within SOC, not international sort order (need to update in SAP2)
- Ensure that category headings are not separated from following items at pagebreaks.
- Timed walk/tap: if 2nd repeat is missing, impute with worst time in entire dataset for 2nd reading, only do this if 1st reading is not missing (needs update in SAP2). If both missing, no imputation. Just leave as missing
- Append protocol deviation guidance document to SAP2
- Add minor deviation frequency (categories only, not deviation brief description) to PDV table for ITT Primary Population. Delete major PDV table for ITT overall population.
- Table 16.1.3.1 - reduce height and bmi by 1 decimal place
- 2 subjects enrolled but not randomized. Need to be added to T16.1.1.1 when PD page entered, please reference listings 17.2.1.2.2, 17.2.1.2.3 and 17.2.1.4
- T16.1.4.1 Make NART footnote "b". Move NART to demography table (T16.1.3.2) instead of PD history then it will be footnote "a".
- Demographics summary of PD meds will not be different from PD meds table, just more detail for PD meds table.
    - For demographic T16.1.4.1, count COMT inhibitors under levodopa prep and under COMT inhibitors (as done on CRF)-just major categories (in bold), screening
    - For prior PD meds table 16.1.6.1.1, use all major codes and minor codes from levodopa equivalents page
    - Use screening
    - This needs to go in SAP2 and footnote to T16.1.6.1.1, rename to "at screening" not "prior".
- Leave T16.1.6.2.1 as is for conmeds
- New listing for levodopa equivalent meds data needed, add to efficacy section
- T16.1.6.1.2 - Fix cut off med names
- T16.1.7 Add initial surg and repositioning surgeries

- T16.1.8 also add initial surg and repositioning surgeries
- T16.1.8
  - Change calculations to use all individual catheter records for summary stats
  - This table will need 1 more decimal place, because raw data will get 1 decimal place
  - Show 3 columns, GDNF, placebo, total
  - N will be the number of subjects in each treatment group and n will be the number of catheters for each treatment group. Add footnote for clarity.
- 045JXG
  - Subject with broken back.
  - Subject will do UPDRS items as possible.
  - We will only count change from baseline for items she can do (refer to filenote for further details)
  - Needs to go in SAP2
  - Will be a major protocol deviation
- T16.2.1.4
  - Add screening, rename "Baseline" to "Week 0 (baseline)"
  - Add screening for this table only, no other UPDRs tables
- T16.2.3.1
  - Add footnote defining good quality on time per day
  - Add screening to this table for all parameters
  - Use 1 decimal place minimum, maximum, 2 decimals mean, 3 for standard deviation, etc.
- T16.2.4.1 and T16.2.4.2
  - Change footnote about handling of missing 2nd test
  - All QOL and cognitive function tables, sort total scores first ahead of subscales"cardiovascular including falls score"
  - Report overall score first
- T16.2.4.3 – remove total from first row and show total score first
- T16.2.4.4 –move total to beginning
- T16.2.4.5.1
  - Do not put vas first (keep as separate table T16.2.4.5.2)
- T16.4.1.1.1 and T16.4.1.1.2
  - Take out "(#)" in 2 places
- T16.4.2.1.1 and T16.4.2.1.2
  - Add footnotes to describe definition of pre-treatment and TEAEs
- T16.4.2.3.1
  - Replace "#" with "n" subjects, "n" events
  - Calculation of >=3 should be within GDNF or placebo only not combined
- T16.4.2.4.1 and T16.4.2.4.2
  - Change number of events to number of visits event occurred after infusion (count the number of unique visits with that TEAE within 7 days-will be 3,4,5 etc)

- T16.4.2.12
  - Footnote population definition for safety enrolled population
  - Change to # of visits
- T16.4.2.13
  - Footnote population definition for safety enrolled population
- T16.4.2.14
  - Footnote population definition for safety enrolled population
- T16.4.2.15
  - Footnote population definition for safety enrolled population
- T16.4.3.1.1
  - Remove "n(%)" from column headers
  - Keep "screening-(baseline)" as is
- T16.4.3.1.2
  - Change total column to missing
  - Remove total row
  - Make same updates to similar shift tables
- T16.4.3.2.1
  - Remove "n(%)" from column headers
  - Keep "screening-(baseline)" as is
  - Remove GFR parameter
  - In listings put >90 if it's for GFR
- T16.4.6
  - As per SAP, present for all test infusion visits and for all study medication infusion visits
  - Add "...... at any visit" as first item for each parameter, followed then by each visit separately
  - "Sitting pulse <50bpm"- remove units and "&"
  - "At any visit"-remove units and "&"
  - Week 0-remove units and "&"
  - Page 291 n (%) should be on week 20 row
  - Page 301, remove criterion section
- T16.4.8.2
  - "Summary of..." not "abnormal" in title
  - Add "normal" category as first subcategory under "overall impression"
  - Change second part of table to read as follows: Any clinically relevant abnormal Week 40 **QTc** .....
  - In second part of table, keep all categories even if 0
  - Remove endnote and use those as categories
  - Check QTC interval week 40 change from B/L of 41 in T16.4.8.1-should have been caught for this table!
- T16.4.9
  - Delete "Subjects with" from table title
  - Keep endnote page

- T16.4.10.1
  - Remove total row and total column
- T16.4.10.2, T16.4.10.3 and T16.4.10.4
  - Remove "n(%)" from column header
- T16.10.5
  - Before score is before Parkinson's
  - Rename for each parameter (screening "before", screening "after" (baseline), week 40 "after")
- T16.4.10.6
  - Update title to "Deary-liewald four-choice reaction time by visit"
  - Remove "n(%)"
- T16.4.10.7
  - Remove "n(%)"
- T16.4.10.8
  - Remove "n(%)"
- T16.4.10.9
  - Remove "n(%)"

Figures:

- Add figure of individual observed subject scores by visit for UPDRS Part III OFF using ITT primary
  - GDNF page and placebo page separate
  - Y axis to the same scale (if possible)
- Add summary figure like 16.5.1.1. for timed tap test left and right hand
- Add individual subject plot for timed tapping test left and right hand
- Please check (Part III) or (part III) in titles and axis labels for consistency, use motor throughout
- F16.5.1.1
  - Add motor to y-axis and remove "Part III"
- F16.5.1.2
  - Add motor to y-axis and remove "Part III"
- F16.5.2.2
  - Add footnote defining good quality on time per day

Listings:

- L17.2.1.2.1 – Last visit should be week 40 for all subjects, currently data issues for example week 16 25APR2014 probably should be 2013
- L17.2.1.4
  - Use reason as PD description instead of category
- L17.2.1.5.1
  - Move NART to demography listing with its footnotes
- L17.2.1.6
  - Capitalise verbatim term in column header to match listing content

    - Remove study day
- L17.2.1.9
    - Change to catheter 1-4
    - Remove all refs to RA/RP/LA/LP including footnote
    - Change to "mean across all catheters" and show 1 decimal
    - Correct spelling of "haemorrhage"
- L17.2.1.10 and L17.2.4.1.2 (IP infusion)
    - Standard or non-standard only-delete description
    - Footnote to define standard:
    - "Standard regime is a linear ramp up of 3-5 µL/min with 400µL total volume"
        - 3µL/min-400µL
        - 5µL/min-400µL
        - 5µL/min-800µL
- L17.2.1.11 and L17.2.4.1.3
    - List Catheters 1-4 separately with all starts and stops for each on separate rows.
- Add levodopa equivalent dose listing (new) to efficacy section in SAP2 and make a shell
- L17.2.2.1
    - Remove seconds
    - Add space between date and time instead of :
- L17.2.2.2
    - Two decimals only maximum
- L17.2.2.3.1
    - Remove seconds
    - Add space between date and time instead of : (applies to all listings with Date and Time)
- L17.2.2.4
    - Remove "total" from subscores and put NMSS total score first
- L17.2.2.5
    - Sort single index (total) score first
- L17.2.4.2
    - "Verbatim Term" in capitals (apply also to any similar listings)
- L17.2.4.11
    - GFR with SHOIV >90 when appropriate
- L17.2.4.17
    - Remove height and update title
- ECG tables - add footnote documenting QTc correction method (Hodges)
- L17.2.4.20
    - Request to only list results <15 then list all parameters at that visit
    - Add "results<15" to title after "Glasgow Coma Scale"
- L17.2.4.21
    - Only list yes results.
    - Change title to "Positive QUIP results by visit".

- Only list behaviours with yes & delete last column.

- L17.2.4.22
  - Total score only.
  - Not subscale data-omit from listing.
- L17.2.4.23
  - Total score only.
- L17.2.4.24
  - Date &time – remove seconds-insert space between.
- L17.2.4.25
  - Before/after is fine-no change.
- Vital Signs Listing L17.2.4.16
  - Limit data directly supporting table, only list out of range values
  - Also list all values of the parameter at that visit (all time points) for that subject
  - Example: Spike in BP during infusion, answer: list all BP for that subject at that infusion (all time points).
  - Rename to: "vital signs from infusions there was a clinically relevant abnormal result" and add footnote to clarify what is listed.
