# Supplementary material for: Randomized trial of intermittent intraputamenal glial cell line-derived neurotrophic factor in Parkinson’s disease
Source: Brain. 2019 Feb 26;142(3):512–25. doi: 10.1093/brain/awz023 (PMC6391602; doi:10.1093/brain/awz023)
Supplement: Supplementary Data [file awz023_supp.zip › awz023-suppl_data/awz023_Supplementary_Data_S7.pdf]

Protocol GDNF 2553

A Placebo-Controlled, Randomized, Double-Blind Trial to Assess the Safety and Efficacy of Intermittent Bilateral Intraputamenal Glial Cell Line-Derived Neurotrophic Factor (GDNF) Infusions Administered via Convection-Enhanced Delivery (CED) in Subjects with Parkinson's Disease

Statistical Analysis Plan
Post Hoc Analysis
(eSAP)
Final Draft

Version: 1.0 – FINAL

Author: Eve Pickering, Ph.D., BTx Statistics, Neuroscience

Date: 10 November 2016

This Document is Company Confidential.

CONTENTS

1 AMENDMENTS FROM PREVIOUS VERSION(S) 3

2 INTRODUCTION 3

2.1 STUDY DESIGN 3
2.2 STUDY OBJECTIVES 3

3 INTERIM ANALYSES, FINAL ANALYSES AND UNBLINDING 4

3.1 INTERIM ANALYSIS 4
3.2 UNBLINDING 4

4 HYPOTHESES AND DECISION RULES 4

4.1 STATISTICAL HYPOTHESES 4
4.2 STATISTICAL DECISION RULES 4

5 ANALYSIS SETS 4

6 ENDPOINTS AND COVARIATES 4

6.1 EFFICACY AND IMAGING ENDPOINTS 4
6.2 SAFETY ENDPOINTS 7
6.3 PHARMACOKINETIC ENDPOINTS 7
6.4 PHARMACODYNAMIC ENDPOINTS 7
6.5 COVARIATES 7

7 HANDLING OF MISSING VALUES 7

8 STATISTICAL METHODOLOGY AND STATISTICAL ANALYSES 8

8.1 EFFICACY ANALYSES 8
8.1.1 OFF State UPDRS Motor Score (part III) and Subscales 8
8.1.2 Responder Analysis 8
8.1.3 L-Dopa Responsiveness 9
8.1.4 Additional Covariates for UPDRS Motor Score 9
8.1.5 Subgroup Analyses 9
8.2 IMAGING ANALYSES 10

9 REFERENCES 11

10 APPENDICES 11

10.1 FINAL MINUTES FROM DATA DEEP DIVE – JULY 19-21, 2016 11
10.2 CALCULATION OF UPDRS MOTOR SCORE SUBSCALES 14
10.3 DEFINITION OF RESPONDER 14
10.4 DEFINITION OF L-DOPA RESPONSIVENESS 15
10.5 IDENTIFICATION OF PREDOMINANTLY AFFECTED BODY SIDE 15
10.6 LIST OF GENES INCLUDED IN TARGETED GENOTYPING 16
10.7 DATA TO BE PROVIDED BY MEDGENESIS/PRA 17

This Document is Company Confidential.

1 AMENDMENTS FROM PREVIOUS VERSION(S)

None

2 INTRODUCTION

This post hoc Statistical Analysis Plan (SAP) supplements the original SAP (version 2.0, dated June 16, 2016) for GDNF protocol 2553 sponsored by North Bristol NHS Trust (NBT). It provides for specific additional analyses requested at a Data Deep Dive Review Meeting held between NBT, MedGenesis, and Pfizer in Victoria, BC, Canada on July 19-21, 2016 after the final study results became available (see minutes in Appendix 10.1). It also references the protocol for GDNF 2553 (version 1.7, dated October 28, 2015).

This SAP describes analysis of source data collected during the conduct of GDNF protocol 2553. New endpoints analyzed in this SAP are derived from source data available at the time of final reporting. For example, the existing full UPDRS motor score (part III) is broken into its subscales and the subscales are analyzed (see Section 6 and Appendix 10.2). One exception to this is that, pending consent, subjects participating in GDNF protocol 2553 may provide DNA samples for genotyping of mutations associated with PD.

Refer to the study protocol and original SAP for information about the study design and analysis sets. In this document text extracted verbatim from the protocol or original SAP *is italicized.*

2.1 STUDY DESIGN

Refer to study protocol for details.

2.2 STUDY OBJECTIVES

The following are the study objectives as stated in the protocol:

Primary Objectives

- *To assess the effect of 4-weekly intermittent bilateral intraputamenal GDNF infusions on OFF state motor function at 9 months.*

Secondary Objectives

- *To assess the effect of intermittent bilateral intraputamenal GDNF infusions on ON state motor function, motor complications, and ON and OFF state activities of daily living (ADL) at 9 months.*
- *To assess the safety of intermittent bilateral intraputamenal GDNF infusions in a small pilot cohort of subjects and in the full study population.*

This Document is Company Confidential.

Other Objectives

- *To explore the effects of intermittent bilateral intraputamenal GDNF infusions on other motor and non-motor functions, quality of life (QOL) assessments, and imaging endpoints at 9 months.*

3 INTERIM ANALYSES, FINAL ANALYSES AND UNBLINDING

3.1 INTERIM ANALYSIS

No interim analyses were performed.

3.2 UNBLINDING

Fully cleaned, unblinded, ready-for-use datasets are provided for this post hoc analysis.

4 HYPOTHESES AND DECISION RULES

Not applicable.

4.1 STATISTICAL HYPOTHESES

Not applicable.

4.2 STATISTICAL DECISION RULES

Not applicable.

5 ANALYSIS SETS

Analysis sets are defined in the original SAP and flagged in the datasets provided for use in the analyses. See original SAP for details. All analyses under this post hoc SAP will be done using the ITT Overall population (ITTFL=”Y”) as the analysis population.

6 ENDPOINTS AND COVARIATES

All summary presentations will be according to the scheduled time point designators. All analyses will be conducted according to the scheduled time points and in the case of assessments made outside of the specified visit window, according to the recorded assessment time (see section 5.0 of the original SAP for definition of windows).

6.1 EFFICACY AND IMAGING ENDPOINTS

The endpoints that will be used in the post hoc analysis are described below.

This Document is Company Confidential.

Efficacy Endpoints

One set of post hoc endpoints is based on the subscales of the UPDRS motor score (part III). The motor score can be divided into 5 subscales of:

(a) tremor,
(b) rigidity,
(c) bradykinesia,
(d) postural instability/gait disorder (PIGD), and
(e) other, consisting of the items not included in subscales (a)-(d).

In addition, a non-tremor UPDRS motor score is derived as a sixth subscale by subtracting the tremor subscale score (a) from the full UPDRS motor score (see Appendix 10.2).

Subjects will be identified as responders or non-responders based on the UPDRS motor score and/or good quality ON-time as reported in the PD diaries (see Appendix 10.3), and the change from baseline in responsiveness to L-dopa following levodopa challenge will also be calculated (Appendix 10.4).

Videos recorded during the motor outcome assessments at baseline and Week 40 will be assessed by an external movement disorders specialist for the assessment of a Clinical Global Impression of Change (CGIC), defined as a rating of improvement or deterioration in motor function in the OFF state. For the assessments, the videos are blinded for treatment and visit and randomly labeled A and B. After reviewing the two videos for a subject, the clinician will report whether the clinical global impression observed in Video B is better or worse than that in Video A, using a 7 point Likert scale. For the analysis, the ordering of assessments will then be de-coded and the comparison direction adjusted so that a positive score implies improvement over time and a negative score implies deterioration over time.

In addition, the predominantly affected body side (left, right or symmetric) of each subject will be identified (see Appendix 10.5). This information will be used to match symptoms with the contralateral PET response.

Imaging Endpoints

Three endpoints determining GDNF distribution characteristics by means of voxel-by-voxel, concentration-based volume analysis using gadolinium (Gd)-enhanced T1-weighted MRI will be assessed. Details of the calculations and modeling assumptions used to derive these endpoints may be found in the Imaging Memo, and all algorithms developed for this analysis have been internally validated and passed QC.

- Volume of distribution – The volume of distribution is the number of voxels in the vicinity of the striatum that are above a minimum threshold in modeled apparent Gd concentration, multiplied by volume of a single voxel.

This Document is Company Confidential.

- Total putamenal coverage –The putamenal coverage is the volume of the intersection in space of the voxels identified as belonging both within the volume of distribution of the contrast agent and within the volume of the putamen as estimated from the T1-weighted image. The putamenal coverage will also be expressed as a percentage of the volume of putamen.
- Coverage in the predefined volume of interest (posterior 2/3 of dorsal half of the putamen) –This is the volume of the intersection in space of the voxels identified as belonging both within the volume of distribution of the contrast agent and within the posterior 2/3 of the dorsal half of the putamen as estimated from the T1-weighted image. The volume of interest coverage will also be expressed as a percentage of the volume of interest.

The following five newly defined regions of interest (ROIs) will be used for the assessment of ^{18}F-DOPA uptake by means of positron emission tomography (PET):

- Dorsal striatum (including 4 slices dorsal to the anterior commissure)
  - Caudate nucleus
  - Anterior putamen
  - Central/posterior putamen
- Ventral striatum (including the slice containing the anterior commissure and one slice ventral to the anterior commissure)
  - Anterior/central/posterior putamen
- Substantia nigra

These ROIs will be assessed by laterality (left, right) and over the total region (both sides), for a total of 15 assessments.

Post Hoc Analysis Endpoints:

- *Percentage change from baseline in the practically defined OFF state UPDRS motor score (part III)* and each of the 6 subscales *after 9 months of double-blind treatment.*
- Raw (absolute) change from baseline in the practically defined OFF state UPDRS motor score (part III) and each of the 6 subscales after 9 months of double-blind treatment.
- *Change from baseline* (raw and percentage) *in ^{18}F-DOPA uptake* in each of the 5 newly defined ROIs *as determined by PET scans after 9 months of double-blind treatment and after 3 months of double-blind treatment (Pilot Stage).*
- CGIC as obtained from expert video review time-blinded comparison of baseline to 9 months of double-blind treatment.
- Change from baseline in L-dopa responsiveness after 9 months of double-blind treatment.
- Responder status after 9 months of double-blind treatment based on criteria described in Appendix 10.3

This Document is Company Confidential.

- Change from baseline in each of the three distribution-related endpoints, determined as described above after 9 months of double-blind treatment.
- Genotype or haplotype on selected list of mutations associated with PD (see Appendix 10.6). Analyses making use of genetic information will be detailed in an addendum to this post hoc SAP when the data are available.

6.2 SAFETY ENDPOINTS

Not applicable.

6.3 PHARMACOKINETIC ENDPOINTS

Not applicable.

6.4 PHARMACODYNAMIC ENDPOINTS

Not applicable.

6.5 COVARIATES

As stated in the original SAP there are no baseline phenotypic covariates assessed in the primary analysis model other than baseline endpoint scores. In this post hoc analysis, covariates of interest are:

- Age
- Sex
- L-Dopa responsiveness at baseline
- Hoehn and Yahr stage in OFF state at screening
- Duration since PD diagnosis
- Duration since first PD symptom
- Dorsal central/posterior putamen PET Kocc at baseline (contralateral to the predominantly affected body side)
- Presence of known genetic variants associated with PD (when available)

7 HANDLING OF MISSING VALUES

See original SAP.

This Document is Company Confidential.

8 STATISTICAL METHODOLOGY AND STATISTICAL ANALYSES

8.1 EFFICACY ANALYSES

8.1.1 OFF State UPDRS Motor Score (part III) and Subscales

The post hoc analysis of the 6 practically defined OFF state UPDRS motor score (part III) subscales will duplicate the model in the original SAP for both (a) percentage change from baseline and (b) raw change from baseline. The primary time point of interest is Week 40. Summary results from the model will include:

(i) Two-sided P-value for the treatment effect (GDNF – placebo)

In addition, "spaghetti plots" will be generated showing subject-level data over time. There will be a two-color plot (one color per treatment) of all subjects together, and a pair of plots with one plot per treatment group (one color per subject). A set of plots will be prepared for each of the following:

(a) Raw values (including screening and baseline values)
(b) Percentage change from baseline
(c) Raw change from baseline

Graphs should be made in color for ease in tracing a particular subject's trajectory over time. Scatterplots of (1) change from baseline in UPDRS motor score (part III) subscale at Week 40 vs baseline (x-axis) and (2) percentage change from baseline in UPDRS motor score (part III) subscale at Week 40 vs baseline (x-axis) will be generated, with treatments coded by symbol and color.

8.1.2 Responder Analysis

The five types of responders are described in Appendix 10.3. For each responder type, the analyses will be done in the same manner: the proportion of responders in each treatment group will be compared using an exact Fisher's test.

In addition, characteristics of the UPDRS motor score responders will be explored by summarizing each of the following factors for the responders and non-responders by treatment:

- Age
- Sex
- L-Dopa responsiveness at baseline
- Screening OFF state UPDRS III score
- Baseline OFF state UPDRS III score
- Change from screening to baseline in OFF state UPDRS III score
- Hoehn and Yahr stage in OFF state at screening
- Duration since PD diagnosis
- Duration since first PD symptom

This Document is Company Confidential.

- Dorsal central/posterior putamen PET Kocc at baseline contralateral to predominantly affected side
- Volume of distribution, VOI coverage and total putamenal coverage at baseline (test infusion), determined as described in section 6.1
- Genotype and/or haplotype (when data are available)

There will be a univariate summary of the distributions of the covariates within each treatment group for responders and non-responders including N, mean, standard deviation, minimum and maximum. Side-by-side boxplots will be generated. An exploratory multivariate classification/prediction tree method will be applied using all the baseline characteristics defined above to identify responders using multiple baseline characteristics. Due to the small sample size, this analysis is not pre-specified, but the analysis process will be documented in a supplement to this document.

8.1.3 L-Dopa Responsiveness

The change from baseline in L-Dopa responsiveness will be assessed using the primary MMRM model. The LS mean treatment difference at 40 weeks will be reported.

8.1.4 Additional Covariates for UPDRS Motor Score

The original MMRM analysis of (a) percentage change from baseline and (b) raw change from baseline in UPDRS motor score (part III) will be augmented by adding covariates of age, sex, Hoehn and Yahr stage in OFF state at screening, duration since PD diagnosis, duration since first PD symptom, baseline L-dopa responsiveness and dorsal central/posterior putamen (contralateral to the predominantly affected body side) PET Kocc at baseline.

For this analysis, the MMRM analysis will be performed adding a single covariate and its interaction with treatment, followed by a multivariate backwards elimination analysis based only on the Week 40 data. The final model results comparing GDNF to placebo based on backwards elimination will be provided, along with the estimates of the least squares (LS) mean treatment effect at Week 40 for the single covariate models.

8.1.5 Subgroup Analyses

The original MMRM analysis of (a) percentage change from baseline and (b) raw change from baseline in UPDRS motor score (part III) will be performed on the subset of subjects having screening H-Y score = 2. If sufficient numbers of subjects with baseline H-Y score > 2 are available, the two subgroups will be compared.

Additional subgroup analyses may be added based on the results of the responder and/or covariate analyses described above.

This Document is Company Confidential.

8.2 IMAGING ANALYSES

The post hoc analysis provides assessment of the association between (1) UPDRS-derived and (2) distribution derived endpoints and ^{18}F-DOPA uptake at Week 40 in each of the 5 newly defined ROIs. Specifically, the following 10 associations will be assessed:

1. *Correlation between* change from baseline (raw and percentage) in both full and non-tremor UPDRS motor score (part III) and percentage *change from baseline in* *^{18}F-DOPA uptake* in each of the 5 newly defined ROIs (pooled across both sides), *as determined by PET scans after 9 months of double-blind treatment.*
2. Correlation between change from baseline (raw and percentage) in both full and non-tremor UPDRS motor score (part III) and absolute ^{18}F-DOPA uptake at Week 40 in each of the 5 newly defined ROIs (pooled across both sides), as determined by PET scans after 9 months of double-blind treatment.
3. *Correlation between* change from baseline (raw and percentage) in both full and non-tremor UPDRS motor score (part III) and percentage *change from baseline in* *^{18}F-DOPA uptake* in each of the 5 newly defined ROIs contralateral to the predominantly affected side, *as determined by PET scans after 9 months of double-blind treatment.*
4. *Correlation between* change from baseline (raw and percentage) in both full and non-tremor UPDRS motor score (part III) and absolute *^{18}F-DOPA uptake* at Week 40 in each of the 5 newly defined ROIs contralateral to the predominantly affected side, *as determined by PET scans after 9 months of double-blind treatment.*
5. Correlation between change from baseline in good quality ON time per day vs percentage change from baseline in ^{18}F-DOPA uptake in each of the 5 newly defined ROIs (pooled across both sides) as determined by PET scans after 9 months of double-blind treatment.
6. Correlation between change from baseline in good quality ON time per day vs absolute ^{18}F-DOPA uptake at Week 40 in each of the 5 newly defined ROIs (pooled across both sides) as determined by PET scans after 9 months of double-blind treatment.
7. Correlation between change from baseline in OFF time per day vs percentage change from baseline in ^{18}F-DOPA uptake in each of the 5 newly defined ROIs (pooled across both sides) as determined by PET scans after 9 months of double-blind treatment.
8. Correlation between change from baseline in OFF time per day vs absolute ^{18}F-DOPA uptake at Week 40 in each of the 5 newly defined ROIs (pooled across both sides) as determined by PET scans after 9 months of double-blind treatment.
9. *Correlation between percentage change from baseline to Week 40 in ^{18}F-DOPA uptake* in both dorsal central/posterior putamen and substantia nigra *as determined by PET scans and percent VOI coverage at baseline*, determined as described above.

This Document is Company Confidential.

10. *Correlation between percentage change from baseline to Week 40 in ^{18}F-DOPA uptake* in each of the 5 newly defined ROIs *as determined by PET scans and percent total putamenal coverage at baseline*, determined as described above.

Associations listed in 1-8 will be assessed considering the F-DOPA endpoints (percentage change from baseline, absolute uptake at Week 40) as the independent endpoint, and UPDRS based endpoints or PD diary-derived time estimates as the dependent endpoint. Analyses will include (a) Pearson correlation coefficient by treatment group, (b) scatterplots, and (c) slope estimates from a linear model including treatment, F-DOPA endpoint and the interaction between them, adjusted for baseline of the dependent endpoint. Covariates (see section 6.5) found to be important in assessing UPDRS based endpoints may be added to the model as a sensitivity analysis.

Associations listed in 9-10 will be assessed considering the coverage endpoint as the independent endpoint and the F-DOPA endpoint as the dependent endpoint. Analyses will include (a) Pearson correlation coefficient by treatment group, (b) scatterplots, and (c) slope estimates from a linear model including treatment, coverage endpoint and the interaction between them, adjusted for baseline of the dependent endpoint. Covariates (see section 6.5) found to be important in assessing UPDRS based endpoints may be added to the model as a sensitivity analysis.

Scatterplots will indicate treatment by symbol/color and the Pearson's correlation coefficient will be provided for each treatment and for data combined.

9 REFERENCES

10 APPENDICES

10.1 FINAL MINUTES FROM DATA DEEP DIVE – JULY 19-21, 2016

Deep Dive Meeting (DDM) Overall Summary
The joint team reviewed Study 2553 data that was available. The team reviewed data across most domains of the study although the most time was spent discussing the UPDRS part III data (the primary efficacy endpoint) and [^{18}F]DOPA uptake data. Although the study did not meet its primary endpoint, there was unanimous agreement that the study provided high-quality data. There was a very robust effect on putamenal [^{18}F]DOPA uptake as measured by PET imaging. In particular, the magnitude of the effect in the posterior putamen (i.e. a mean increase of 100% at Week 40 vs. baseline in GDNF-treated subjects) is unprecedented. The Ki values of [^{18}F]DOPA in the GDNF treated group improved to levels comparable to those observed among mild PD subjects and ½ of those in healthy controls. However the motor outcome showed minimal improvement by GDNF, and reflect greater impairment than is seen among mild PD subjects. While there was not statistical separation between treatment groups on any clinical measure, most clinical endpoints demonstrated a numeric advantage for GDNF treatment compared to placebo. The consistency of this observation across multiple endpoints lends further support to the interpretation that GDNF-CED may have had an effect - albeit one that is too small to demonstrate statistical significance. The clinical

This Document is Company Confidential.

effect observed in the placebo group, though greater than expected, was consistent with historical data. The discrepancy between the biomarker (PET) result and the clinical endpoints is not understood. Several possible explanations were discussed: 1) Although there was a roughly 100% increase in PET signal in the posterior putamen, this may not represent enough restoration of dopaminergic function to result in measurable clinical benefit, 2) There may be a temporal disconnect (a lag or hysteresis) between observed PET effect and clinical benefit, 3) the study population may be too advanced to show clinical improvement of the necessary magnitude, 4) the primary outcome parameter (OFF-state UPDRS motor score) may be too blunt of an instrument to detect improvement following GDNF treatment, 5) there may be no relationship or a lack of translation between improvement in [^{18}F]DOPA uptake and improvement in OFF-state UPDRS motor score.

Numerous additional analyses that were not included in the statistical analysis plan (SAP) were identified. These post hoc analyses are intended to help provide a better understanding of the study results and to aid in drawing conclusions. Pfizer statistics will draft a supplemental SAP for review by DDM participants by ~Aug 6. Most of the analyses identified at the DDM will be included in this supplemental SAP though some may be done outside of it.

Sequence and timing of DDM actions and analyses

Both the SAP-specified analyses and the post hoc analyses will be used to interpret Study 2553 results and to inform possible next steps in GDNF-CED development. One goal is to uncover findings that will provide a compelling scientific rationale for continuing to treat the current study subjects beyond the planned end of Study 2797 (Open-label Extension Study). The Pfizer TRC will review the complete Study 2553 results, the currently available Study 2797 results (15 subjects; 6 Pilot Stage, 9 Primary Study) and any proposed future development plans including core protocol elements of any proposed studies. This may be accomplished through two separate TRC reviews. In addition to the formal process of TRC review of the results and forward plan, the Pfizer team will communicate the outcome of the DDM including recommendations to the Category Triad in an informal manner by Aug 5.

Listing of agreed-to action items (additional analyses)

Clinical endpoints

1) Construct patient "profiles" to capture all key data per subject
2) Conduct a "responder"analysis. Responder was defined in three different ways: UPDRS part III OFF-state improvement: a) ≥5 pts, b) ≥10 pts (BL to Week 40); Diary responder: ≥1 hour increase in good quality ON time (BL to Week 40)
    a) Characterize responders and non-responders using univariate summary and other classification methodology
3) Repeat primary endpoint analysis with additional covariates (covariates to be proposed by Eve & Eunhee)
4) Conduct central-rater assessment of Part III videos in a randomized fashion; collect CGI-severity using 7-point Likert scale per assessment and calculate change at Week 40 (Jim)
5) Reconstruct individual slope from baseline to Week 40 for ITT overall (Tim Nicholas; low priority)

This Document is Company Confidential.

6) Evaluate correlation of [^{18}F]DOPA CFB on the putamen contralateral to the predominantly sided Part III findings (i.e. don't use an average of the bilateral putamen values) (Alan to provide sidedness information to Tim McC)
7) Calculate L-dopa responsiveness at BL and Week 40 (GDNF vs. placebo) to determine whether GDNF renders the subject more sensitive (MMRM)
8) Define phenotype of PD symptoms for each subject: tremor-predominant, bradykinesia-predominant, rigidity-predominant, etc. (Alan/trial team to provide for each subject)
9) Derive UPDRS motor subscales of tremor, rigidity, bradykinesia, and PIGD and repeat the MMRM analyses (4 comparisons instead of 1)
10) Derive UPDRS motor score without tremor, and repeat the MMRM analysis
11) UPDRS part III vs PET correlation analyses after subtracting the tremor subscale score from the part III score
12) Subgroup analysis for the primary endpoint with demographic and baseline disease characteristics (univariate and multivariate); Conduct key primary endpoint analysis using the dataset of subjects with a H-Y score = 2.0; years since diagnosis; years since first symptom; posterior putamen PET K_i (or K_{oc}?)at BL
13) UPDRS part III summary/graphical analysis (individual data) of all available data (BL to last) (Tim N)
14) Perform genotyping on all consenting subjects. (Alan to engage Prof Huw Morris as academic collaborator)

[^{18}F]DOPA PET

Note: UBC is generating numbers based on reference region of Occipital Cortex (Koc) rather than the formal Ki (plasma-derived input function). Any comparison to literature values needs to account for this difference in methodology.

1) Thoroughly review all of the PET data including the analysis techniques applied, processing pipeline and values generated.
2) Review all PET acquisition data (QC process, etc.) with Cardiff directly
3) Confirm PET scanner information and identify any issues with scans.
4) Review all baseline Koc values to ensure that they lie within the range expected for the subject's clinical score (H&Y and UPDRS part III OFF)
5) Assess subjects (based on blinded video recordings) to determine if clinical manifestation is impacting left (L), right (R) or both putamen; then correlate with the L and R posterior putamen data – or modified region analysis from UBC/Renishaw maps (see below).
6) Arrange TC/Webex with Pfizer/Renishaw for week of 7/25 to review methodology for generation of distribution clouds, based on T1 Gd-based imaging - include Alan Whone. Have Renishaw generate distribution volumes based on the "Matthias map" and transfer to UBC.
7) UBC will generate SPM images demonstrating differences in Koc between baseline and Week 40 data – should be available within two weeks.

This Document is Company Confidential.

8) Once available, co-register the Gd distribution volumes from Renishaw onto the SPM images to see if this further focuses the impacted region(s) of the putamen.
9) If Gd distribution volume data are going to take longer than expected to generate, we can also constrain SPM images by the new VOI maps generated by UBC in which they have drawn a (long elliptical) region that covers the posterior 2/3 of the dorsal half of the putamen (regions B & C of the "Matthias map").
10) Recommended to UBC that they attempt to measure Koc in the substantia nigra; can achieve this base on coregistered MRI images. This will allow us to evaluate if there is an impact on [^{18}F]DOPA uptake further down the nigrostriatal axon.

Potential future studies

If it is determined that there is a compelling scientific rationale for continuing to treat the current study subjects beyond the planned end of Study 2797, there are two levers which can be pulled to investigate the possible explanations for the discrepancy between the PET and clinical outcomes – duration and dose.

1) It was agreed that a proposed protocol synopsis and associated budget would be prepared now for a dose-finding study using the existing subjects.
2) The potential for NBT to continue as the study sponsor will also be investigated at this time.

10.2 CALCULATION OF UPDRS MOTOR SCORE SUBSCALES

Six subscales of the UPDRS motor score (part III) are defined based on sums of individual items. The subscales are as follows:

Subscale 1: Tremor (range 0-28), including items 20 (rest tremor for head and each of the four extremities) and 21 (postural/action tremor in each hand)

Subscale 2: Rigidity (range 0-20), including item 22 (rigidity in the neck and each of the limbs)

Subscale 3: Bradykinesia (range 0-36), including items 23-26 and 31

Subscale 4: Postural instability/gait disorder (PIGD) (range 0-8), including items 29 (gait) and 30 (postural stability)

Subscale 5: Other (range 0-16), including items 18 (speech), 19 (facial expression), 27 (arising from chair) and 28 (posture)

Subscale 6: UPDRS motor excluding tremor (range 0-80): this is the sum of subscales 2 through 5.

10.3 DEFINITION OF RESPONDER

This Document is Company Confidential.

A total of five different responder definitions will be used.

Two different thresholds will be used to define OFF state UPDRS motor score responders:

1) subjects who have a UPDRS Part III improvement (decrease) from baseline to Week 40 of ≥ 5 points,

2) subjects who have a UPDRS Part III improvement (decrease) from baseline to Week 40 of ≥ 10 points.

PD diary responders are defined as subjects who reported a ≥ 1 hour improvement (increase) in good quality ON-time from baseline to Week 40.

In addition, two composite responder definitions combine the OFF state UPDRS motor score requirements with the PD diary requirements. Composite 1 (C1) responders are those who achieve both a ≥ 1 hour improvement (increase) in good quality ON-time from baseline to Week 40 AND who have a UPDRS Part III improvement (decrease) from baseline to Week 40 of ≥ 5 points. Composite 2 (C2) responders are those who achieve both a ≥ 1 hour improvement (increase) in good quality ON-time from baseline to Week 40 AND who have a UPDRS Part III improvement (decrease) from baseline to Week 40 of ≥ 10 points.

10.4 DEFINITION OF L-DOPA RESPONSIVENESS

L-Dopa responsiveness is defined as the percent improvement in UPDRS motor score (part III) following an L-Dopa challenge. This will be calculated as:

$$\frac{(UPDRS(OFF) - UPDRS(ON))}{UPDRS(OFF)} * 100$$

10.5 IDENTIFICATION OF PREDOMINANTLY AFFECTED BODY SIDE

Patients with Parkinson's disease most often have asymmetric motor features both at onset and throughout the course of their disease, with one side of the body being characteristically more affected by the cardinal features of tremor, rigidity and bradykinesia.

Each trial subject underwent a detailed clinical consultation and neurological examination, performed by the principal investigator, as part of their Screening Visit 2 assessments.

Body side predominance was established through systematic review of the clinical notes, specifically the initial entries by the PI regarding:

- Parkinson’s disease history including first symptom,
- patient-reported motor symptoms and

This Document is Company Confidential.

- clinical examination detailing limb predominance of tremor, rigidity and bradykinesia.

10.6 LIST OF GENES INCLUDED IN TARGETED GENOTYPING

Pending patient consent, samples will be taken for DNA extraction and genotyping on the following genes:

Disease/ PARK Designation	Gene, location	Inheritance	Phenotype
PARK-parkin (PARK2)	*parkin,* 6q25.2–q27	AR	Early-onset PD
PARK-DJ1 (PARK7)	*DJ-1,* 1p36.23	AR	Early-onset PD
PARK-PINK1 (PARK6)	*PINK1,* 1p36.12	AR	Early-onset PD
PARK-DNAJC6 (PARK-19)	*DNAJC6,* 1p31.3	AR	Early-onset PD
PARK-SNCA (PARK1/PARK4)	*SNCA,* 4q22-1	AD	Early-onset PD
PARK-LRRK2 (PARK8)	*LRRK2,* 12q12	AD	Classical PD
PARK-VPS35 (PARK17)	*VPS35,* 16q11.2	AD	Classical PD
PARK-EIF4G1 (PARK18)	*EIF4G1,* 3q27.1	AD	Classical PD
PARK-CHCHD2	*CHCHD2,* 7p11.2	AD	Classical PD
GBA heterozygous mutations (increased susceptibility to PD)	*GBA,* 1q22	AD	Classical PD
Dopamine transporter type 1 gene variants modifying response to L-dopa treatment	*SLC6A3,* 5p15.33	N/A	Pharmacogenetic correlations in PD patients

This Document is Company Confidential.

10.7 DATA TO BE PROVIDED BY MEDGENESIS/PRA

The following data required for the above analyses will be provided to Pfizer by MedGenesis/PRA:

1) UPDRS motor score (part III) subscales 1-6
2) L-Dopa responsiveness
3) Responder status based on quality ON time from the PD Diary
4) Predominantly-affected body side
5) Genotype (pending availability)
6) New F-DOPA ROI Kocc
7) New, updated Volume data

This Document is Company Confidential.
